# Peptide *Carbo*cycles: From −SS– to −CC– via a Late-Stage “Snip-and-Stitch”

**DOI:** 10.1021/acscentsci.2c00456

**Published:** 2022-10-28

**Authors:** Samuel Gary, Steven Bloom

**Affiliations:** Department of Medicinal Chemistry, University of Kansas, Lawrence, Kansas66045, United States

## Abstract

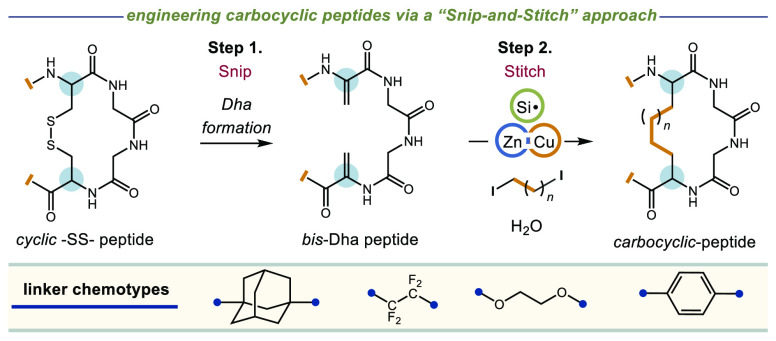

One way to improve the therapeutic potential of peptides is through cyclization. This is commonly done using a disulfide bond between two cysteine residues in the peptide. However, disulfide bonds are susceptible to reductive cleavage, and this can deactivate the peptide and endanger endogenous proteins through covalent modification. Substituting disulfide bonds with more chemically robust carbon-based linkers has proven to be an effective strategy to better develop cyclic peptides as drugs, but finding the optimal carbon replacement is synthetically laborious. We report a new late-stage platform wherein a single disulfide bond in a cyclic peptide can serve as the progenitor for any number of new carbon-rich groups, derived from organodiiodides, using a Zn:Cu couple and a hydrosilane. We show that this platform can furnish entirely new carbocyclic scaffolds with enhanced permeability and structural integrity and that the stereochemistry of the new cycles can be biased by a judicious choice in silane.

## Abstract 2

Late-stage skeletal editing of peptide −SS− bonds through bis-dehydroalanine intermediates provides ready access to chemically unique −CC− carbocycles with enhanced biopharmaceutical properties.

## Introduction

**Figure 1:**
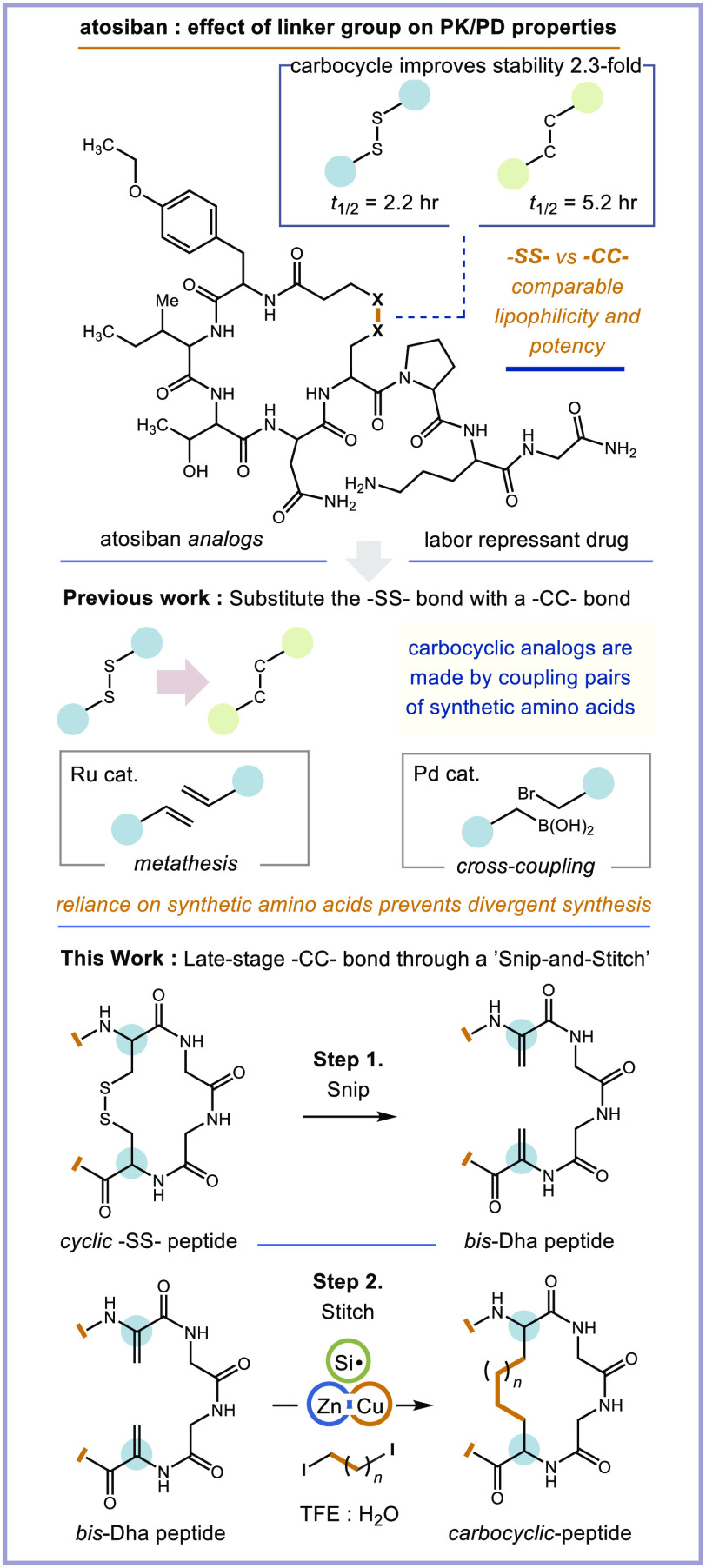
Contemporary carbocyclic peptide formation and proposed “Snip-and-Stitch” strategy.

Cyclic peptides are among the most popular modalities for new peptide therapeutics.^1−3^ Cyclization enhances the metabolic stability of linear peptides to enzymatic hydrolysis.^4−8^ Cyclization also enhances cell permeability by reducing overall polarity and hydrogen bonding^9−13^ and potency by stabilizing peptide conformations that better complement the target binding site.^14−17^ Many peptide therapeutics take advantage of disulfide bonds between two cysteine residues (−SS−) to form the peptide cycle. However, the susceptibility of −SS– bonds to reductive ring-opening can be a metabolic liability. Disulfide bonds are redox sensitive and are prone to rapid reductive cleavage, which shortens their half-life *in vivo*.^18,19^ Disulfide linkages can also participate in disulfide-exchange reactions with glutathione or cellular proteins having free thiol groups, resulting in protein modification and the generation of neo-antigens.^20,21^ Hence, modern cyclic peptide drugs aim to replace labile −SS– bonds with chemically benign −CC– linkages (as shown for atosiban, Figure 1).^22^ This can be accomplished using scaffold-based cyclization technologies such as ring closing metathesis (RCM)^23^ or palladium cross-coupling (Heck or Suzuki),^24−26^ wherein unique pairs of synthetic amino acids are first positioned in the peptide by solid-phase peptide synthesis (SPPS) and then coupled together to form a single *carbo*cyclic product. Alternatively, one can start from an orthogonally protected diamino diacid linker and use amide bond forming reactions to lay in the remaining peptide around, from end-to-end, this synthetic core unit.^27−30^ Besides the expense and limited availability of synthetic amino acids,^31^ one caveat to these approaches is that the new −CC– linkage can alter the three-dimensional structure of the peptide,^32^ impacting its bioactivity. Identifying −CC– replacements that maximize biopharmaceutical properties (e.g., half-life) without diminishing drug potency can, therefore, require numerous carbocyclic analogs, each requiring a separate multistep SPPS to complete. A carbocyclization platform, through direct −SS– skeletal editing, could offer a highly modular way to pan out the best carbocyclic analogs for any −SS– cyclic peptide and could provide a general route to access *entirely new* carbocyclic frameworks from a single disulfide-containing peptide.

## Experimental Section

We envisaged that we could cleave (i.e., “*Snip*”) the −SS– bond of a cyclic disulfide-containing peptide into a pair of electrophilic dehydroalanine (Dha) residues. Then, by combining the bis-Dha peptide with an appropriate bridging group (e.g., a bis-radical, bis-anion, or bis-metallic species), we could insert (i.e., “*Stitch*”) a library of new linker groups in place of the original −SS– bond in one convenient step. To test our proposed diversification strategy, we needed to find (1) a general method for converting the −SS– bond of a macrocyclic peptide into two Dha residues, (2) a functional precursor for inserting diverse linker chemotypes in place of the −SS– bond, and (3) chemistry to mediate bridge installation. We selected terlipressin, an −SS– containing cyclic dodecapeptide previously evaluated in the clinic to treat hepatorenal syndrome type-1,^33^ as a model substrate for designing our *Snip-and-Stitch* strategy. We found that terlipressin could be converted to the unconstrained *bis*-Dha peptide, Dha_2_-terlipressin, with the combination of methyl 2,5-dibromopentanoate^34^ and TCEP in good yield (>60% isolated). Other reagents for bis-Dha formation were ineffective; see the Supporting Information p S20 for complete details. Next, we sought to identify reagents and chemistries to link the two Dha fragments together through unique tethering groups. The challenge here is that the overall process of macrocyclization involves the formation of two different C–C bonds, one at each Dha residue, and a net input of up to four electrons. Thus, we explored three different chemical approaches for bridge installation, namely, photoredox catalysis (single-electron transfer chemistry), transition metal catalysis (the use of organometallic reagents as bis-anion equivalents), and electrochemistry (the generation of open-shell or bis-anionic intermediates through cathodic reduction or anode-to-cathode cycling with a redox mediator). These approaches and the intermediates they generate have been shown to transform Dha residues in peptides and proteins into α-amino acids.^35−38^ To evaluate each platform, we prepared seven distinct linker chemotypes (Table 1, compounds **A**–**G**), each linker consisting of a central four-carbon fragment that is capped with a unique pair of functional groups that serve as latent handles for radical or anion generation (viz., **X**[CH_2_]_4_**X**). Optimal conditions for converting each linker chemotype to the corresponding radical or anionic intermediate were selected on the basis of a comprehensive survey of the literature.^39−46^ Linkers were evaluated in 9:1 H_2_O:DMSO (for transition metals), 1:1 H_2_O:DMSO (for photoredox catalysis), or 1:1 H_2_O:MeCN (for electrochemistry) at 1 mM. A brief synopsis of our results is shown in Table 1. See the Supporting Information pp S31–S50 for all entries.

**Table 1:**
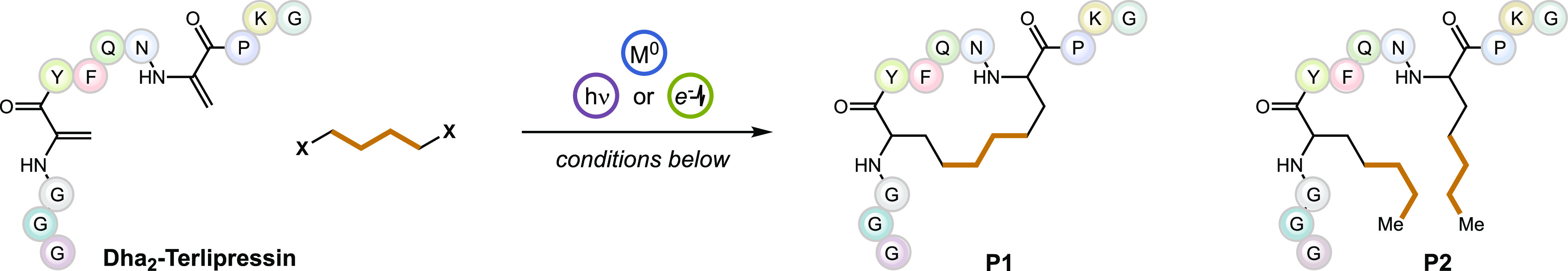

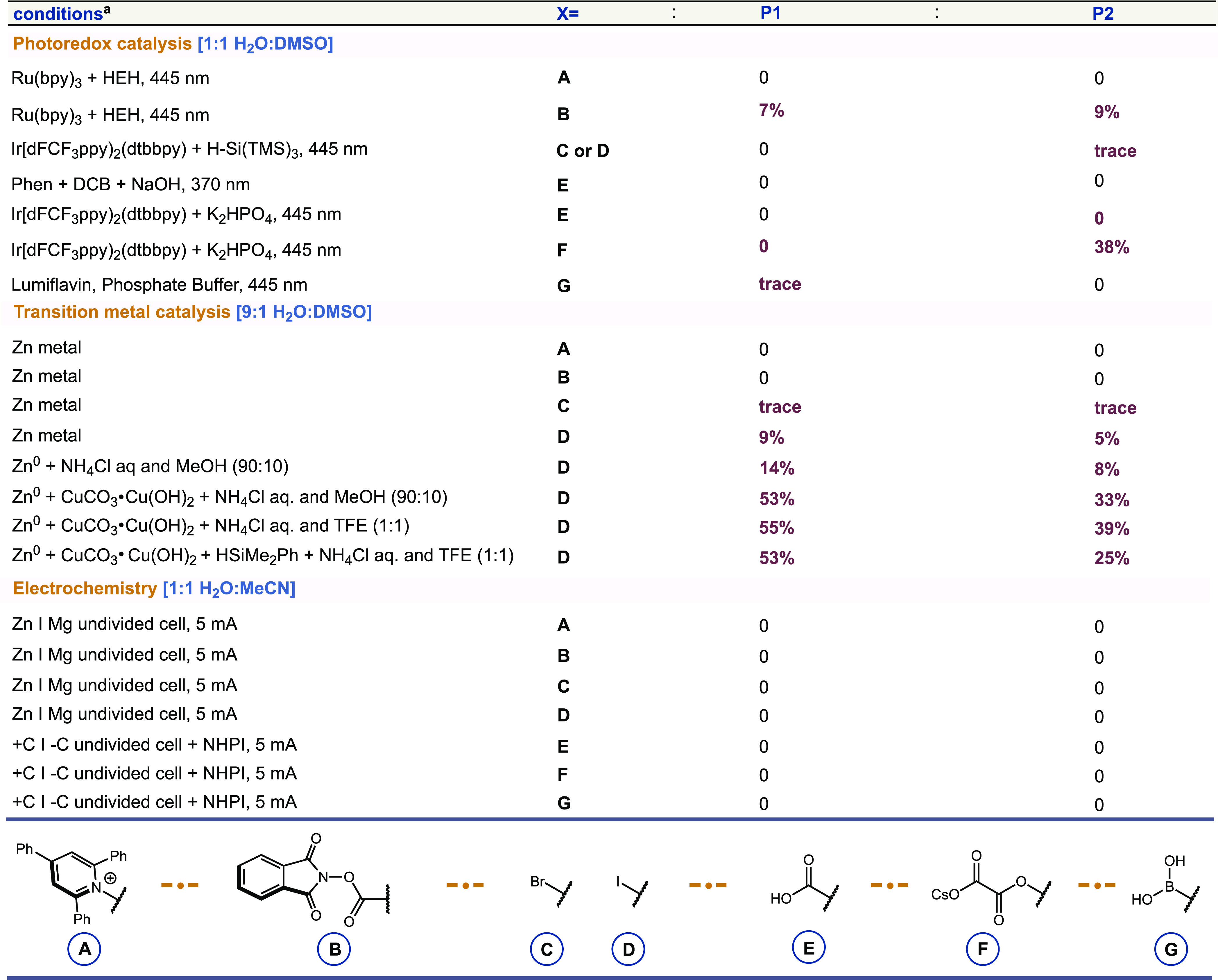
Survey of Reaction Conditions and Synthetic Platforms for Peptide Macrocyclization

a See the Supporting Information pp S31–S56 for full experimental details and additional reaction conditions.

### Safety Statement

No unexpected or unusually high safety hazards were encountered.

## Results and Discussion

We surveyed over five-hundred unique reaction conditions: seven different linker chemotypes over three different synthetic platforms. We identified two sets of conditions for converting Dha_2_-terlipressin to the desired −[CH_2_]_4_– carbocyclic peptide (Table 1, compound **P1**): (1) 1,4-diiodobutane in the presence of zinc metal (9% conversion) and (2) the combination of Ru(bpy)_3_ photocatalyst, Hantzsch ester (HEH), and reagent **B** (7% conversion). Other organometallic or photochemical methods gave varying amounts of a dialkylated byproduct **P2**, failed to react, or afforded considerable amounts of unidentifiable byproducts by HRMS analyses. Electrochemical methods performed on Dha_2_-terlipressin or on a test Dha monomer, Ac-Dha-OMe, formed only large amounts of alanine. We attempted to independently optimize both our zinc-mediated and Ru(bpy)_3_ photocatalyzed protocols to improve the yield of the carbocyclic peptide. We were unable to improve the yield of our photocatalyzed reaction beyond 10% conversion. Performing our zinc-mediated protocol in a solvent of 1:1 TFE:NH_4_Cl (sat. aq.) and adding basic copper carbonate (CuCO_3_·Cu(OH)_2_) to the reaction improved the conversion to carbocyclic peptide to 40% as a 1:1:1:1 mixture of diastereomers.^47−53^ A stoichiometry of 12:12:1 of Zn:Cu:diiodide gave an optimal 55% conversion to **P1** and 39% to **P2** with complete consumption of Dha_2_-terlipressin. See the Supporting Information pp S50–S56 for complete optimization details.

**Figure 2:**
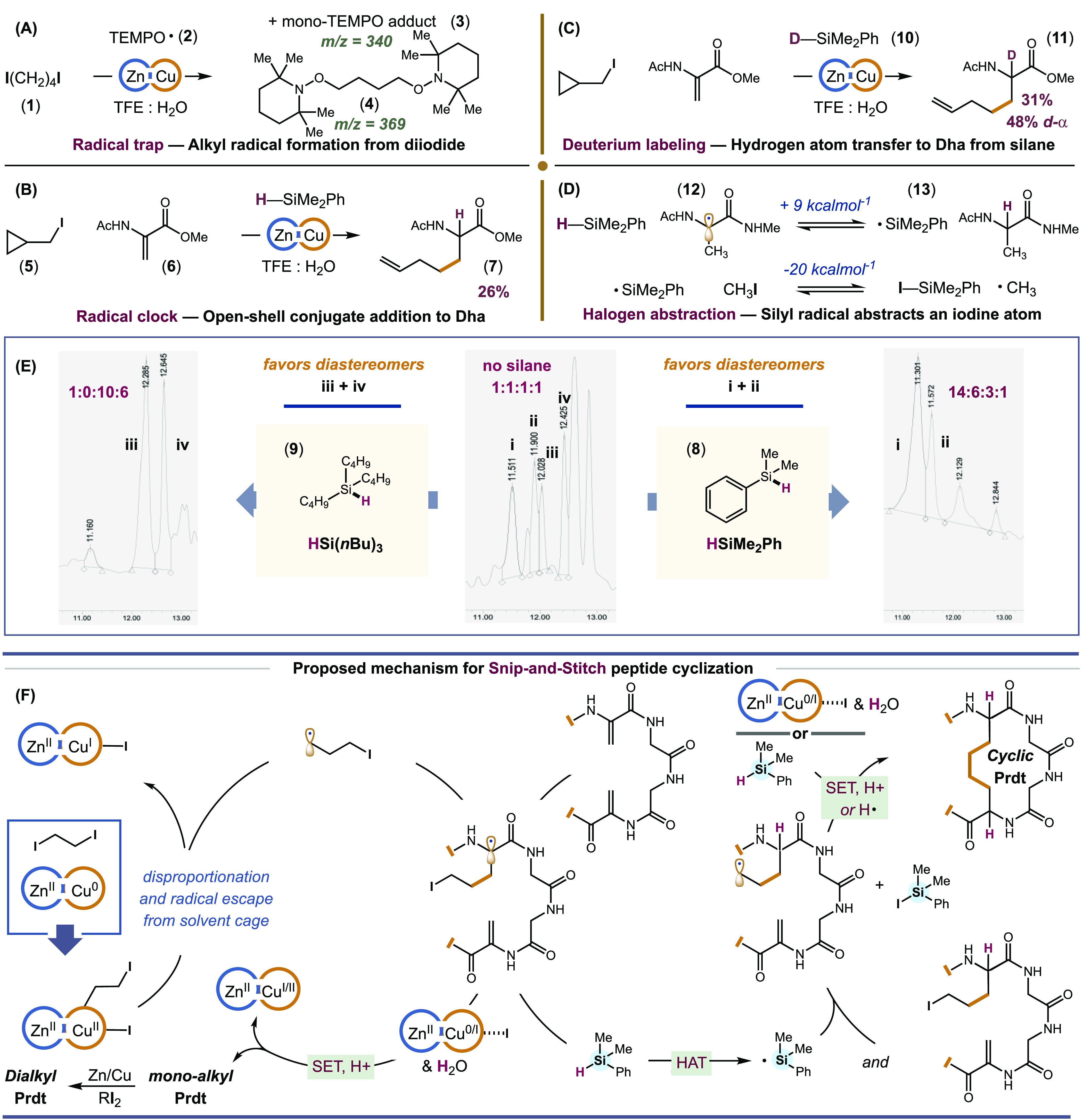
Mechanistic studies and proposed mechanism. (A–D) Mechanistic and computational studies. (E) The influence of hydrosilanes on diastereoselectivity of setmelanotide cyclization. (F) Proposed mechanism of “Snip-and-Stitch” cyclization. See the Supporting Information pp S106–S117 for full experimental details.

One drawback of the above protocol is that it forms a mixture of the desired carbocyclic peptide and a dialkylated byproduct wherein each Dha residue reacts with a single alkyl diiodide. To improve carbocyclic product selectivity, we attempted to gain some mechanistic insight into our reaction. Combining CuCO_3_·Cu(OH)_2_, Zn^0^, and NH_4_Cl in water afforded a black insoluble material, presumably a Zn^II^:Cu^0^ couple. This material slowly turned blue upon exposure to air, characteristic of reoxidation to Cu^II^ ions. We imagined that the copper metal in our couple might be able to reduce our diiodide (*E*_1/2_^red^ = −1.44 V vs SCE for I(CH_2_)_2_I in DMF)^54^ to an alkyl radical via an outer-sphere electron transfer mechanism. However, this seems unlikely given the modest reducing capacity of Cu^0^ (Cu^0^/Cu^I^ = −0.26 V vs SCE in H_2_O, pH 6.82)^55^ and would necessitate that Zn^II^ participates as a *strong* Lewis acid to lower the barrier to outer-sphere electron transfer. Another possibility is that a small amount of very reducing Rieke zinc is generated in situ. While we cannot rule out these possibilities, we propose that Cu^0^ can oxidatively insert, albeit slowly,^56^ into the C–I bond of the diiodide, forming an alkyl–Cu^II^–I intermediate.^57^ This process should be enthalpically feasible due to the cleavage of the weak C–I bond (∼50 kcalmol^–1^) and formation of a comparable Cu–I bond (∼47 kcalmol^–1^)^58−60^ in addition to a Cu^II^–C bond, reported as ∼33 kcalmol^–1^ for ClCu^II^–C_3_H_7_.^61^ The Cu^II^ organometallic can disproportionate to give an alkyl radical and Cu^I^–I.^53^ To probe the formation of a free alkyl radical, we combined our Zn:Cu couple with 1,4-diiodobutane (**1**) and TEMPO· (**2**) in H_2_O:TFE. By LC-MS analysis, we observed complete conversion of the diiodide to a mono- and di-TEMPO adduct (**3** and **4**, respectively), wherein one or both iodine atoms of the diiodide were replaced by TEMPO, Figure 2A. In a separate set of experiments with cyclopropylmethyl iodide (**5**) as a radical clock and Ac-Dha-OMe (**6**) as a surrogate electrophile for our bis-Dha-peptide, we obtained only the ring-opened product (**7**) under our reaction conditions (26% ^1^H NMR yield), Figure 2B. The formation of a free alkyl radical therefore seems plausible, and its generation via disproportionation of an intermediate Alkyl-Cu^II^–I is probable. The radical intermediate then adds to a single Dha residue in our bis-Dha peptide to give a monoalkylated species, wherein one iodine atom is retained on the linker. From here, one of two possible scenarios can occur: (1) the second iodine atom is cleaved to a primary alkyl radical, which adds to the second Dha residue in our peptide, forming the carbocyclic product, or (2) another equivalent of diiodide in solution is reduced to a radical intermediate, which adds to the remaining Dha residue in our peptide, forming the dialkylated product after both iodine atoms have been reductively excised. The fact that we observe the cyclic and dialkylated products in similar proportions suggests that both pathways are likely.

A reagent that could mediate ring closure might bias our reaction in favor of cyclization. An ideal reagent would homolytically cleave the remaining C–I bond on the linker and facilitate the formation of a new C–C bond at the second Dha residue in our peptide. To satiate these requirements, we investigated hydrosilanes. The α-carbonyl radical, formed after addition of an alkyl radical to a Dha residue, has a BDE of ∼87 kcalmo1^–1^ (calculated for Ac-Ala-NHCH_3_ at the B3LYP 6-311+G** level of theory). The Si–H bond for some hydrosilanes is weaker than the α-carbonyl-H bond,^62^ suggesting that a favorable hydrogen-atom transfer (HAT) from the hydrosilane to the α-carbonyl radical can occur, yielding an α-amino acid and a silyl radical. The silyl radical can then abstract the remaining iodine atom^63^ from the linker, affording a nucleophilic carbon-centered radical proximal to, and poised to react with, the second Dha residue in our peptide (Si–I BDE ∼ 80 kcalmol^–1^ vs C–I BDE ∼ 54 kcalmol^–1^).^64^ Experimentally, we found that hydrosilane HSiMe_2_Ph (**8**) improved product selectivity in favor of cyclization (Table 1). The overall conversion was comparable, but the formation of mono- and dialanine peptides (from Dha residues) was now observed in addition to smaller amounts of dialkylated byproduct.

To test the ability of phenyldimethylhydrosilane (H-SiMe_2_Ph) to quench an α-carbonyl radical through hydrogen-atom transfer and to generate an alkyl radical via iodine atom abstraction, we performed additional mechanistic experiments. While it is well-known that silyl radicals can abstract halogen atoms,^46,65−67^ the ability of a hydrosilane to transfer a hydrogen atom to an α-carbonyl radical in an amino acid has not been observed previously. We used Ac-Dha-OMe acceptor and cyclopropylmethyl iodide to separately investigate each process. To assess hydrogen atom transfer (HAT), we combined our Dha acceptor and cyclopropylmethyl iodide with our Zn:Cu couple and a nonexchangeable (with protonaceous solvent) deutero-silane D-SiMe_2_Ph (**10**), Figure 2C. We observed the ring opened amino acid product with an α-deuterium atom in 31% yield (48% *d*-content), compound **11**. When the reaction was performed with the hydrosilane congener H-SiMe_2_Ph in D_2_O:TFE, a 45% yield (65% *d*-content) of the ring opened product with an α-deuterium atom was obtained. This suggests that HAT from H-SiMe_2_Ph to Dha is viable but is not the only source of hydrogen in our reaction. To assess iodine abstraction, we removed Zn:Cu from the reaction and instead used one of a myriad of reagents to convert H-SiMe_2_Ph to halidophilic ·SiMe_2_Ph. Unfortunately, the thermal and photolytic instability of our iodide, admixed with competitive halogen abstraction, complicated our studies, and we were not able to observe the ring opened amino acid product or I-SiMe_2_Ph (Supporting Information pp S110–S112). However, computations suggest that the generation of ·SiMe_2_Ph (**13**) by HAT to an α-carbonyl radical (**12**) followed by iodine atom abstraction to give I–SiMe_2_Ph should be a favorable process overall (−11 kcalmol^–1^, Figure 2D). One explanation for the competitive formation of the dialkylated product in our peptide cyclization reaction could be that electron transfer from our Zn:Cu couple (to form an α-carbanion at Dha) outcompetes HAT from the hydrosilane in some instances. This is supported by our experimental observation that D_2_O is able to incorporate deuterium atoms into our ring-opened amino acid product.

We prepared and tested a second bis-Dha peptide, Dha_2_-setmelanotide. Setmelanotide (SMT) is a cyclic −SS– containing octapeptide that was approved by the FDA in 2020 to treat genetic-associated obesity.^68^ Under our optimized cyclization conditions with 1,5-diiodopentane, SMT produced all four possible cyclic peptide diastereomers in an ∼14:6:3:1 ratio with two diastereomers being heavily favored. All four diastereomers were produced in equal amounts when H-SiMe_2_Ph was removed from the reaction (Figure 2E). This unexpected result led us to investigate the effect of other hydrosilanes in our reaction (see the Supporting Information p S54 and pp S112–S118 for all experimental data). For SMT, we found that hydrosilanes (30 equiv) having Si–H BDE ≥ 96 kcalmol^–1^ (determined computationally at the B3LYP 6-311+G** level of theory) were best at biasing cyclization. Hydrosilanes with BDEs < 96 kcalmol^–1^ gave more dialkylated product. Although many of the hydrosilanes we examined could enforce cyclization, they did not affect diastereoselectivity. Interestingly, tri-*n*-butylhydrosilane (**9**; H–Si(*n*Bu)_3_) afforded only three of the four cyclic SMT diastereomers in an ∼1:0:10:6 ratio, favoring two diastereomers that were not produced in significant quantities when H-SiMe_2_Ph was used as additive. Between H-SiMe_2_Ph and *n*-tributylhydrosilane, we can bias cyclization in favor of two different sets of the four possible cyclic peptide diastereomers of SMT (Figure 2E). The capacity of achiral hydrosilanes to bias the stereochemistry of amino acids formed by radical addition to a prochiral dehydroalanine residue has not been reported previously, and this has untapped potential for synthetic peptide chemistry. This also lends further support to the important role of hydrosilane in our proposed cyclization mechanism, as depicted in Figure 2F. Because of its unique ability to bias our cyclization reaction and its cleaner reaction profile, we proceeded in our studies with H-SiMe_2_Ph as additive.

To obtain sufficient quantitates of our SMT carbocyclic peptide, we performed our diversification protocol on a scale of 5 or 10 mg of Dha_2_-setmelanotide. The efficiency of our reaction was not affected on either scale when 1,5-diiodopentane was used as linker. We explored three different methods to isolate the carbocyclic product formed in our scaled reaction. We elected not to use high-performance liquid chromatography, a sufficient but rather slow purification strategy, in lieu of exploring alternative strategies that would be faster and more available to most synthetic and medicinal chemists. We examined (1) liquid-phase extraction using an aqueous solution of saturated ammonium sulfate and various organic solvents,^69^ (2) solid-phase extraction (SPE) using a Waters Oasis HLB cartridge,^70^ and (3) liquid-phase flash chromatography. Of these, flash chromatography afforded the cyclic product (and its individual diastereomers) in high purity (>90%), using an eluent of H_2_O/0.1%TFA:EtOH/0.1%TFA. This strategy is particularly useful when purifying large quantities of carbocyclic peptides that would be unsuitable for HPLC. (The cyclic products could not be separated using H_2_O:MeCN or H_2_O:MeOH as eluents.) For these reasons, we used C18 flash chromatography to purify our cyclic peptides.

We assessed other diiodide linkers for bis-Dha peptide cyclization using Dha_2_-setmelanotide. Primary alkyl diiodides (I(CH_2_)_*n*_I, *n* = 4–8 carbons; Table 2 compounds **14**–**18**) were first evaluated as −SS– replacements. These substrates worked well in our diversification platform, furnishing **C**_**4–8**_-**SMT** carbocyclic analogs in 24–38% conversion. Diiodomethane (**19**) afforded the methylene bridged (ring-contracted) carbocycle in 22% conversion and several intractable byproducts. 1,2-Diiodoethane (**20**), prone to ethylene gas formation, did not furnish the desired macrocycle. Only unreacted Dha_2_-setmelanotide was recovered. Diiodides containing an ethylene glycol spacer (**PEG-SMT**; **21**), a perfluoroalkyl unit (**F_12_-SMT**; **22**), and an adamantane polycycle (**23**) afforded the desired macrocyclic products in good overall conversions (28–47%). Finally, cyclohexyl diiodide (**24**), benzylic diiodide (**25**), and phenyl diiodide (**Ph-SMT**; **26**) furnished new macrocyclic products in useful conversions (17–35%), Table 2. It is important to point out that our products were all isolated (0.09–2.91 mg) as a mixture of diastereomers in an average amount of 0.61 mg at >90% purity. However, individual peptide diastereomers can be separated by our flash chromatography procedure when desirable. Our lab has shown that mixtures of peptide diastereomers isolated in greater than 0.05 mg and exceeding 65% purity are more than sufficient for completing accurate biochemical experiments.^70^ The data obtained from these assays compares well with experiments performed using single diastereomer products. As a word of caution, using mixtures of peptide diastereomers is very useful when surveying large numbers of peptides for relative biochemical activity. Optimal substitutions generally lead to order of magnitude enhancements.^71−73^ However, separating the mixture and testing individual diastereomers is necessary to ascribe physiochemical properties to a specific peptide sequence. This becomes rather important when dealing with certain peptides whose molecular mechanism, i.e., ability to self-assemble^74^ or penetrate cell membranes,^75^ depends on its innate chirality.

**Table 2:**
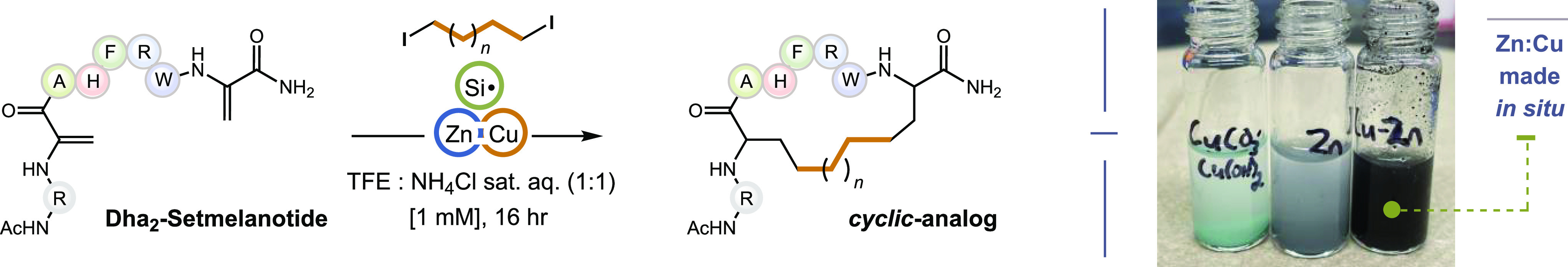

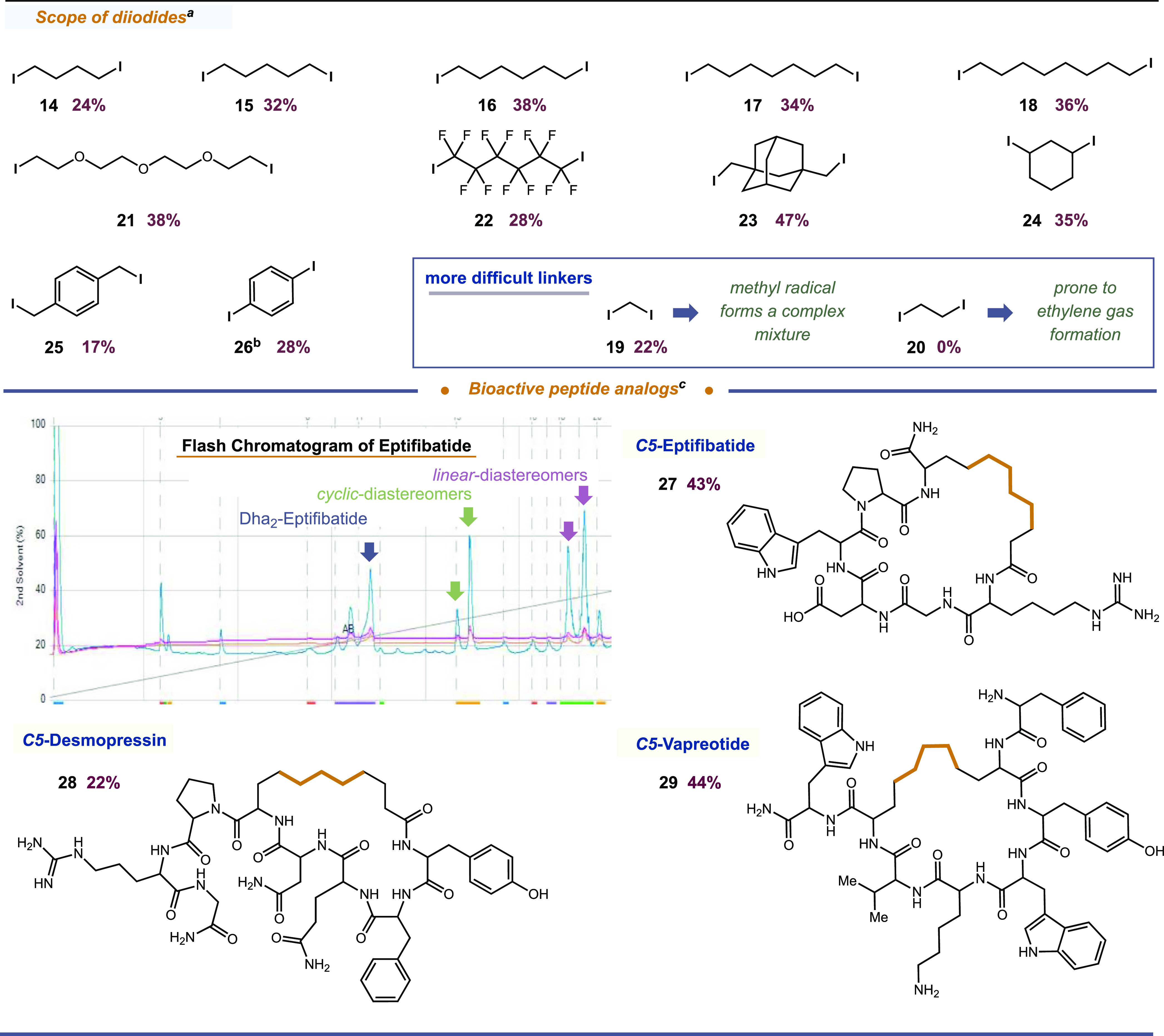
Scope of Diiodides for Setmelanotide Cyclization and Survey of Bioactive Peptides for “Snip-and-Stitch” Cyclization

a All reactions were performed using 951 nmol of Dha_2_-setmelanotide, 5 equiv. of diiodide, 30 equiv. of CuCO_3_·Cu(OH)_2_, 60 equiv. of zinc mesh, and 10 equiv. of HSiMe_2_Ph in a solution of 1:1:2 sat. aq. NH_4_Cl:H_2_O:TFE (1 mM) for 16 h. Yields are reported as % conversion to cyclic products at 280 nm.

b Reaction performed using 10 equiv. of diiodide.

c All reactions were performed using 5.0 mg of Dha_2_-peptide, 5 equiv. of diiodide, 30 equiv. of CuCO_3_·Cu(OH)_2_, 60 equiv. of zinc mesh, and 10 equiv. of HSiMe_2_Ph in a solution of 1:1:2 sat. aq. NH_4_Cl:H_2_O:TFE (1 mM) for 16 h. Yields are reported as % conversion to cyclic products at 214 nm.

We examined other bioactive peptides, including several FDA approved peptide drugs for −SS– bond diversification. We assessed eptifibatide (platelet inhibitor),^76^ desmopressin (antidiuretic),^77^ and vapreotide (vasoconstrictor).^78^ For these studies, we used 1,5-diiodopentane as a standard linker. We found that bis-Dha analogs of each peptide could be formed and reacted under our diversification conditions to afford new carbocyclic peptides in conversions of 22–44%, Table 2 compounds **27**–**29**. The new cyclic products were readily isolated by flash chromatography, as shown for eptifibatide in Table 2. For eptifibatide and desmopressin, the C-terminal thiol is converted to an acrylamide rather than a prochiral Dha residue. Hence, only two cyclic peptide diastereomers are generated in these cases. While our results show that our cyclization method can fashion head-to-tail (eptifibatide), side chain-to-side chain (vapreotide and terlipressin), and head-to-side chain (desmopressin) peptide cycles, they also provide us with additional insights into our cyclization mechanism. First, whereas the two diastereomers of the dialkylated product of eptifibatide are formed in an ∼1:1 ratio, the two diastereomers of the cyclic peptide are formed in ∼2:1 (see Table 2). This highly suggests that ring closure from one diastereomer is more favored over the other diastereomer, encouraging a single diastereomer product to form. Thus, in some cases, our cyclization method can favor a single diastereomer product. Second, while desmopressin and terlipressin have nearly identical sequences, including the positioning of their Dha residues, desmopressin cyclizes >10-fold more efficiently with almost no dialkylated byproduct being observed. Thus, intrinsic geometries may be more important for cyclization than any specific sequence or Dha loci.

**Figure 3:**
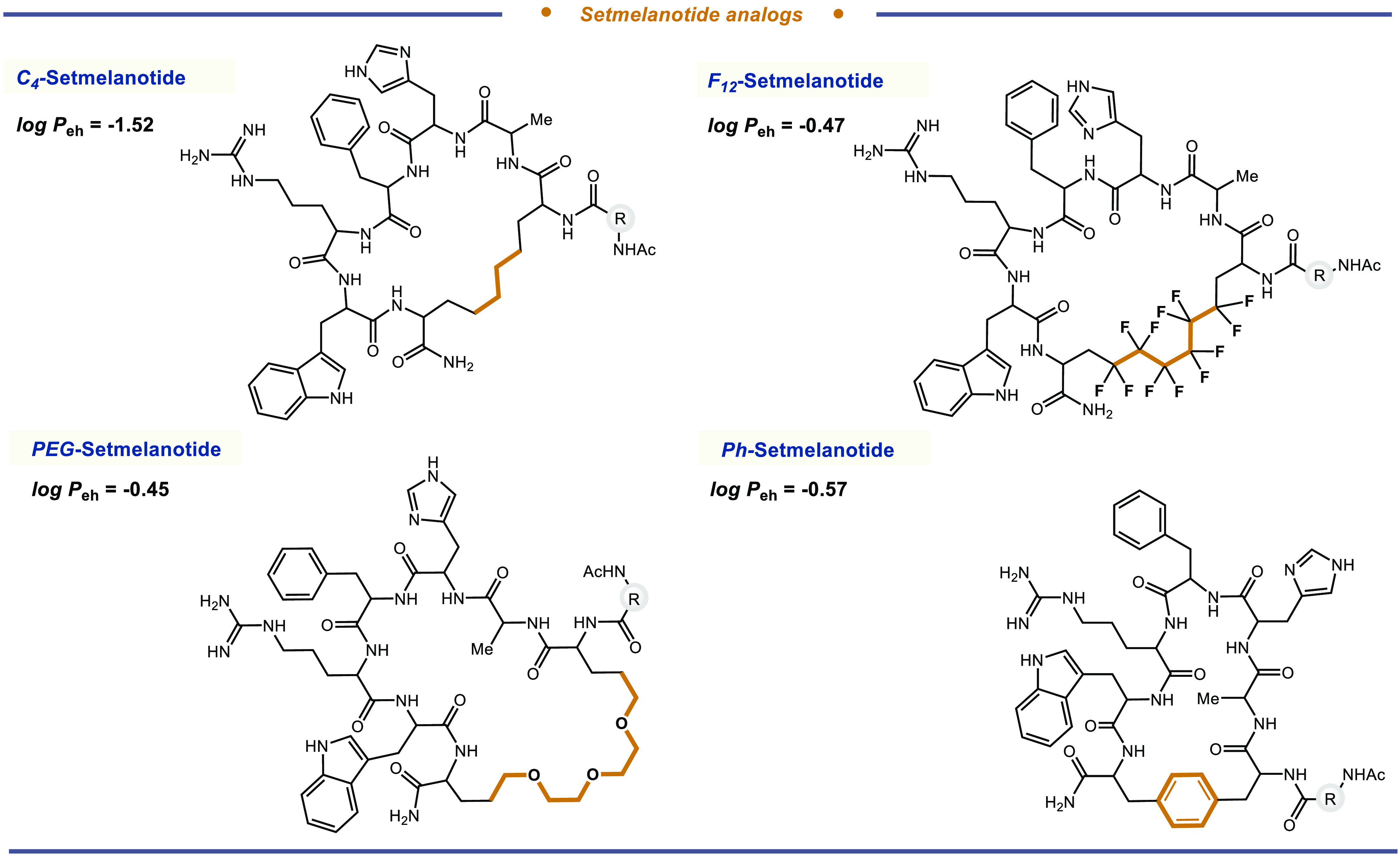
Permeability values (log *P*_eh_) for setmelanotide analogs. For setmelanotide, log *P*_eh_ = −1.16.

We next examined the effect of our linkers on the biopharmaceutical properties of setmelanotide. The capacity of setmelanotide (SMT) to regulate appetite depends on its ability to penetrate the blood–brain barrier and to activate melanocortin-4 (MC4) receptors in the brain.^79,80^ Replacing the −SS– bond of SMT with more lipophilic −CC– linkages could improve the CNS permeability of SMT and could also enhance its proteolytic stability. Hence, we determined the aqueous solubility, cellular permeability (log *P*_eh_), and general stability for some of our new carbocyclic analogs, namely, **C**_**4**_-**SMT**, **PEG**-**SMT**, **F**_**12**_-**SMT**, and **Ph**-**SMT**, and compared them to SMT. For aqueous solubility, we measured solvation in deionized water (pH 6 at 25 °C). We found that our carbocyclic peptides were completely soluble at a concentration of 1 mg of peptide per 1 mL of water. To assess cell permeability, we measured the partition coefficient *P*_eh_ of our carbocyclic peptides in a mixture of ethylene glycol and heptane.^81−84^ Previous studies by Borchardt and co-workers showed that the permeabilities (log *P*_eh_) of peptides obtained using this partitioning system agreed well with permeabilities measured from an *in vitro* model of the blood–brain barrier and from physiologic saline to rat brain.^82^ Thus, log *P*_eh_ values are a good approximation of *in vivo* permeabilities. For reference, log *P*_eh_ of water immiscible (brain penetrable) toluene = 0.994, and log *P*_eh_ of water miscible (brain impenetrable) benzamide = −3.69. Common small molecule CNS drugs have log *P*_eh_ values between −1.5 and −2.5. Examples include zolantidine (log *P*_eh_ −1.47), clonidine (log *P*_eh_ −1.80), and antipyrine (log *P*_eh_ −2.28).^78^ For SMT, we determined log *P*_eh_ = −1.16, reflective of its good water solubility and mild blood–brain permeability. For our carbocyclic peptides **C**_**4**_-**SMT**, **Ph**-**SMT**, **F**_**12**_-**SMT**, and **PEG**-**SMT**, we measured log *P*_eh_ values of −1.52, −0.57, −0.47, and −0.45, respectively (Figure 3). Thus, many of our carbocyclic analogs should have improved (up to 2.6-fold) blood–brain penetrance *in vivo*. Finally, we determined the stability of our carbocyclic peptides to aqueous hydrolysis and to reductive cleavage by glutathione (GSH), an approximate measure of their plasma stability.^85^ GSH in the cytoplasm can reduce disulfide bonds to dicysteine peptides and can also cleave disulfide bonds through a disulfide exchange reaction.^18−21^ Hydrolysis of disulfide bonds has also been observed, and this can result in the loss of one or both sulfur atoms from the peptide and in several cleavage byproducts.^86−89^ All measurements were compared to setmelanotide (SMT). As expected, SMT underwent disulfide exchange (2% mixed disulfide after 24 h) in the presence of 1 equiv. of GSH at physiological conditions (pH 7.4, 37 °C). Increasing the amount of GSH to 10 equiv. afforded more of the mixed disulfide (17%). No disulfide exchange was observed in the case of our carbocyclic analogs, which lack a disulfide bond. To assess hydrolytic stability, we dissolved SMT in pH 8.5 water and incubated the solution at 50 °C for 16 h. The products were analyzed by LC-MS. We observed the loss of a single sulfur atom (−32 *m*/*z*) from SMT. Only 5% SMT remained intact. In comparison, our carbocyclic peptides were susceptible to racemization at pH 8.5, but the peptide cycles remained completely intact. The only “odd” result came in the case of **F_12_**-**SMT**, which underwent extensive protodefluorination, with up to four fluorine atoms being lost. No C-terminal deamidation was observed for any peptide. Our carbocyclic analogs are, therefore, less likely to lose their biological activity due to processes that cleave the peptide cycle. Taken together, our carbocyclic analogs display superior cellular permeability and general stability and also maintain good aqueous solubility, thus imparting them with pharmacokinetic properties unrivalled by ordinary −SS– cyclic peptides.

## Conclusions

In conclusion, we have developed a first-generation Snip-and-Stitch platform to routinely transform disulfide bonds into new C–C bonds. We demonstrate the ability of our newly minted platform to produce entirely new classes of peptide carbocycles with improved biopharmaceutical properties. Our mechanistic studies detail the multifarious role of a hydrosilane in guiding peptide cyclization and for achieving stereospecific outcomes.

## Supporting Information Available

Additional information regarding peptide and small molecule syntheses, reaction optimizations, experimental procedures and purifications, product characterization, and computational and spectral data (PDF)

## References

[ref1] Joo S. H. Cyclic peptides as therapeutic agents and biochemical tools. Biomol. Ther. (Seoul) 2012, 20, 19–26. 10.4062/biomolther.2012.20.1.019. 24116270 PMC3792197

[ref2] Choi J.-S.; Joo S. H. Recent Trends in Cyclic Peptides as Therapeutic Agents and Biochemical Tools. Biomol. Ther. (Seoul) 2020, 28, 18–24. 10.4062/biomolther.2019.082. 31597413 PMC6939695

[ref3] Vinogradov A. A.; Yin Y.; Suga H. Macrocyclic Peptides as Drug Candidates: Recent Progress and Remaining Challenges. J. Am. Chem. Soc. 2019, 141, 4167–4181. 10.1021/jacs.8b13178. 30768253

[ref4] Li X.; Wang S.; Zhu X.; Zhangsun D.; Wu Y.; Luo S. Effects of Cyclization on Activity and Stability of α-Conotoxin TxIB. Marine Drugs 2020, 18, 180 10.3390/md18040180. 32235388 PMC7230940

[ref5] Clark R. J.; Fischer H.; Dempster L.; Daly N. L.; Rosengren K. J.; Nevin S. T.; Meunier F. A.; Adams D. J.; Craik D. J. Engineering stable peptide toxins by means of backbone cyclization: Stabilization of the α-conotoxin MII. Proc. Natl. Acad. Sci. U. S. A. 2005, 102, 13767–13772. 10.1073/pnas.0504613102. 16162671 PMC1236553

[ref6] Ngo K. H.; Yang R.; Das P.; Nguyen G. K. T.; Lim K. W.; Tam J. P.; Wu B.; Phan A. T. Cyclization of a G4-specific peptide enhances its stability and G-quadruplex binding affinity. Chem. Commun. 2020, 56, 1082–1084. 10.1039/C9CC06748E. 31894763

[ref7] Chan L. Y.; Zhang V. M.; Huang Y.-h.; Waters N. C.; Bansal P. S.; Craik D. J.; Daly N. L. Cyclization of the Antimicrobial Peptide Gomesin with Native Chemical Ligation: Influences on Stability and Bioactivity. ChemBioChem. 2013, 14, 617–624. 10.1002/cbic.201300034. 23426877

[ref8] Rozek A.; Powers J.-P. S.; Friedrich C. L.; Hancock R. E. W. Structure-Based Design of an Indolicidin Peptide Analogue with Increased Protease Stability. Biochemistry 2003, 42, 14130–14138. 10.1021/bi035643g. 14640680

[ref9] Dougherty P. G.; Sahni A.; Pei D. Understanding Cell Penetration of Cyclic Peptides. Chem. Rev. 2019, 119, 10241–10287. 10.1021/acs.chemrev.9b00008. 31083977 PMC6739158

[ref10] Matsson P.; Doak B. C.; Over B.; Kihlberg J. Cell permeability beyond the rule of 5. Adv. Drug Delivery Rev. 2016, 101, 42–61. 10.1016/j.addr.2016.03.013. 27067608

[ref11] Beck J. G.; Chatterjee J.; Laufer B.; Kiran M. U.; Frank A. O.; Neubauer S.; Ovadia O.; Greenberg S.; Gilon C.; Hoffman A.; Kessler H. Intestinal Permeability of Cyclic Peptides: Common Key Backbone Motifs Identified. J. Am. Chem. Soc. 2012, 134, 12125–12133. 10.1021/ja303200d. 22737969

[ref12] Thansandote P.; Harris R. M.; Dexter H. L.; Simpson G. L.; Pal S.; Upton R. J.; Valko K. Improving the passive permeability of macrocyclic peptides: Balancing permeability with other physicochemical properties. Bioorg. Med. Chem. 2015, 23, 322–327. 10.1016/j.bmc.2014.11.034. 25533323

[ref13] Ernenwein D.; St. John S. E.; Stewart A. J.; Morimoto B. H.; Chmielewski J.; Lipton M. A. Structural studies and cyclization of the neuroprotective octapeptide NAPVSIPQ to improve cell permeability. Peptide Sci. 2020, 112, e24179 10.1002/pep2.24179.

[ref14] Xu S.; Li H.; Shao X.; Fan C.; Ericksen B.; Liu J.; Chi C.; Wang C. Critical Effect of Peptide Cyclization on the Potency of Peptide Inhibitors against Dengue Virus NS2B-NS3 Protease. J. Med. Chem. 2012, 55, 6881–6887. 10.1021/jm300655h. 22780881

[ref15] Tørfoss V.; Isaksson J.; Ausbacher D.; Brandsdal B.-O.; Flaten G. E.; Anderssen T.; Cavalcanti-Jacobsen C. d. A.; Havelkova M.; Nguyen L. T.; Vogel H. J.; Strøm M. B. Improved anticancer potency by head-to-tail cyclization of short cationic anticancer peptides containing a lipophilic β2,2-amino acid. J. Pept. Sci. 2012, 18, 609–619. 10.1002/psc.2441. 22933412

[ref16] Yousif A. M.; Minopoli M.; Bifulco K.; Ingangi V.; Di Carluccio G.; Merlino F.; Motti M. L.; Grieco P.; Carriero M. V. Cyclization of the Urokinase Receptor-Derived Ser-Arg-Ser-Arg-Tyr Peptide Generates a Potent Inhibitor of Trans-Endothelial Migration of Monocytes. PLoS One 2015, 10, e0126172 10.1371/journal.pone.0126172. 25938482 PMC4418665

[ref17] Oudhoff M. J.; Kroeze K. L.; Nazmi K.; van den Keijbus P. A. M.; van’t Hof W.; Fernandez-Borja M.; Hordijk P. L.; Gibbs S.; Bolscher J. G. M.; Veerman E. C. I. Structure-activity analysis of histatin, a potent wound healing peptide from human saliva: cyclization of histatin potentiates molar activity 1000-fold. FASEB J. 2009, 23, 3928–3935. 10.1096/fj.09-137588. 19652025

[ref18] Góngora-Benítez M.; Tulla-Puche J.; Albericio F. Multifaceted Roles of Disulfide Bonds. Peptides as Therapeutics. Chem. Rev. 2014, 114, 901–926. 10.1021/cr400031z. 24446748

[ref19] Cumming R. C.; Andon N. L.; Haynes P. A.; Park M.; Fischer W. H.; Schubert D. Protein Disulfide Bond Formation in the Cytoplasm during Oxidative Stress. J. Biol. Chem. 2004, 279, 21749–21758. 10.1074/jbc.M312267200. 15031298

[ref20] Chandrasekhar S.; Epling D. E.; Sophocleous A. M.; Topp E. M. Thiol-Disulfide Exchange in Peptides Derived from Human Growth Hormone. J. Pharm. Sci. 2014, 103, 1032–1042. 10.1002/jps.23906. 24549831 PMC4283463

[ref21] Rabenstein D. L.; Yeo P. L. Thiol/Disulfide Exchange Reactions of Captopril and Penicillamine with Arginine Vasopressin and Oxytocin. Bioorg. Chem. 1995, 23, 109–118. 10.1006/bioo.1995.1009.

[ref22] Stymiest J. L.; Mitchell B. F.; Wong S.; Vederas J. C. Synthesis of Oxytocin Analogues with Replacement of Sulfur by Carbon Gives Potent Antagonists with Increased Stability. J. Org. Chem. 2005, 70, 7799–7809. 10.1021/jo050539l. 16277299

[ref23] Gleeson E. C.; Jackson W. R.; Robinson A. J. Ring-closing metathesis in peptides. Tetrahedron Lett. 2016, 57, 4325–4333. 10.1016/j.tetlet.2016.08.032.

[ref24] Elder A. M.; Rich D. H. Two Syntheses of the 16- and 17-Membered DEF Ring Systems of Chloropeptin and Complestatin. Org. Lett. 1999, 1, 1443–1446. 10.1021/ol990990x. 10825992

[ref25] Afonso A.; Feliu L.; Planas M. Solid-phase synthesis of biaryl cyclic peptides by borylation and microwave-assisted intramolecular Suzuki-Miyaura reaction. Tetrahedron 2011, 67, 2238–2245. 10.1016/j.tet.2011.01.084.

[ref26] Byk G.; Cohen-Ohana M.; Raichman D. Fast and versatile microwave-assisted intramolecular Heck reaction in peptide macrocyclization using microwave energy. Peptide Sci. 2006, 84, 274–282. 10.1002/bip.20411. 16283655

[ref27] Cui H.-K.; Guo Y.; He Y.; Wang F.-L.; Chang H.-N.; Wang Y.-J.; Wu F.-M.; Tian C.-L.; Liu L. Diaminodiacid-Based Solid-Phase Synthesis of Peptide Disulfide Bond Mimics. Angew. Chem., Int. Ed. 2013, 52, 9558–9562. 10.1002/anie.201302197. 23804284

[ref28] Gleeson E. C.; Wang Z. J.; Robinson S. D.; Chhabra S.; MacRaild C. A.; Jackson W. R.; Norton R. S.; Robinson A. J. Stereoselective synthesis and structural elucidation of dicarba peptides. Chem. Commun. 2016, 52, 4446–4449. 10.1039/C5CC10540D. 26892179

[ref29] Qi Y.-K.; Qu Q.; Bierer D.; Liu L. A Diaminodiacid (DADA) Strategy for the Development of Disulfide Surrogate Peptides. Chem.—Asian J. 2020, 15, 2793–2802. 10.1002/asia.202000609. 32780939

[ref30] Pascoe C. A.; Engelhardt D. B.; Rosana A. R. R.; van Belkum M. J.; Vederas J. C. Methylene Analogues of Neopetrosiamide as Potential Antimetastatic Agents: Solid-Supported Synthesis Using Diamo Diacids for Pre-Stapling of Peptides with. Multiple Disulfides. Org. Lett. 2021, 23, 9216–9220. 10.1021/acs.orglett.1c03532. 34784223

[ref31] Schwieter K. E.; Johnston J. N. On-Demand Complex Peptide Synthesis: An Aspirational (and Elusive?) Goal for Peptide Synthesis. J. Am. Chem. Soc. 2016, 138, 14160–14169. 10.1021/jacs.6b08663. 27740747

[ref32] Jwad R.; Weissberger D.; Hunter L. Strategies for Fine-Tuning the Conformations of Cyclic Peptides. Chem. Rev. 2020, 120, 9743–9789. 10.1021/acs.chemrev.0c00013. 32786420

[ref33] Wong F.; Pappas S. C.; Curry M. P.; Reddy K. R.; Rubin R. A.; Porayko M. K.; Gonzalez S. A.; Mumtaz K.; Lim N.; Simonetto D. A.; Sharma P.; Sanyal A. J.; Mayo M. J.; Frederick R. T.; Escalante S.; Jamil K. Terlipressin plus Albumin for the Treatment of Type 1 Hepatorenal Syndrome. N. Eng. J. Med. 2021, 384, 818–828. 10.1056/NEJMoa2008290. 33657294

[ref34] Morrison P. M.; Foley P. J.; Warriner S. L.; Webb M. E. Chemical generation and modification of peptides containing multiple dehydroalanines. Chem. Commun. 2015, 51, 13470–13473. 10.1039/C5CC05469A. PMC4847484 26219458

[ref35] Peng X.; Xu K.; Zhang Q.; Liu L.; Tan J. Dehydroalanine modification sees the light: a photochemical conjugate addition strategy. Trends Chem. 2022, 4, 643–657. 10.1016/j.trechm.2022.04.008.

[ref36] Bogart J. W.; Bowers A. A. Dehydroamino acids: chemical multi-tools for late-stage diversification. Org. Biomol. Chem. 2019, 17, 3653–3669. 10.1039/C8OB03155J. 30849157 PMC6637761

[ref37] Larionov V. A.; Stoletova N. V.; Maleev V. I. Advances in Asymmetric Amino Acid Synthesis Enabled by Radical Chemistry. Adv. Synth. Catal. 2020, 362, 4325–4367. 10.1002/adsc.202000753.

[ref38] Zhang M.; He P.; Li Y. Contemporary Approaches to α,β-Dehydroamino Acid Chemical Modifications. Chem. Res. Chin. Univ. 2021, 37, 1044–1054. 10.1007/s40242-021-1307-z.

[ref39] Chilamari M.; Immel J. R.; Bloom S. General Access to C-Centered Radicals: Combining a Bioinspired Photocatalyst with Boronic Acids in Aqueous Media. ACS Catal. 2020, 10, 12727–12737. 10.1021/acscatal.0c03422. PMC12622572 41257217

[ref40] Chu L.; Ohta C.; Zuo Z.; MacMillan D. W. C. Carboxylic Acids as A Traceless Activation Group for Conjugate Additions: A Three-Step Synthesis of (±)-Pregabalin. J. Am. Chem. Soc. 2014, 136, 10886–10889. 10.1021/ja505964r. 25032785 PMC4132975

[ref41] Nawrat C. C.; Jamison C. R.; Slutskyy Y.; MacMillan D. W. C.; Overman L. E. Oxalates as Activating Groups for Alcohols in Visible Light Photoredox Catalysis: Formation of Quaternary Centers by Redox-Neutral Fragment Coupling. J. Am. Chem. Soc. 2015, 137, 11270–11273. 10.1021/jacs.5b07678. 26322524 PMC4632490

[ref42] Okada K.; Okamoto K.; Morita N.; Okubo K.; Oda M. Photosensitized decarboxylative Michael addition through N-(acyloxy)phthalimides via an electron-transfer mechanism. J. Am. Chem. Soc. 1991, 113, 9401–9402. 10.1021/ja00024a074.

[ref43] Wang M.; Wang C.; Huo Y.; Dang X.; Xue H.; Liu L.; Chai H.; Xie X.; Li Z.; Lu D.; Xu Z. Visible-light-mediated catalyst-free synthesis of unnatural α-amino acids and peptide macrocycles. Nat. Commun. 2021, 12, 6873 10.1038/s41467-021-27086-x. 34824205 PMC8617070

[ref44] Wright T. H.; Bower B. J.; Chalker J. M.; Bernardes G. J. L.; Wiewiora R.; Ng W.-L.; Raj R.; Faulkner S.; Vallée M. R. J.; Phanumartwiwath A.; Coleman O. D.; Thézénas M.-L.; Khan M.; Galan S. R. G.; Lercher L.; Schombs M. W.; Gerstberger S.; Palm-Espling M. E.; Baldwin A. J.; Kessler B. M.; Claridge T. D. W.; Mohammed S.; Davis B. G. Posttranslational mutagenesis: A chemical strategy for exploring protein side-chain diversity. Science 2016, 354, 597–623. 10.1126/science.aag1465. 27708059

[ref45] Yang A.; Ha S.; Ahn J.; Kim R.; Kim S.; Lee Y.; Kim J.; Söll D.; Lee H.-Y.; Park H.-S. A chemical biology route to site-specific authentic protein modifications. Science 2016, 354, 623–626. 10.1126/science.aah4428. 27708052 PMC5135561

[ref46] Zhang P.; Le C. C.; MacMillan D. W. C. Silyl Radical Activation of Alkyl Halides in Metallaphotoredox Catalysis: A Unique Pathway for Cross-Electrophile Coupling. J. Am. Chem. Soc. 2016, 138, 8084–8087. 10.1021/jacs.6b04818. 27263662 PMC5103281

[ref47] Shin J.-A.; Kim J.; Lee H.; Ha S.; Lee H.-Y. Cu(OTf)2-Promoted 1,4-Addition of Alkyl Bromides to Dehydroalanine. J. Org. Chem. 2019, 84, 4558–4565. 10.1021/acs.joc.9b00369. 30893548

[ref48] Luche J. L.; Allavena C. Ultrasound in organic synthesis 16. Optimisation of the conjugate additions to α,β-unsaturated carbonyl compounds in aqueous media. Tetrahedron Lett. 1988, 29, 5369–5372. 10.1016/S0040-4039(00)82870-1.

[ref49] Petrier C.; Dupuy C.; Luche J. L. Conjugate additions to α,β-unsaturated carbonyl compounds in aqueous media. Tetrahedron Lett. 1986, 27, 3149–3152. 10.1016/S0040-4039(00)84739-5.

[ref50] Huang T.; Keh C. C. K.; Li C.-J. Synthesis of α-amino acid derivatives and amines via activation of simple alkyl halides by zinc in water. Chem. Commun. 2002, 2440–2441. 10.1039/B204585K. 12430480

[ref51] Lipshutz B. H.; Huang S.; Leong W. W. Y.; Zhong G.; Isley N. A. C-C bond formation via copper-catalyzed conjugate addition reactions to enones in water at room temperature. J. Am. Chem. Soc. 2012, 134, 19985–19988. 10.1021/ja309409e. 23190029 PMC3959802

[ref52] Blanchard P.; El Kortbi M. S.; Fourrey J.-L.; Robert-Gero M. A conjugate addition of primary alkyl iodide derived species to electron deficient olefins. Tetrahedron Lett. 1992, 33, 3319–3322. 10.1016/S0040-4039(00)92077-X.

[ref53] Fleming F. F.; Gudipati S.; Aitken J. A. Alkenenitriles: Conjugate Additions of Alkyl Iodides with a Silica-Supported Zinc-Copper Matrix in Water. J. Org. Chem. 2007, 72, 6961–6969. 10.1021/jo0711539. 17685575

[ref54] Ye D.; Zhang Y.; Gao X. Electrocatalytic reduction of 1,2-diiodoethane by anions of supramolecular complex of (β-CD)2/C60 in DMF solution. Electrochim. Acta 2007, 52, 6686–6691. 10.1016/j.electacta.2007.04.091.

[ref55] Zhang Z.; Wang P. Highly stable copper oxide composite as an effective photocathode for water splitting via a facile electrochemical synthesis strategy. J. Mater. Chem. 2012, 22, 2456–2464. 10.1039/C1JM14478B.

[ref56] Kornfilt D. J. P.; MacMillan D. W. C. Copper-Catalyzed Trifluoromethylation of Alkyl Bromides. J. Am. Chem. Soc. 2019, 141, 6853–6858. 10.1021/jacs.9b03024. 30983333 PMC7557108

[ref57] Jenks C. J.; Bent B. E.; Bernstein N.; Zaera F. The Chemistry of Alkyl Iodides on Copper Surfaces. 1. Adsorption Geometry. J. Phys. Chem. B 2000, 104, 3008–3016. 10.1021/jp993021s.

[ref58] Cottrell T. L. The Strengths of Chemical Bonds, 2nd ed.; Butterworth: London, 1958.

[ref59] Darwent B. D. National Standard Reference Data Series; National Bureau of Standards: Washington, D.C., 1970; Vol. 31.

[ref60] Kerr J. A. Bond Dissociation Energies by Kinetic Methods. Chem. Rev. 1966, 66, 465–500. 10.1021/cr60243a001.

[ref61] Golubeva E. N.; Zhidomirov G. M.; Kokorin A. I. On the stability of copper(II) organic compounds with the σ bond Cu-C: A quantum-chemical study. Dokl. Chem. 2009, 426, 143–145. 10.1134/S0012500809060081.

[ref62] Chatgilialoglu C.; Ferreri C.; Landais Y.; Timokhin V. I. Thirty Years of (TMS)3SiH: A Milestone in Radical-Based Synthetic Chemistry. Chem. Rev. 2018, 118, 6516–6572. 10.1021/acs.chemrev.8b00109. 29938502

[ref63] Li J.-S.; Wu J. Recent Developments in the Photo-Mediated Generation of Silyl Radicals and Their Application in Organic Synthesis. ChemPhotoChem. 2018, 2, 839–846. 10.1002/cptc.201800110.

[ref64] Walsh R. Bond dissociation energy values in silicon-containing compounds and some of their implications. Acc. Chem. Res. 1981, 14, 246–252. 10.1021/ar00068a004.

[ref65] Sakai H. A.; Liu W.; Le C. C.; MacMillan D. W. C. Cross-Electrophile Coupling of Unactivated Alkyl Chlorides. J. Am. Chem. Soc. 2020, 142, 11691–11697. 10.1021/jacs.0c04812. 32564602 PMC7750884

[ref66] ElMarrouni A.; Ritts C. B.; Balsells J. Silyl-mediated photoredox-catalyzed Giese reaction: addition of non-activated alkyl bromides. Chem. Sci. 2018, 9, 6639–6646. 10.1039/C8SC02253D. 30310596 PMC6115631

[ref67] Dong J.; Lyu X.; Wang Z.; Wang X.; Song H.; Liu Y.; Wang Q. Visible-light-mediated Minisci C-H alkylation of heteroarenes with unactivated alkyl halides using O2 as an oxidant. Chem. Sci. 2019, 10, 976–982. 10.1039/C8SC04892D. 30774891 PMC6349069

[ref68] Heyder N. A.; Kleinau G.; Speck D.; Schmidt A.; Paisdzior S.; Szczepek M.; Bauer B.; Koch A.; Gallandi M.; Kwiatkowski D.; Bürger J.; Mielke T.; Beck-Sickinger A. G.; Hildebrand P. W.; Spahn C. M. T.; Hilger D.; Schacherl M.; Biebermann H.; Hilal T.; Kühnen P.; Kobilka B. K.; Scheerer P. Structures of active melanocortin-4 receptor-Gs-protein complexes with NDP-α-MSH and setmelanotide. Cell Res. 2021, 31, 1176–1189. 10.1038/s41422-021-00569-8. 34561620 PMC8563958

[ref69] Wingfield P. Protein precipitation using ammonium sulfate. Curr. Protoc. Protein Sci. 1998, 13, A.3F.1–A.3F.8. 10.1002/0471140864.psa03fs13. PMC4817497 18429073

[ref70] Immel J. R.; Chilamari M.; Bloom S. Combining flavin photocatalysis with parallel synthesis: a general platform to optimize peptides with non-proteinogenic amino acids. Chem. Sci. 2021, 12, 10083–10091. 10.1039/D1SC02562G. 34377401 PMC8317666

[ref71] Siriwardena T. N.; Gan B.-H.; Köhler T.; van Delden C.; Javor S.; Reymond J.-L. Stereorandomization as a Method to Probe Peptide Bioactivity. ACS Cent. Sci. 2021, 7, 126–134. 10.1021/acscentsci.0c01135. 33532575 PMC7845017

[ref72] Udagawa H.; Yoneda T. H.; Masuda R.; Koide T. A Strategy for Discovering Heterochiral Bioactive Peptides by Using the OB2^*n*^P Library and SPOTs Method. ChemBioChem. 2019, 20, 2070–2073. 10.1002/cbic.201900237. 31111638

[ref73] Berman J.; Green M.; Sugg E.; Anderegg R.; Millington D. S.; Norwood D. L.; McGeehan J.; Wiseman J. Rapid Optimization of Enzyme Substrates Using Defined Substrate Mixtures. J. Biol. Chem. 1992, 267, 1434–1437. 10.1016/S0021-9258(18)45963-7. 1309783

[ref74] Zheng Y.; Mao K.; Chen S.; Zhu H. Chirality Effects in Peptide Assembly Structures. Bioeng. Biotechnol. 2021, 9, 703004 10.3389/fbioe.2021.703004. PMC8258317 34239866

[ref75] Hosono Y.; Morimoto J.; Sando S. A comprehensive study on the effect of backbone stereochemistry of a cyclic hexapeptide on membrane permeability and microsomal stability. Org. Biomol. Chem. 2021, 19, 10326–10331. 10.1039/D1OB02090K. 34821247

[ref76] Sabatino G.; D’Ercole A.; Pacini L.; Zini M.; Ribecai A.; Paio A.; Rovero P.; Papini A. M. An Optimized Scalable Fully Automated Solid-Phase Microwave-Assisted cGMP-Ready Process for the Preparation of Eptifibatide. Org. Process Res. Dev. 2021, 25, 552–563. 10.1021/acs.oprd.0c00490.

[ref77] Drucker D. J. Advances in oral peptide therapeutics. Nat. Rev. Drug Discovery 2020, 19, 277–289. 10.1038/s41573-019-0053-0. 31848464

[ref78] Rai U.; Thrimawithana T. R.; Valery C.; Young S. A. Therapeutic uses of somatostatin and its analogues: Current view and potential applications. Pharmacol. Ther. 2015, 152, 98–110. 10.1016/j.pharmthera.2015.05.007. 25956467

[ref79] Clément K.; Biebermann H.; Farooqi I. S.; Van der Ploeg L.; Wolters B.; Poitou C.; Puder L.; Fiedorek F.; Gottesdiener K.; Kleinau G.; Heyder N.; Scheerer P.; Blume-Peytavi U.; Jahnke I.; Sharma S.; Mokrosinski J.; Wiegand S.; Müller A.; Weiß K.; Mai K.; Spranger J.; Grüters A.; Blankenstein O.; Krude H.; Kühnen P. MC4R agonism promotes durable weight loss in patients with leptin receptor deficiency. Nat. Med. 2018, 24, 551–555. 10.1038/s41591-018-0015-9. 29736023

[ref80] Kühnen P.; Clément K.; Wiegand S.; Blankenstein O.; Gottesdiener K.; Martini L. L.; Mai K.; Blume-Peytavi U.; Grüters A.; Krude H. Proopiomelanocortin Deficiency Treated with a Melanocortin-4 Receptor Agonist. N. Engl. J. Med. 2016, 375, 240–246. 10.1056/NEJMoa1512693. 27468060

[ref81] Abraham M. H.; Martins F.; Mitchell R. C.; Salter C. J. Hydrogen bonding. 47. Characterization of the ethylene glycol-heptane partition system: Hydrogen bond acidity and basicity of peptides. J. Pharm. Sci. 1999, 88, 241–247. 10.1021/js980242l. 9950645

[ref82] Chikhale E. G.; Ng K.-Y.; Burton P. S.; Borchardt R. T. Hydrogen Bonding Potential as a Determinant of the in Vitro and in Situ Blood-Brain Barrier Permeability of Peptides. Pharm. Res. 1994, 11, 412–419. 10.1023/A:1018969222130. 8008709

[ref83] Conradi R. A.; Hilgers A. R.; Ho N. F. H.; Burton P. S. The Influence of Peptide Structure on Transport Across Caco-2 Cells. Pharm. Res. 1991, 8, 1453–1460. 10.1023/A:1015825912542. 1808606

[ref84] Paterson D. A.; Conradi R. A.; Hilgers A. R.; Vidmar T. J.; Burton P. S. A Non-aqueous Partitioning System for Predicting the Oral Absorption Potential of Peptides. Quant. Struct.-Act. Rel. 1994, 13, 4–10. 10.1002/qsar.19940130103.

[ref85] Bogdanowich-Knipp S. J.; Chakrabarti S.; Siahaan T. J.; Williams T. D.; Dillman R. K. Solution stability of linear vs. cyclic RGD peptides. Pept. Res. 1999, 53, 530–541. 10.1034/j.1399-3011.1999.00052.x. 10424348

[ref86] Galande A. K.; Trent J. O.; Spatola A. F. Understanding base-assisted desulfurization using a variety of disulfide-bridged peptides. Peptide Sci. 2003, 71, 534–551. 10.1002/bip.10532. 14635094

[ref87] Gawron O.; Odstrchel G. Kinetic studies on the alkaline decomposition of cystine derivatives and peptides. J. Am. Chem. Soc. 1967, 89, 3263–3267. 10.1021/ja00989a029. 6042762

[ref88] Robbins F. M.; Fioriti J. A. Alkaline Degradation of Cystine, Glutathione and Sulphur-containing Proteins. Nature 1963, 200, 577–578. 10.1038/200577a0. 14082230

[ref89] Wang Z.; Rejtar T.; Zhou Z. S.; Karger B. L. Desulfurization of cysteine-containing peptides resulting from sample preparation for protein characterization by mass spectrometry. Rapid Commun. Mass Spectrom. 2010, 24, 267–275. 10.1002/rcm.4383. 20049891 PMC2908508

