# Supplementary material for: Peptide Carbocycles: From −SS– to −CC– via a Late-Stage “Snip-and-Stitch”
Source: ACS Cent Sci. 2022 Oct 28;8(11):1537–47. doi: 10.1021/acscentsci.2c00456 (PMC9686213; doi:10.1021/acscentsci.2c00456)
Supplement: Supplementary file 1 — oc2c00456_si_001.pdf [file oc2c00456_si_001.pdf]

Peptide *Carbo*cycles: From -SS- to -CC- via a Late-stage 'Snip-and-Stitch'

Samuel Gary and Steven Bloom*

Department of Medicinal Chemistry, University of Kansas, Lawrence, Kansas 66045

*Corresponding author email: spbloom@ku.edu

This PDF contains the following:

Pages S1-S168

Tables S1-S36

Table of Contents

General Information 2

Small Molecule Synthesis 3

Dehydroalanine Synthesis 20

Screening for Dha-Forming Reagents 20

General Procedure for Dha Synthesis 20

Peptide-Dha_2 Characterization 21

Identifying Platforms for Cyclization 31

Photochemical Approaches 31

Electrochemical Approaches 36

Organometallic Approaches 48

Representative LC Traces 49

Peptide Cyclization 50

Reaction Optimization 50

Representative LC Traces for Optimization 55

General Procedure for Peptide Cyclization 57

Carbocyclic Setmelanotide Analogue Characterization 58

Characterization of Other Carbocyclic Peptides 92

Generation of Setmelanotide Standard Curve 100

Pharmacokinetic Studies 101

Procedure for Solubility Determination 101

Procedure for Permeability Determination 101

Procedure for Hydrolytic Stability Determination 101

LC Spectra 101
Mechanistic Studies 106
Hydrosilane Diastereoselectivity 112
DFT Calculations 118
NMR Spectra 135
Peptide Diastereomer Characterization 155
References 168

General Information

Unless otherwise stated, reagents and solvents were purchased and used as received (Sigma-Aldrich, Alfa Aesar, Fisher Scientific, Combi-Blocks, and Oakwood Chemicals). ddH_2O refers to using Millipore Grade I water (Resistivity > 5 MΩ cm, Conductivity < 0.2 µS/cm, TOC <30 ppb) and was used for all peptide reactions. Aqueous buffers and solutions were prepared using ddH_2O and the pH adjusted using a Mettler Toledo FiveEasy pH meter. ^{1}H NMR, ^{13}C NMR, ^{19}F NMR, ^{11}B NMR, and ^{2}H NMR spectra were recorded on a Bruker Ascend 400 NMR spectrometer. ^{1}H NMR and ^{13}C NMR spectra were referenced to residual solvent peaks ($CDCl_3$ 7.26 and 77.06 ppm; D_2O 4.79 ppm; DMSO-d_6 2.50 and 39.53 ppm). Peak multiplicities are designated by the following abbreviations: b, broad; s, singlet; d, doublet; t, triplet; q, quartet; quint, quintet; sept, septet; m, multiplet; dd, doublet of doublets; dt, doublet of triplets; tt, triplet of triplets; and ddd, doublet of doublet of doublets. Peptide NMR spectra were recorded on Avance AVIII 500 MHz spectrometer equipped with a multi-nuclear BBFO cryoprobe or Bruker AVIII 600 MHz NMR spectrometer equipped with a triple resonance inverse probe. Electrospray ionization spectra were acquired on a LCT Premier (Waters Corp.) time of flight mass spectrometer (HRMS). LC-MS/MS data was acquired on a Quatro Ultima (Waters Corp.) triple quadrupole mass analyzer with an electrospray ion source. LC-MS spectra were acquired on a Waters Acquity UPLC H-Class equipped with a Waters QDa Mass Detector using a Discovery BIO Wide Pore C8 HPLC column (Supelco; 5 µm, 10 cm x 4.6 mm). LC spectra were obtained at 214 nm or 280 nm using the following gradient (A, H_2O/0.1% formic acid; B, MeCN/0.1% formic acid): initial 95% A, 2.0 min. 95% A, 3.0 min. 90% A, 9.0 min. 70% A, 17.0 min. 50% A, 17.5 min. 5% A, 19.0 min. 5% A, 20 min. 95% A. Small molecules were purified via flash chromatography using an Isolera 1 Biotage instrument with the indicated solvent systems with Buchi FlashPure EcoFlex prepacked columns. Peptides were purified via flash chromatography using a Buchi Pure C-815 instrument

with Biotage Sfär C18 Duo prepacked columns with the indicated solvent system. Thin-layer chromatography (TLC) was performed on silica gel coated glass TLC plates (Merck, TLC Silica gel 60 F254) and visualized using a UV lamp (254 nm) in combination with various stains. Electrochemistry was performed using an ElectraSyn 2.0 (IKA). Photochemical reactions were carried out using Lumidox® Generation II Controller and the 96-well LED array (445 nm, 295 mW per well) with a Lens Mat Active Cooling Base from Analytical Sales and Services Inc. or LED lights from Kessil (PR160L, 525 nm, 40 W).

Small Molecule Synthesis

Bis(1,3-dioxoisoindolin-2-yl) adipate **(B)**

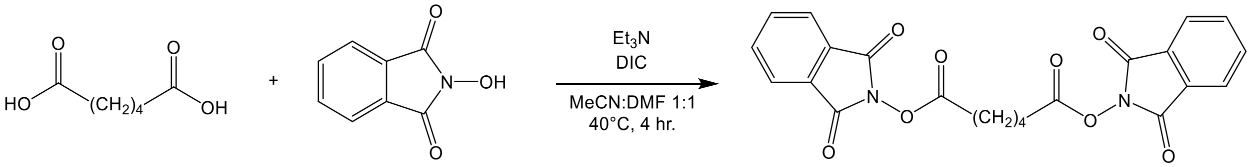

Adipic acid (2.00 mmol) and *N*-hydroxyphthalimide (4.10 mmol, 668.8 mg) were added to a scintillation vial containing a magnetic stir bar. 2.0 mL of *N,N*-dimethylformamide followed by 2.0 mL of acetonitrile were added to the vial (0.50 M). The solution was heated to 40 °C, at which point triethylamine (4.10 mmol, 571 µL) followed by N,N'-diisopropylcarbodiimide (4.10 mmol, 642 µL) were added. The reaction was stirred at 40 °C for 4 hours. The solvent was then removed under reduced pressure. The desired product was recrystallized from 65 mL methanol, collected via vacuum filtration, and dried under high vacuum with no further purification. The product was obtained as a white powder in 503 mg (58% yield).

^{1}H NMR (DMSO-*d*$_6$, 400 MHz): δ 7.94-8.00 (8H, m), 2.86 (4H, m), 1.79 (4H, m)

^{13}C NMR (DMSO-*d*$_6$, 101 MHz): δ 169.87, 161.88, 135.56, 128.21, 124.03, 29.64, 23.33

HRMS (ESI+): Calculated for $C_{22}H_{16}N_2NaO_8$ [M+Na]$^+$: 459.0804 Found: 459.0808

1,1'-(butane-1,4-diyl)bis(2,4,6-triphenylpyridin-1-ium) tetrafluoroborate **(A)**

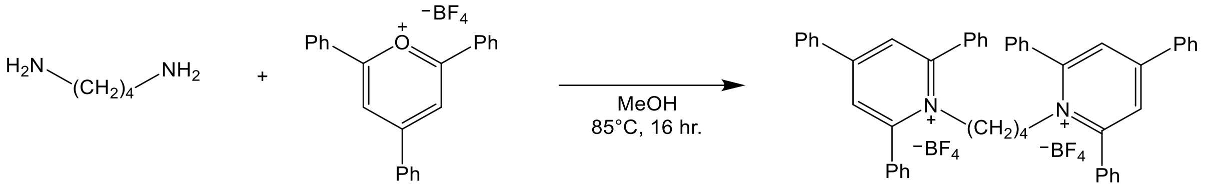

1,4-diaminobutane (251 µL, 2.5 mmol) was added to a 100-mL round bottom flask containing a magnetic stir bar and 55 mL methanol. 2,4,6-thriphenylpyrilium tetrafluoroborate (1.96 g, 4.95 mmol) was added, at which point a deep red color developed. The resulting solution was heated to 85 °C under a reflux condenser for ~16 hours, followed by removal of the solvent under reduced pressure. The solid was diluted with hot water (80-90 °C, 50 mL), stirred, and allowed to cool to room temperature naturally. Cooling via an ice bath for an additional hour followed by vacuum filtration afforded the desired compound in 577 mg (27%) as a beige-tan powder.

^{1}H NMR (DMSO-*d*$_6$, 400 MHz): δ 8.41 (4H, s), 8.24-8.24 (4H, m), 7.72-7.76 (4H, m), 7.59-7.67 (22H, m), 3.81-3.84 (4H, m), 0.73-0.77 (4H, m)

^{13}C NMR (DMSO-*d*$_6$, 101 MHz): δ 156.22, 154.69, 133.46, 133.08, 132.98, 131.54, 130.10, 129.54, 129.38, 129.13, 126.39, 53.39, 25.41

^{19}F NMR (DMSO-*d*$_6$, 376 MHz): δ -148.24, -148.30

HRMS (ESI+): Calculated for $C_{50}H_{42}N_2Na/2$ [M-2BF$_4$/2]$^+$: 335.1669 Found: 335.1676

O,O'-(butane-1,4-diyl) diethyl dioxalate

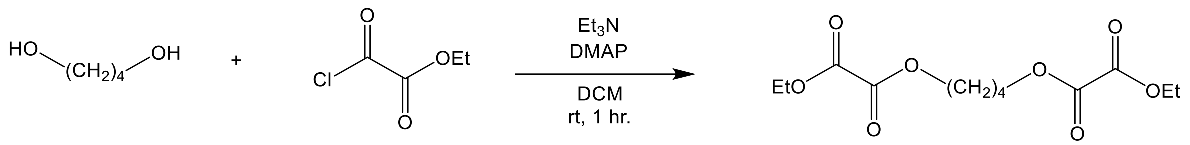

1,4-butanediol (558 µL, 6.3 mmol), triethylamine (2.20 mL, 15.8 mmol), and 4-(dimethylamino)pyridine (77.0 mg, 0.63 mmol) were dissolved in 58 mL DCM. The solution was stirred vigorously, then ethyl chloroglyoxylate (1.76 mL, 15.8 mmol) in DCM (1 mL) was added dropwise over ~10 minutes. The reaction was stirred at room temperature for an additional hour, quenched slowly with excess saturated aqueous ammonium chloride, and extracted into DCM (3 x 60 mL). The combined organic layers were dried over $MgSO_4$, concentrated, and purified by flash chromatography (silica; hexanes to EtOAc). The target compound was isolated in 1.47 g (80%) as a viscous, pale yellow oil.

^{1}H NMR ($CDCl_3$, 400 MHz): δ 4.19-4.24 (8H, m), 1.69-1.80 (4H, m), 1.24 (t, *J*=7.1 Hz)

^{13}C NMR ($CDCl_3$, 101 MHz): δ 157.64, 157.48, 66.00, 62.94, 24.64, 13.69.

HRMS (ESI+): Calculated for $C_{12}H_{18}O_8Na$ $[M+Na]^+$: 313.0899 Found: 313.0894

O,O'-(butane-1,4-diyl) dicesium dioxalate **(F)**

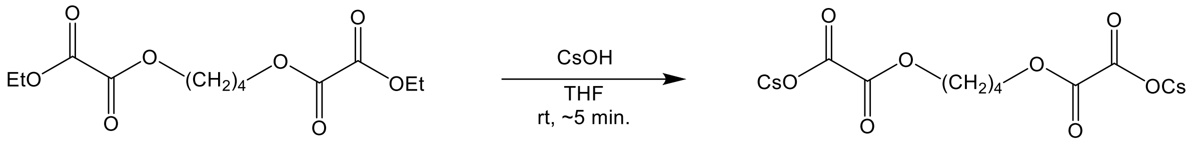

O,O'-(butane-1,4-diyl) diethyl dioxalate (580 mg, 2.0 mmol) was added to a 1-dram vial containing a magnetic stir bar and 2.00 mL of THF. Aqueous CsOH (4.0 mmol) was added, followed by vigorous stirring for ~5 minutes. The solvent was removed under reduced pressure followed by drying under high vacuum to yield the target compound as a white solid (967 mg, 97%).

^{1}H NMR (D_2O, 400 MHz): δ 3.26-3.28 (4H, m), 1.23-1.25 (4H, m)

1,4-bis(4,4,5,5-tetramethyl-1,3,2-dioxaborolan-2-yl)butane

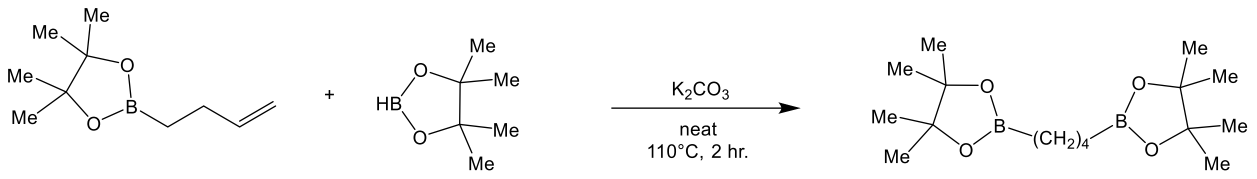

To a 5-mL vial containing a magnetic stir bar was added 2-(but-3-en-1-yl)-4,4,5,5-tetramethyl-1,3,2-dioxaborolane (250 mg, 1.37 mmol), pinacolborane (460 µL, 2.74 mmol), and K_2CO_3 (18.9 mg, 0.137 mmol). The suspension was then heated to 110 °C under a reflux condenser for 2 hours. After cooling to room temperature, the reaction was quenched with water (1.5 mL). The product was extracted into DCM (3 x 10 mL), combined organic layers dried over $MgSO_4$, and the solvent removed under reduced pressure. Flash chromatography (silica gel; hexanes to DCM) afforded the target compound as a pale yellow oil (155 mg, 37%).

^{1}H NMR ($CDCl_3$, 400 MHz): δ 1.35-1.37 (4H, br. m), 1.19-1.21 (24H, app. d, *J* = 8.8 Hz), 0.70-0.78 (4H, br. m)

^{13}C NMR ($CDCl_3$, 101 MHz): δ 82.94, 27.03, 24.95

^{11}B NMR ($CDCl_3$, 128 MHz): δ 33.82

HRMS (ESI+): Calculated for $C_{16}H_{32}B_2O_4Na$ $[M+Na]^+$: 333.2384 Found: 333.2383

Butane-1,4-diyldiboronic acid **(G)**

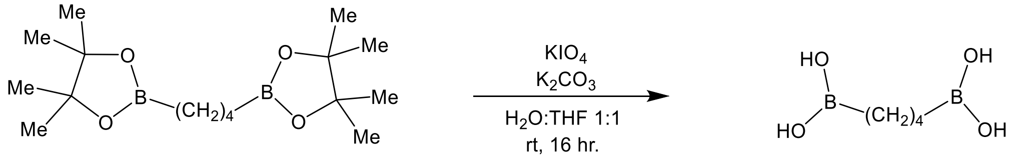

To a scintillation vial charged with a magnetic stir bar, 1,4-bis(4,4,5,5-tetramethyl-1,3,2-dioxaborolan-2-yl)butane (70.1 mg, 0.23 mmol), H_2O (2.3 mL) and THF (2.3 mL) were added. Next, K_2CO_3 (62 mg, 0.45 mmol) was added, and the solution was stirred at room temperature for 14 hours. KIO_4 (104 mg, 0.45 mmol) was added and stirring at room temperature continued for an additional 2 hours. The solvent was removed under reduced pressure, the solid washed with EtOAc (3 x 5 mL), filtered, and organic layers again concentrated. The target compound was obtained in 18 mg (55 %) as a colorless solid.

^{1}H NMR (D_2O, 400 MHz): δ 1.33 (4H, m), 0.60 (4H, m)

^{11}B NMR (D_2O, 128 MHz): δ 3.58

Methyl 2,5-dibromovalerate

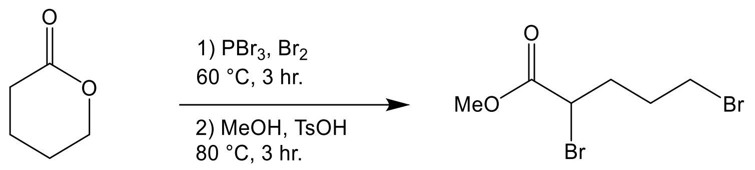

To a 50 mL flask charged with a magnetic stir bar was added δ-valerolactone (2.40 mL, 25.9 mmol), bromine (2.00 mL, 39.0 mmol), and PBr_3 (77 µL, 0.518 mmol). The solution was heated under reflux for 3 hours at 60 °C, at which point it was allowed to cool to room temperature. MeOH (5.23 mL, 130 mmol) and p-toluene sulfonic acid monohydrate (47 mg, 0.259 mmol) were then added, and the solution was heated to 80 °C under reflux for an additional 3 hours. The solvent was removed under reduced pressure and the products extracted between DCM (3 x 30 mL) and 10% aq. NaOH. The combined organic layers were dried over $MgSO_4$, concentrated, and purified by flash chromatography (silica gel; hexanes to EtOAc). The product was obtained in 4.32 g (61%) as a pale yellow, viscous oil.

^{1}H NMR ($CDCl_3$, 400 MHz): δ 4.28 (1H, dd, *J*=8.2, 6.1 Hz), 3.80 (3H, s), 3.44 (2H, t, *J*=6.4 Hz), 1.90-2.31 (4H, br. m)

Spectral data is consistent with published data.[1]

Dimethylphenyl deuterosilane

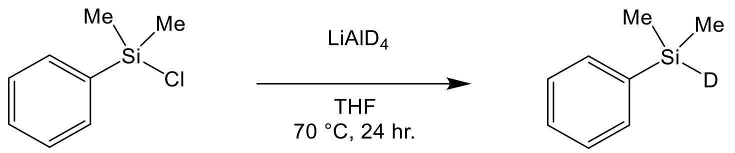

A 50 mL oven-dried flask was charged with a magnetic stir bar and 30 mL of THF (dried and stored over 4 Å molecular sieves). Lithium aluminum deuteride (419.8 mg, 10.0 mmol) was added in small portions, followed by dropwise addition of dimethylphenyl chlorosiliane (839 µL, 5.0 mmol). THe resulting suspension was stirred rapidly for 30 minutes at room temperature, then heated to 70 °C under a reflux condenser for 24 hours. Upon cooling to room temperature, acetone (10 mL) followed by saturated aqueous NH_4Cl (5 mL) were added slowly. The suspension was filtered over a small pad of Celite and concentration under reduced pressure. The products were portioned between water (30 mL) and DCM (3 x 30 mL). The combined organic layers were dried over $MgSO_4$, concentrated under reduced pressure, and purified by flash chromatography (silica gel; hexanes to DCM). The title compound was obtained as a colorless oil in 323 mg (47%).

^{1}H NMR ($CDCl_3$, 400 MHz): δ 7.65-7.67 (2H, m), 7.46-7.48 (3H, m), 0.46 (6H, s)

^{2}H NMR ($CHCl_3$, 61 MHz): δ4.00 (1H, s)

^{13}C NMR ($CDCl_3$, 101 MHz): δ 137.55, 134.15, 129.33, 128.01, -3.72

HRMS(ESI+): calculated for $C_8H_{11}DSiNa$ $(M+Na)^+$: 160.0669. Found: 160.0676

1-isopropyl-2,4,6-triphenylpyridin-1-ium tetrafluoroborate

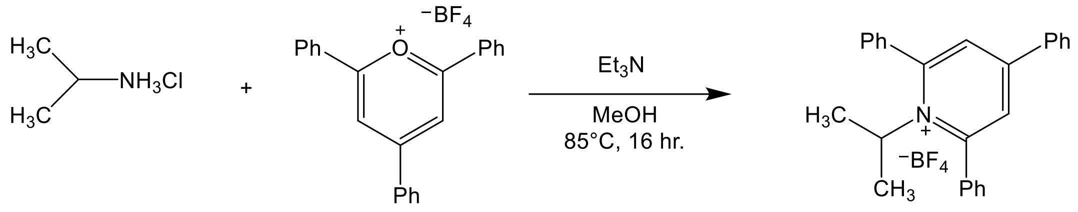

In a 100 mL flask charged with a magnetic stir bar, isopropylammonium chloride (191 mg, 2 mmol) and 2,4,6-triphenylpyrilium tetrafluoroborate (792 mg, 2 mmol) were dissolved in 40 mL MeOH. The resulting solution was heated to 85 °C under a reflux condenser, at which point triethylamine (279 µL, 2 mmol) was added. After heating for 16 hours, the solution was cooled to room temperature and the product precipitated with 50 mL precooled Et_2O (~ 0 °C). Vacuum filtration afforded the title compound as a slight yellow powder (407 mg, 47%).

^{1}H NMR (DMSO-*d*$_6$, 400 MHz): δ 7.58-7.89 (14 H, m), 7.31-7.50 (3H, m), 5.14 (1H, sept, *J*=6.9 Hz), 1.31 (6H, d, *J*=7.1 Hz)

Spectral data is consistent with published data.[2]

1,3-dioxoisoindolin-2-yl isobutyrate

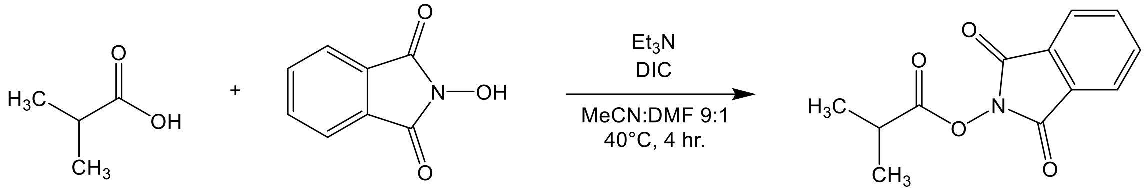

Isobutyric acid (908 µL, 10 mmol), *N,N'*-diisopropylcarbodiimide (1.88 mL, 12 mmol), and triethylamine (1.67 mL, 12 mmol) were dissolved in 20 mL of 9:1 MeCN:DMF in a 100 mL flask equipped with a magnetic stir bar. The solution was heated to 40 °C with stirring for 1 hour, after which *N*-hydroxyphthalimide (1.63 g, 10 mmol) was added. After stirring for an additional 3 hours, H_2O (~ 0 °C, 30 mL) was added. The precipitate was collected via vacuum filtration then purified via flash chromatography (silica gel; hexanes to EtOAc). The title compound was obtained as a white solid (1.24 g, 53%).

^{1}H NMR (DMSO-*d*$_6$, 400 MHz): δ 7.96-8.00 (4 H, m), 3.05 (1H, sept, *J*=7.0 Hz), 1.27 (6H, d, *J*=7.0 Hz)

Spectral data is consistent with published data.[3]

Ethyl isopropyl oxalate

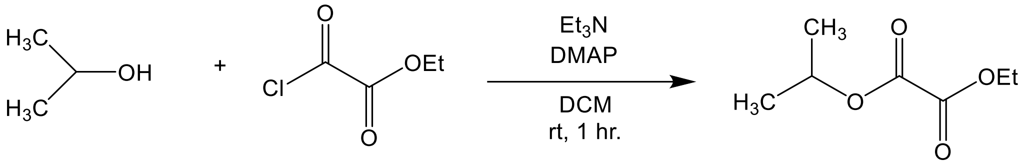

To a 250 mL flask containing a magnetic stir bar was added 2-propanol (765 µL, 10 mmol), triethylamine (1.67 mL, 12 mmol), 4-(dimethylamino)pyridine (122 mg, 1.0 mmol), and 100 mL DCM. With rapid stirring, ethyl oxalyl chloride (1.12 mL, 10 mmol) was slowly added. Upon completion of addition, the reaction was stirring for 1 hour at room temperature, upon which 100 mL of water was carefully added to quench the reaction. The product was partitioned into DCM, extracted an additional 2 times (2 x 100 mL DCM), and organic layers combined. After drying over $MgSO_4$ and concentrating under reduced pressure, the product was isolated by flash chromatography (silica gel; hexanes to DCM). The desired product was obtained as a pale oil in 477 mg (30 %).

^{1}H NMR ($CDCl_3$, 400 MHz): δ 5.17 (1H, sept, *J*=6.3 Hz), 4.35 (2H, q, *J*=7.2 Hz), 1.33-1.43 (9H, m)

^{13}C NMR ($CDCl_3$, 101 MHz): δ 158.27, 157.63, 71.55, 63.12, 21.60, 14.03

HRMS (ESI+): Calculated for $C_7H_{12}O_4Na$ $(M+Na)^+$: 183.0633. Found: 183.0628

Cesium isopropyl oxalate

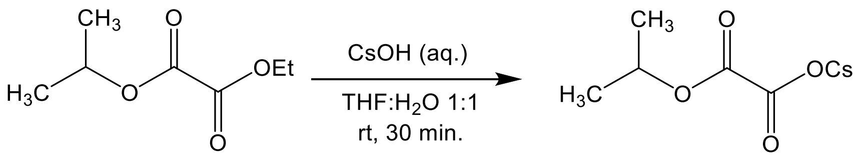

Ethyl isopropyl oxalate (105 mg, 0.66 mmol) was added to a scintillation vial equipped with a magnetic stir bar. THF (3.3 mL) and water (3.3 mL) were added next, followed by aqueous CsOH (0.66 mmol). The resulting solution was stirred vigorous at room temperature for ~30 minutes as monitored by TLC. Upon consumption of the starting material, the THF was removed under reduced pressure at 25 °C. The aqueous solution was then flash-frozen and lyophilized with no further purification to yield a white powder (88.1 mg, 51%).

^{1}H NMR (D_2O, 400 MHz): δ 4.89 (1H, br. m), 1.38 (6H, d, *J*=6.9 Hz)

General Procedure for the Reduction of Dicarboxylic Esters

Di-ester (1.0 equivalent) was dissolved in THF (0.2 M) in a round-bottom flask equipped with a magnetic stir bar. This solution was cooled in an ice bath and lithium aluminum hydride (2.2 equivalents) was added in small portions over 30 minutes. After stirring for 1 hour in an ice bath, the mixture was allowed to warm to room temperature and stirred for an additional 4 hours. The reaction was quenched by the slow addition of acetone (10 mL) followed by sat. aq. NH_4Cl (15 mL) The mixture was filtered, washed with acetone, then concentrated under reduced pressure. The resulting diol was extracted into ethyl acetate (3 x 20 mL), dried over $MgSO_4$, concentrated, and used without further purification.

General Procedure for the Reduction of Dicarboxylic Acids

Di-ester (1.0 equivalent) was dissolved in THF (0.2 M) in a round-bottom flask equipped with a magnetic stir bar. This solution was cooled in an ice bath and lithium aluminum hydride (4.4 equivalents) was added in small portions over 30 minutes. After stirring for 1 hour in an ice bath, the mixture was allowed to warm to room temperature, heated to 70 °C under a reflux condenser, and stirred for an additional 4 hours. The reaction was cooled to room temperature and quenched by the slow addition of acetone (10 mL) followed by sat. aq. NH_4Cl (15 mL) The mixture was filtered, washed with acetone, then concentrated under reduced pressure. The resulting diol was extracted into ethyl acetate (3 x 20 mL), dried over $MgSO_4$, concentrated, and used without further purification.

General Procedure for the Conversion of Diols to Di-iodides

Diol (1.0 equivalent) was dissolved in THF (0.2 M) in a round-bottom flask equipped with a magnetic stir bar. Imidazole (2.0 equivalents) and triphenylphosphine (2.2 equivalents) were added to the flask, followed by the portion-wise addition of iodine crystals (2.5 equivalents). The reaction was stirred at room temperature for 10 minutes, then heated to 60 °C for 5 hours. After cooling to room temperature, aqueous sodium thiosulfate was added until the brown coloration disappeared. The solvent was removed under reduced pressure, diluted with water (20 mL) and extracted into dichloromethane (3 x 20 mL). The combined organic fractions were dried over $MgSO_4$ and evaporated under reduced pressure. Flash chromatography (silica gel; hexanes to EtOAc) furnished pure di-iodides.

1,3-diiodopentane

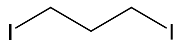

^{1}H NMR ($CDCl_3$, 400 MHz): δ 3.27 (4H, t, *J*=6.5 Hz), 2.24 (2H, quint, *J*=6.6 Hz)

858 mg of yellow oil from 6 mmol of malonic acid (55% over 2 steps)

Spectral data is consistent with published data.[4]

1,4-diiodobutane **(14)**

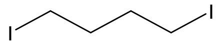

^{1}H NMR ($CDCl_3$, 400 MHz): δ 3.19 (4H, m), 1.94 (4H, m)

246 mg of pale oil from 4 mmol of succinic acid (20% over 2 steps)

Spectral data is consistent with published data.[5]

1,5-diiodopentane **(15)**

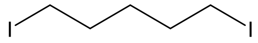

^{1}H NMR ($CDCl_3$, 400 MHz): δ 3.21(4H, t, J=6.9 Hz), 1.87 (4H, quint, *J*=7.5 Hz), 1.51-1.59 (2H, m)

350 mg of colorless oil from 4 mmol of dimethyl glutarate (27% over 2 steps)

Spectral data is consistent with published data.[6]

1,6-diiodohexane **(16)**

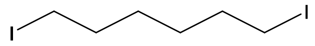

^{1}H NMR ($CDCl_3$, 400 MHz): δ 3.18 (4H, t, *J*= 6.9 Hz), 1.79-1.87 (4H, m) 1.38-1.45 (4H, m)

401 mg of pale oil from 4 mmol of dimethyl adipate (30% over 2 steps)

Spectral data is consistent with published data.[7]

1,7-diiodoheptane **(17)**

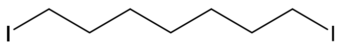

^{1}H NMR ($CDCl_3$, 400 MHz): δ 3.18 (4H, t, *J*= 7.0 Hz), 1.82 (4H, quint, *J*=7.0 Hz), 1.27-1.45 (6H, m)

243 mg of yellow oil from 4 mmol of pimelic acid (17% over 2 steps)

Spectral data is consistent with published data.[8]

1,8-diiodooctane **(18)**

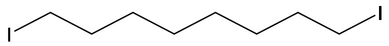

^{1}H NMR ($CDCl_3$, 400 MHz): δ 3.15 (4H, t, *J*= 7.0 Hz), 1.79 (4H, quint, *J*=7.0 Hz), 1.32-1.42 (4H, m), 1.23-1.29 (4H, m)

609 mg of a low-melting yellow solid from 3 mmol of 1,8-octanediol (55%)

Spectral data is consistent with published data.[9]

1-iodo-2-(2-(2-(2-iodoethoxy)ethoxy)ethoxy)ethane **(21)**

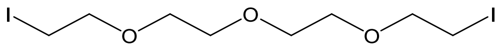

^{1}H NMR (CDCl$_3$, 400 MHz): δ 3.78 (4H, dd, *J*=7.3, 6.5 Hz), 3.67 (8H, s), 3.27 (4H, dd, *J*=7.3, 6.5 Hz)

^{13}C NMR (CDCl$_3$, 101 MHz): δ 71.90, 70.62, 70.18, 3.14

HRMS (ESI+): calculated for $C_8H_{16}O_3I_2Na$ $(M+Na)^+$: 436.9087. Found: 436.9091

214 mg of pale oil from 4 mmol of tetraethylene glycol (13%)

1,3-bis(iodomethyl)adamantane **(23)**

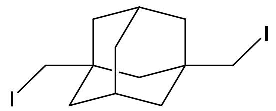

^{1}H NMR (CDCl$_3$, 400 MHz): δ 3.05 (4H, s), 2.03-2.05 (2H, m), 1.49-1.51 (2H, m), 1.43 (8H, s), 1.27 (2H, s)

^{13}C NMR (CDCl$_3$, 101 MHz): δ 46.37, 41.31, 35.64, 33.64, 29.19, 25.22

HRMS (ESI+): calculated for $C_{12}H_{18}I_2Na$ $(M+Na)^+$: 438.9390. Found: 438. 9387

433 mg of a white solid from adamantane 1,3-dicarboxylic acid (26% over 2 steps)

1,3-diiodocyclohexane **(24)**

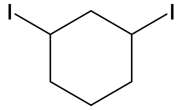

^{1}H NMR ($CDCl_3$, 400 MHz): 5.30-5.38 (2H, m), 5.02-5.11 (4H, tt, *J*=9.1, 3.9 Hz), 2.34-2.43 (2H, m), 2.09-2.16 (2H, t, *J*=5.6 Hz), 1.99-2.07 (6H, m), 1.89-1.96 (4H, m), 1.74-1.84 (4H, m), 1.60-1.72 (4H, m), 1.42-1.56 (2H, m)

HRMS (ESI+): calculated for $C_6H_{10}I_2Na$ $(M+Na)^+$: 358.8764. Found: 358.8758

182 mg of a yellow oil from 2 mmol of cyclohexyl-1,3-diol (27 % as a mixture of diastereomers)

1,4-bis(iodomethyl)benzene **(25)**

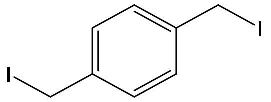

^{1}H NMR ($CDCl_3$, 400 MHz): δ 7.30 (4H, s), 4.42 (4H, s)

441 mg of a tan powder from 2 mmol of benzene-1,4-dimethanol (62 %)

Spectral data is consistent with published data.[10]

Dehydroalanine Synthesis

Screening for Dha-Forming Reagents

Terlipressin (0.5 mg, 408 nmol) and tris(2-carboxyethyl)phosphine HCl (TCEP) (0.2 mg, 698 nmol) were dissolved in 1.0 mL water in a 1-dram vial equipped with magnetic stir bar. After stirring at room temperature for 1 hour, reagent (4.08 µmol, **Figure S1**) dissolved in DMF (1.0 mL) was added to the vial. After 4 hours, K_2CO_3 (0.6 mg, 4.08 µmol) was added. Stirring at room temperature was continued for an additional 4 hours, at which point the reaction was filtered and analyzed by LC-MS.

Figure S1. Survey of Reagents Screened for Dha_2-Terlipressin Synthesis

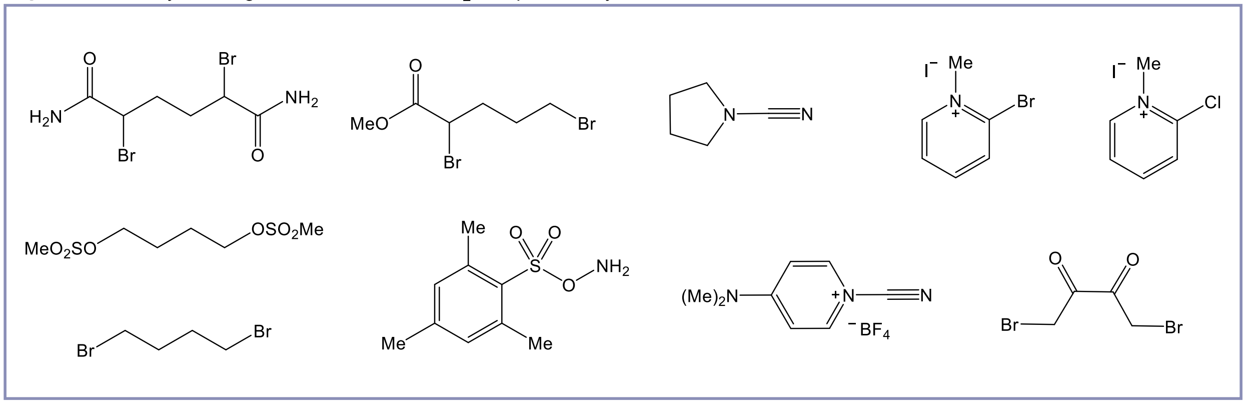

General Procedure for Dha Synthesis

Disulfide-containing peptide (1.0 equivalents, 5-200 mg) was dissolved in ddH_2O to a concentration of 10 mM in a flask or vial containing a magnetic stir bar. TCEP was added next (1.2 equivalents) and the solution stirred for 1 hour at room temperature. DMF was then added to the solution to give a final concentration of 5 mM, followed by 10 equivalents of methyl 2,5-dibromovalerate. After stirring at room temperature for an additional 4 hours, solid K_2CO_3 (10 equivalents) was added and stirring was continued at room temperature. Reaction progress was monitored by LC-MS (time after K_2CO_3 addition ranged from 3-16 hours). 1M aqueous acetic acid was added to quench the reaction until a pH of 5-6 was reached. The solvent was removed under reduced pressure and the Dha_2-peptide purified by RP-flash chromatography (C18; H_2O/0.01%

formic acid to MeCN/0.01% formic acid). The purified peptide was lyophilized and stored at 4°C until use.

Peptide-Dha_2 Characterization

Dha2-eptifibatide

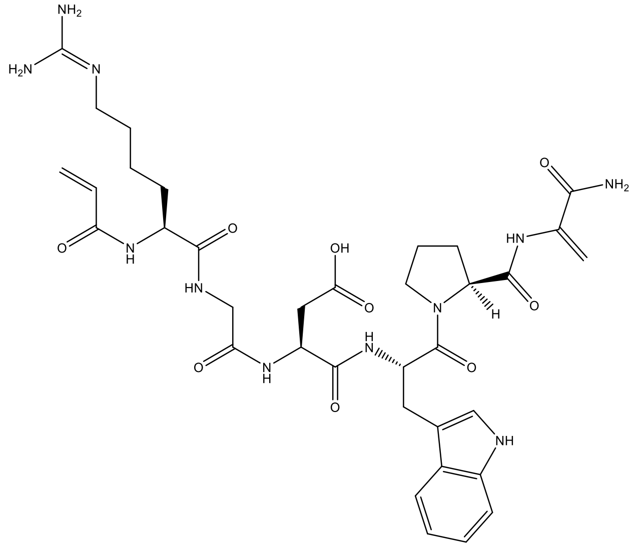

Yield: 8.4 mg from 28.5 mg of eptifibatide (32%)

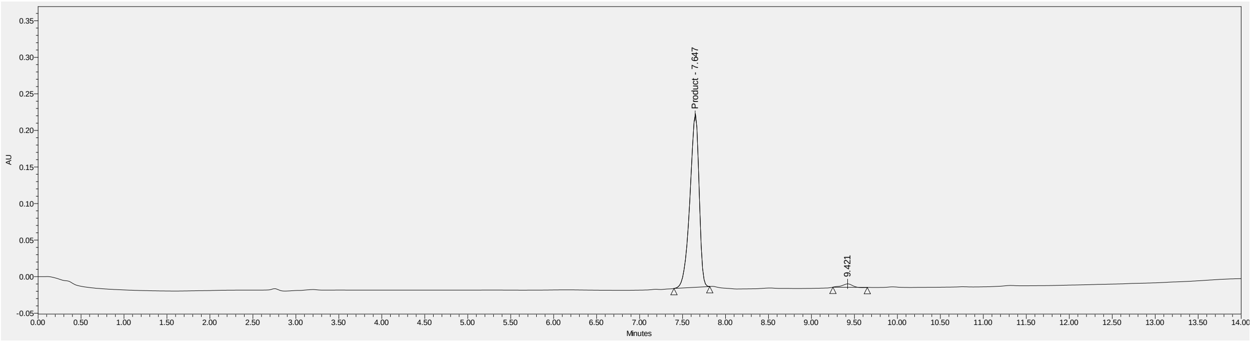

	Name	Retention Time	Area	% Area
1	Product	7.647	1759341	97.84
2		9.421	38914	2.16

HRMS (ESI+)

Calculated for $C_{35}H_{48}N_{11}O_9$ $(M+H)^+$: 766.3631

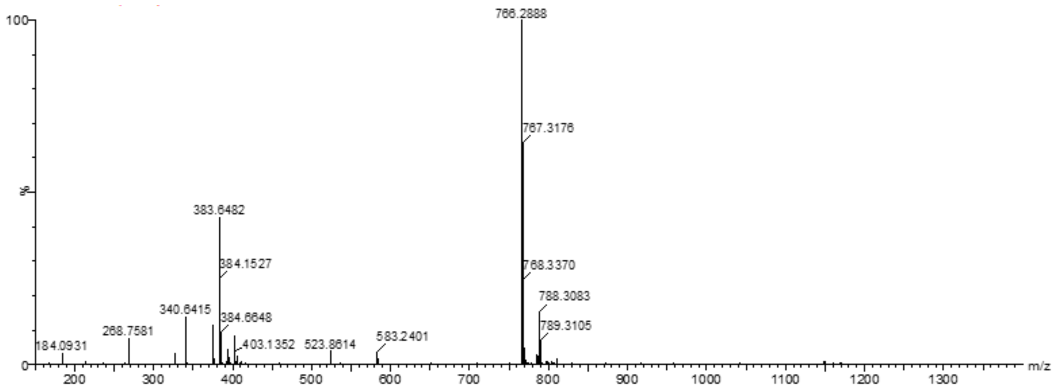

MS/MS (ESI+)

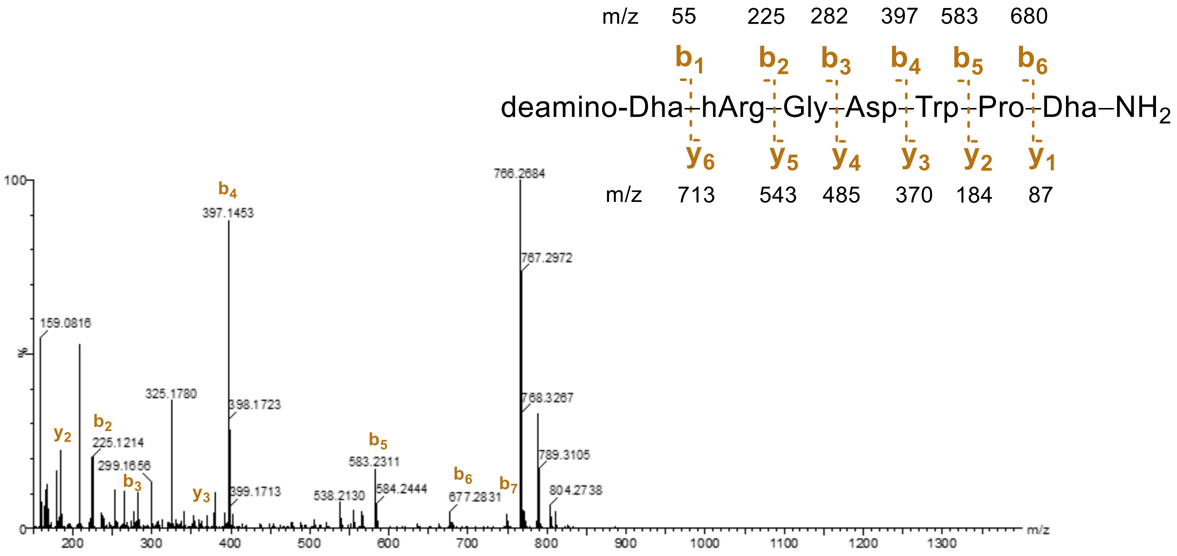

Dha$_2$-terlipressin

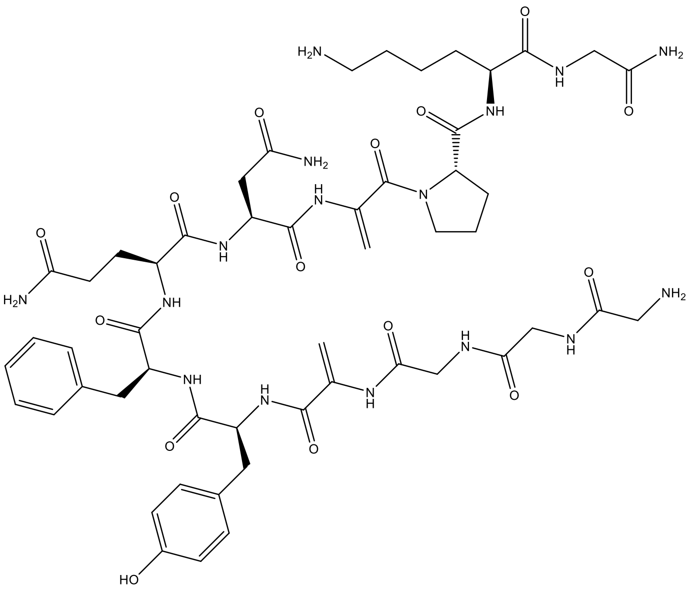

Yield: 22.4 mg from 40.0 mg of terlipressin acetate (59%)

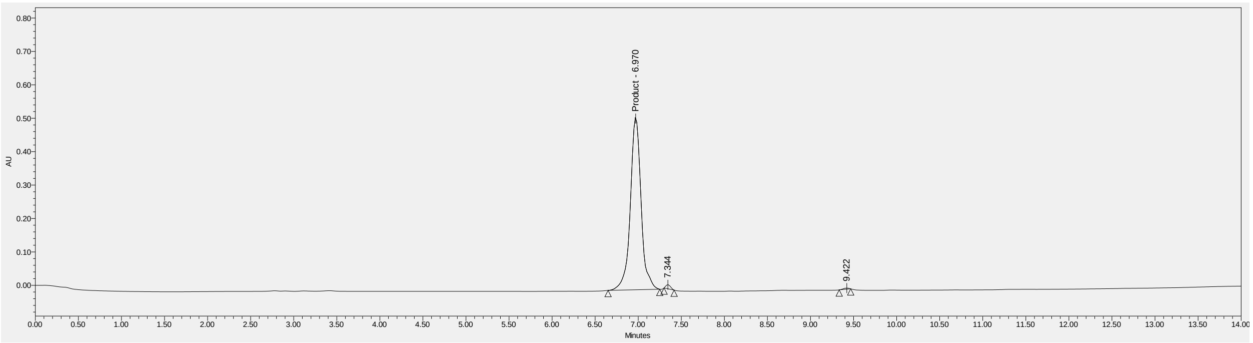

	Name	Retention Time	Area	% Area
1	Product	6.970	4485283	98.66
2		7.344	45373	1.00
3		9.422	15437	0.34

HRMS (ESI+)

Calculated for $C_{52}H_{72}N_{16}O_{15}Na$ $(M+Na)^+$: 1183.5261

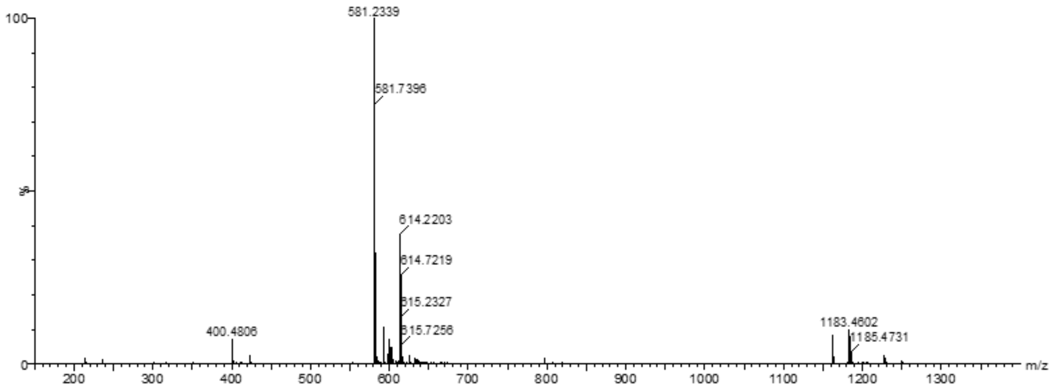

MS/MS (ESI+)

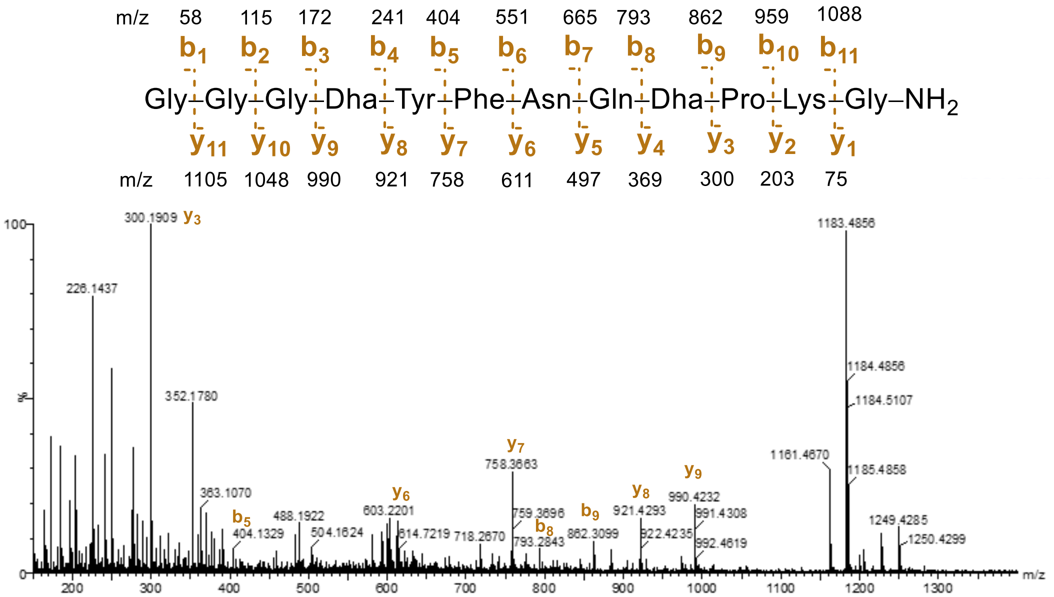

Dha$_2$-vapreotide

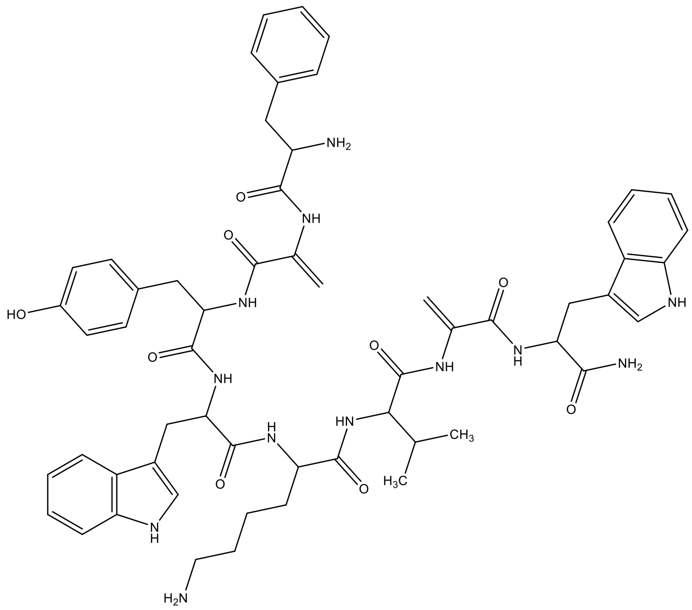

Yield: 19.2 mg from 46.6 mg of vapreotide acetate (44%)

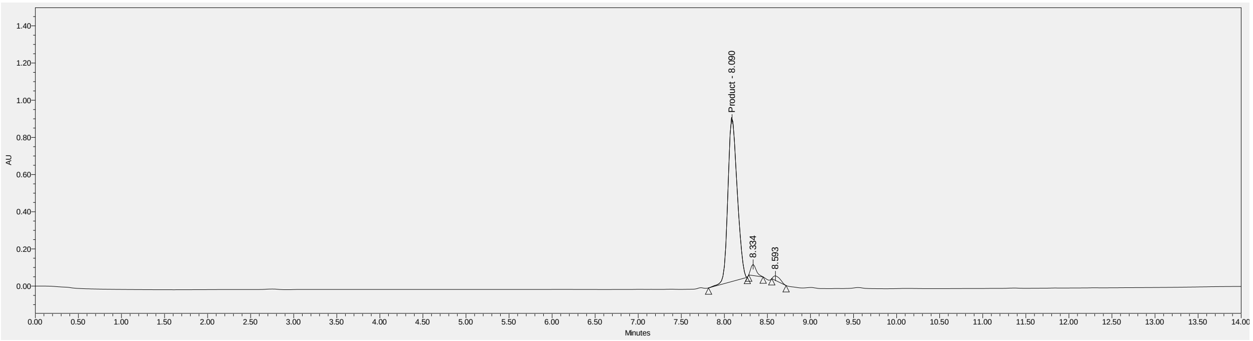

	Name	Retention Time	Area	% Area
1	Product	8.090	6597975	94.24
2		8.334	261256	3.73
3		8.593	141729	2.02

HRMS (ESI+)

Calculated for $C_{57}H_{69}N_{12}O_9$ $(M+H)^+$: 1065.5305

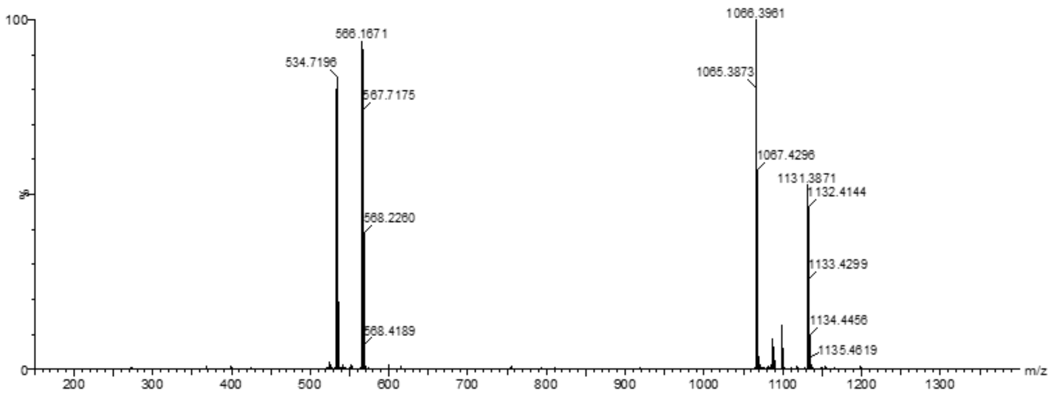

MS/MS (ESI+)

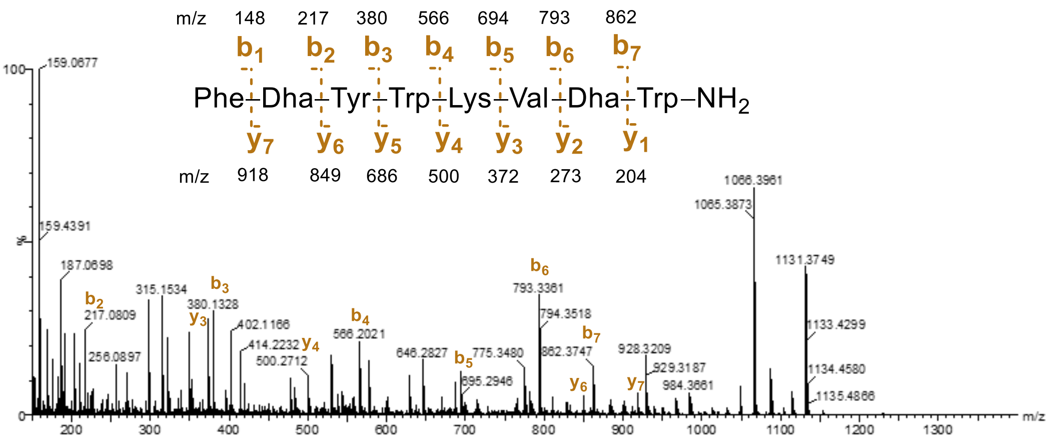

Dha$_2$-setmelanotide

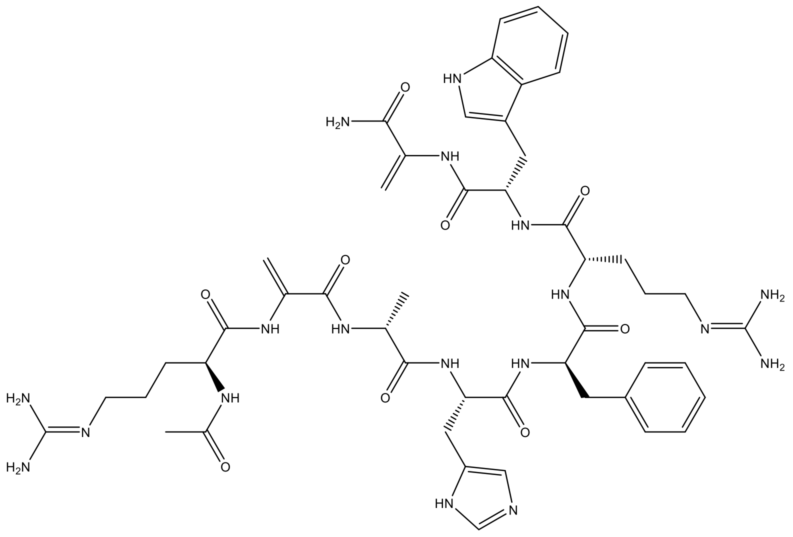

Yield: 71 mg from 200 mg of setmelanotide (38%)

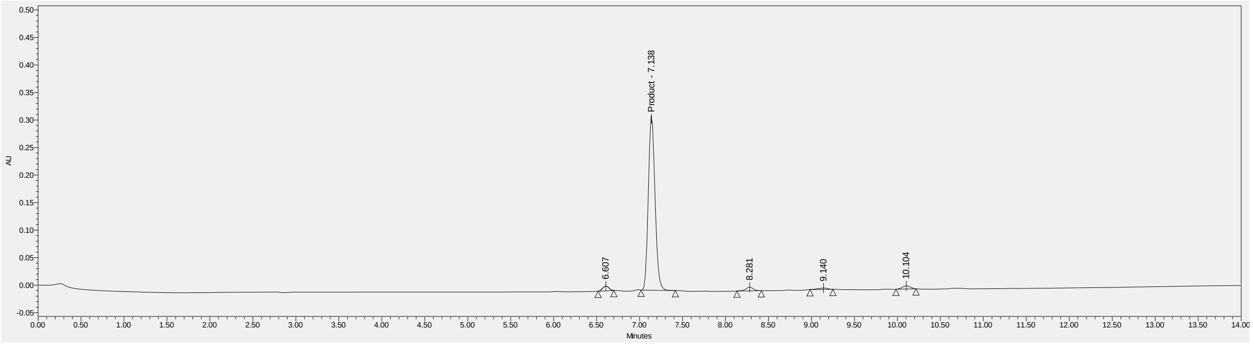

	Name	Retention Time	Area	% Area
1		6.607	44445	2.39
2	Product	7.138	1719226	92.33
3		8.281	40468	2.17
4		9.140	20783	1.12
5		10.104	37126	1.99

HRMS (ESI+)

Calculated for $C_{49}H_{67}N_{18}O_9$ $(M+H)^+$: 1051.5333

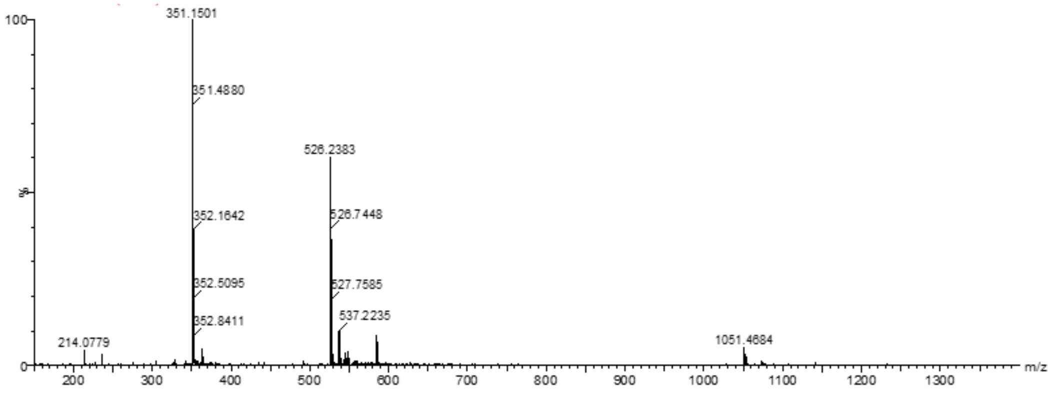

MS/MS (ESI+)

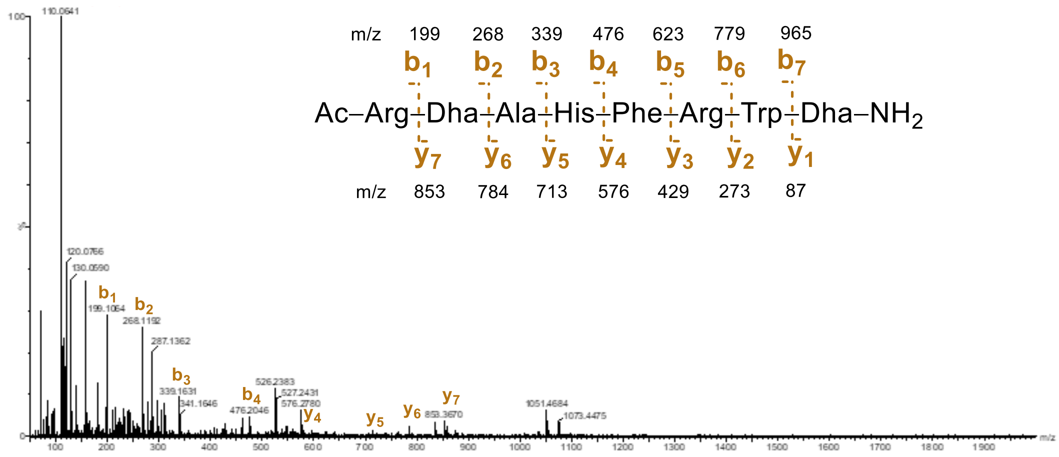

Dha2-desmopressin

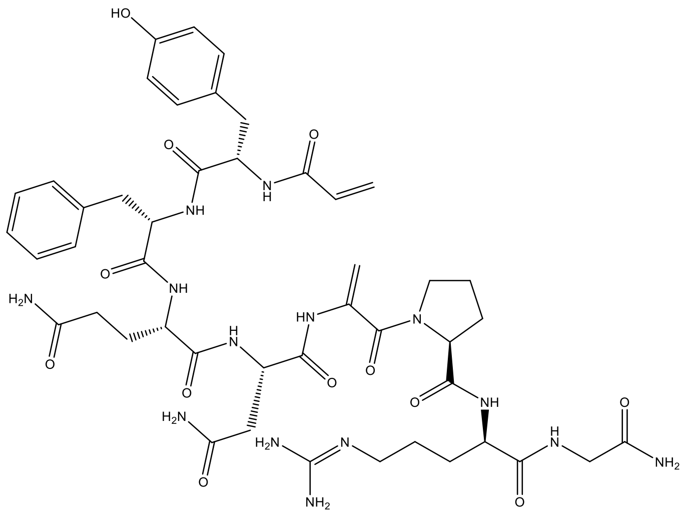

Yield: 9.9 mg from 35.1 mg of desmopressin (34%)

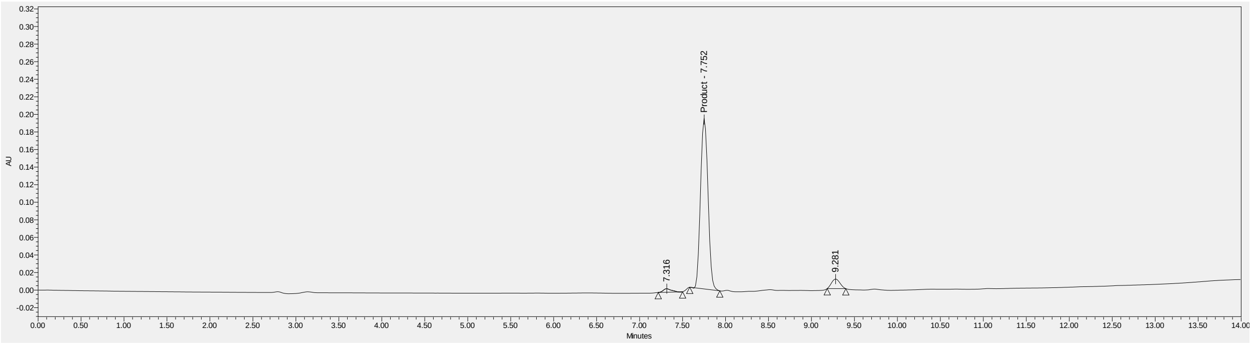

	Name	Retention Time	Area	% Area
1		7.316	26622	2.08
2	Product	7.752	1176711	92.12
3		9.281	73971	5.79

HRMS (ESI+)

Calculated for $C_{46}H_{63}N_{14}O_{12}$ $(M+H)^+$: 1003.4744

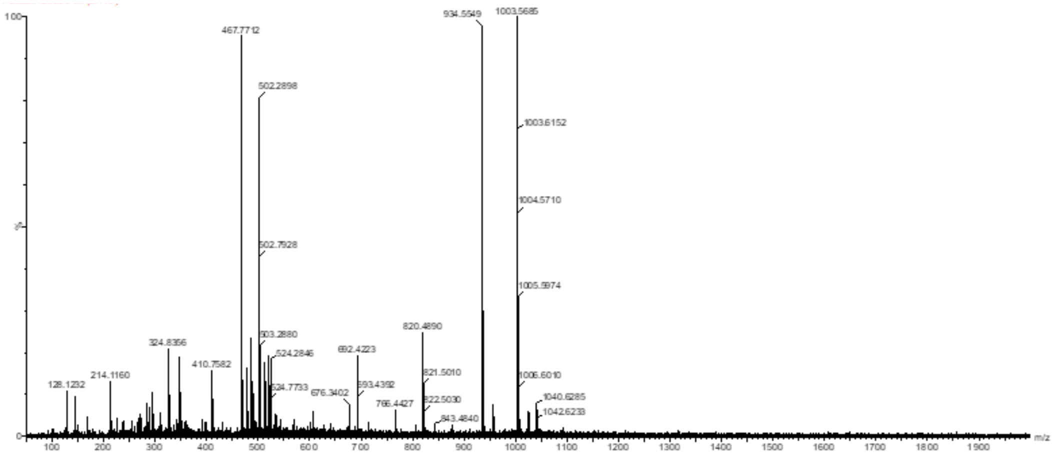

MS/MS (ESI+)

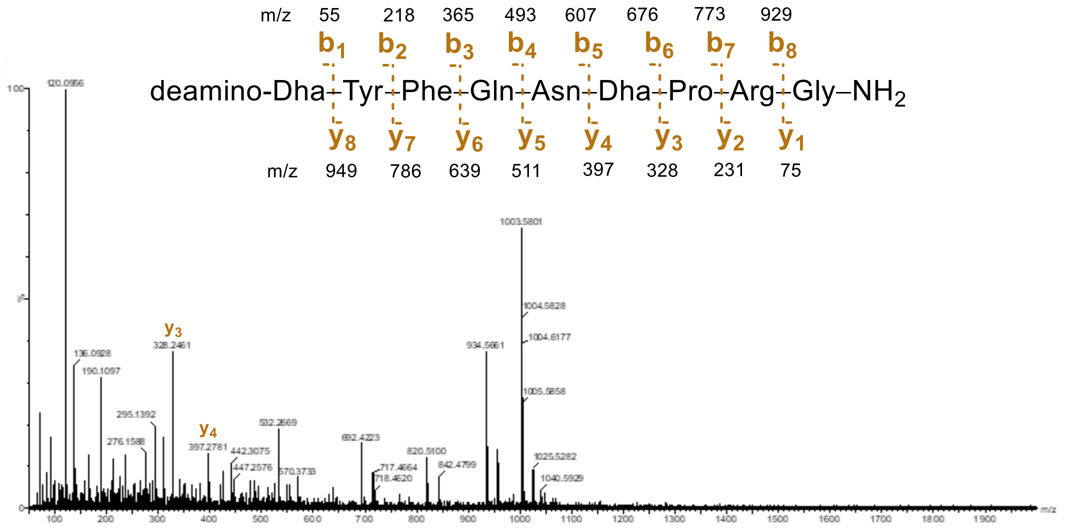

Identifying Platforms for Cyclization

Photochemical Approaches

General Procedure for Photochemical Cyclization Screening

Dha_2-terlipressin (0.3 mg, 259 nmol) was added to a 600 µL vial equipped with a magnetic stir bar. The remaining reagents (as indicated for each reaction) and 259 µL of H_2O:DMSO (1:1) were added sequentially, using stock solutions as appropriate. The complete reaction mixtures were purged with N_2 for 3 minutes, sealed with parafilm, and irradiated under the indicated wavelength for 4 hours. Reactions were then filtered, analyzed by LC-MS, and products verified by HRMS. Conversions indicate the percent area of the product relative to all other peptide-derived products at 214 nm.

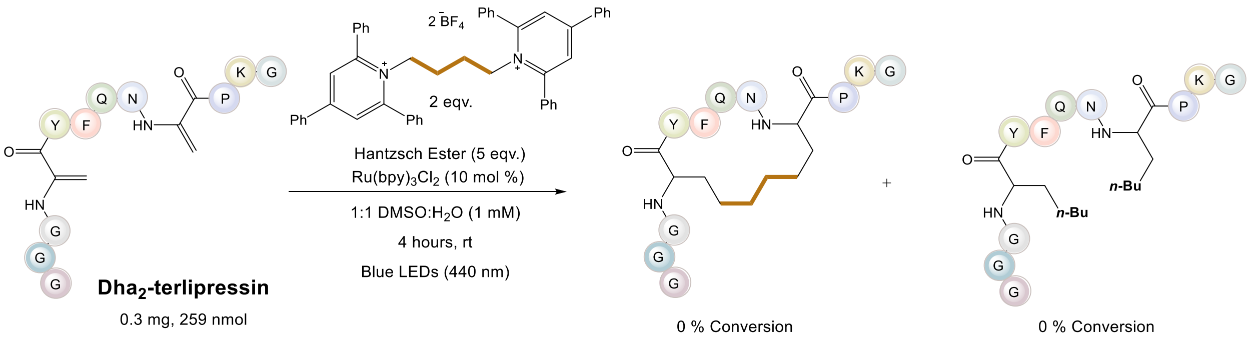

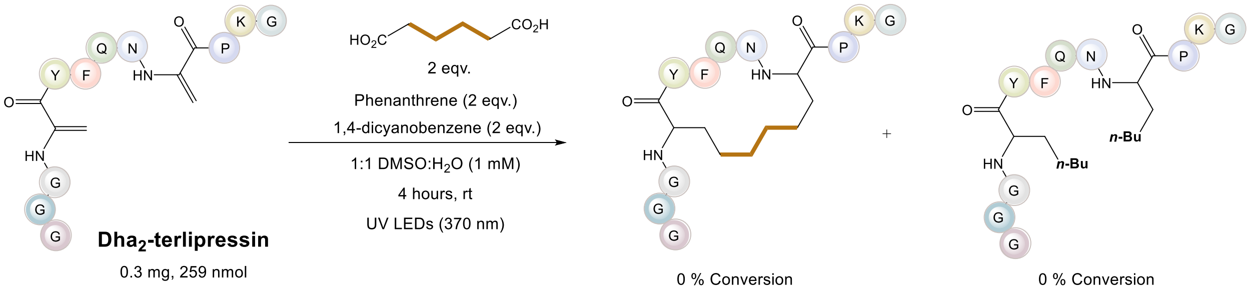

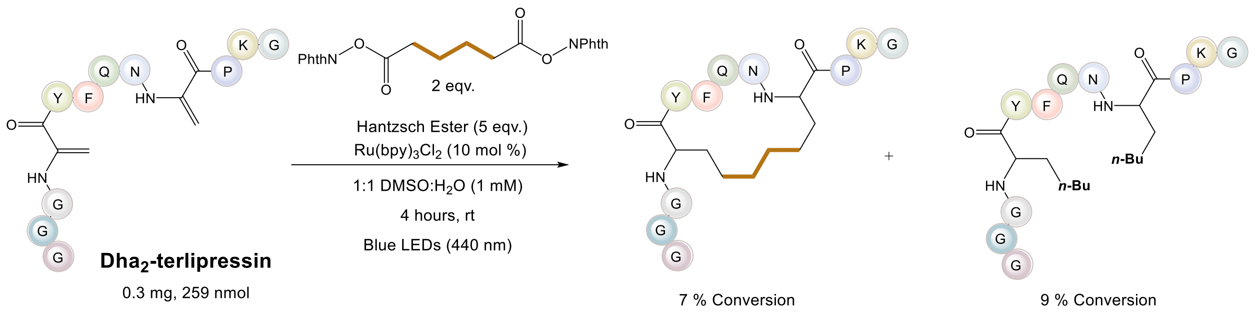
PhthN
NPhth
2 eqv.
Hantzsch Ester (5 eqv.)
Ru(bpy)3Cl2 (10 mol %)
1:1 DMSO:H2O (1 mM)
4 hours, rt
Blue LEDs (440 nm)
Dha2-terlipressin
0.3 mg, 259 nmol
n-Bu
7 % Conversion
9 % Conversion

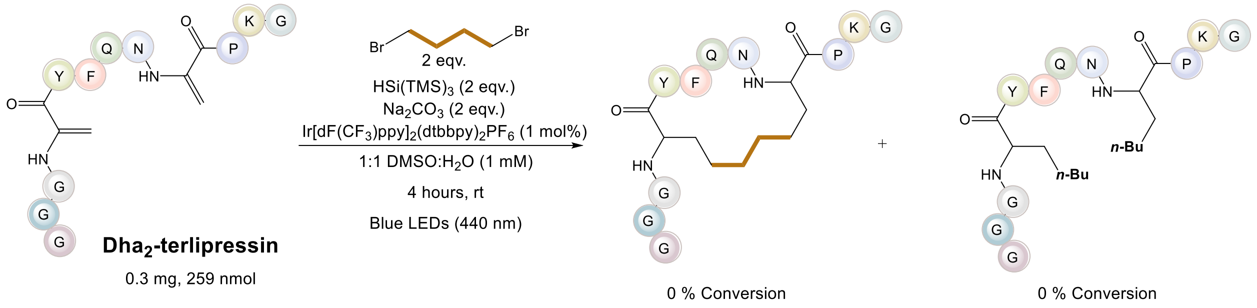
Br
Br
2 eqv.
HSi(TMS)3 (2 eqv.)
Na2CO3 (2 eqv.)
Ir[dF(CF3)ppy]2(dtbbpy)2PF6 (1 mol%)
1:1 DMSO:H2O (1 mM)
4 hours, rt
Blue LEDs (440 nm)
Dha2-terlipressin
0.3 mg, 259 nmol
n-Bu
0 % Conversion
0 % Conversion

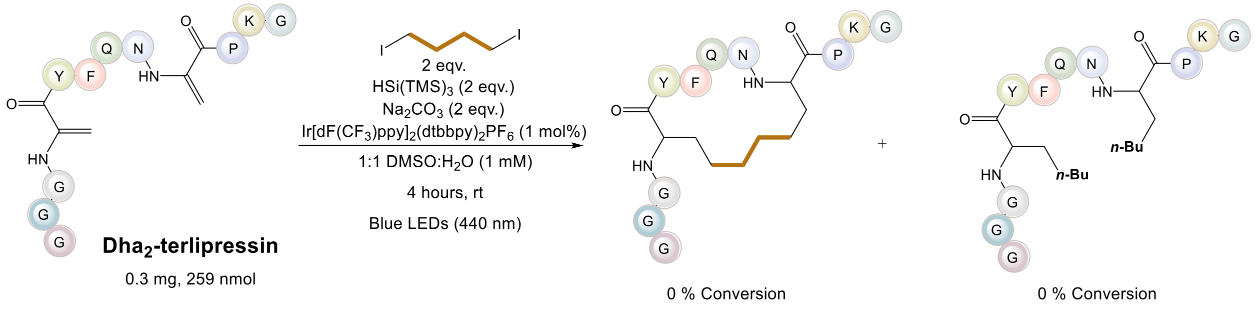
I
I
2 eqv.
HSi(TMS)3 (2 eqv.)
Na2CO3 (2 eqv.)
Ir[dF(CF3)ppy]2(dtbbpy)2PF6 (1 mol%)
1:1 DMSO:H2O (1 mM)
4 hours, rt
Blue LEDs (440 nm)
Dha2-terlipressin
0.3 mg, 259 nmol
n-Bu
0 % Conversion
0 % Conversion

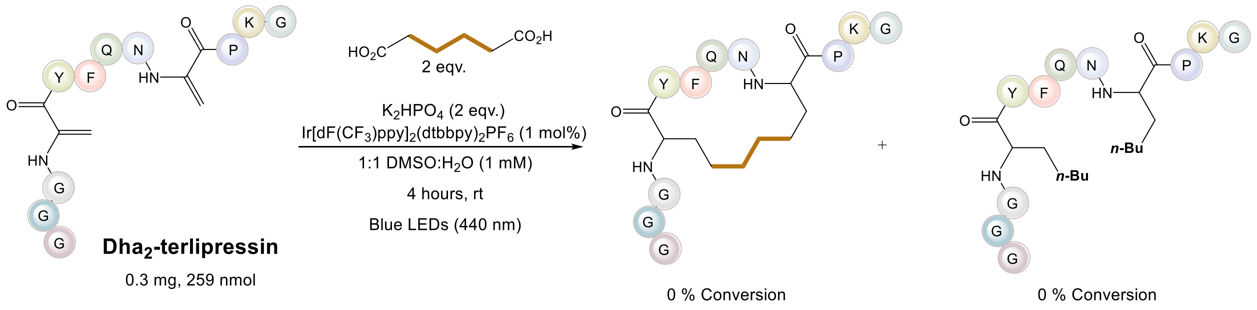
HO2C
CO2H
2 eqv.
K2HPO4 (2 eqv.)
Ir[dF(CF3)ppy]2(dtbbpy)2PF6 (1 mol%)
1:1 DMSO:H2O (1 mM)
4 hours, rt
Blue LEDs (440 nm)
Dha2-terlipressin
0.3 mg, 259 nmol
n-Bu
0 % Conversion
0 % Conversion

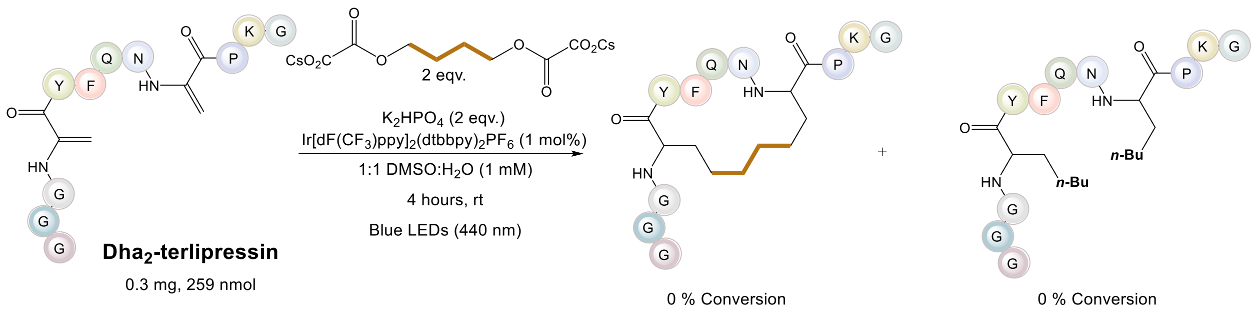
CsO2C
CO2Cs
2 eqv.
K2HPO4 (2 eqv.)
Ir[dF(CF3)ppy]2(dtbbpy)2PF6 (1 mol%)
1:1 DMSO:H2O (1 mM)
4 hours, rt
Blue LEDs (440 nm)
Dha2-terlipressin
0.3 mg, 259 nmol
n-Bu
0 % Conversion
0 % Conversion

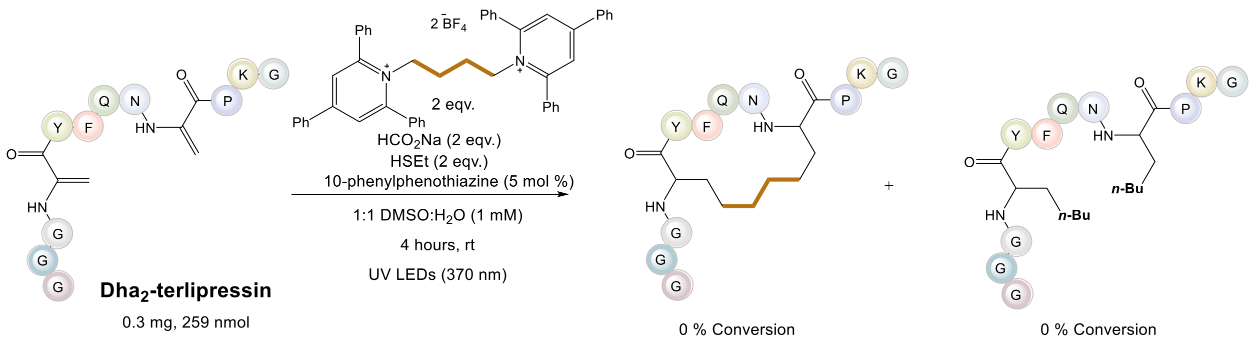
2 BF4
Ph
2 eqv.
HCO2Na (2 eqv.)
HSEt (2 eqv.)
10-phenylphenothiazine (5 mol %)
1:1 DMSO:H2O (1 mM)
4 hours, rt
UV LEDs (370 nm)
Dha2-terlipressin
0.3 mg, 259 nmol
n-Bu
0 % Conversion
0 % Conversion

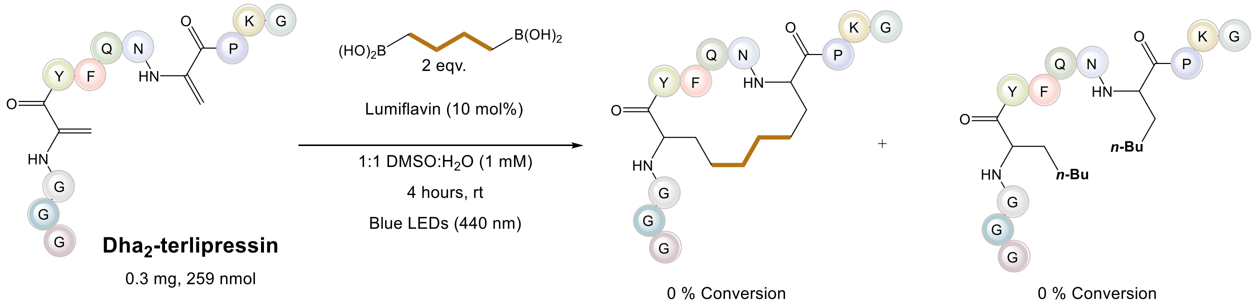
(HO)2B
B(OH)2
2 eqv.
Lumiflavin (10 mol%)
1:1 DMSO:H2O (1 mM)
4 hours, rt
Blue LEDs (440 nm)
Dha2-terlipressin
0.3 mg, 259 nmol
n-Bu
0 % Conversion
0 % Conversion

Table S1. Photocatalyst Screen for Cyclization with Hantzsch Ester Under Blue LEDs.

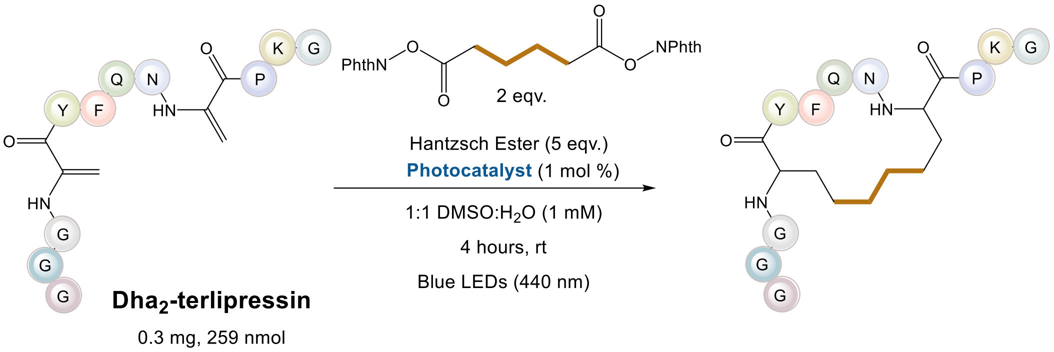

Photocatalyst	% Conversion
$Ru(bpm)_3Cl_2$	0 %
$Ru(bpy)_3Cl_2$	4 %
$Ru(bpz)_3(PF_6)_2$	0 %
$Ru(dtbbpy)_3(PF_6)_2$	trace
$Ru(phen)_3Cl_2$	0 %
$Ir(ppy)_3$	0 %
$Ir[dF(CF_3)ppy]_2(dtbbpy)\ (PF_6)$	0 %
(Mes-Acr) ClO_4	0 %
meso-tetraphenylporphine	0 %
10-phenylphenothiazine	0 %
3,6-diaminoacridine HCl	0 %
$Ir[dF(CF_3)ppy]_2(bpy)\ (PF_6)$	0 %

Table S2. Photocatalyst Screen for Cyclization with BNAH Under Blue LEDs.

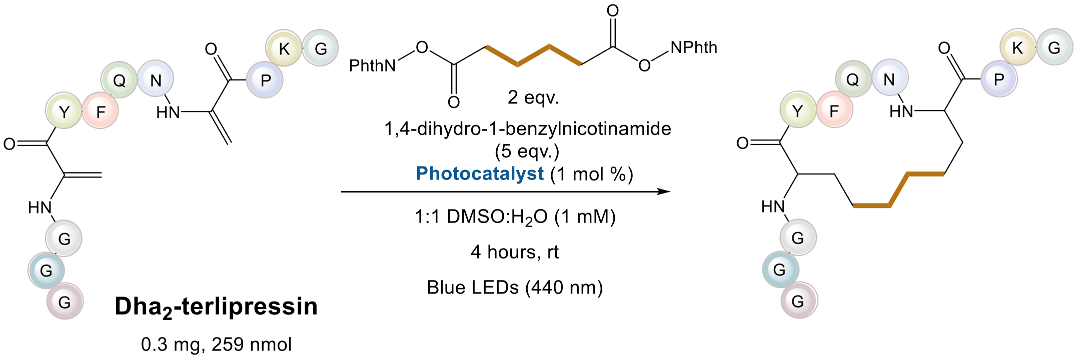

Photocatalyst	% Conversion
$Ru(bpy)_3Cl_2$	3 %
$Ir[dF(CF_3)ppy]_2(dtbbpy)\ (PF_6)$	4 %
10-phenylphenothiazine	0 %

Table S3. Photocatalyst Screen for Cyclization with Hantzsch Ester Under Green LEDs

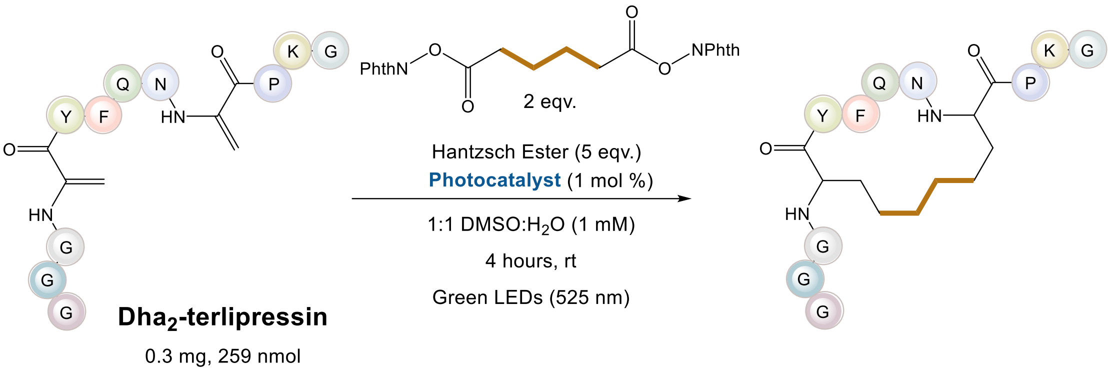

Photocatalyst	% Conversion
Fluorescein	0 %
Eosin Y	trace
Cu(dap)$_2$Cl	0 %
Erythrosin	0 %
Rhodamine B	0 %
Eosin B	3 %
Rose Bengal	0 %

Table S4. Photocatalyst Screen for Cyclization with BNAH Under Green LEDs.

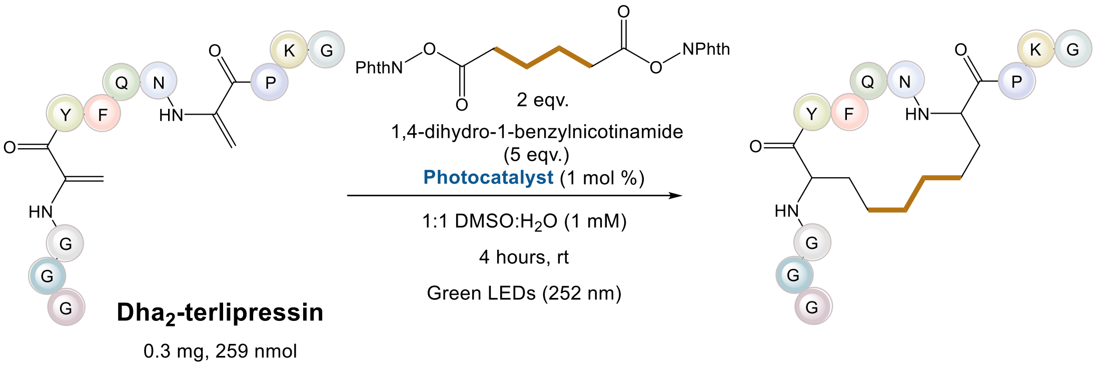

Photocatalyst	% Conversion
Fluorescein	trace
Rhodamine B	3 %
Rose Bengal	10 %
Eosin Y	0 %

Electrochemical Approaches

General Procedure for Terlipressin-Dha$_2$ Electrochemical Cyclization

In a 10 mL ElectraSyn vial with a magnetic stir bar, terlipressin-Dha$_2$ (861 nmol, 1.0 mg) was dissolved in 7.00 mL of 1:1 H_2O: MeCN containing 0.2 M $LiClO_4$. 3.0 equivalents of **X**-butyl-**X** (2.58 µmol) and 3.0 equivalents of additive (2.58 µmol) were added, and the resulting solution was capped and purged with N_2 for 3 minutes. 5.0 F/mol electron equivalents were delivered and a constant current of 5.0 mA. The crude reaction was analyzed by LC/MS.

Table S5. Attempted Electrochemical Cyclization of Terlipressin

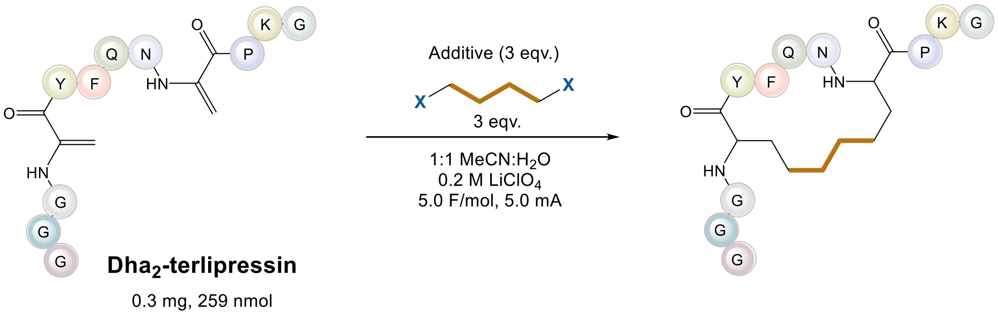

X	Anode:Cathode	Additive	% Conversion
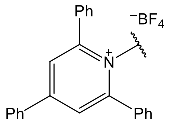	Zn:Mg	—	0 %
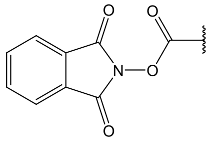	Zn:Mg	—	0 %
Br	Zn:Mg	—	0 %
I	Zn:Mg	—	0 %
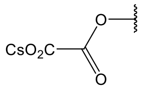	graphite:graphite	NHPI	0 %
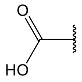	graphite:graphite	NHPI	0 %
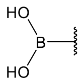	graphite:graphite	NHPI	0 %

General Procedure for Methyl 2-Acetamido Acrylate Alkylation

In a 10 mL ElectraSyn vial with a magnetic stir bar, methyl 2-acetamidoacrylate (70 µmol, 10.0 mg) was dissolved in 7.00 mL of 1:1 H_2O: MeCN containing 0.2 M $LiClO_4$. 3.0 equivalents of 2-propyl-**X** (210 µmol) and 3.0 equivalents of additive (210 µmol) were added, and the resulting solution was capped and purged with N_2 for 3 minutes. 2.5 F/mol electron equivalents were delivered and a constant current of 5.0 mA. The crude reaction was analyzed by TLC (hexanes:EtOAc 1:1, UV_{254} and $KMnO_4$) in comparison to authentic material. Unidentified products were further analyzed by ^{1}H NMR. **None of the conditions screened (listed below) provided the desired alkylated product.**

Table S6. Electrochemical Alkylation with Boronic Acid and Formic Acid

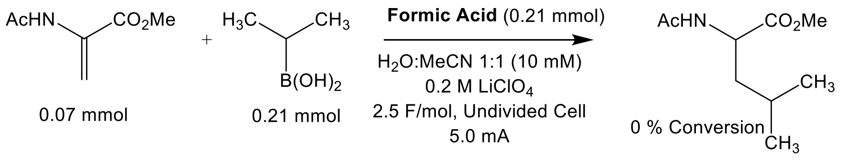

Anode	Cathode
RVC	Zinc
RVC	Magnesium
RVC	Stainless Steel
RVC	RVC
RVC	Graphite
Stainless Steel	Zinc
Stainless Steel	Magnesium
Stainless Steel	Stainless Steel
Stainless Steel	RVC
Stainless Steel	Graphite
Graphite	Zinc
Graphite	Magnesium
Graphite	Stainless Steel
Graphite	RVC
Graphite	Graphite

Table S7. Electrochemical Alkylation with Boronic Acid and NHPI

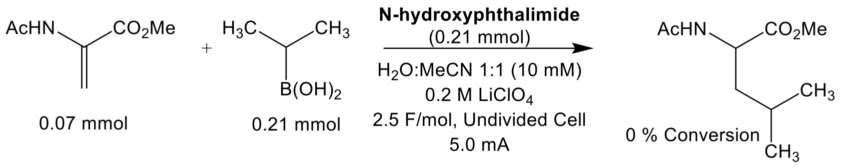

Anode	Cathode
RVC	Zinc
RVC	Magnesium
RVC	Stainless Steel
RVC	RVC
RVC	Graphite
Stainless Steel	Zinc
Stainless Steel	Magnesium
Stainless Steel	Stainless Steel
Stainless Steel	RVC
Stainless Steel	Graphite
Graphite	Zinc
Graphite	Magnesium
Graphite	Stainless Steel
Graphite	RVC
Graphite	Graphite

Table S8. Electrochemical Alkylation with Boronic Acid and Benzoic Acid

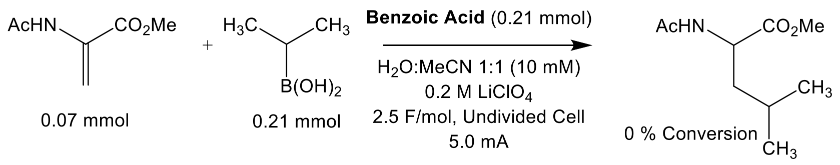

Anode	Cathode
RVC	Zinc
RVC	Magnesium
RVC	Stainless Steel
RVC	RVC
RVC	Graphite
Stainless Steel	Zinc
Stainless Steel	Magnesium
Stainless Steel	Stainless Steel
Stainless Steel	RVC
Stainless Steel	Graphite
Graphite	Zinc
Graphite	Magnesium
Graphite	Stainless Steel
Graphite	RVC
Graphite	Graphite

Table S9. Electrochemical Alkylation with Boronic Acid and L-Cysteine

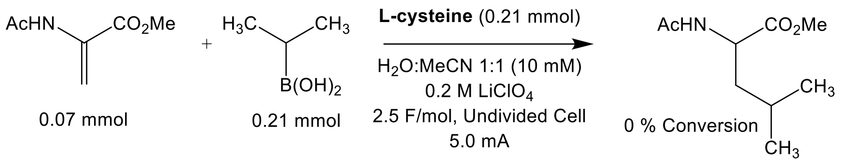

Anode	Cathode
RVC	Zinc
RVC	Magnesium
RVC	Stainless Steel
RVC	RVC
RVC	Graphite
Stainless Steel	Zinc
Stainless Steel	Magnesium
Stainless Steel	Stainless Steel
Stainless Steel	RVC
Stainless Steel	Graphite
Graphite	Zinc
Graphite	Magnesium
Graphite	Stainless Steel
Graphite	RVC
Graphite	Graphite

Table S10. Electrochemical Alkylation with Oxalate and Formic Acid

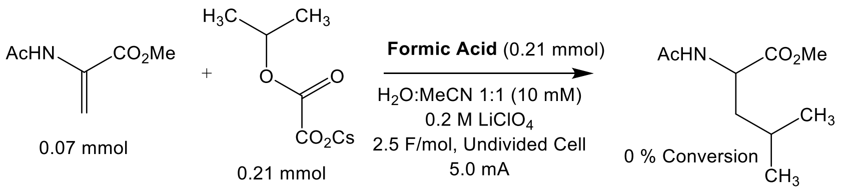

Anode	Cathode
RVC	Zinc
RVC	Magnesium
RVC	Stainless Steel
RVC	RVC
RVC	Graphite
Stainless Steel	Zinc
Stainless Steel	Magnesium
Stainless Steel	Stainless Steel
Stainless Steel	RVC
Stainless Steel	Graphite
Graphite	Zinc
Graphite	Magnesium
Graphite	Stainless Steel
Graphite	RVC
Graphite	Graphite

Table S11. Electrochemical Alkylation with Oxalate and NHPI

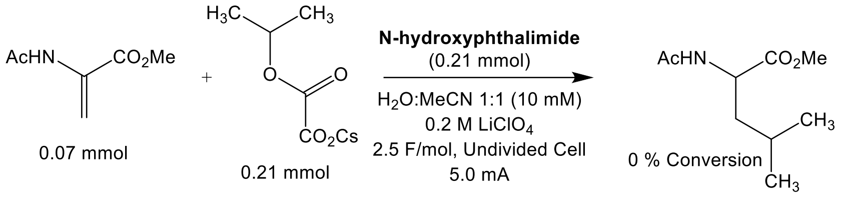

Anode	Cathode
RVC	Zinc
RVC	Magnesium
RVC	Stainless Steel
RVC	RVC
RVC	Graphite
Stainless Steel	Zinc
Stainless Steel	Magnesium
Stainless Steel	Stainless Steel
Stainless Steel	RVC
Stainless Steel	Graphite
Graphite	Zinc
Graphite	Magnesium
Graphite	Stainless Steel
Graphite	RVC
Graphite	Graphite

Table S12. Electrochemical Alkylation with Oxalate and Benzoic Acid

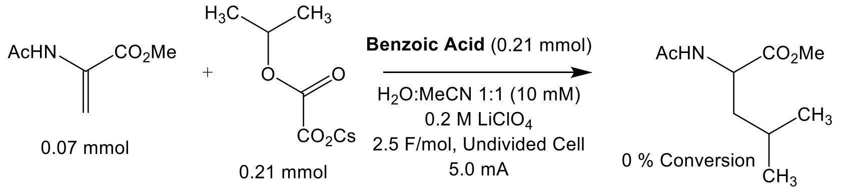

Anode	Cathode
RVC	Zinc
RVC	Magnesium
RVC	Stainless Steel
RVC	RVC
RVC	Graphite
Stainless Steel	Zinc
Stainless Steel	Magnesium
Stainless Steel	Stainless Steel
Stainless Steel	RVC
Stainless Steel	Graphite
Graphite	Zinc
Graphite	Magnesium
Graphite	Stainless Steel
Graphite	RVC
Graphite	Graphite

Table S13. Electrochemical Alkylation with Oxalate and L-Cysteine

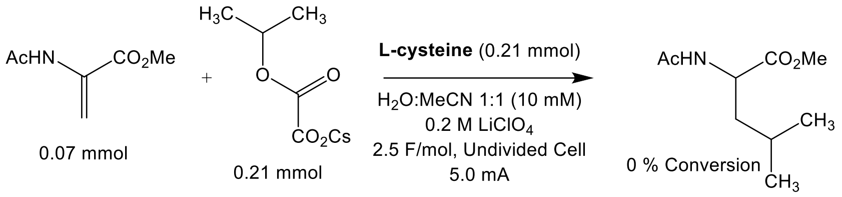

Anode	Cathode
RVC	Zinc
RVC	Magnesium
RVC	Stainless Steel
RVC	RVC
RVC	Graphite
Stainless Steel	Zinc
Stainless Steel	Magnesium
Stainless Steel	Stainless Steel
Stainless Steel	RVC
Stainless Steel	Graphite
Graphite	Zinc
Graphite	Magnesium
Graphite	Stainless Steel
Graphite	RVC
Graphite	Graphite

Table S14. Electrochemical Alkylation with Carboxylate and Formic Acid

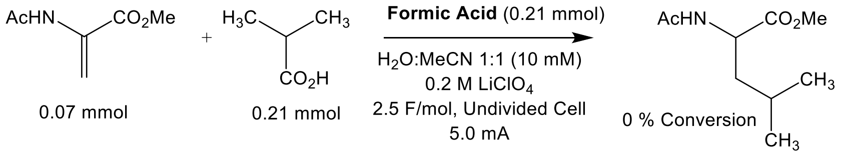

Anode	Cathode
RVC	Zinc
RVC	Magnesium
RVC	Stainless Steel
RVC	RVC
RVC	Graphite
Stainless Steel	Zinc
Stainless Steel	Magnesium
Stainless Steel	Stainless Steel
Stainless Steel	RVC
Stainless Steel	Graphite
Graphite	Zinc
Graphite	Magnesium
Graphite	Stainless Steel
Graphite	RVC
Graphite	Graphite

Table S15. Electrochemical Alkylation with Carboxylate and NHPI

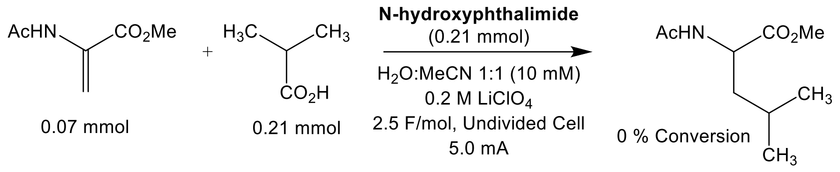

**Anode**	**Cathode**
RVC	Zinc
RVC	Magnesium
RVC	Stainless Steel
RVC	RVC
RVC	Graphite
Stainless Steel	Zinc
Stainless Steel	Magnesium
Stainless Steel	Stainless Steel
Stainless Steel	RVC
Stainless Steel	Graphite
Graphite	Zinc
Graphite	Magnesium
Graphite	Stainless Steel
Graphite	RVC
Graphite	Graphite

Table S16. Electrochemical Alkylation with Carboxylate and Benzoic Acid

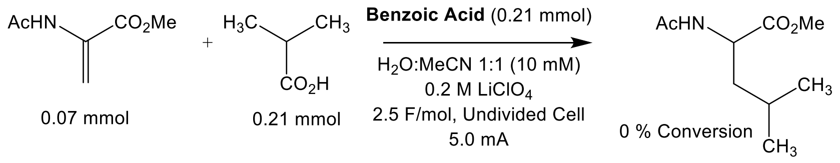

**Anode**	**Cathode**
RVC	Zinc
RVC	Magnesium
RVC	Stainless Steel
RVC	RVC
RVC	Graphite
Stainless Steel	Zinc
Stainless Steel	Magnesium
Stainless Steel	Stainless Steel
Stainless Steel	RVC
Stainless Steel	Graphite
Graphite	Zinc
Graphite	Magnesium
Graphite	Stainless Steel
Graphite	RVC
Graphite	Graphite

Table S17. Electrochemical Alkylation with Carboxylate and L-Cysteine

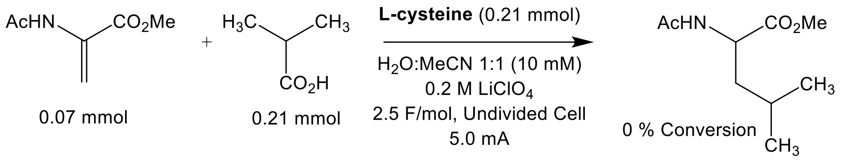

Anode	Cathode
RVC	Zinc
RVC	Magnesium
RVC	Stainless Steel
RVC	RVC
RVC	Graphite
Stainless Steel	Zinc
Stainless Steel	Magnesium
Stainless Steel	Stainless Steel
Stainless Steel	RVC
Stainless Steel	Graphite
Graphite	Zinc
Graphite	Magnesium
Graphite	Stainless Steel
Graphite	RVC
Graphite	Graphite

Table S18. Electrochemical Alkylation with Pyridinium and Hantzsch Ester

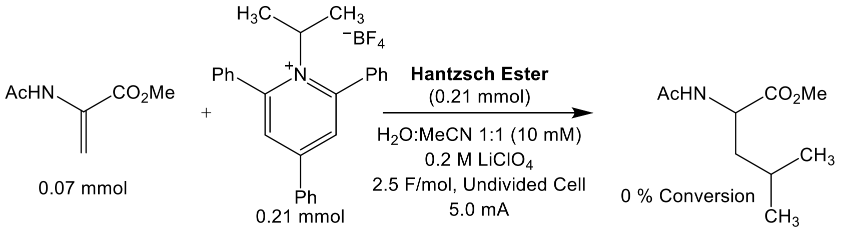

Anode	Cathode
Zinc	Zinc
Zinc	Magnesium
Zinc	Nickel Foam
Zinc	Stainless Steel
Zinc	Graphite
Zinc	RVC
Magnesium	Zinc
Magnesium	Magnesium
Magnesium	Nickel Foam
Magnesium	Stainless Steel
Magnesium	Graphite
Magnesium	RVC
Nickel Foam	Zinc
Nickel Foam	Magnesium
Nickel Foam	Nickel Foam
Nickel Foam	Stainless Steel
Nickel Foam	Graphite
Nickel Foam	RVC

Table S19. Electrochemical Alkylation with Iodide and Hantzsch Ester

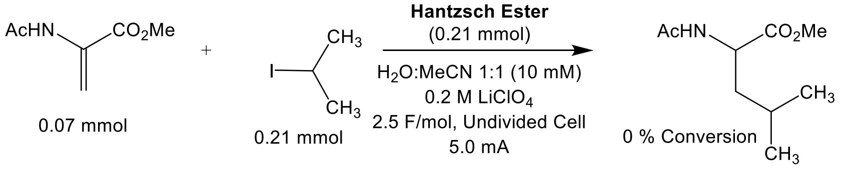

Anode	Cathode
Zinc	Zinc
Zinc	Magnesium
Zinc	Nickel Foam
Zinc	Stainless Steel
Zinc	Graphite
Zinc	RVC
Magnesium	Zinc
Magnesium	Magnesium
Magnesium	Nickel Foam
Magnesium	Stainless Steel
Magnesium	Graphite
Magnesium	RVC
Nickel Foam	Zinc
Nickel Foam	Magnesium
Nickel Foam	Nickel Foam
Nickel Foam	Stainless Steel
Nickel Foam	Graphite
Nickel Foam	RVC

Table S20. Electrochemical Alkylation with Bromide and Hantzsch Ester

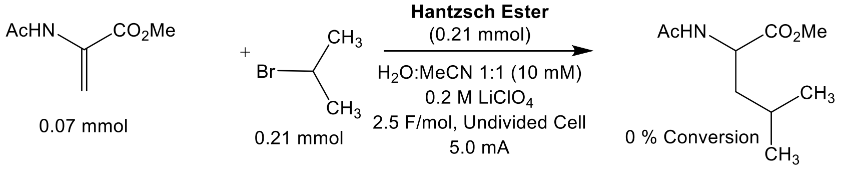

Anode	Cathode
Zinc	Zinc
Zinc	Magnesium
Zinc	Nickel Foam
Zinc	Stainless Steel
Zinc	Graphite
Zinc	RVC
Magnesium	Zinc
Magnesium	Magnesium
Magnesium	Nickel Foam
Magnesium	Stainless Steel
Magnesium	Graphite
Magnesium	RVC
Nickel Foam	Zinc
Nickel Foam	Magnesium
Nickel Foam	Nickel Foam
Nickel Foam	Stainless Steel
Nickel Foam	Graphite
Nickel Foam	RVC

Table S21. Electrochemical Alkylation with NHPI Ester and Hantzsch Ester

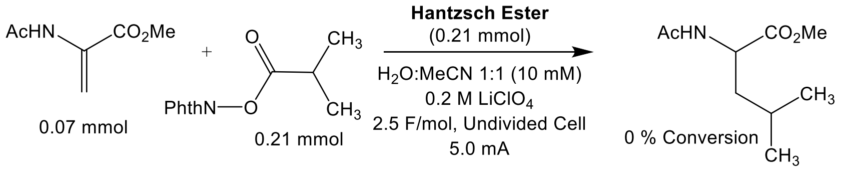

Anode	Cathode
Zinc	Zinc
Zinc	Magnesium
Zinc	Nickel Foam
Zinc	Stainless Steel
Zinc	Graphite
Zinc	RVC
Magnesium	Zinc
Magnesium	Magnesium
Magnesium	Nickel Foam
Magnesium	Stainless Steel
Magnesium	Graphite
Magnesium	RVC
Nickel Foam	Zinc
Nickel Foam	Magnesium
Nickel Foam	Nickel Foam
Nickel Foam	Stainless Steel
Nickel Foam	Graphite
Nickel Foam	RVC

Table S22. Electrochemical Alkylation with Pyridinium

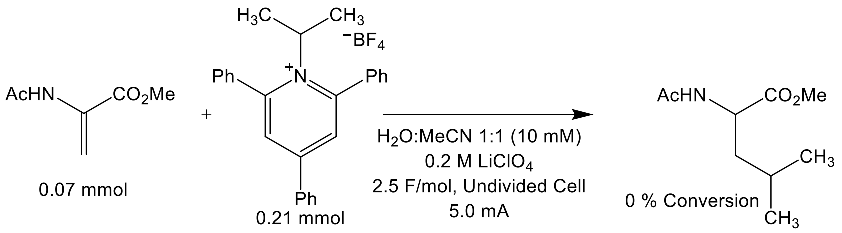

Anode	Cathode
Zinc	Zinc
Zinc	Magnesium
Zinc	Nickel Foam
Zinc	Stainless Steel
Zinc	Graphite
Zinc	RVC
Magnesium	Zinc
Magnesium	Magnesium
Magnesium	Nickel Foam
Magnesium	Stainless Steel
Magnesium	Graphite
Magnesium	RVC
Nickel Foam	Zinc
Nickel Foam	Magnesium
Nickel Foam	Nickel Foam
Nickel Foam	Stainless Steel
Nickel Foam	Graphite
Nickel Foam	RVC

Table S23. Electrochemical Alkylation with Iodide

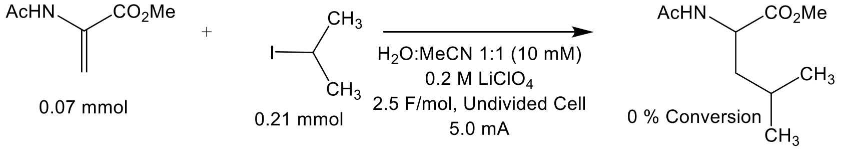

Anode	Cathode
Zinc	Zinc
Zinc	Magnesium
Zinc	Nickel Foam
Zinc	Stainless Steel
Zinc	Graphite
Zinc	RVC
Magnesium	Zinc
Magnesium	Magnesium
Magnesium	Nickel Foam
Magnesium	Stainless Steel
Magnesium	Graphite
Magnesium	RVC
Nickel Foam	Zinc
Nickel Foam	Magnesium
Nickel Foam	Nickel Foam
Nickel Foam	Stainless Steel
Nickel Foam	Graphite
Nickel Foam	RVC

Table S24. Electrochemical Alkylation with Bromide

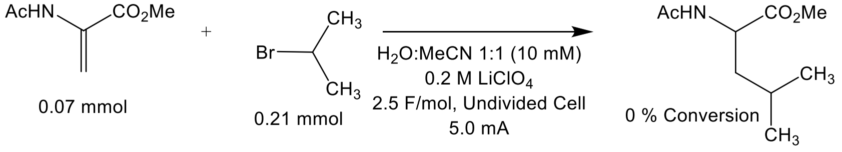

Anode	Cathode
Zinc	Zinc
Zinc	Magnesium
Zinc	Nickel Foam
Zinc	Stainless Steel
Zinc	Graphite
Zinc	RVC
Magnesium	Zinc
Magnesium	Magnesium
Magnesium	Nickel Foam
Magnesium	Stainless Steel
Magnesium	Graphite
Magnesium	RVC
Nickel Foam	Zinc
Nickel Foam	Magnesium
Nickel Foam	Nickel Foam
Nickel Foam	Stainless Steel
Nickel Foam	Graphite
Nickel Foam	RVC

Table S25. Electrochemical Alkylation with NHPI Ester

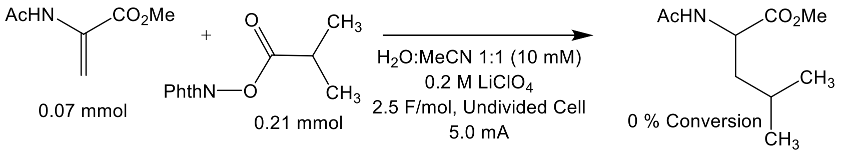

Anode	Cathode
Zinc	Zinc
Zinc	Magnesium
Zinc	Nickel Foam
Zinc	Stainless Steel
Zinc	Graphite
Zinc	RVC
Magnesium	Zinc
Magnesium	Magnesium
Magnesium	Nickel Foam
Magnesium	Stainless Steel
Magnesium	Graphite
Magnesium	RVC
Nickel Foam	Zinc
Nickel Foam	Magnesium
Nickel Foam	Nickel Foam
Nickel Foam	Stainless Steel
Nickel Foam	Graphite
Nickel Foam	RVC

Organometallic Approaches

General Procedure for Organometallic Cyclization

Terlipressin-Dha$_2$ (0.3 mg, 259 nmol) was dissolved in 0.233 mL of 1:9 DMSO:H_2O in a 600 µL vial equipped with a magnetic stir bar. The indicated linker (1.30 µmol) was dissolved in DMSO (26 µL), which was subsequently added to the aqueous peptide. The listed metal (15.5 µmol) was then added, and the mixture was capped, purged with N_2 (3 minutes), and sealed with parafilm. After stirring at room temperature for 8 hours, the reaction was filtered and analyzed by LC-MS. Reactions in which products were observed were verified by HRMS.

Table S26. Screening of Organometallic Approaches for Cyclization

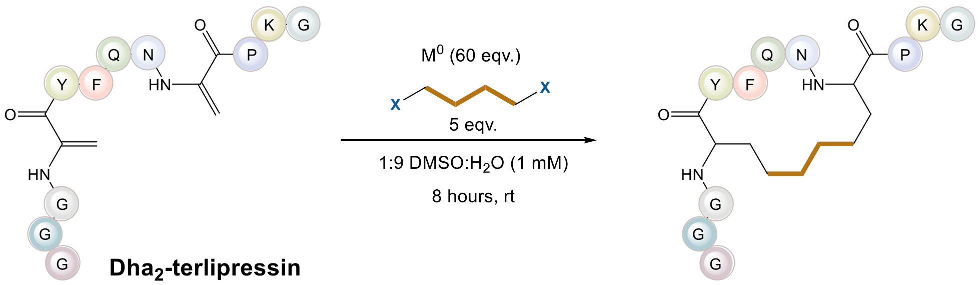

Dha$_2$-terlipressin
0.3 mg, 259 nmol

X	M^0	% Conversion
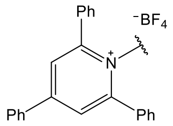	Zn	0 %
	Mg	0 %
	In	0 %
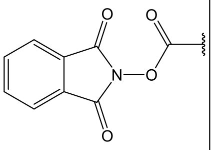	Zn	0 %
	Mg	0 %
	In	0 %
Br–	Zn	trace
	Mg	0 %
	In	trace
I–	Zn	8 %
	Mg	0 %
	In	0 %

Representative LC Traces

Table 1, Entry 2

Ru(bpy)3, HEH, bis-NHPI ester, 445 nm

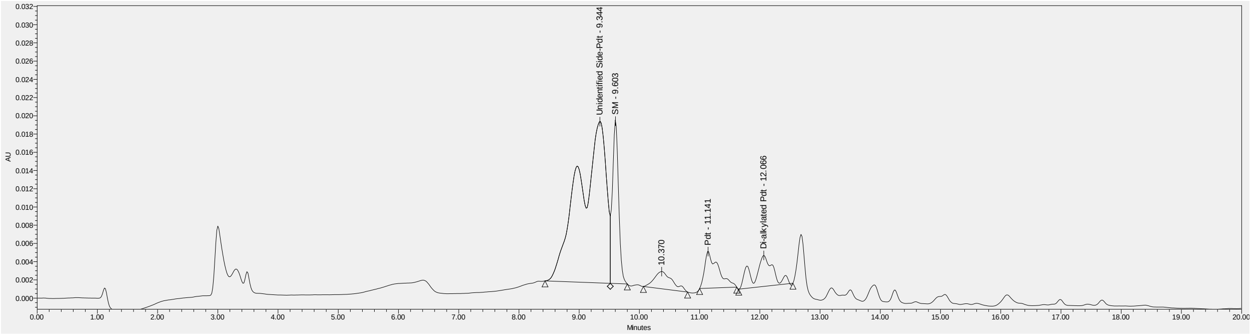

	Name	Retention Time	% Area
1	Unidentified Side-Pdt	9.344	64.48
2	SM	9.603	14.96
3		10.370	4.59
4	Pdt	11.141	7.31
5	Di-alkylated Pdt	12.066	8.66

Table 1, Entry 11

Zn metal, diiodide

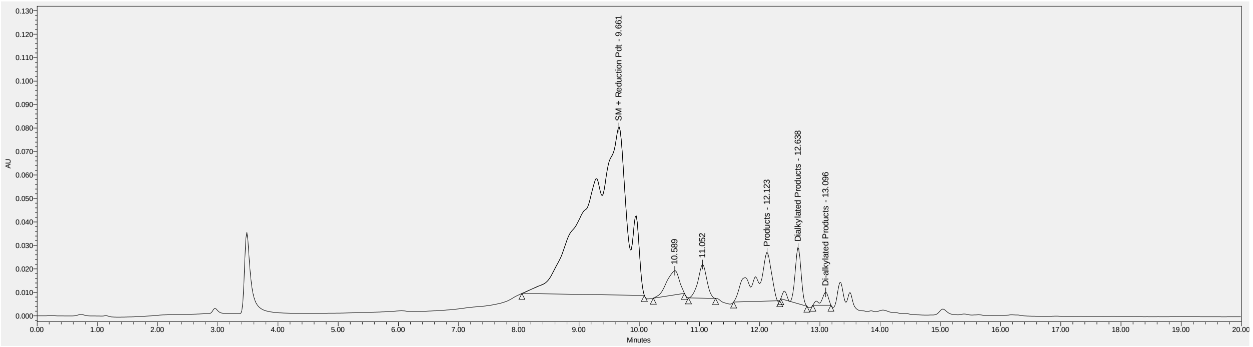

	Name	Retention Time	% Area
1	SM + Reduction Pdt	9.661	78.44
2		10.589	3.51
3		11.052	3.43
4	Products	12.123	9.34
5	Dialkylated Products	12.638	4.25
6	Di-alkylated Products	13.096	1.02

Table 1, Entry 20

+C|-C, undivided cell, bis-oxalate, NHPI, 5 mA

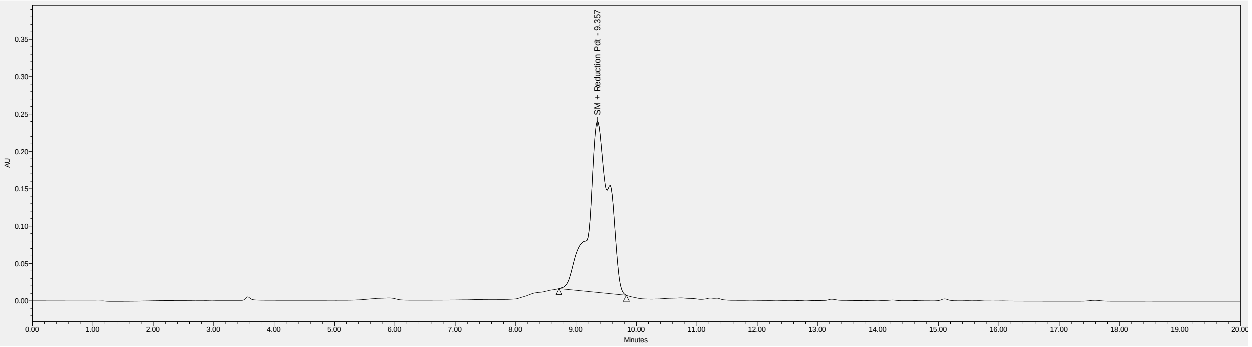

Peptide Cyclization

Reaction Optimization

All reactions were carried under the conditions indicated. All reactions were purged with N_2 (3 minutes), sealed with parafilm, and wrapped in aluminum foil for the duration of the reaction. Reactions were filtered and then analyzed by LC-MS.

Table S27. Screening of Aqueous Solvents

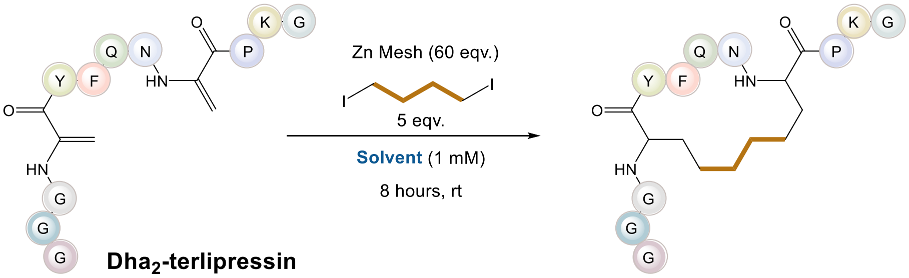

Dha_2-terlipressin

0.3 mg, 259 nmol

Solvent	% Conversion
Water	0 %
TFA buffer, pH 1.3	6 %
Cesium formate buffer, pH 3.51	3 %
Sodium acetate buffer, pH 5.14	0 %
Potassium phosphate buffer, pH 7.05	0 %
Sodium borate buffer, pH 10.22	15 %

Table S28. Screening of Zero-Valent Metals

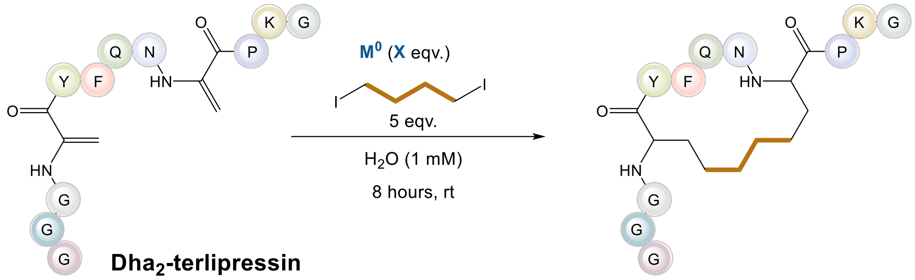

Dha$_2$-terlipressin

0.3 mg, 259 nmol

M^0	Equivalents	% Conversion
Zn	120	0 %
Zn	240	0 %
Zn	600	13 %
Cu	60	2 %
Cu + Zn	60 + 60	12 %
Cu + Zn	600 + 600	0 %
Sm	60	< 1 %
Sm + Zn	60 + 60	0 %

Table S29. Screening of Cosolvents

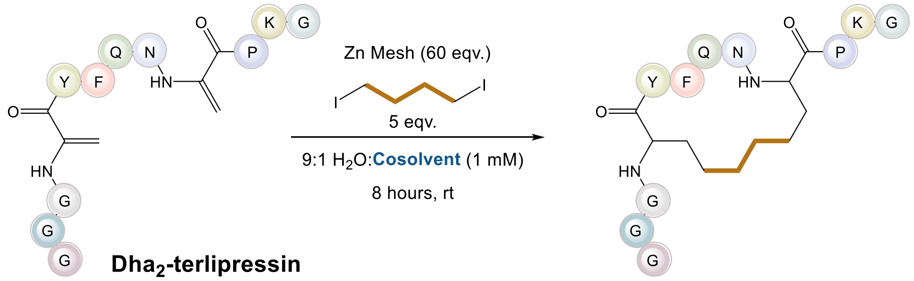

Dha$_2$-terlipressin

0.3 mg, 259 nmol

Cosolvent	% Conversion
DMSO	5 %
t-BuOH	0 %
DMF	0 %
acetone	0 %
MeCN	0 %
MeOAc	0 %
MeOH	12%
MeOH/sat. aq. NH_4Cl 1:9	14%

Table S30. Screening of Copper (II) and Copper (I) Species

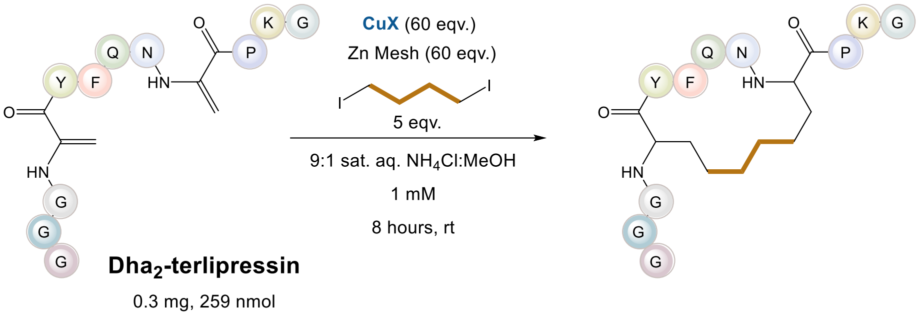

CuX	% Conversion
$Cu(OAc)_2$	21 %
$CuCO_3 \cdot Cu(OH)_2$	53 %
$CuBr_2$	0 %
Cu(2-ethylhexanoate)$_2$	35 %
Cu(2-thiophenecarboxylate)	0 %
CuF_2	0 %
$CuCl_2$	0 %
$CuSO_4$	0 %
$Cu(BF_4)_2$	0 %
$Cu(acac)_2$	trace
$Cu(TFA)_2$	0 %
$Cu(OTf)_2$	1 %

Table S31. Screening of Alcohol Cosolvents

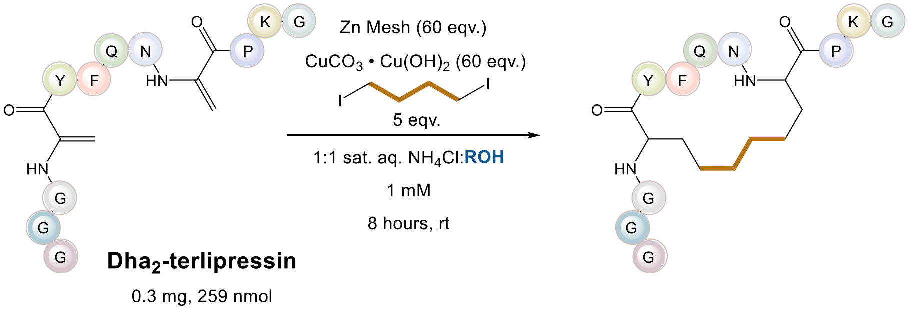

ROH	% Conversion
EtOH	0 %
i-PrOH	0 %
t-BuOH	0 %
2,2,2-trifluoroethanol	55 %
hexafluoroisopropanol	29 %
ethylene glycol	6 %

Table S32. Screening of Zinc Sources

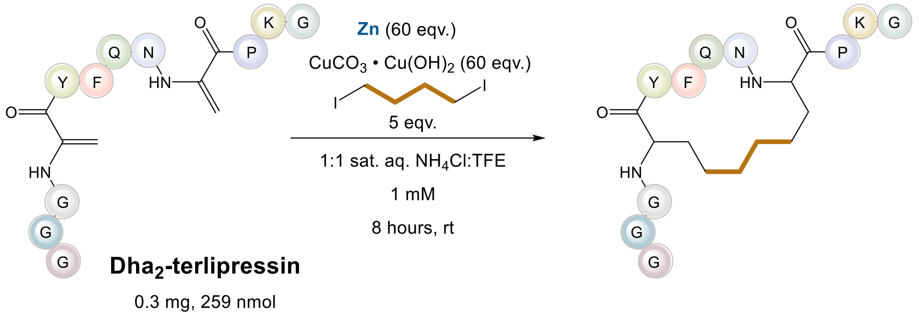

Zn	% Conversion
Zinc mesh	43 %
Zinc dust	39 %
Zinc powder	<10 %

Table S33. Optimization of Ammonium Salt

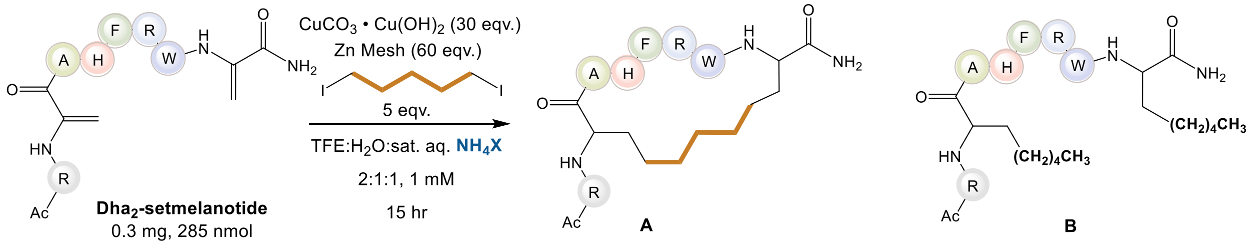

NH_4X	% SM Remaining	% Conversion A	% Conversion B
NH_4Cl	39 %	16 %	16 %
NH_4HCO_3	17 %	3 %	6 %
NH_4PF_6	66 %	15 %	9 %
$(NH_4)_2CO_3$	25 %	0 %	0 %
$(NH_4)_2SO_4$	10 %	9 %	26 %
NH_4OAc	27 %	6 %	4 %
$(NH_4)_2HPO_4$	9 %	20 %	45 %
NH_4BF_4	49 %	23 %	21 %
NH_4HCO_2	7 %	21 %	12 %
NH_4F	71 %	< 1 %	< 1 %
NH_4OH	31 %	< 1 %	< 1 %
$NH_4SO_3NH_2$	13 %	15 %	23 %
NH_4NO_3	16 %	0 %	0 %
$(NH_4)_2SO_3$	7 %	33 %	37 %
NH_4SCN	17 %	23 %	44 %

Table S34. Screening of Various Additives

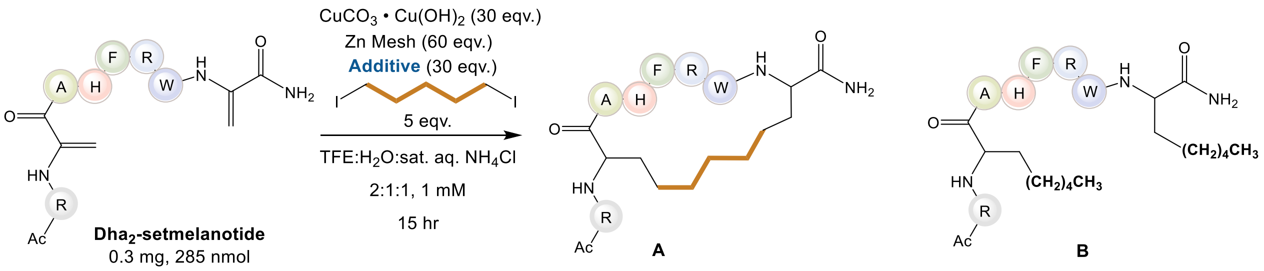

Additive	% SM Remaining	% Conversion A	% Conversion B
$HSi[Si(CH_3)_3]_3$	63 %	17 %	7 %
$HSn(n\text{-}Bu)_3$	72 %	10 %	8 %
$NaBH_3CN$	47 %	10 %	29 %
$Na_2S_2O_3$	100 %	0 %	0 %
In^0	0 %	5 %	7 %
$NaHCO_2$	15 %	0 %	0 %

Table S35. Screening of Hydrosilanes

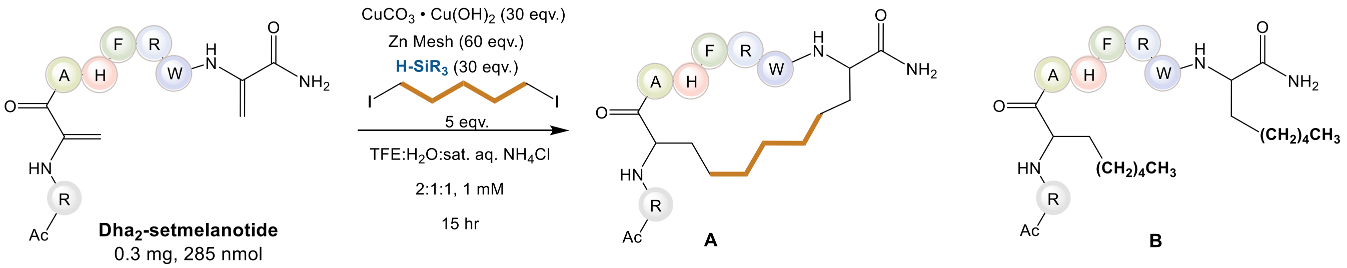

$H\text{-}SiR_3$	% SM Remaining	% Conversion A	% Conversion B
$HSiMe_2Ph$	9 %	43 %	13 %
$HSiPh_2Me$	0 %	33 %	49 %
$HSiEt_3$	6 %	62 %	32 %
$HSi(OEt)_3$	0 %	41 %	60 %
$HSi(n\text{-}Bu)_3$	5 %	70 %	25 %
$HSi(i\text{-}Pr)_3$	6 %	69 %	10 %
$HSiMe_2(t\text{-}Bu)$	64 %	26 %	10 %
$HSiMe_2(i\text{-}Pr)$	23 %	34 %	15 %

Table S36. Screening of Metal Equivalents

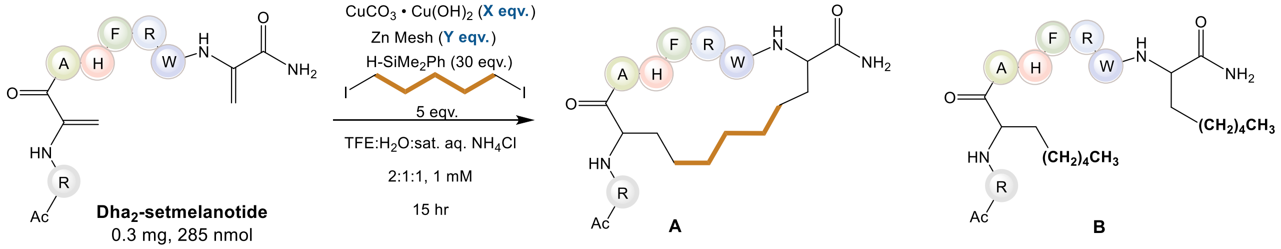

Zn Mesh Equivalents	$CuCO_3 \cdot Cu(OH)_2$ Equivalents	% SM Remaining	% Conversion A	% Conversion B
60	20	64 %	25 %	12 %
60	10	60 %	34 %	6 %
60	5	79 %	12 %	9 %
60	1	80 %	13 %	8 %
40	20	91 %	7%	2 %
20	10	91 %	6 %	3 %
10	5	100 %	0 %	0 %
2	1	100 %	0 %	0 %

Representative LC Traces for Optimization

Table 1, Entry 12

Zn metal, NH_4Cl (aq.), MeOH

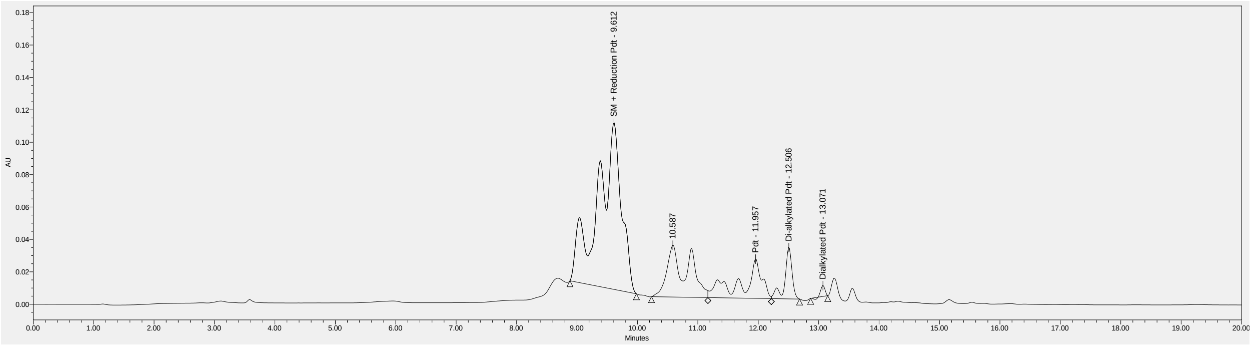

	Name	Retention Time	% Area
1	SM + Reduction Pdt	9.612	63.15
2		10.587	17.30
3	Pdt	11.957	12.67
4	Di-alkylated Pdt	12.506	6.02
5	Dialkylated Pdt	13.071	0.86

Table 1, Entry 14

Zn metal, NH_4Cl (aq.), $CuCO_3{\bullet}Cu(OH)_2$, TFE (1:1)

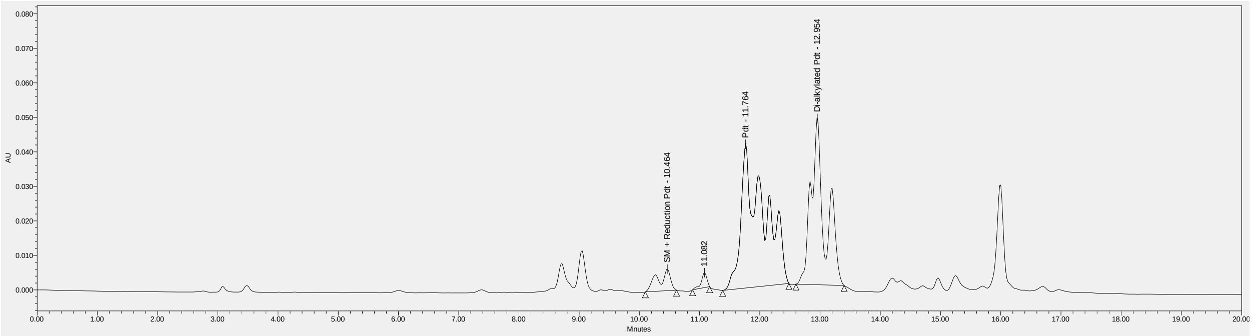

	Name	Retention Time	% Area
1	SM + Reduction Pdt	10.464	4.51
2		11.082	1.39
3	Pdt	11.764	54.92
4	Di-alkylated Pdt	12.954	39.18

Table 1, Entry 15

Zn metal, NH_4Cl (aq.), $CuCO_3{\bullet}Cu(OH)_2$, $HSiMe_2Ph$, TFE (1:1)

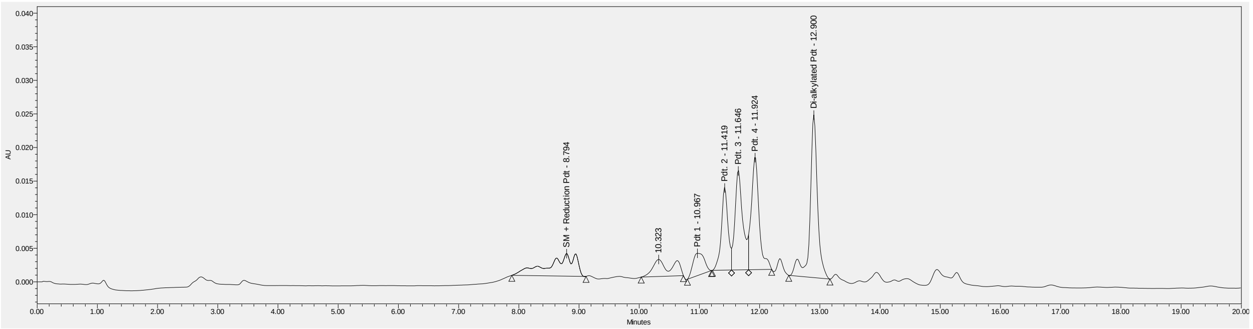

	Name	Retention Time	% Area
1	SM + Reduction Pdt	8.794	13.39
2		10.323	6.76
3	Pdt 1	10.967	5.43
4	Pdt. 2	11.419	11.89
5	Pdt. 3	11.646	17.23
6	Pdt. 4	11.924	18.80
7	Di-alkylated Pdt	12.900	26.50

General Procedure for Peptide Cyclization

In a 1-dram vial charged with a magnetic stir bar, setmelanotide-Dha_2 (1.0 mg, 951 nmol) was dissolved in H_2O (238 µL), saturated aqueous NH_4Cl (238 µL), and 2,2,2-trifluoroethanol (475 µL) to a final concentration of 1 mM. Next were added $CuCO_3 \cdot Cu(OH)_2$ (28.5 µmol, 6.3 mg), diiodide (4.76 µmol), and $HSiMe_2Ph$ (14.3 µmol, 2.19 µL). Zinc powder (325 mesh, 57.1 µmol, 3.7 mg) was added last and the resulting suspension was purged with N_2 for 3 minutes, sealed with parafilm, wrapped in aluminum foil, and stirred for 15 hours at ambient temperature. The resulting suspension was filtered and analyzed by LC-MS. Large scale reactions were concentrated and purified by flash chromatography (C18; H_2O/0.1% TFA to 95% EtOH/0.1% TFA). Conversions are reported on 1.0 mg scales as the percent area of cyclic products to percent area of all peptide-derived products and starting material. Overall conversion was determined by the total area of all peptide-derived products against the area of starting material at 280 nm. Isolated yields are determined via a calibration curve (pg. **S98**) on 5.0 or 10.0 mg scales (as indicated) at 280 nm. Purity was determined was total percent area of cyclic products against impurities at 280 nm. Note that retention times vary between crude reactions and purified peptides due to high concentrations of metals and salts. Whereas diastereomers typically separated during flash chromatography, coalescence of diastereomers is often observed in the purified products. No attempt was made to isolate individual diastereomers. For MS/MS data, the characteristic fragment observed is b_1/y_7 (i.e., between the amide bond of Arg_1 and AA_2 to yield the macrocyclic portion).

Carbocyclic Setmelanotide Analogue Characterization

C_1-setmelanotide

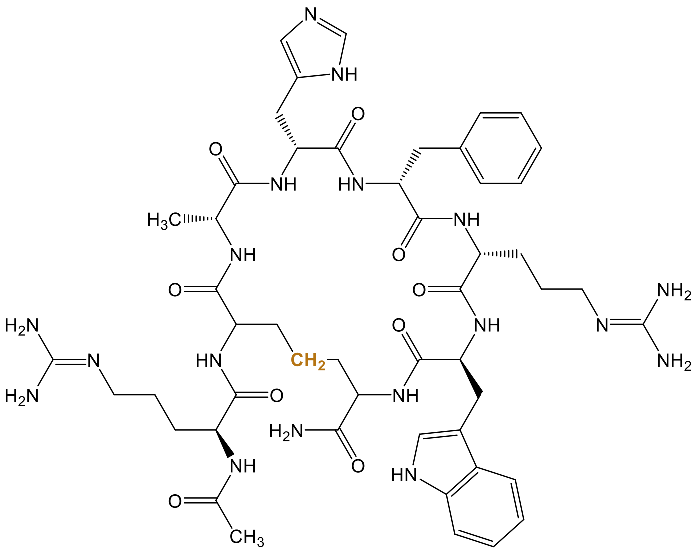

Crude Spectrum

Conversion 21.7%

d.r. 1.8 : 1.0 : 3.1

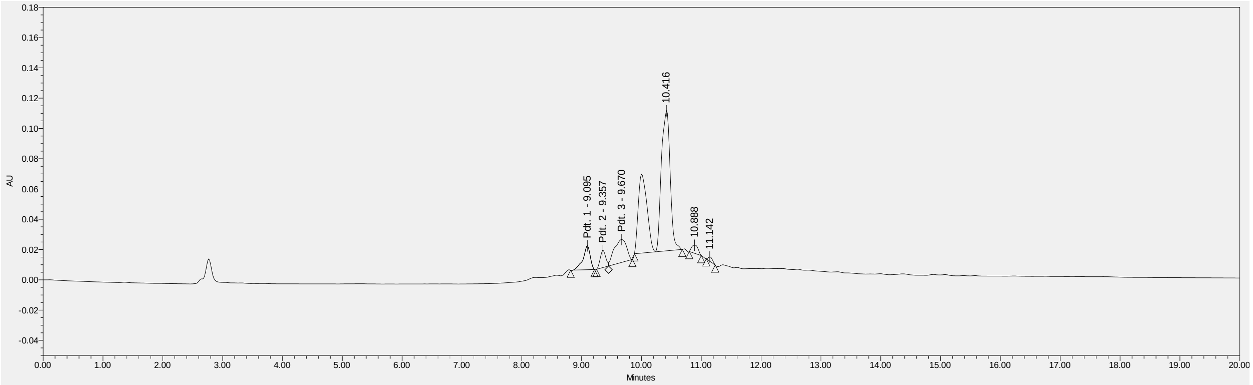

	Name	Retention Time	Area	% Area
1	Pdt. 1	9.095	132105	6.79
2	Pdt. 2	9.357	71533	3.68
3	Pdt. 3	9.670	219323	11.27
4		10.416	1467806	75.42
5		10.888	40474	2.08
6		11.142	15022	0.77

C4-setmelanotide

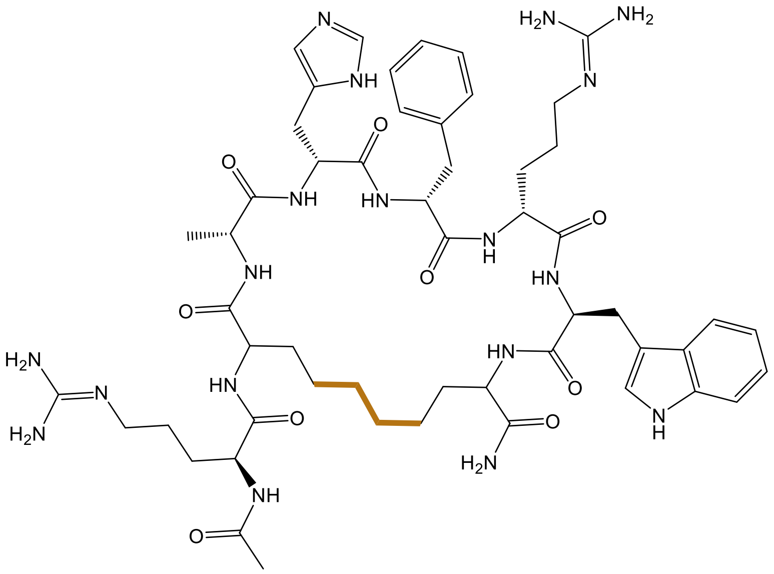

Crude Spectrum:

Conversion 23.8%

d.r. 1.8 : 1.0 : 1.5 : 2.3

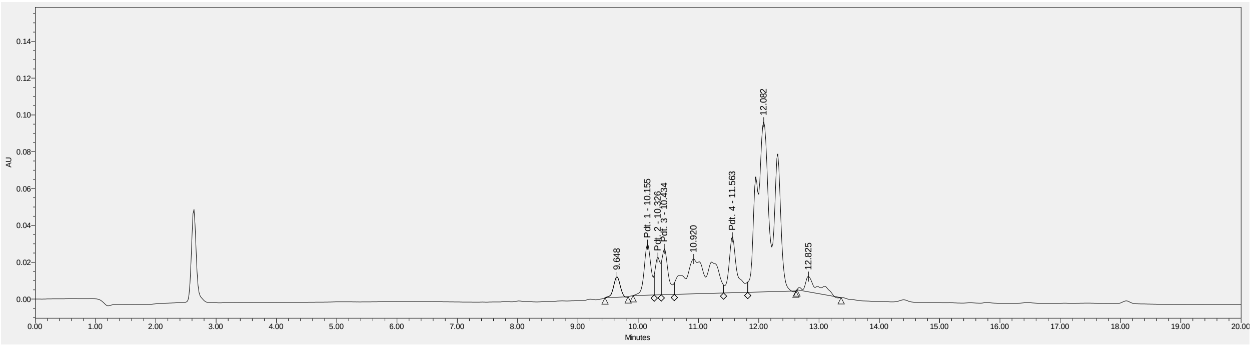

	Name	Retention Time	% Area
1		9.648	2.50
2	Pdt. 1	10.155	6.48
3	Pdt. 2	10.326	3.59
4	Pdt. 3	10.434	5.31
5		10.920	18.10
6	Pdt. 4	11.563	8.43
7		12.082	51.74
8		12.825	3.85

Isolated Products

Yield 0.17 mg from 5.0 mg, Purity 95.3%

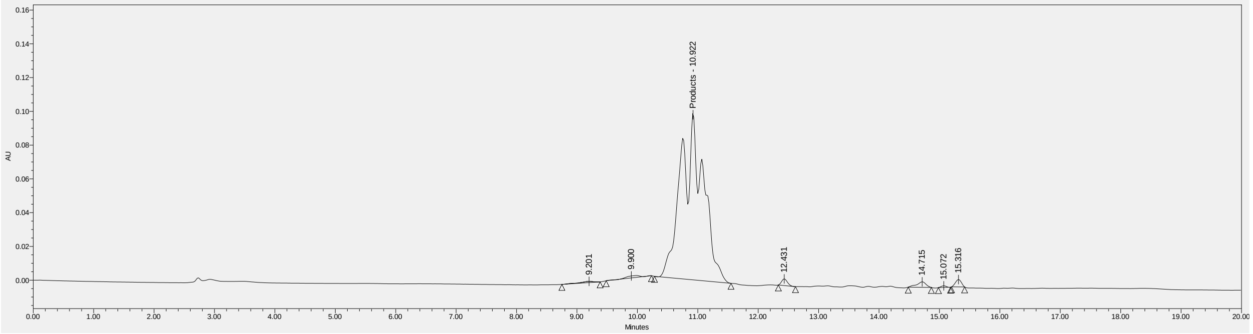

	Name	Retention Time	Area	% Area
1		9.201	11261	0.42
2		9.900	18479	0.69
3	Products	10.922	2539966	95.27
4		12.431	26227	0.98
5		14.715	34866	1.31
6		15.072	5625	0.21
7		15.316	29672	1.11

HRMS (ESI+)

Calculated for $C_{53}H_{77}N_{18}O_9$ $(M+H)^+$: 1109.6116

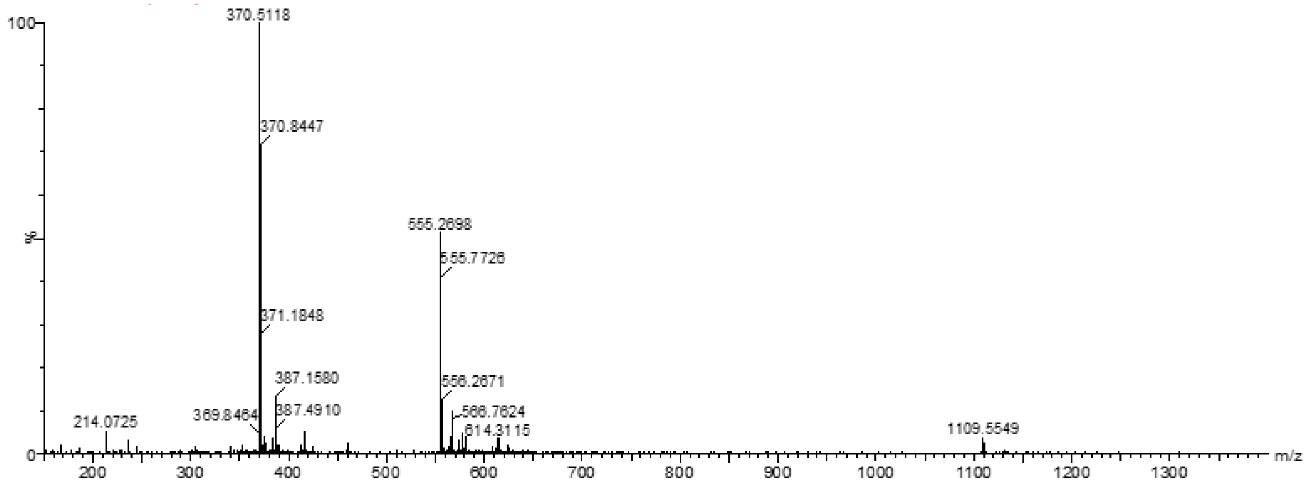

MS/MS (ESI+)

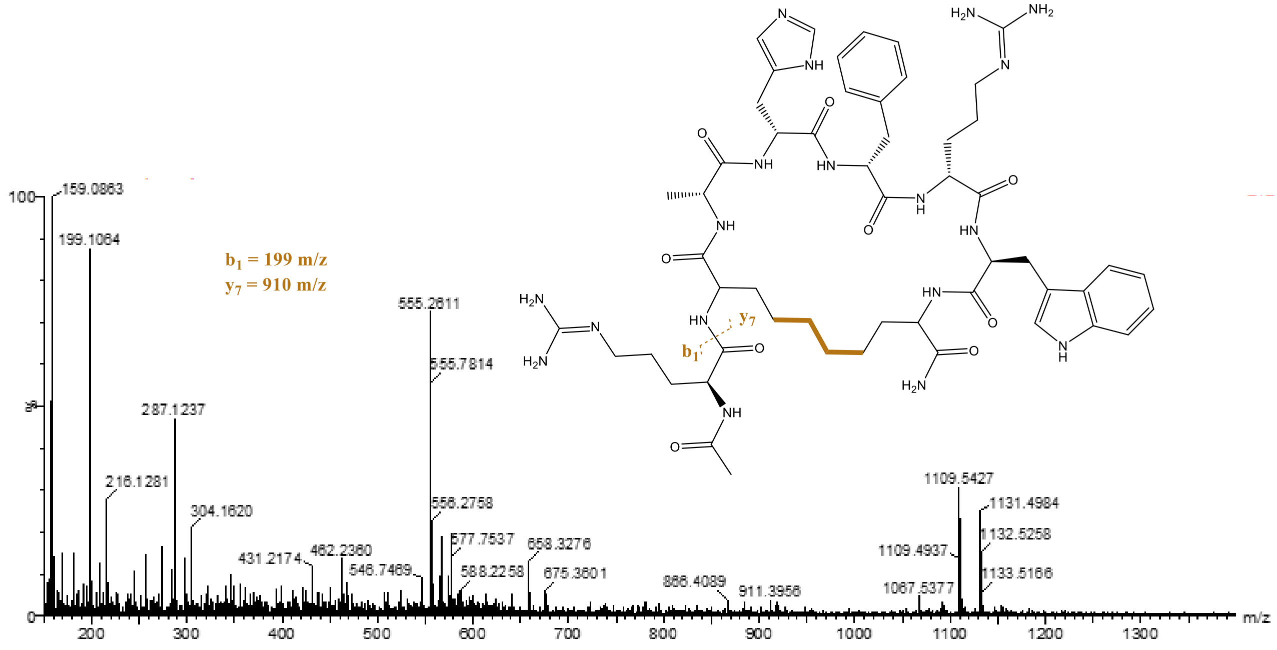

b1 = 199 m/z
y7 = 910 m/z
159.0863
199.1064
216.1281
287.1237
304.1620
431.2174
462.2360
546.7469
555.2611
555.7814
556.2758
577.7537
588.2258
658.3276
675.3601
866.4089
911.3956
1067.5377
1109.4937
1109.5427
1131.4984
1132.5258
1133.5166
m/z

C$_5$-setmelanotide

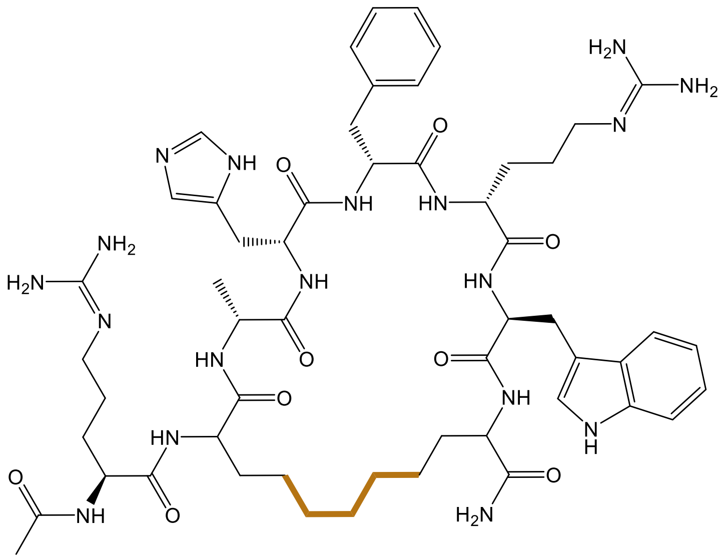

Crude Spectrum

Conversion 31.9%

d.r. 1.4 : 1.9 : 1.0 : 2.1

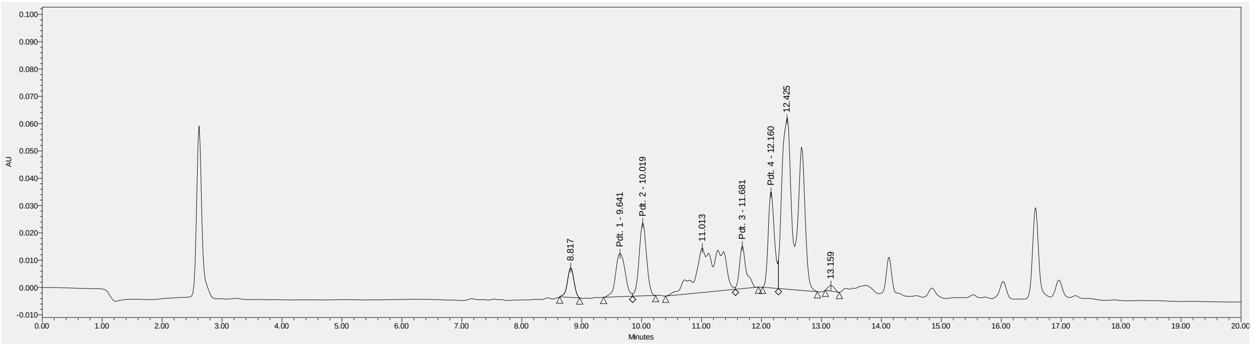

	Name	Retention Time	% Area
1		8.817	3.36
2	Pdt. 1	9.641	7.09
3	Pdt. 2	10.019	9.29
4		11.013	20.23
5	Pdt. 3	11.681	4.94
6	Pdt. 4	12.160	10.56
7		12.425	43.93
8		13.159	0.60

Isolated Products

Yield 0.09 mg from 5.0 mg, Purity 90.4%

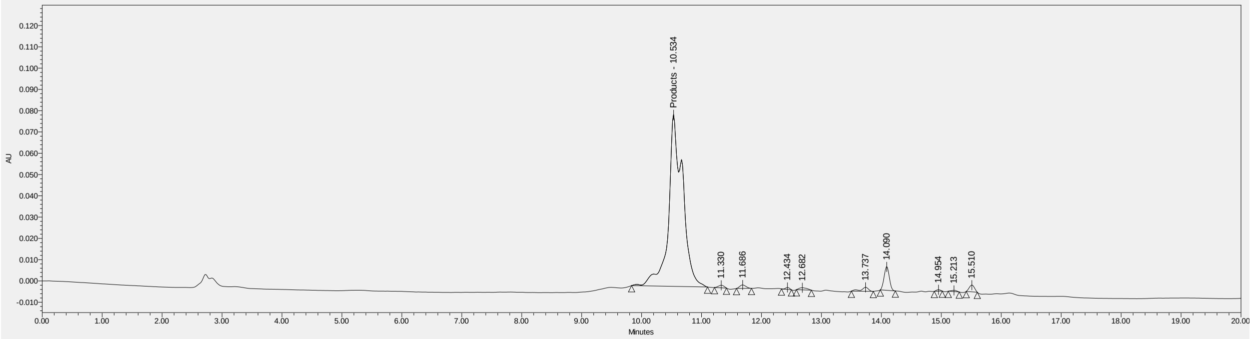

	Name	Retention Time	Area	% Area
1	Products	10.534	1337295	90.38
2		11.330	7843	0.53
3		11.686	11689	0.79
4		12.434	4282	0.29
5		12.682	9267	0.63
6		13.737	16017	1.08
7		14.090	66804	4.51
8		14.954	3313	0.22
9		15.213	3828	0.26
10		15.510	19277	1.30

HRMS (ESI+)

Calculated for $C_{54}H_{79}N_{18}O_9$ $(M+H)^+$: 1123.6272

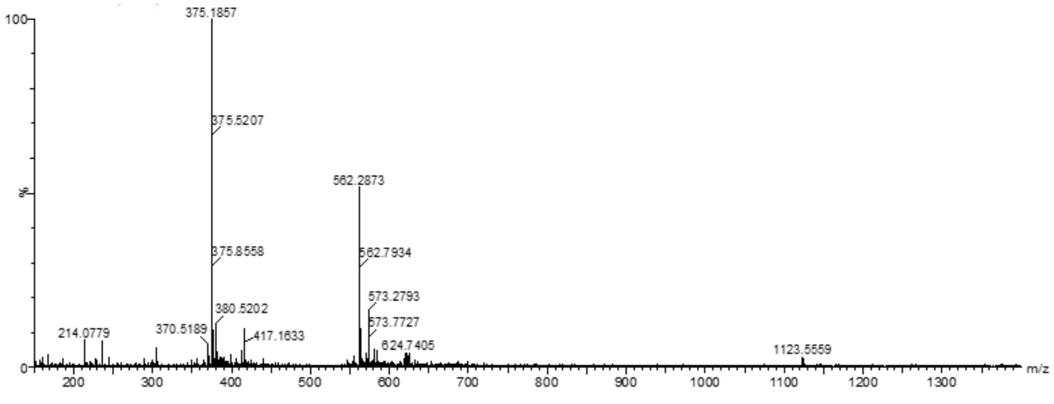

MS/MS (ESI+)

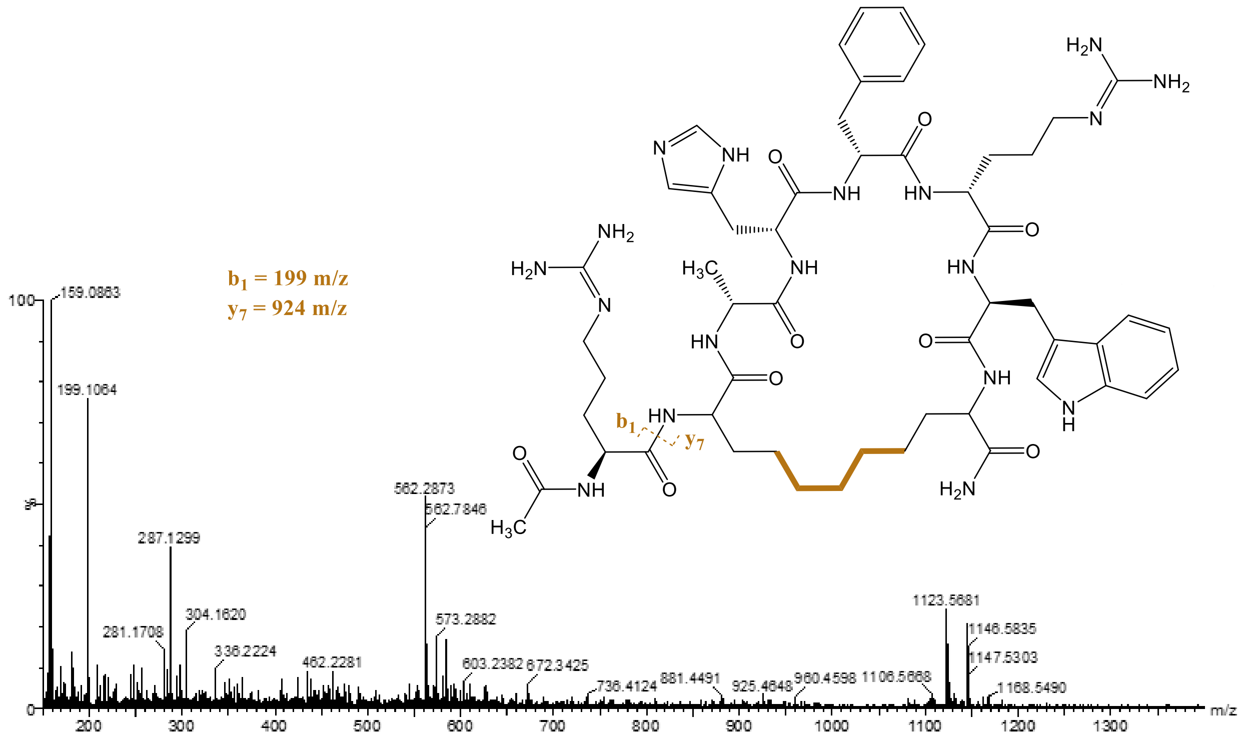

b1 = 199 m/z
y7 = 924 m/z
159.0863
199.1064
287.1299
281.1708
304.1620
336.2224
462.2281
562.2873
562.7846
573.2882
603.2382
672.3425
736.4124
881.4491
925.4648
960.4598
1106.5668
1123.5681
1146.5835
1147.5303
1168.5490
m/z

C_6-setmelanotide

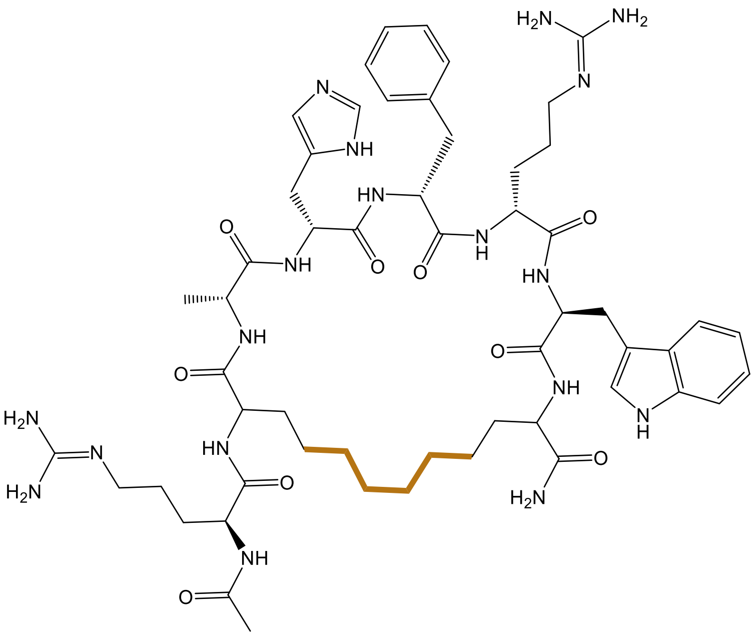

Crude Spectrum

Conversion 38.1%

d.r. 1.1 : 1.5 : 2.3 : 1.0

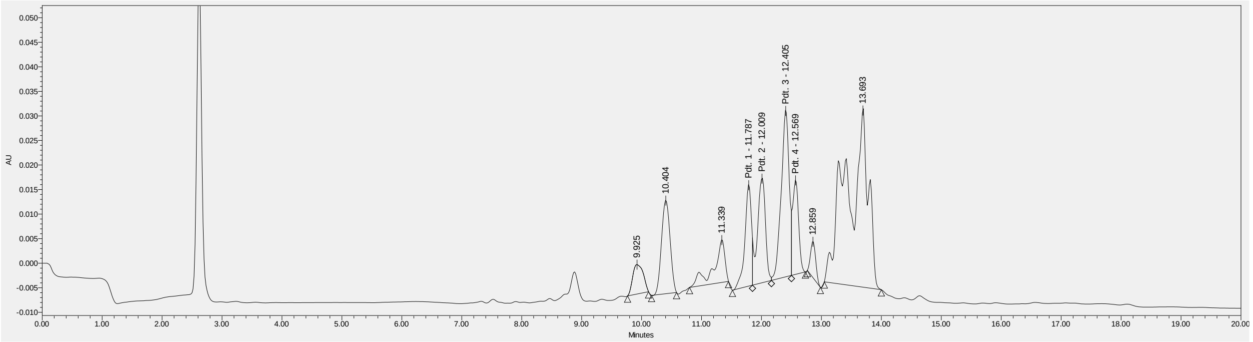

	Name	Retention Time	% Area
1		9.925	3.48
2		10.404	9.04
3		11.339	5.35
4	Pdt. 1	11.787	7.25
5	Pdt. 2	12.009	9.60
6	Pdt. 3	12.405	14.82
7	Pdt. 4	12.569	6.45
8		12.859	2.11
9		13.693	41.90

Isolated Products

Yield 0.16 mg from 5.0 mg, Purity 90.3%

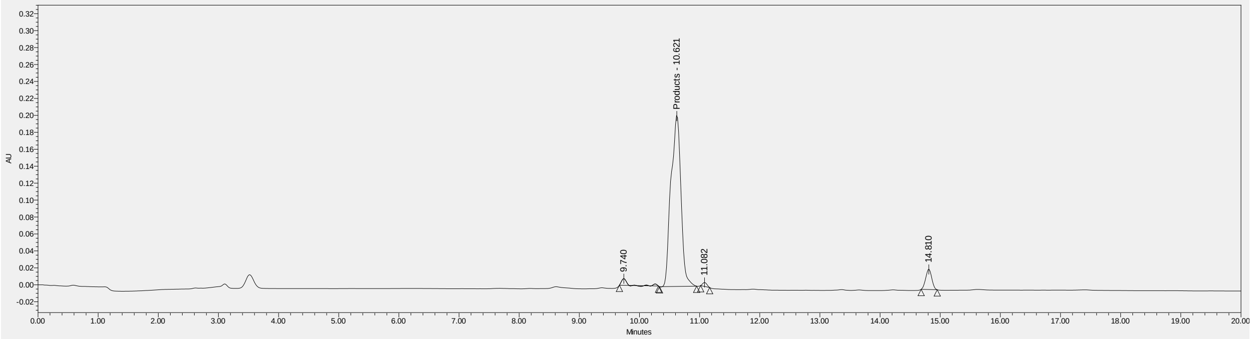

	Name	Retention Time	Area	% Area
1		9.740	61620	2.38
2	Products	10.621	2334814	90.29
3		11.082	22965	0.89
4		14.810	166587	6.44

HRMS (ESI+)

Calculated for $C_{55}H_{81}N_{18}O_{9}$ $(M+H)^{+}$: 1137.6429

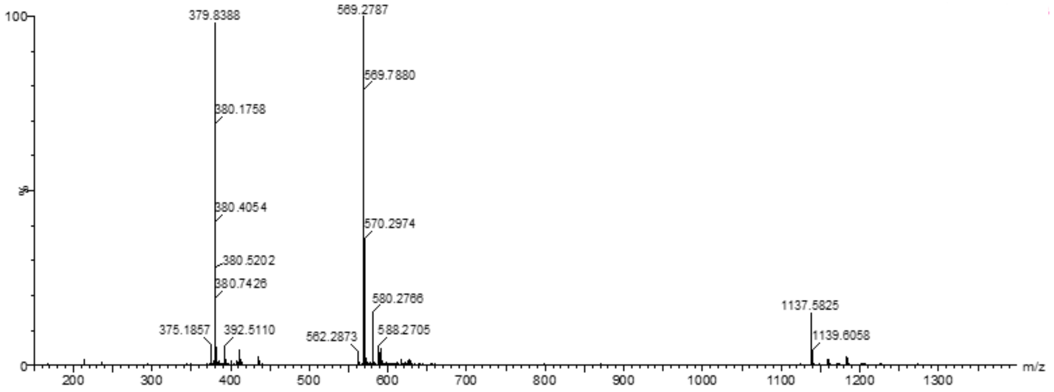

MS/MS (ESI+)

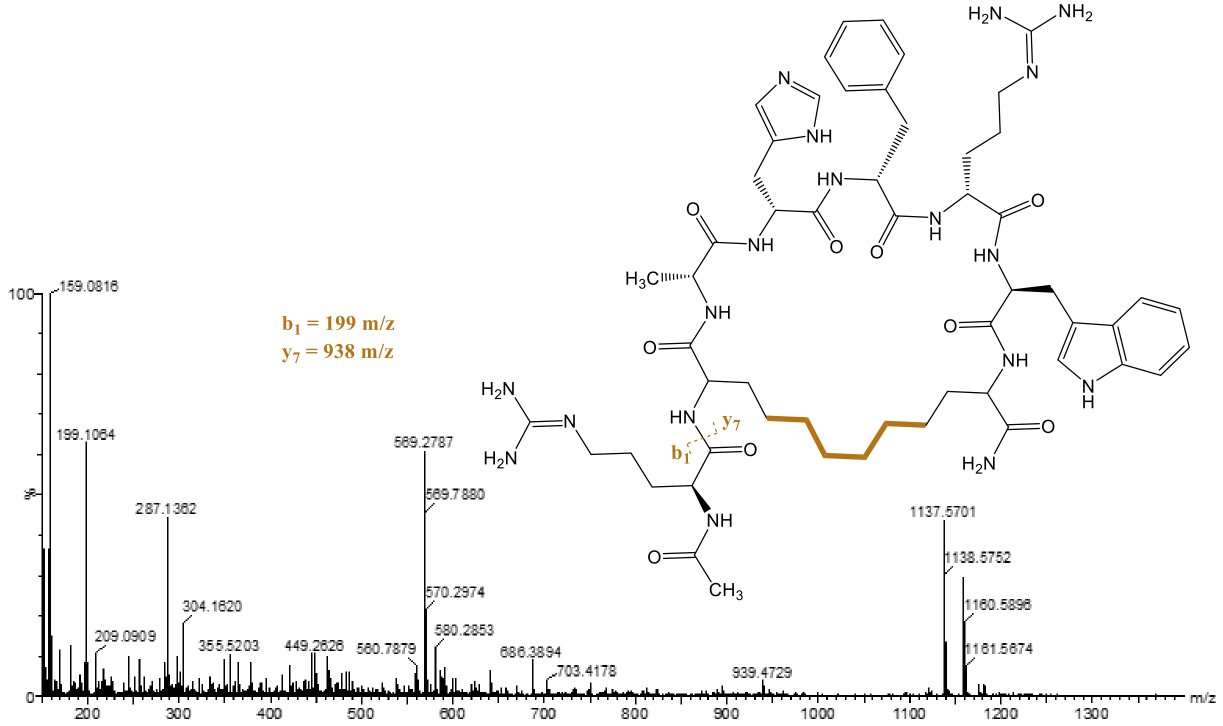

b1 = 199 m/z
y7 = 938 m/z
159.0816
199.1064
287.1362
304.1620
209.0909
355.5203
449.2626
560.7879
569.2787
569.7880
570.2974
580.2853
686.3894
703.4178
939.4729
1137.5701
1138.5752
1160.5896
1161.5674
m/z

C$_7$-setmelanotide

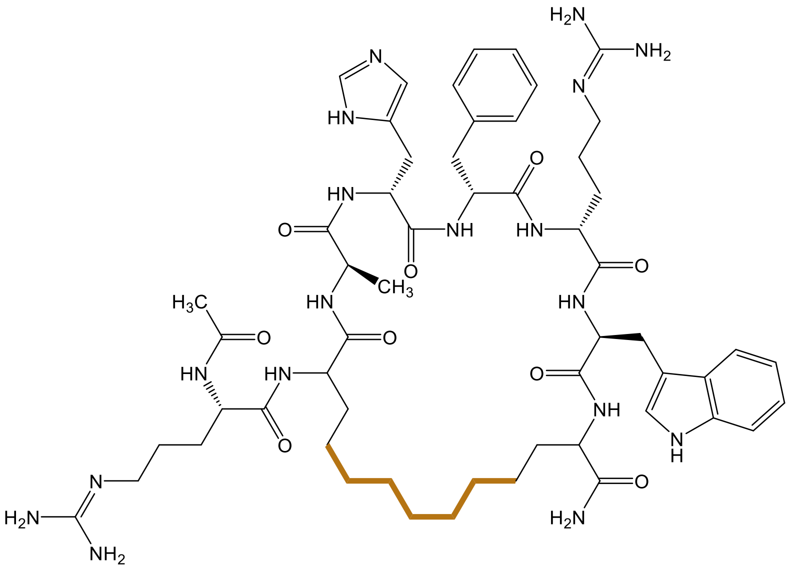

Crude Spectrum

Conversion 34.2%

d.r. 1.1 : 1.0 : 4.7 : 2.4

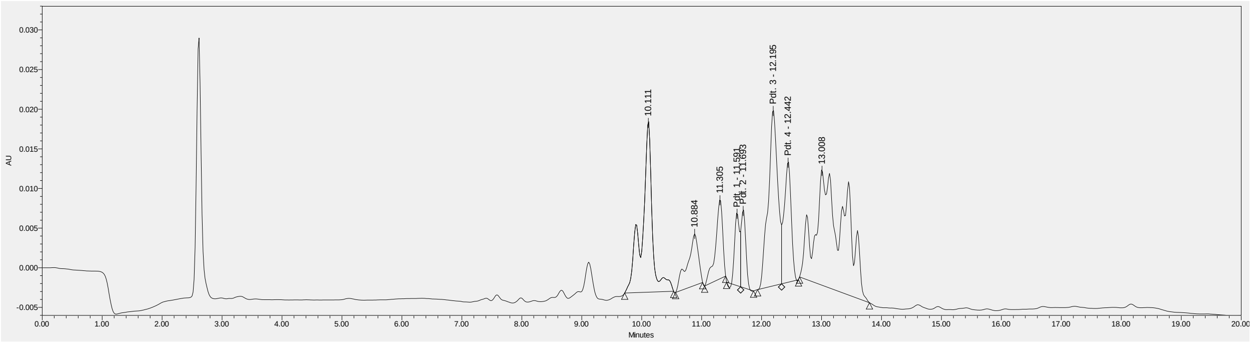

	Name	Retention Time	% Area
1		10.111	18.37
2		10.884	5.89
3		11.305	5.85
4	Pdt. 1	11.591	3.94
5	Pdt. 2	11.693	3.71
6	Pdt. 3	12.195	17.56
7	Pdt. 4	12.442	8.99
8		13.008	35.69

Isolated Products

Yield 0.25 mg from 5.0 mg, Purity 92.0%

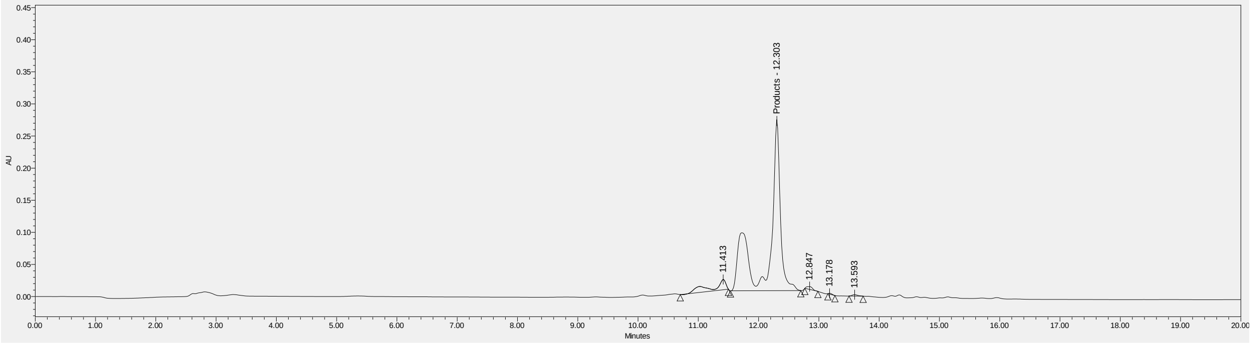

	Name	Retention Time	Area	% Area
1		11.413	256829	6.78
2	Products	12.303	3485440	91.95
3		12.847	28646	0.76
4		13.178	5388	0.14
5		13.593	14255	0.38

HRMS (ESI+)

Calculated for $C_{56}H_{83}N_{18}O_9$ $(M+H)^+$: 1151.6585

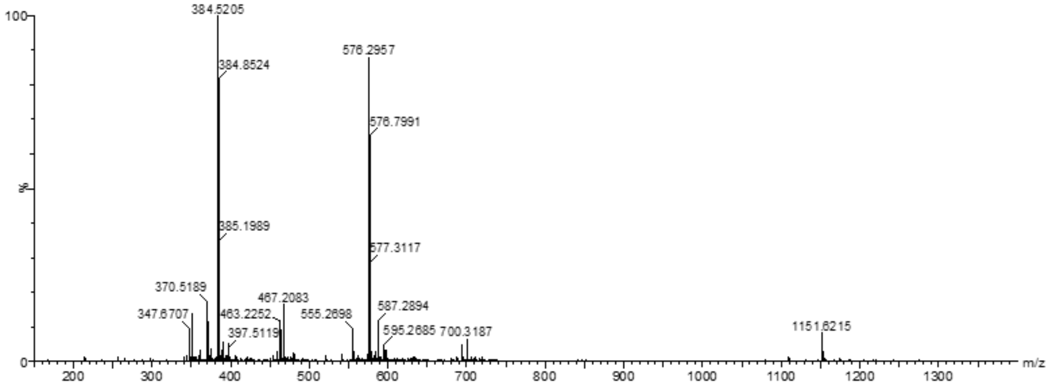

MS/MS (ESI+)

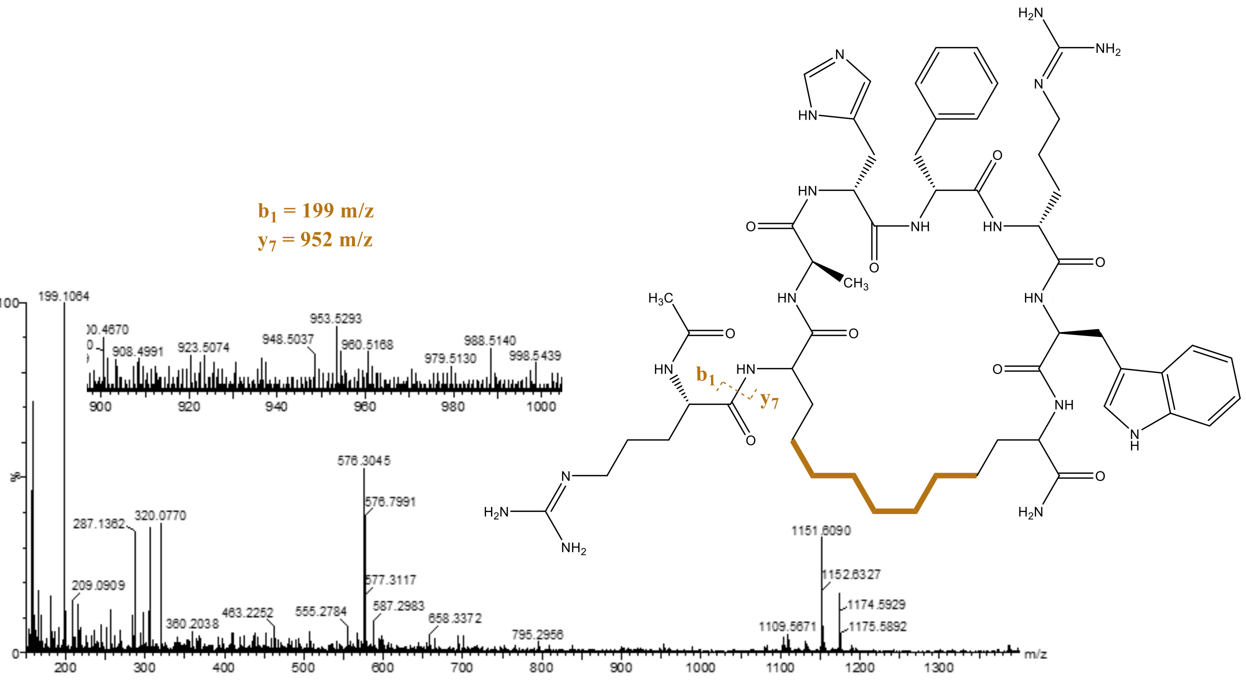

b1 = 199 m/z
y7 = 952 m/z
199.1064
953.5293
948.5037
960.5168
988.5140
979.5130
998.5439
908.4991
923.5074
576.3045
576.7991
577.3117
587.2983
287.1362
320.0770
209.0909
360.2038
463.2252
555.2784
658.3372
795.2956
1151.6090
1152.6327
1174.5929
1175.5892
1109.5671
m/z

C$_8$-setmelanotide

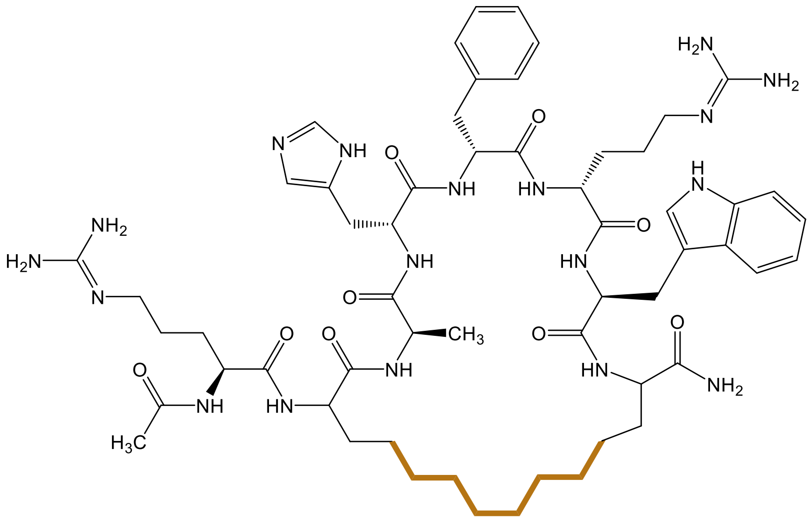

Crude Spectrum

Conversion 35.7%

d.r. 1.0 : 2.2 : 1.9 : 3.4

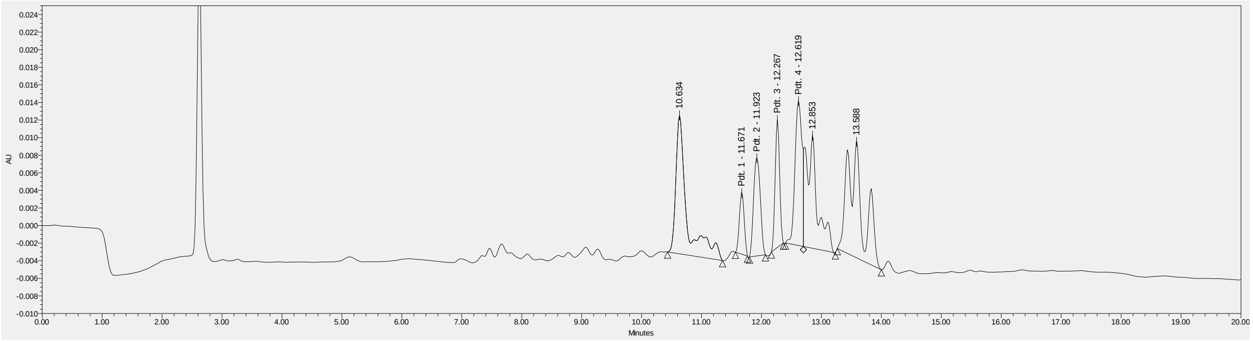

	Name	Retention Time	% Area
1		10.634	21.33
2	Pdt. 1	11.671	4.17
3	Pdt. 2	11.923	9.27
4	Pdt. 3	12.267	7.98
5	Pdt. 4	12.619	14.25
6		12.853	18.19
7		13.588	24.80

Isolated Products

Yield 0.28 mg from 5.0 mg, Purity 94.3%

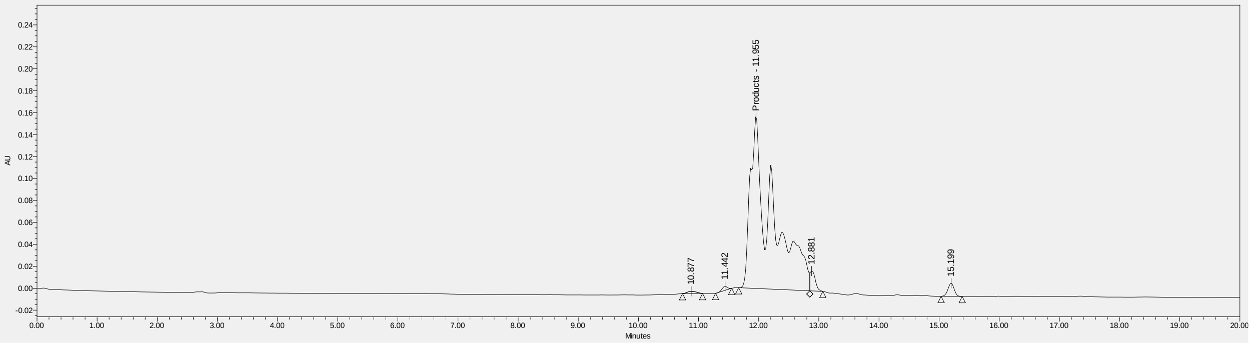

	Name	Retention Time	Area	% Area
1		10.877	21215	0.51
2		11.442	22349	0.54
3	Products	11.955	3921444	94.28
4		12.881	107894	2.59
5		15.199	86318	2.08

HRMS (ESI+)

Calculated for $C_{57}H_{85}N_{18}O_9$ $(M+H)^+$: 1165.6742

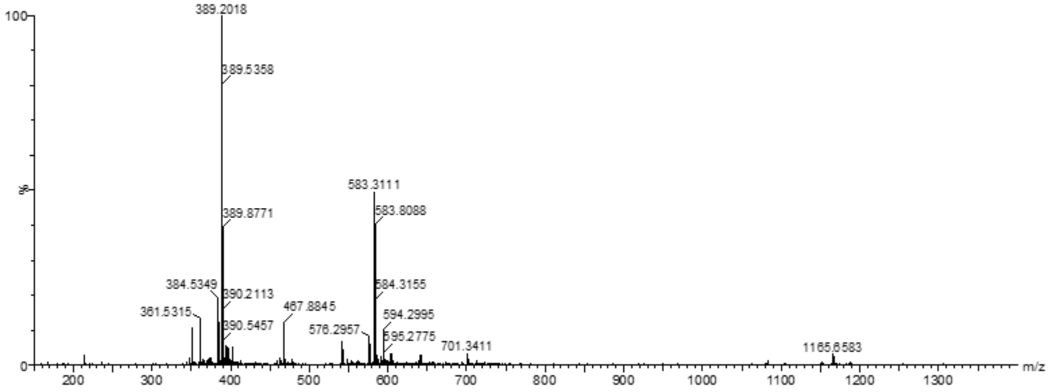

MS/MS (ESI+)

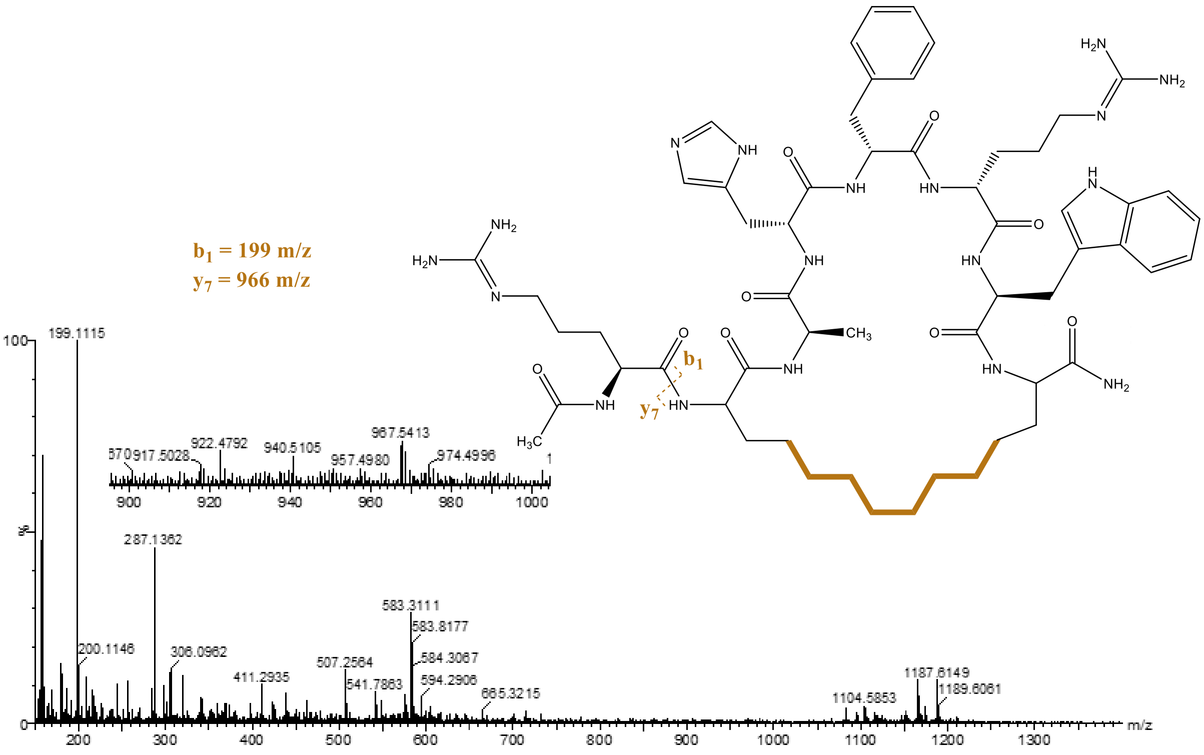

b1 = 199 m/z
y7 = 966 m/z
199.1115
287.1362
583.3111
583.8177
584.3067
594.2906
200.1146
306.0962
411.2935
507.2564
541.7863
665.3215
1104.5853
1187.6149
1189.6061
917.5028
922.4792
940.5105
957.4980
967.5413
974.4996
m/z
%

Cy-setmelanotide

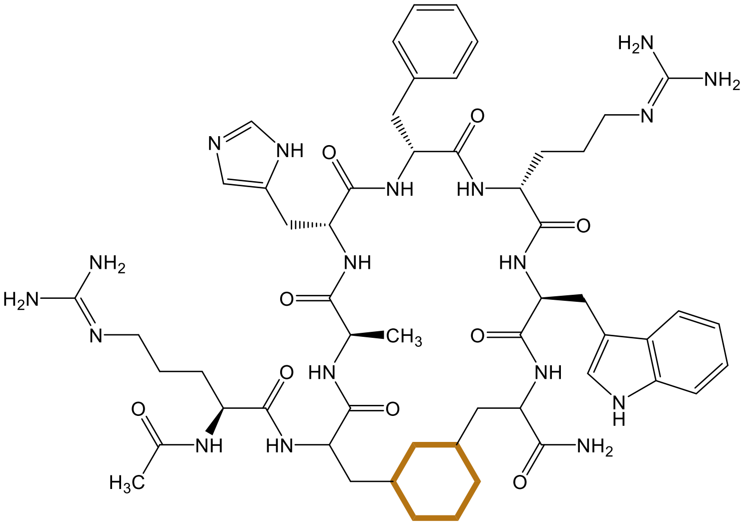

Crude Spectrum

Conversion 35.2%

d.r. 1.0 : 5.7 : 4.0 : 4.0 : 3.3

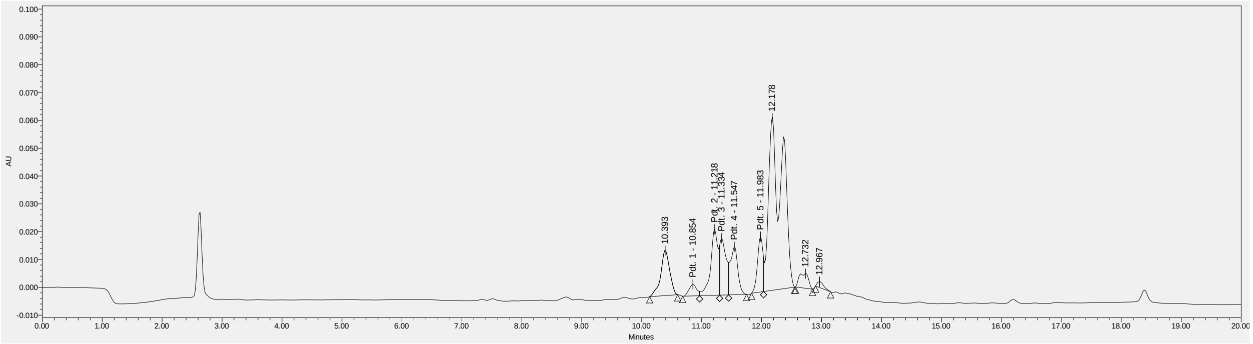

	Name	Retention Time	% Area
1		10.393	9.00
2	Pdt. 1	10.854	1.96
3	Pdt. 2	11.218	11.11
4	Pdt. 3	11.334	7.79
5	Pdt. 4	11.547	7.93
6	Pdt. 5	11.983	6.38
7		12.178	51.99
8		12.732	2.99
9		12.967	0.84

Isolated Products

Yield 0.33 mg from 5.0 mg, Purity 92.0%

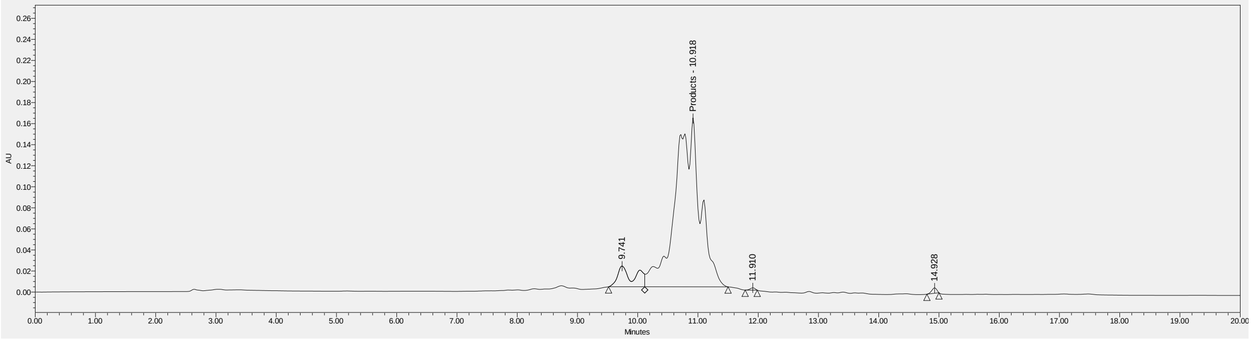

	Name	Retention Time	Area	% Area
1		9.741	368938	7.24
2	Products	10.918	4689123	91.96
3		11.910	11978	0.23
4		14.928	29255	0.57

HRMS (ESI+)

Calculated for $C_{55}H_{79}N_{18}O_9$ $(M+H)^+$: 1135.6272

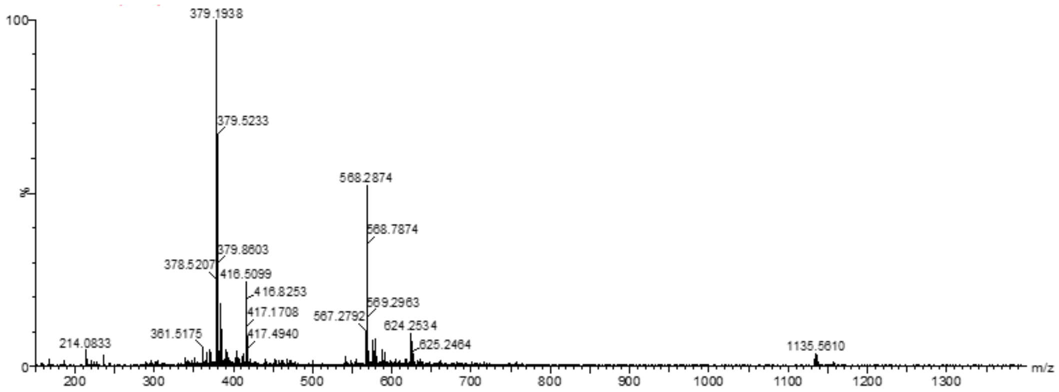

MS/MS (ESI+)

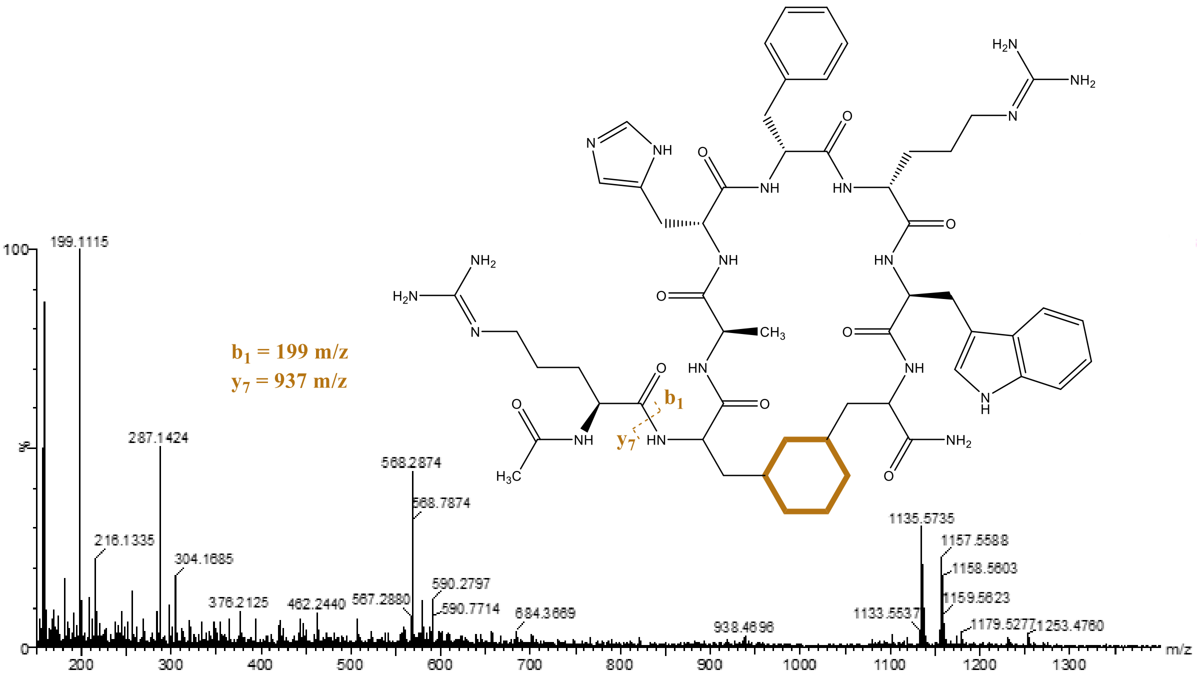

b1 = 199 m/z
y7 = 937 m/z
199.1115
287.1424
216.1335
304.1685
376.2125
462.2440
567.2880
568.2874
568.7874
590.2797
590.7714
684.3669
938.4696
1133.5537
1135.5735
1157.5588
1158.5603
1159.5623
1179.5277
1253.4760
m/z
%

PEG-setmelanotide

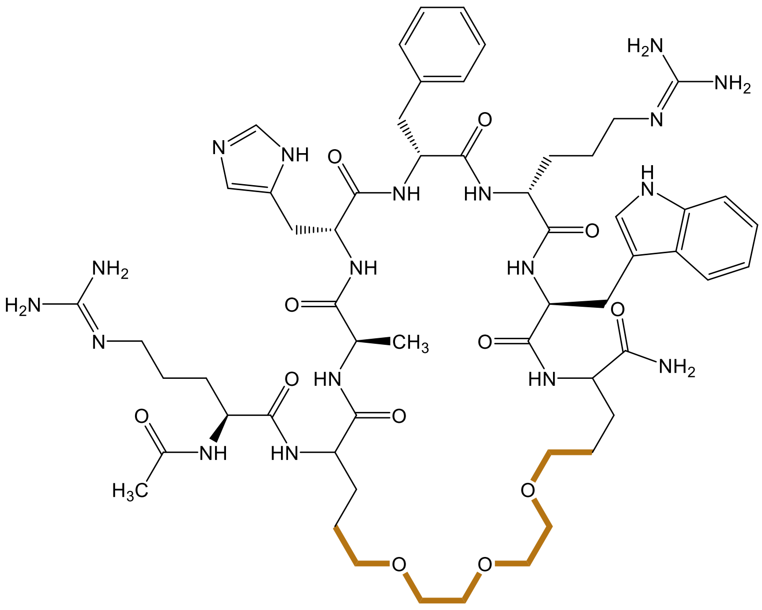

Crude Spectrum

Conversion 38.3%

d.r. 2.0 : 3.5 : 1.0

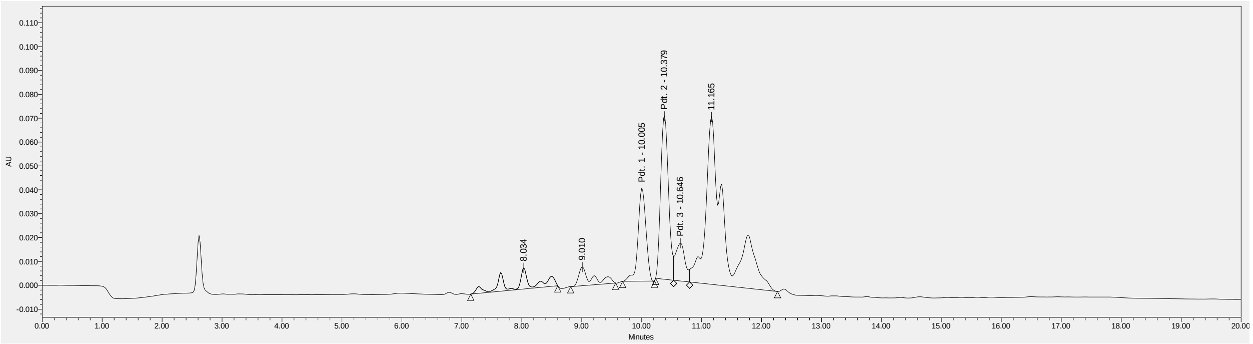

	Name	Retention Time	% Area
1		8.034	6.22
2		9.010	3.91
3	Pdt. 1	10.005	11.89
4	Pdt. 2	10.379	20.55
5	Pdt. 3	10.646	5.84
6		11.165	51.58

Isolated Products

Yield 0.66 mg from 10.0 mg, Purity 95.9%

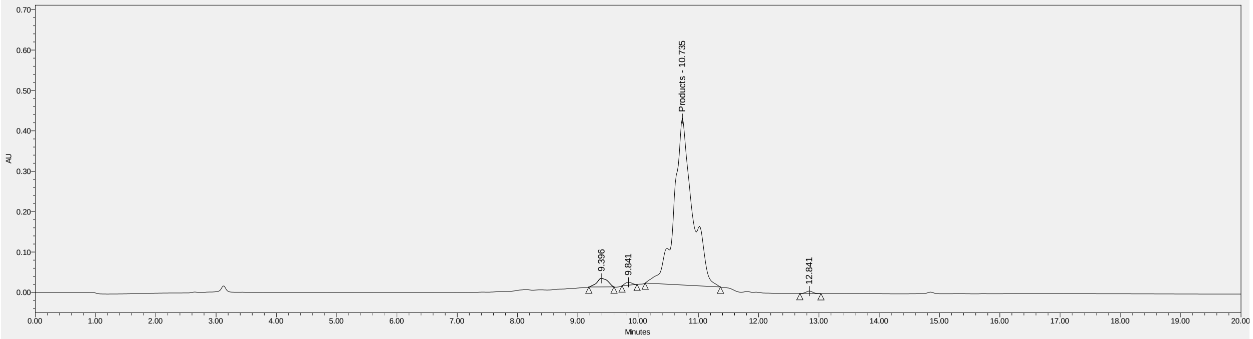

	Name	Retention Time	Area	% Area
1		9.396	272555	2.96
2		9.841	56349	0.61
3	Products	10.735	8842541	95.92
4		12.841	47274	0.51

HRMS (ESI+)

Calculated for $C_{57}H_{85}N_{18}O_{12}$ $(M+H)^+$: 1213.6589

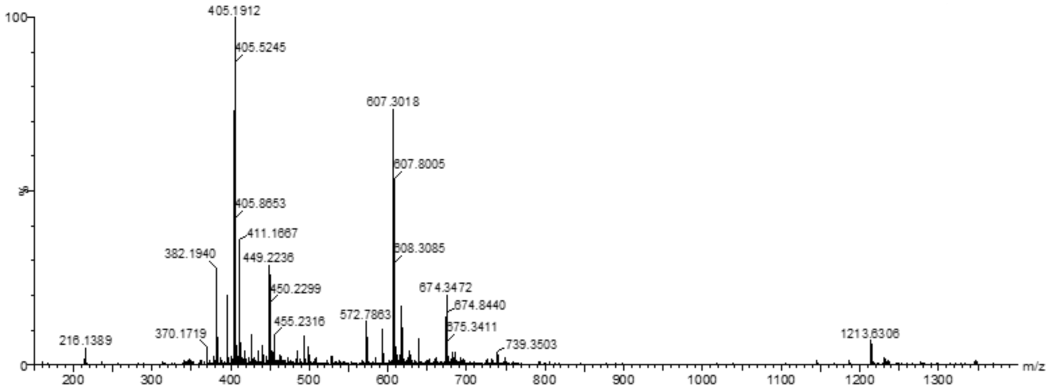

MS/MS (ESI+)

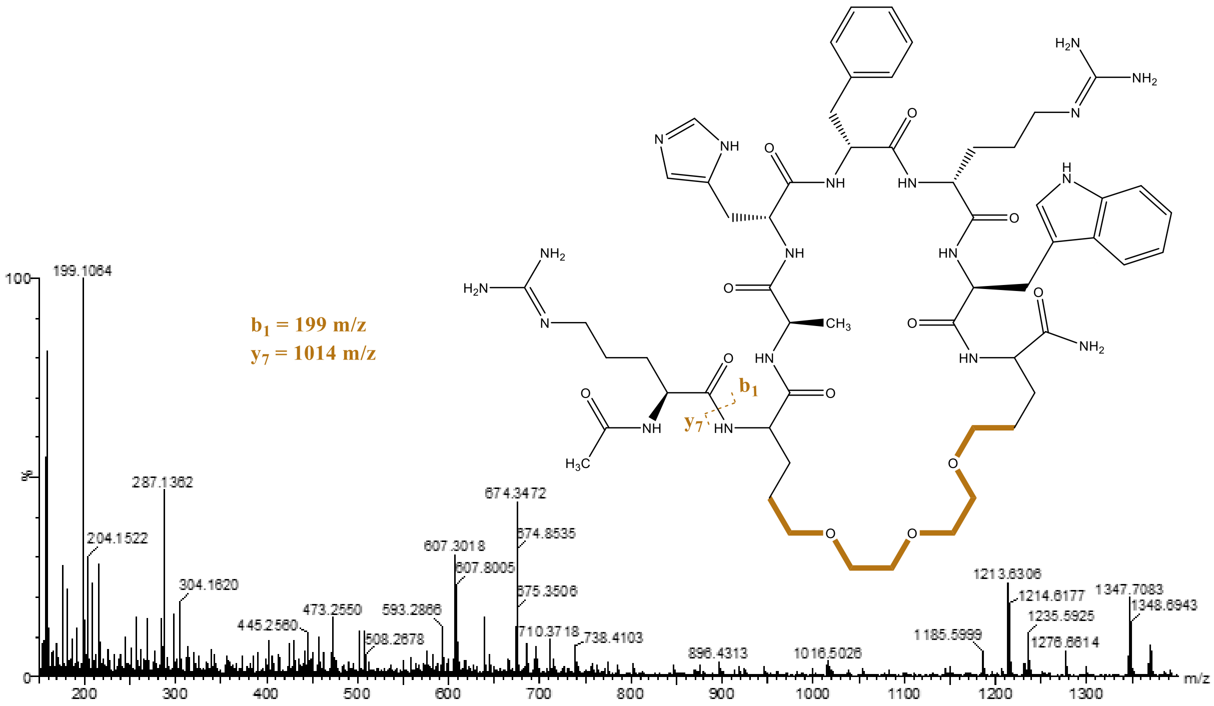

b1 = 199 m/z
y7 = 1014 m/z
199.1064
287.1362
204.1522
304.1620
445.2560
473.2550
508.2678
593.2866
607.3018
607.8005
674.3472
674.8535
675.3506
710.3718
738.4103
896.4313
1016.5026
1185.5999
1213.6306
1214.6177
1235.5925
1276.6614
1347.7083
1348.6943
m/z
%

F_{12}-setmelanotide

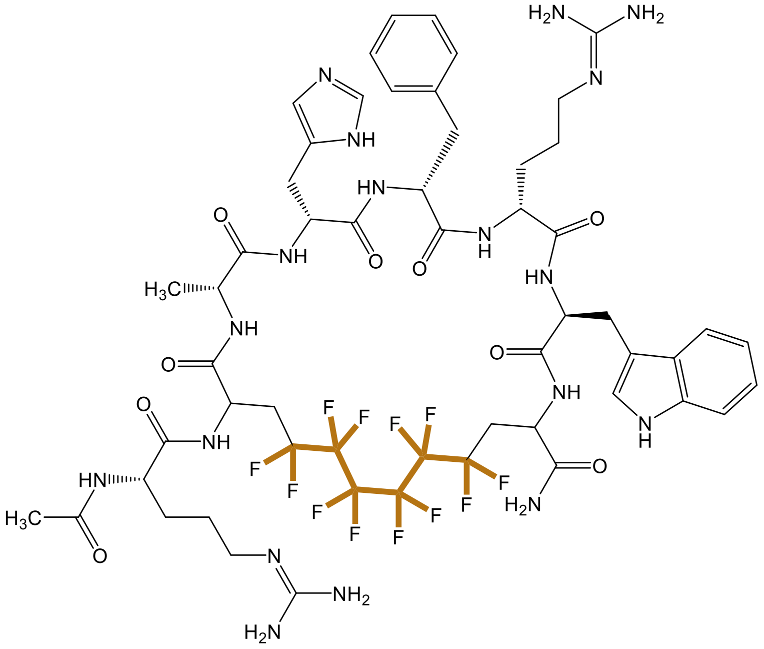

Crude Spectrum

Conversion 27.8%

d.r. 1.5 : 1.9 : 1.0

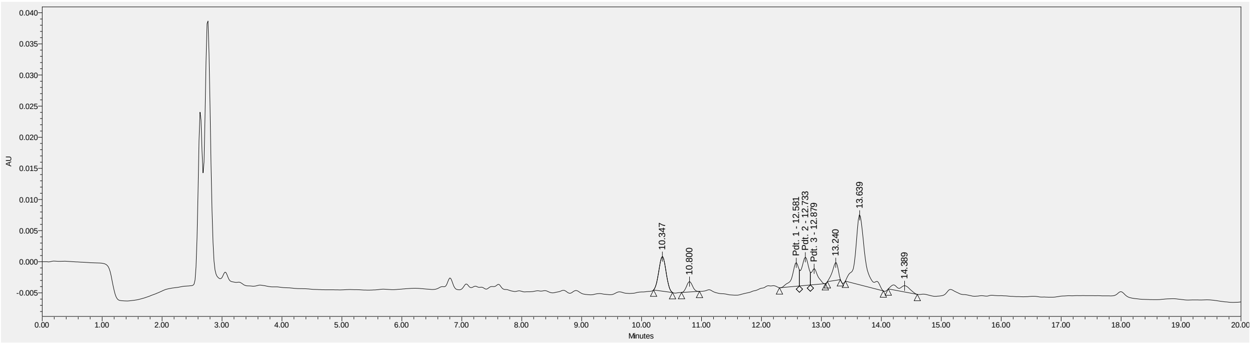

	Name	Retention Time	% Area
1		10.347	15.40
2		10.800	3.83
3	Pdt. 1	12.581	9.33
4	Pdt. 2	12.733	12.13
5	Pdt. 3	12.879	6.38
6		13.240	6.16
7		13.639	41.44
8		14.389	5.33

Isolated Products

Yield 2.91 mg from 10 mg, Purity 98.2%

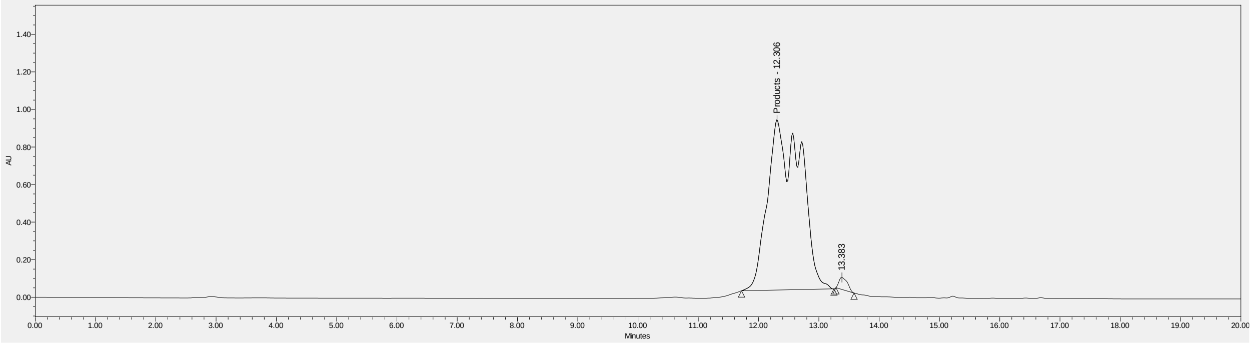

	Name	Retention Time	Area	% Area
1	Products	12.306	35125696	98.24
2		13.383	629381	1.76

HRMS (ESI+)

Calculated for $C_{55}H_{69}F_{12}N_{18}O_9$ $(M+H)^+$: 1353.5298

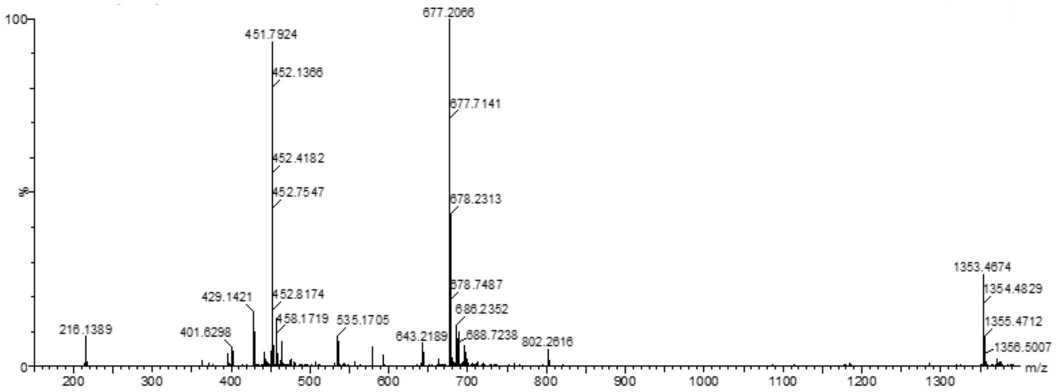

MS/MS (ESI+)

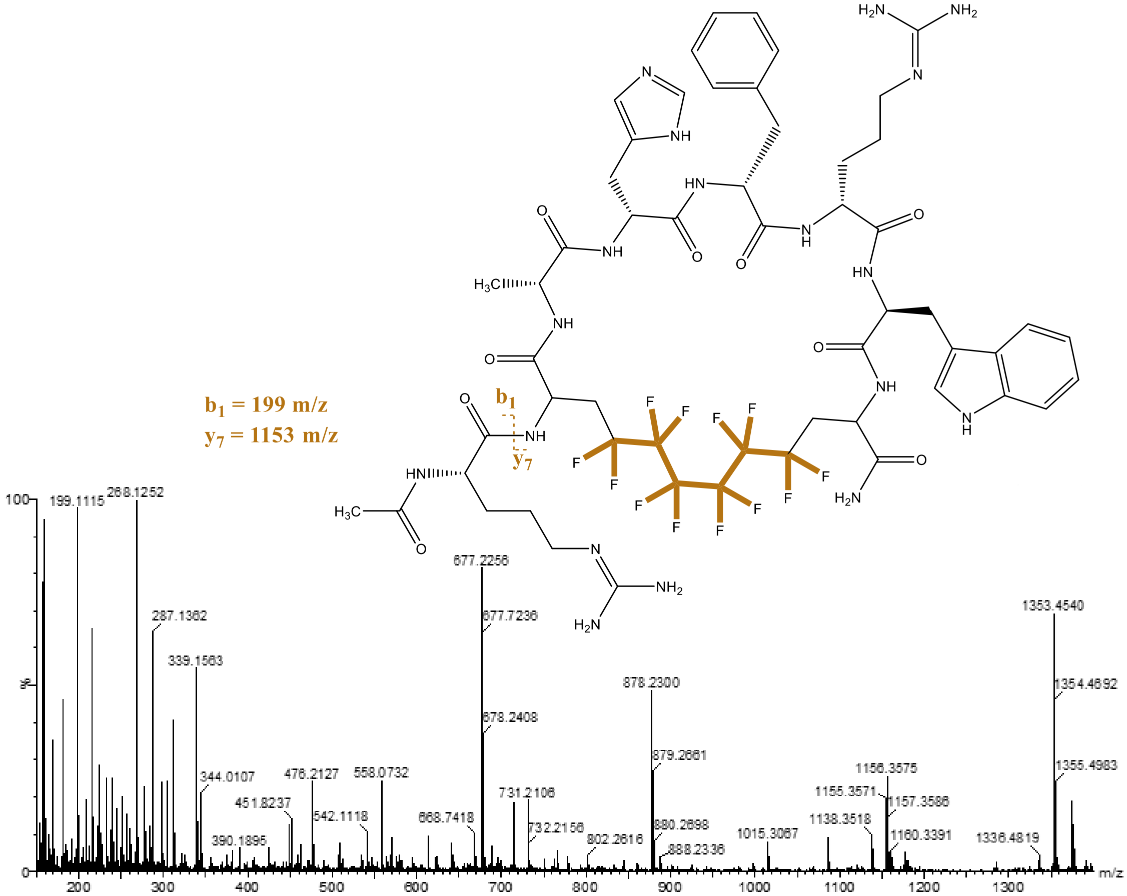
b1 = 199 m/z
y7 = 1153 m/z
199.1115
268.1252
287.1362
339.1563
344.0107
390.1895
451.8237
476.2127
542.1118
558.0732
668.7418
677.2256
677.7236
678.2408
731.2106
732.2156
802.2616
878.2300
879.2661
880.2698
888.2336
1015.3067
1138.3518
1155.3571
1156.3575
1157.3586
1160.3391
1336.4819
1353.4540
1354.4692
1355.4983
m/z
%

Bnz-setmelanotide

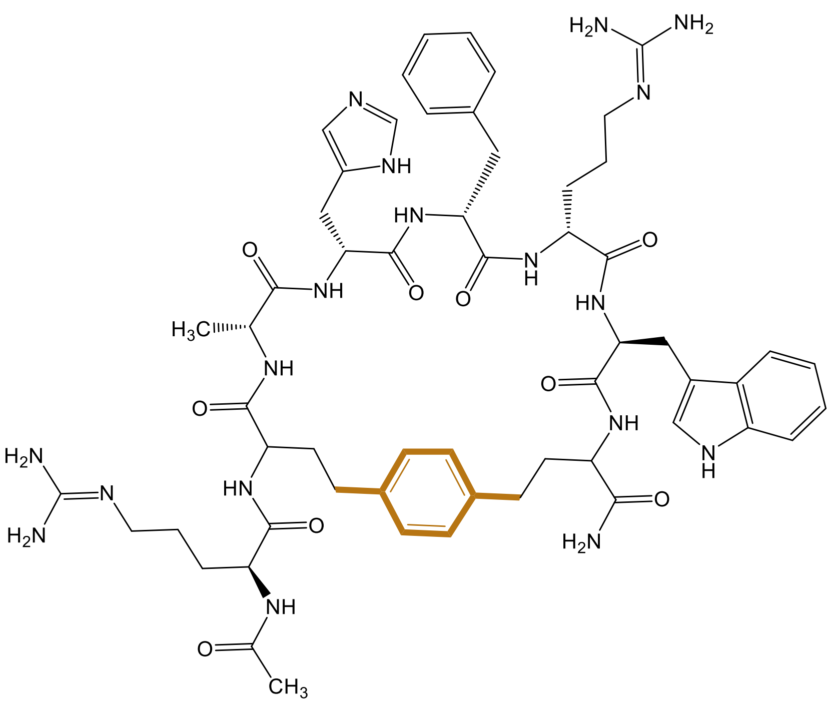

Crude Spectrum

Conversion 17.1%

d.r. 6.3 : 1.3 : 1.0

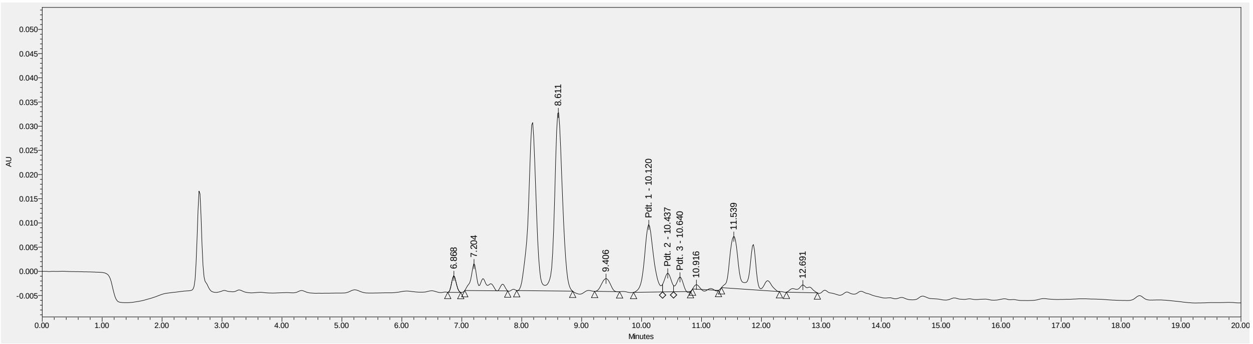

	Name	Retention Time	% Area
1		6.868	1.56
2		7.204	5.61
3		8.611	55.01
4		9.406	2.22
5	Pdt. 1	10.120	12.52
6	Pdt. 2	10.437	2.56
7	Pdt. 3	10.640	2.00
8		10.916	0.66

9		11.539	15.81
10		12.691	2.05

Isolated Products

Yield 0.64 mg from 10 mg, Purity 93.8%

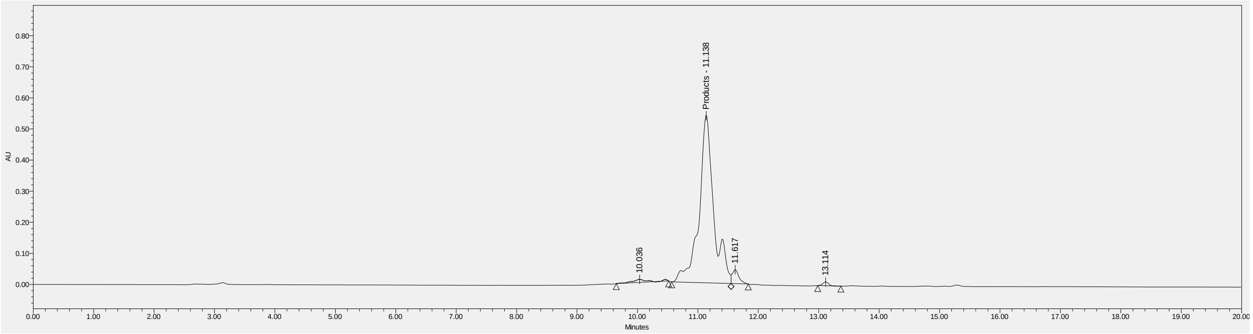

	Name	Retention Time	Area	% Area
1		10.036	174127	1.80
2	Products	11.138	9090106	93.81
3		11.617	343672	3.55
4		13.114	81878	0.84

HRMS (ESI+)

Calculated for $C_{57}H_{77}N_{18}O_9$ $(M+H)^+$: 1157.6116

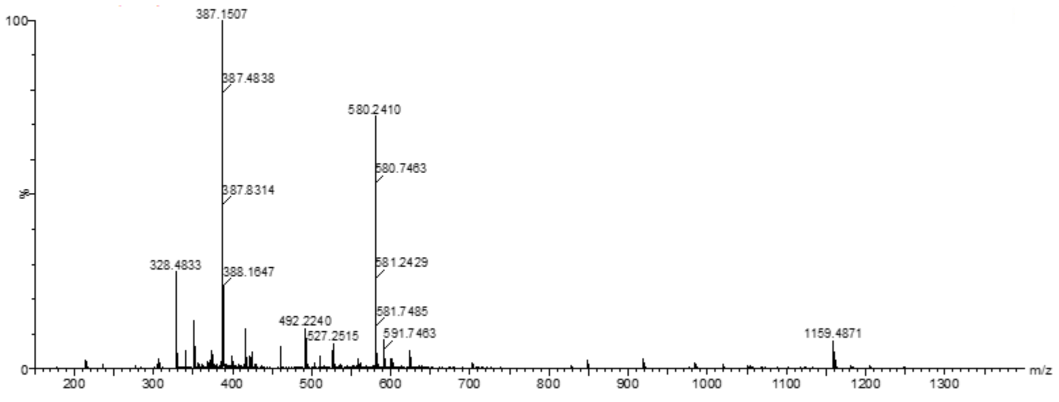

MS/MS (ESI+)

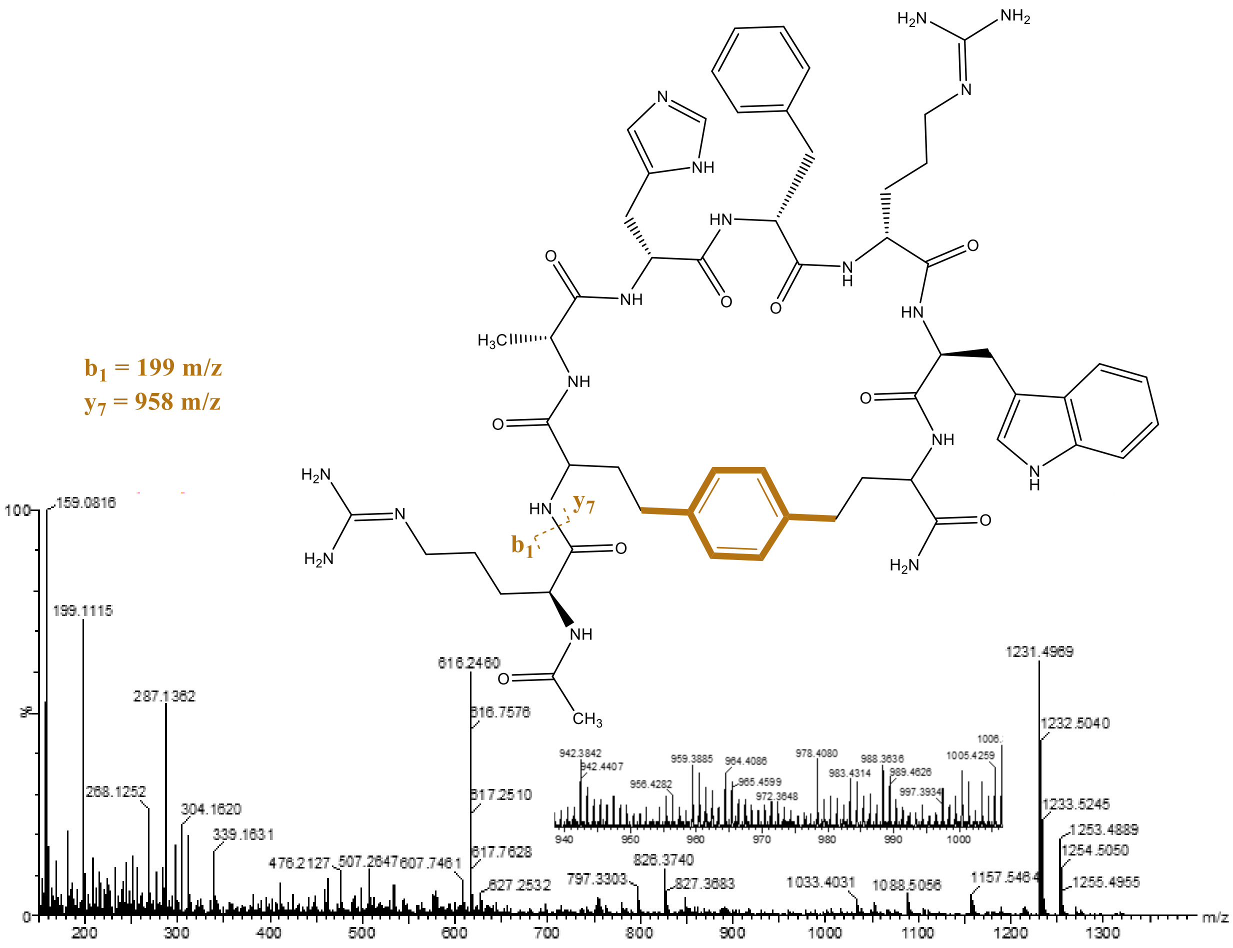

b_1 = 199 m/z
y_7 = 958 m/z
159.0816
199.1115
287.1362
268.1252
304.1620
339.1631
476.2127
507.2647
607.7461
616.2460
616.7576
617.2510
617.7628
627.2532
797.3303
826.3740
827.3683
1033.4031
1088.5056
1157.5464
1231.4969
1232.5040
1233.5245
1253.4889
1254.5050
1255.4955
m/z

Ph-setmelanotide

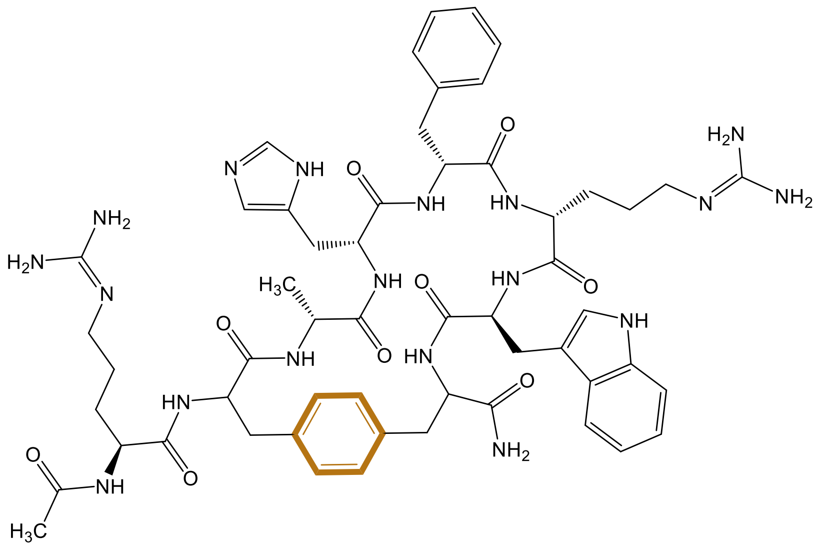

Crude Spectrum

Conversion 28.1%

d.r. 1.1 : 1.3 : 1.5 : 1.0

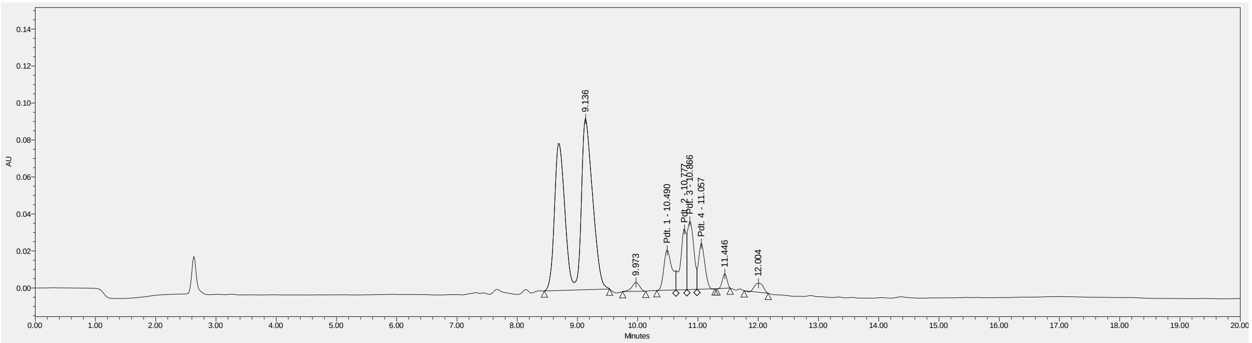

	Name	Retention Time	% Area
1		9.136	67.59
2		9.973	1.37
3	Pdt. 1	10.490	6.56
4	Pdt. 2	10.777	7.22
5	Pdt. 3	10.866	8.59
6	Pdt. 4	11.057	5.71
7		11.446	1.34
8		12.004	1.63

Isolated Products

Yield 0.79 mg from 10 mg, Purity 98.7%

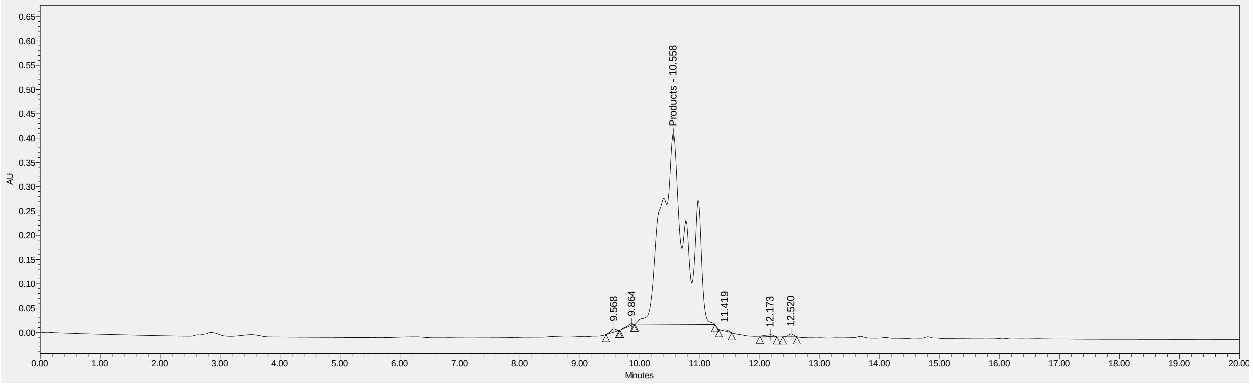

	Name	Retention Time	Area	% Area
1		9.568	43628	0.38
2		9.864	25634	0.22
3	Products	10.558	11340126	98.65
4		11.419	17367	0.15
5		12.173	32193	0.28
6		12.520	35868	0.31

HRMS (ESI+)

Calculated for $C_{55}H_{73}N_{18}O_{9}$ $(M+H)^{+}$: 1129.5803

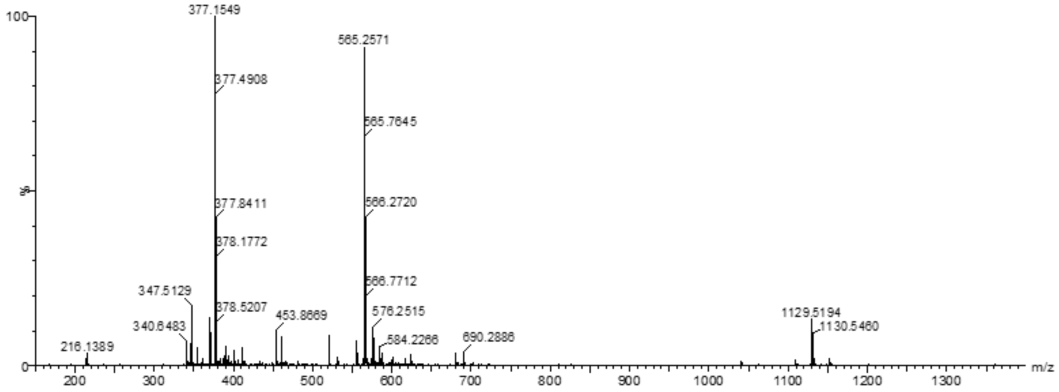

MS/MS (ESI+)

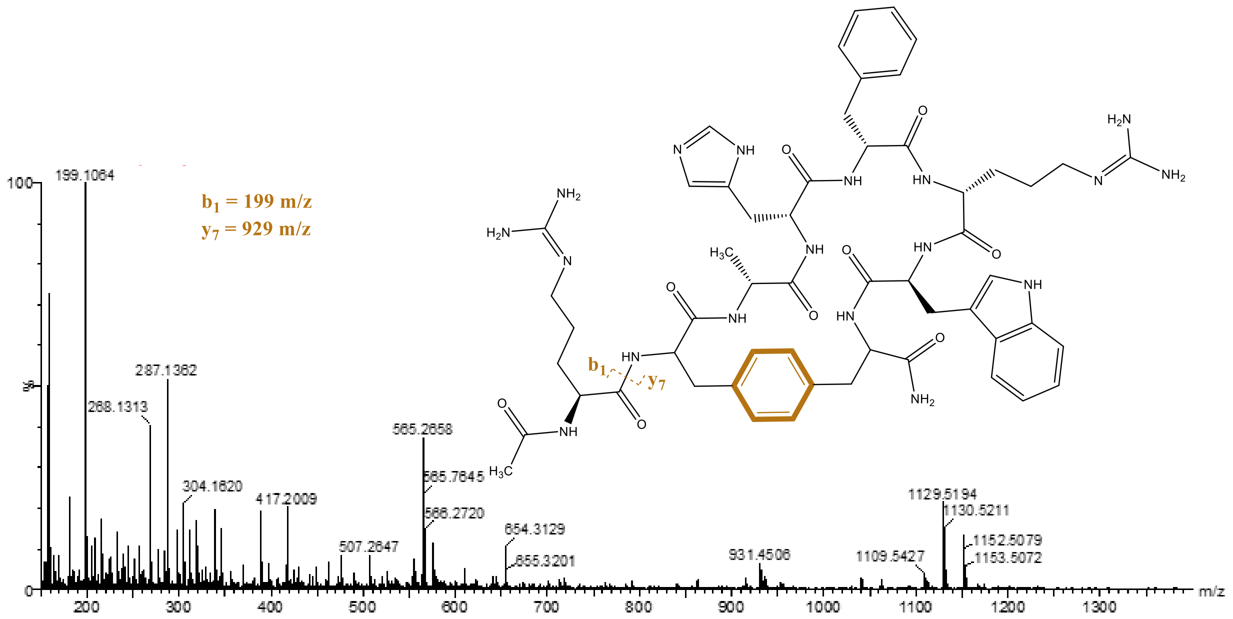

199.1064
b_1 = 199 m/z
y_7 = 929 m/z
287.1362
268.1313
304.1620
417.2009
565.2658
565.7645
566.2720
507.2647
654.3129
655.3201
931.4506
1109.5427
1129.5194
1130.5211
1152.5079
1153.5072
m/z

Ad-setmelanotide

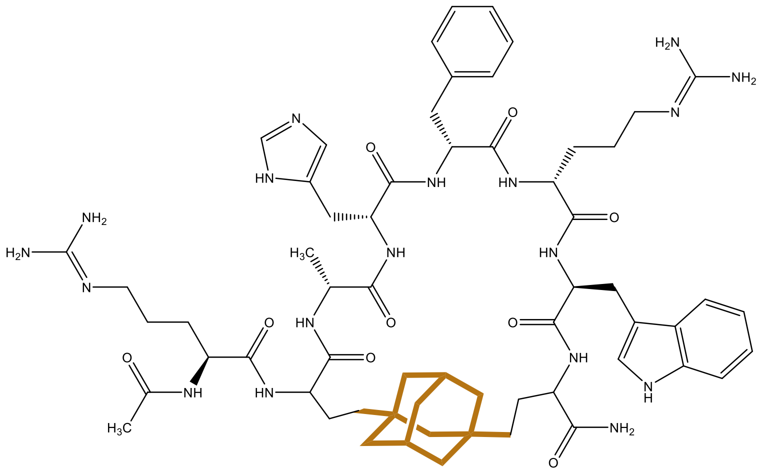

Crude Spectrum

Conversion 47.1%

d.r. 1.0 : 1.0 : 1.1

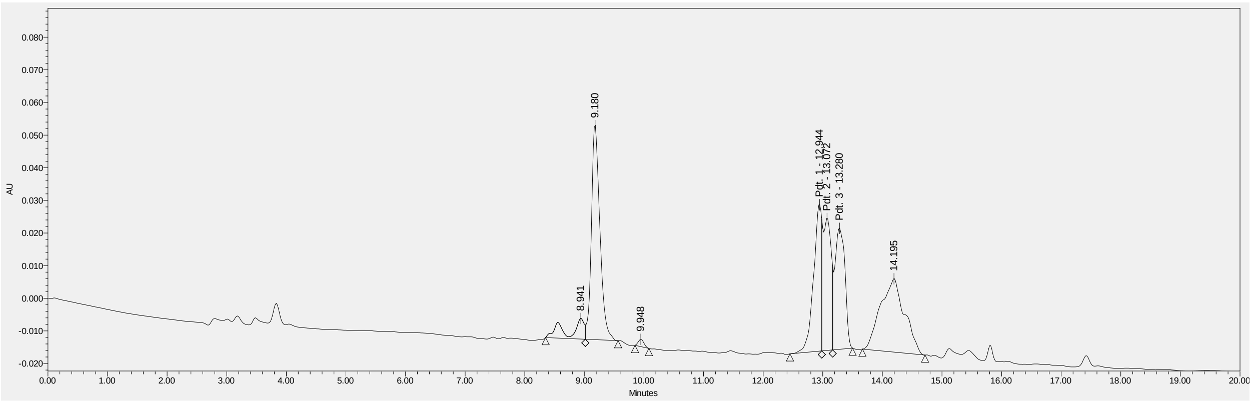

	Name	Retention Time	Area	% Area
1		8.941	107329	4.04
2		9.180	653924	24.61
3		9.948	14786	0.56
4	Pdt. 1	12.944	407030	15.32
5	Pdt. 2	13.072	399408	15.03
6	Pdt. 3	13.280	444378	16.73
7		14.195	629812	23.71

Isolated Products

Yield 0.48 mg from 10 mg, Purity 91.1%

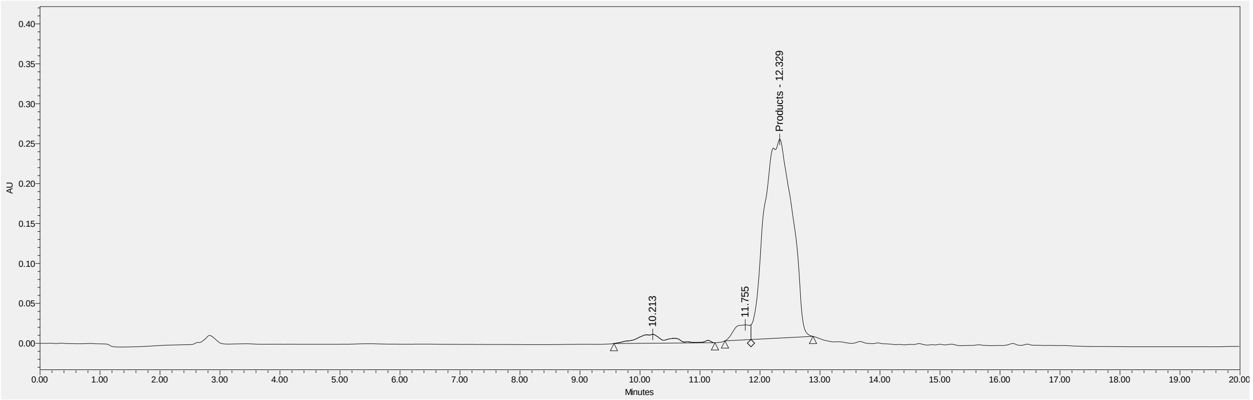

	Name	Retention Time	Area	% Area
1		10.213	421742	4.79
2		11.755	351131	3.98
3	Products	12.329	8040545	91.23

HRMS (ESI+)

Calculated for $C_{61}H_{87}N_{18}O_9$ $(M+H)^+$: 1215.6898

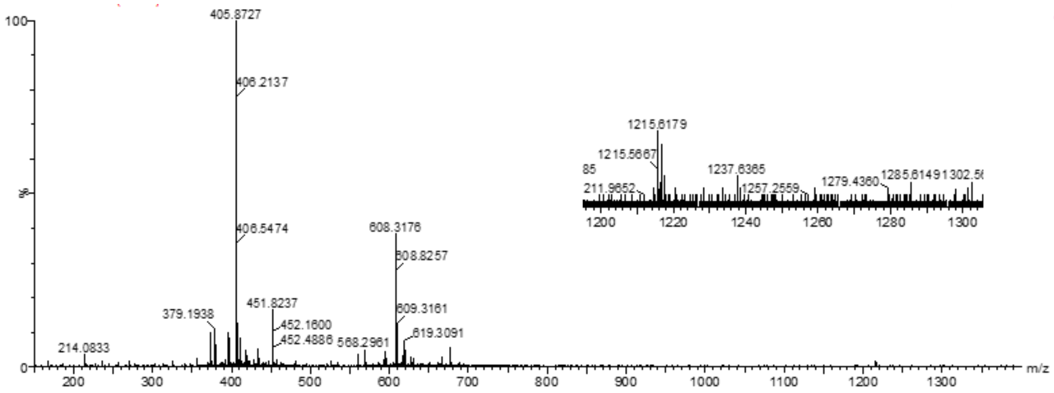

MS/MS (ESI+)

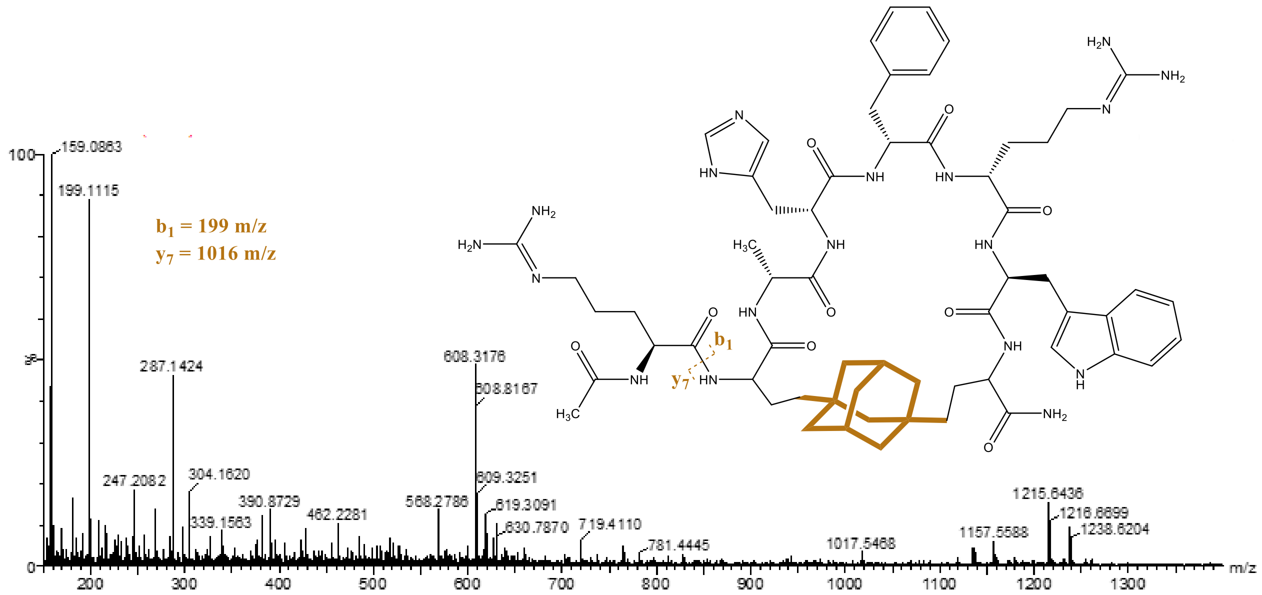

b1 = 199 m/z
y7 = 1016 m/z
159.0863
199.1115
287.1424
247.2082
304.1620
339.1563
390.8729
462.2281
568.2786
608.3176
608.8167
609.3251
619.3091
630.7870
719.4110
781.4445
1017.5468
1157.5588
1215.6436
1216.6699
1238.6204
m/z

Characterization of Other Carbocyclic Peptides

C_5-vapreotide

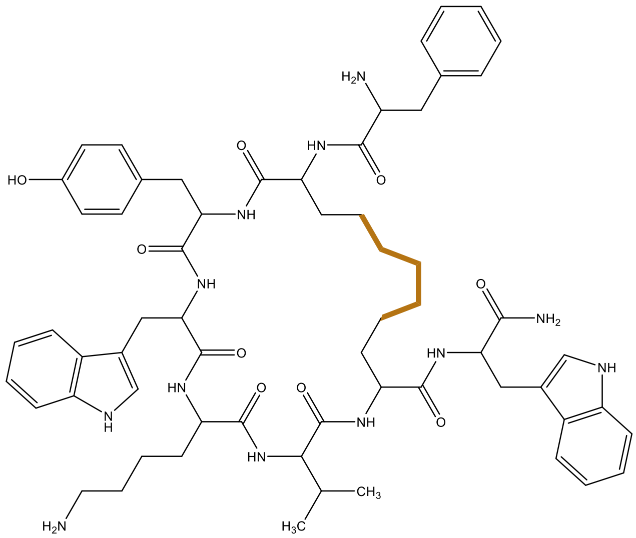

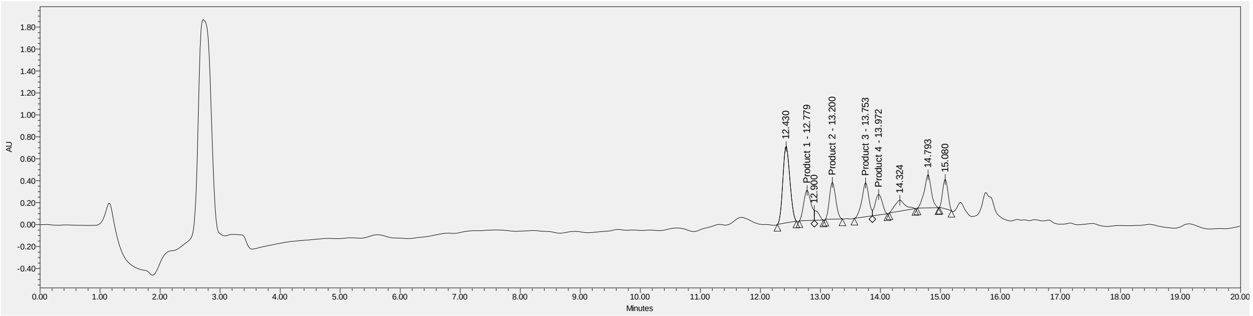

	Name	Retention Time	Area	% Area
1		12.430	5662675	28.06
2	Product 1	12.779	2335843	11.57
3		12.900	458728	2.27
4	Product 2	13.200	2426764	12.03
5	Product 3	13.753	2487944	12.33
6	Product 4	13.972	1535124	7.61
7		14.324	1194087	5.92
8		14.793	2463928	12.21
9		15.080	1615234	8.00

HRMS (ESI+)

Calculated for $C_{62}H_{81}N_{12}O_9$ $(M+H)^+$: 1137.6244

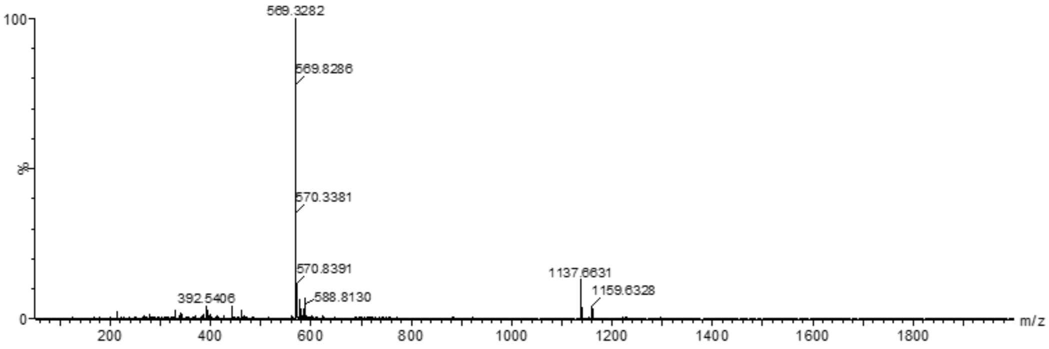

MS/MS (ESI+)

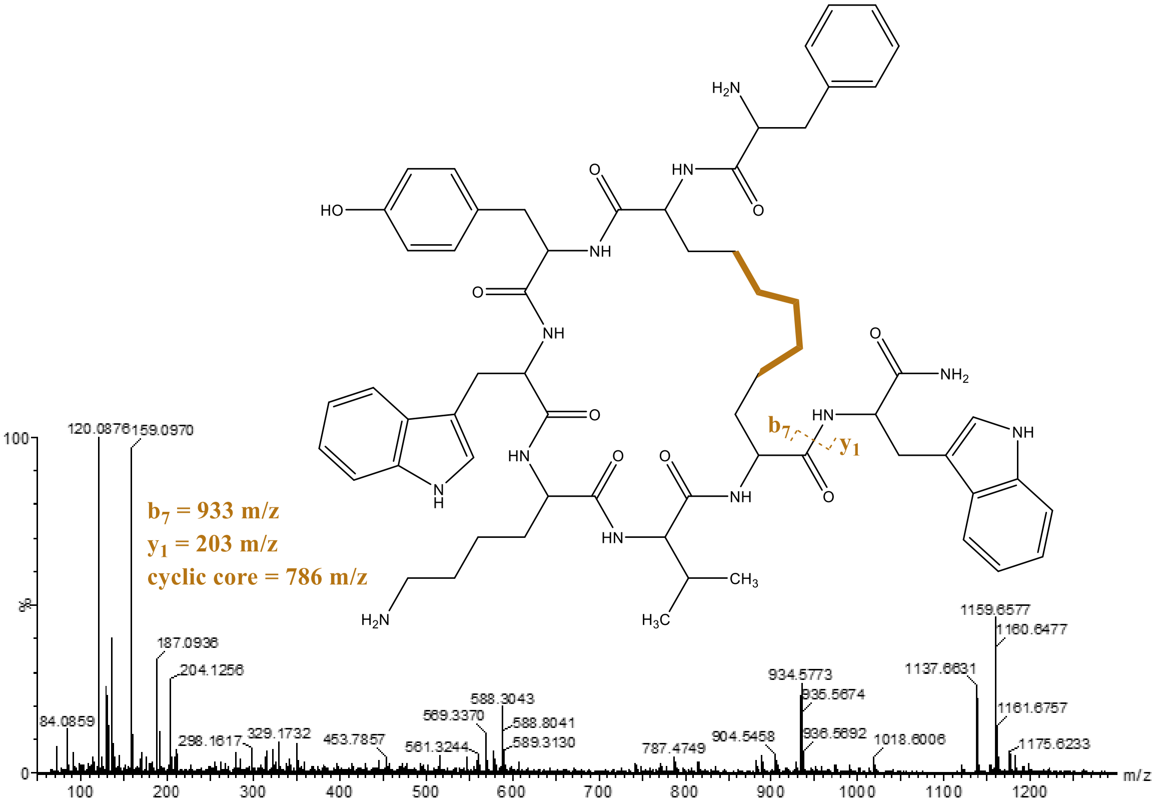

C$_5$-terlipressin

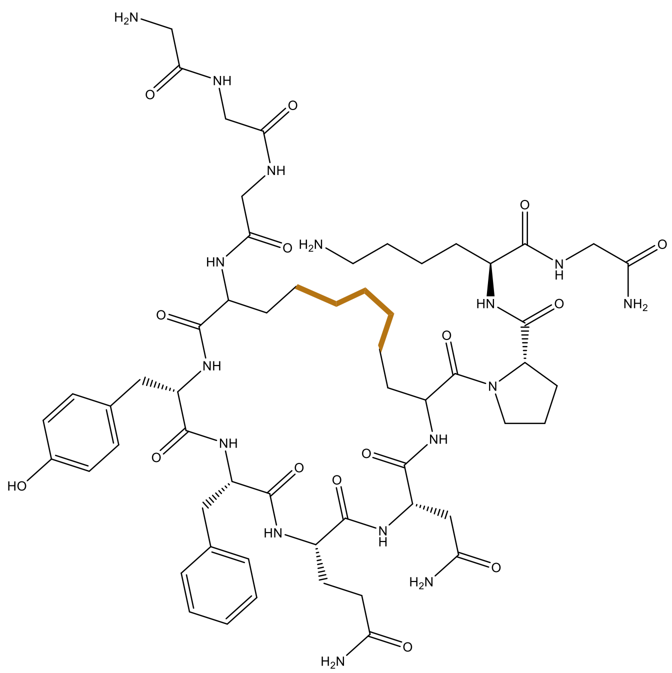

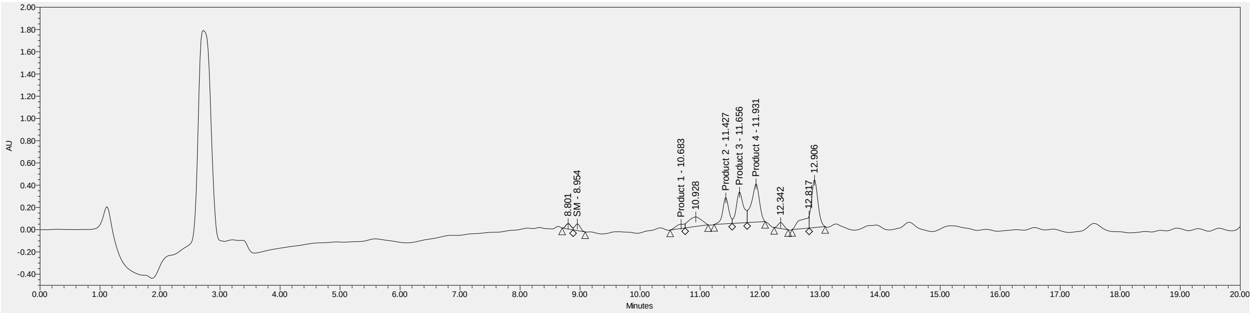

	Name	Retention Time	Area	% Area
1		8.801	295025	2.08
2	SM	8.954	372598	2.63
3	Product 1	10.683	379298	2.68
4		10.928	1277350	9.03
5	Product 2	11.427	1647552	11.64
6	Product 3	11.656	2349981	16.60
7	Product 4	11.931	3149098	22.25
8		12.342	359991	2.54
9		12.817	1124532	7.95
10		12.906	3197738	22.59

HRMS(ESI+)

Calculated for $C_{57}H_{85}N_{16}O_{15}$ $(M+H)^+$: 1233.6375

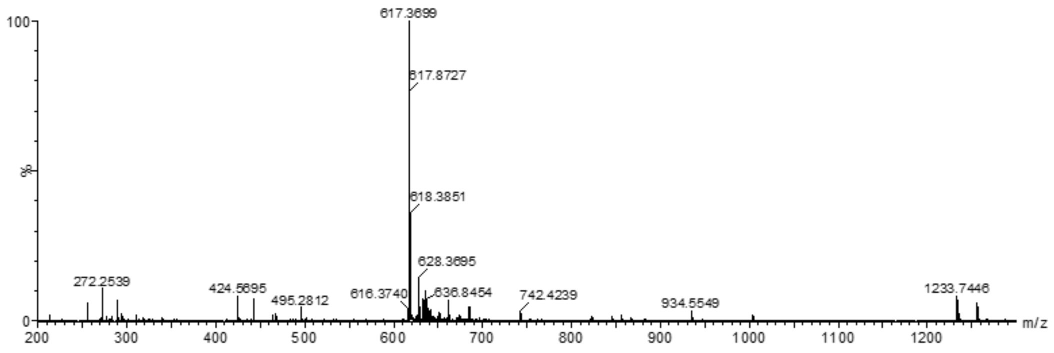

MS/MS (ESI+)

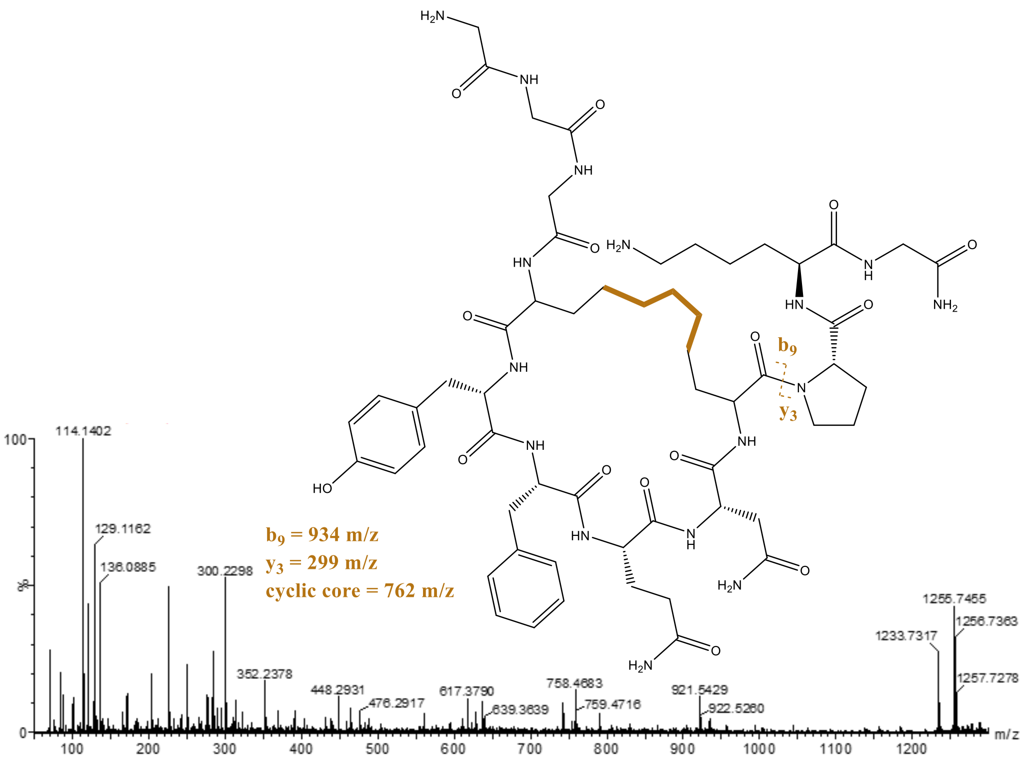

C$_5$-eptifibatide

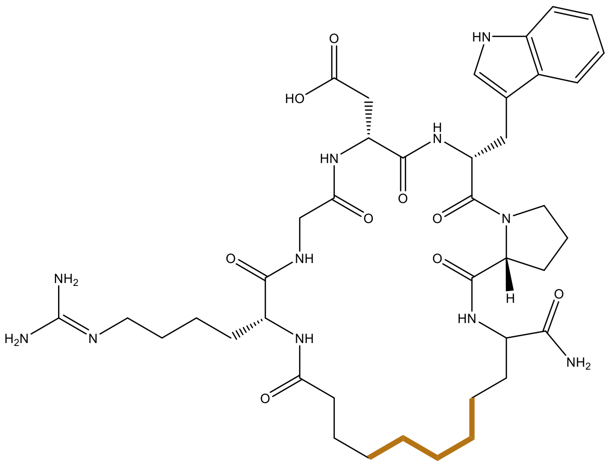

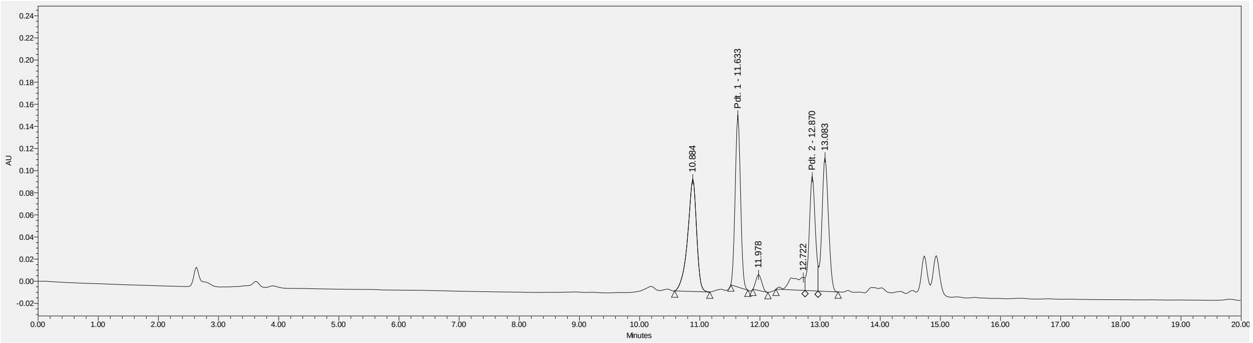

	Name	Retention Time	Area	% Area
1		10.884	1046184	26.61
2	Pdt. 1	11.633	957217	24.35
3		11.978	95429	2.43
4		12.722	195620	4.98
5	Pdt. 2	12.870	717147	18.24
6		13.083	919422	23.39

HRMS (ESI+)

Calculated for $C_{40}H_{59}N_{11}O_9$ $(M+H)^+$: 838.4570

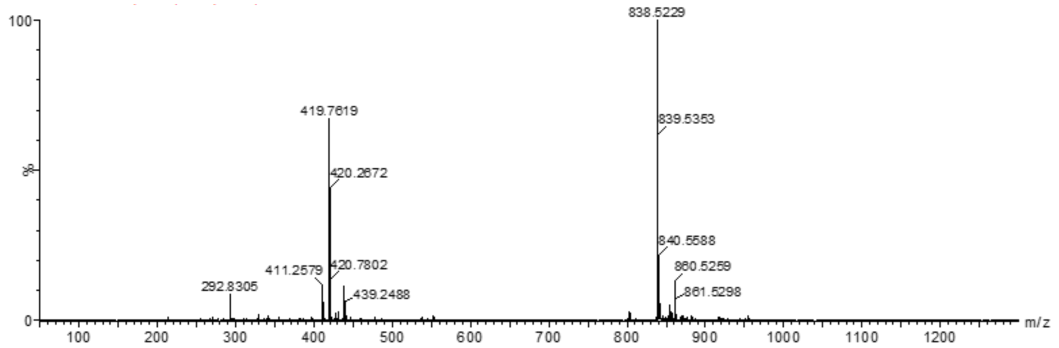

MS/MS (ESI+)

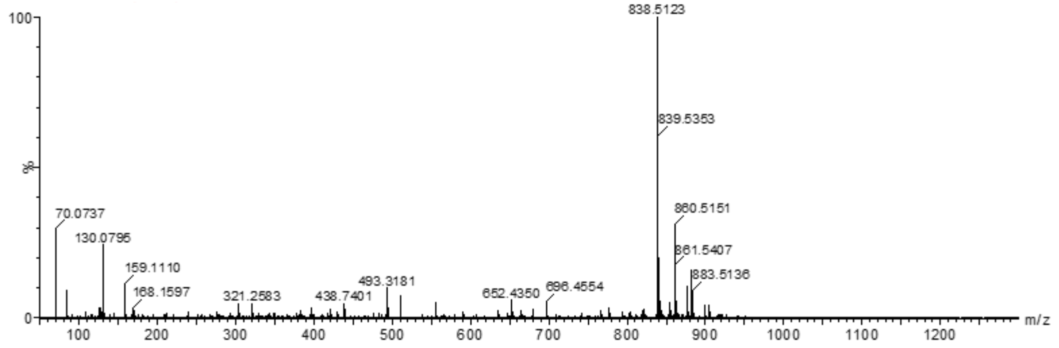

C$_5$-desmopressin

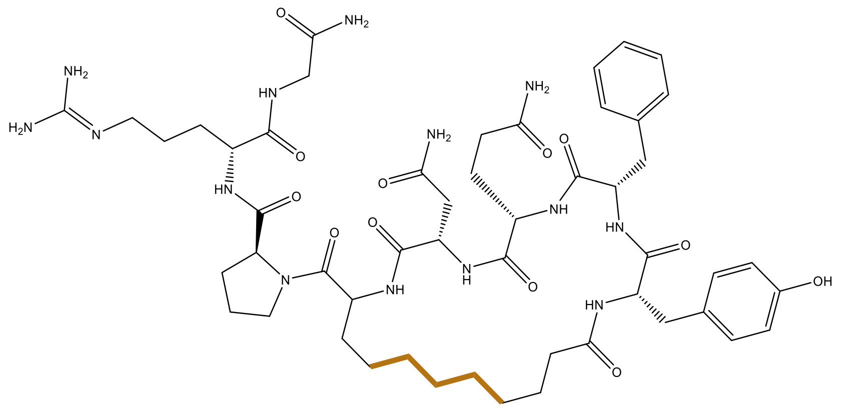

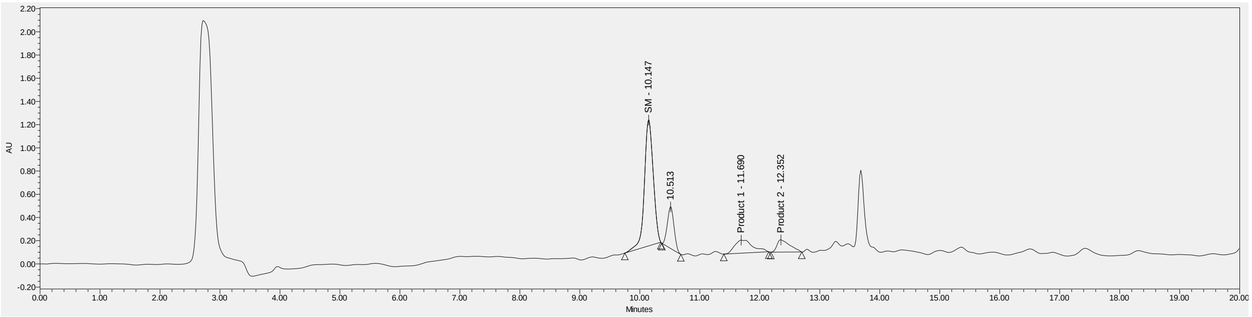

	Name	Retention Time	Area	% Area
1	SM	10.147	11133281	62.37
2		10.513	2771962	15.53
3	Product 1	11.690	2373090	13.29
4	Product 2	12.352	1572612	8.81

HRMS (ESI+)

Calculated for $C_{51}H_{75}N_{14}O_{12}$ $(M+H)^+$: 1075.5684

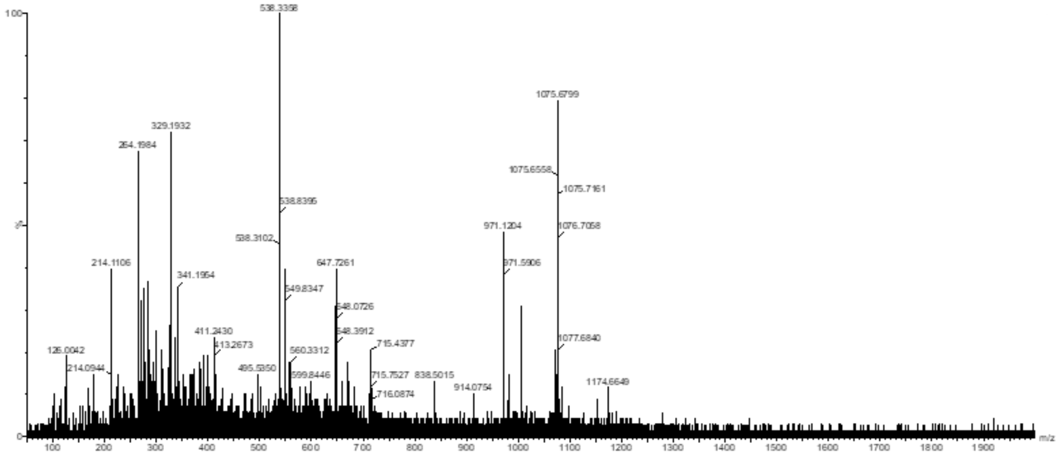

MS/MS (ESI+)

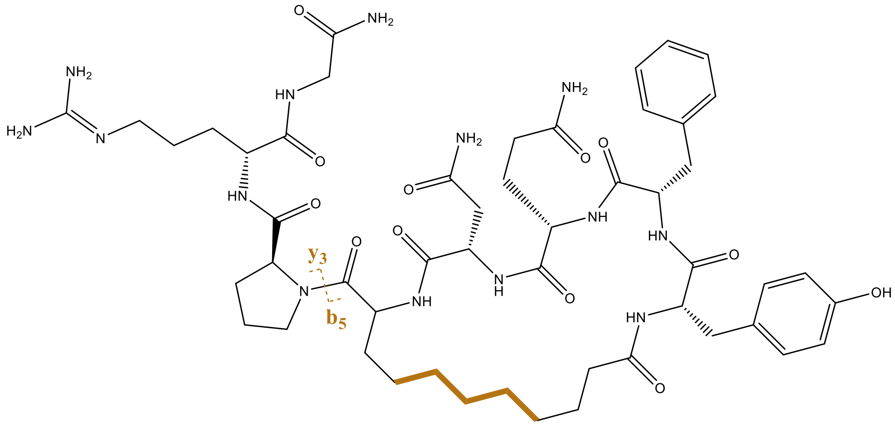

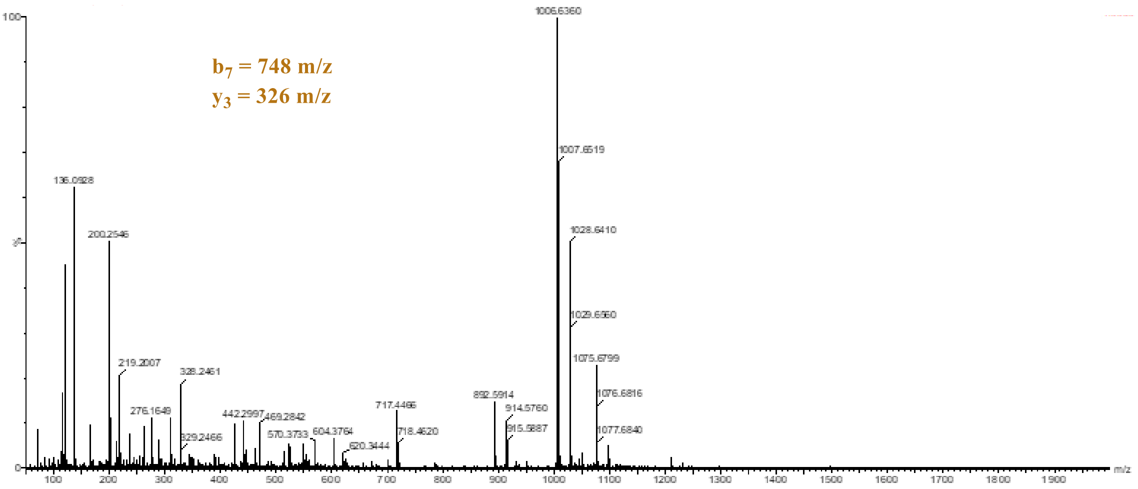

Generation of Setmelanotide Standard Curve

Setmelanotide (7.5 mg) was dissolved in 1.500 mL of ddH_2O. Additional concentrations were prepared by repeated 2-fold serial dilutions, with the final each volume of each sample 0.750 mL. Each sample was analyzed by LC-MS and the peak area recorded. A total of three independent replicates were conducted and average areas were plotted against setmelanotide concentration.

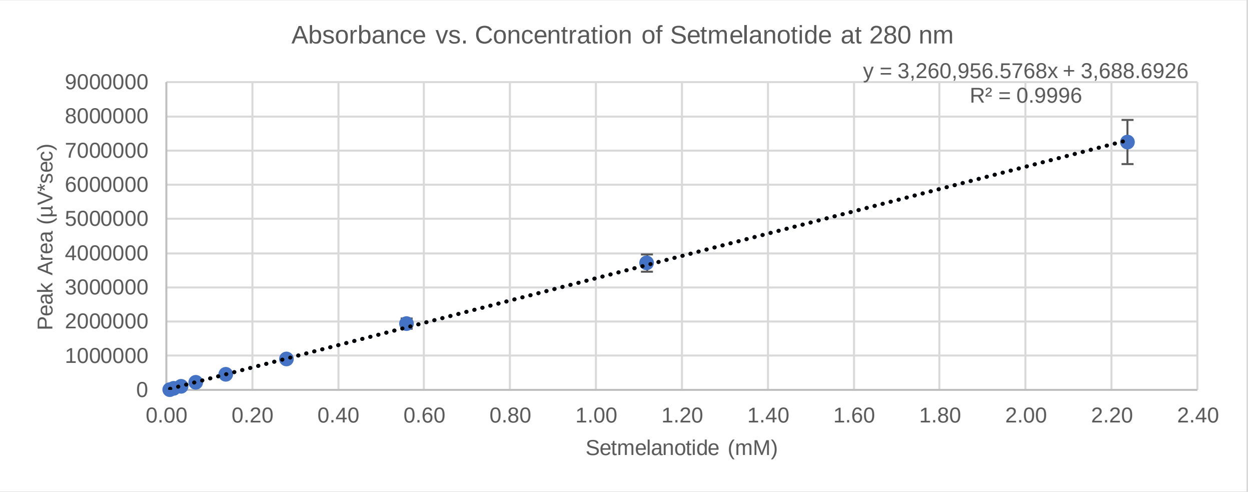

Setmelanotide (mg/ml)	Setmelanotide (mM)	Average Area (µV*sec)	Area1 (µV*sec)	Area2 (µV*sec)	Area3 (µV*sec)	Standard Deviation	Standard Error
5.00	4.47	10234021	16493291	12934261	1274511	7960608.533	4596059
2.50	2.24	7248655.667	8420898	7131611	6193458	1118323.222	645664.2
1.25	1.12	3708221	4212542	3429781	3482340	437544.701	252616.6
0.625	0.559	1937516	2073914	2100745	1637889	259831.1567	150013.6
0.313	0.280	898961.6667	987218	912359	797308	95661.21696	55230.03
0.156	0.140	440098.6667	485996	421256	413044	39959.76904	23070.78
0.0781	0.0699	223233.3333	229430	192057	248213	28586.23956	16504.27
0.0391	0.0350	98307.33333	95765	90770	108387	9079.497031	5242.05
0.0195	0.0175	34097	33761	33839	34691	515.8953382	297.8523
0.00977	0.00874	8366.333333	6842	11606	6651	2807.258508	1620.771

Pharmacokinetic Studies

Procedure for Solubility Determination

The isolated peptide was initially fully dissolved in 100 µL of 1:1:1 H_2O:MeCN:MeOH and analyzed by UPLC to determine total peptide amount using the standard curve generated on page **S98**. The solvent was then removed under reduced pressure and the peptide lyophilized. To the lyophilized peptide was added ddH_2O to give a concentration of 1.0 mg/mL. The solution was briefly vortexed, sonicated for 30 seconds, filtered, and again analyzed by UPLC. All peptides were fully soluble at 1.0 mg/mL.

Procedure for Permeability Determination

In a 600 µL vial, the lyophilized isolated peptide was dissolved in 100 µL of ethylene glycol. 100 µL of heptane was then added, the vial was capped, and the biphasic mixture was vortexed rapidly for 30 seconds. The layers were then allowed 10 minutes for separate fully. 75 µL of each layer was carefully removed, both of which were then diluted with an additional 75 µL of 1:1 MeCN:MeOH. The samples were analyzed by UPLC and total product peak area at 280 nm between the two fractions was recorded. P_{eh} was calculated as follows: $P_{eh} = \log([\text{area}_{\text{heptane}}]/[\text{area}_{\text{ethylene glycol}}])$.

Procedure for Hydrolytic Stability Determination

In a 2 mL vial containing a magnetic stir bar, the lyophilized peptide was dissolved in 1.00 mL of 50 mM potassium phosphate buffer, pH 8.5. The vial was placed in a preheated water bath at 50 °C and stirred for 14 hours. Samples were then immediately analyzed by LC-MS at 280 nm.

LC Spectra

F$_{12}$-Setmelanotide—Ethylene Glycol

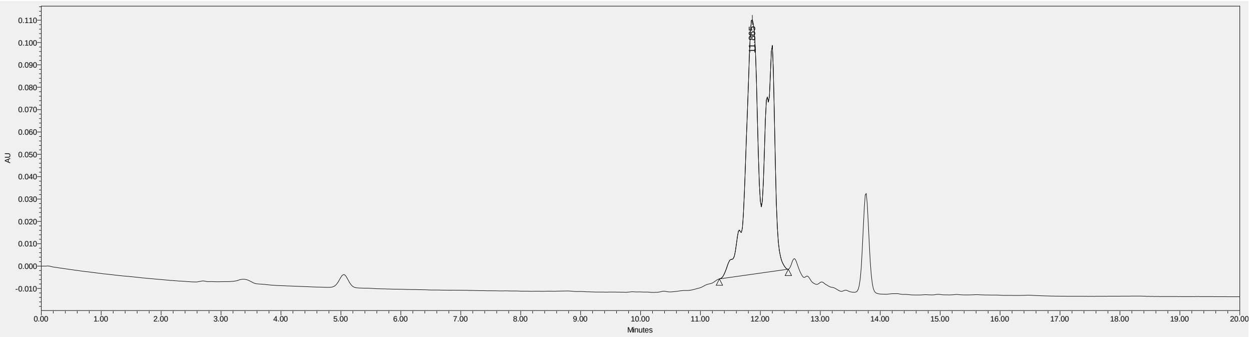

	Retention Time	Area
1	11.865	2704578

F₁₂-Setmelanotide––Heptane

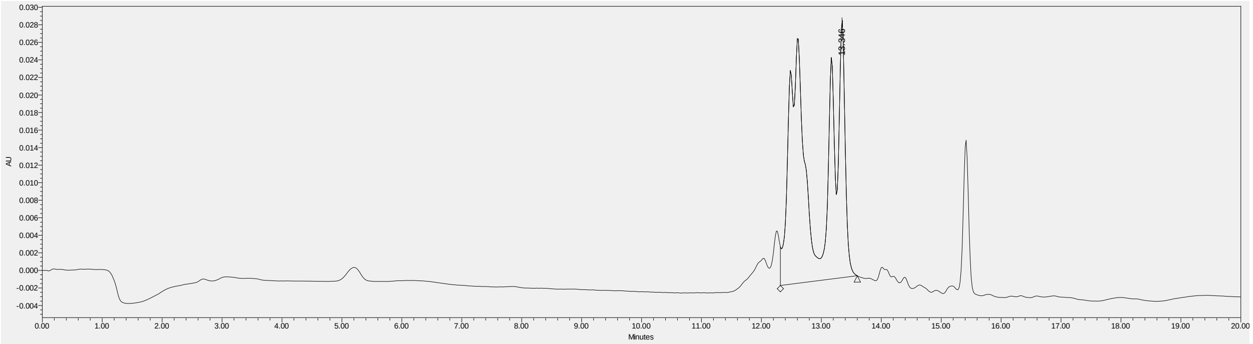

	Retention Time	Area
1	13.346	910219

Ph-Setmelanotide––Ethylene Glycol

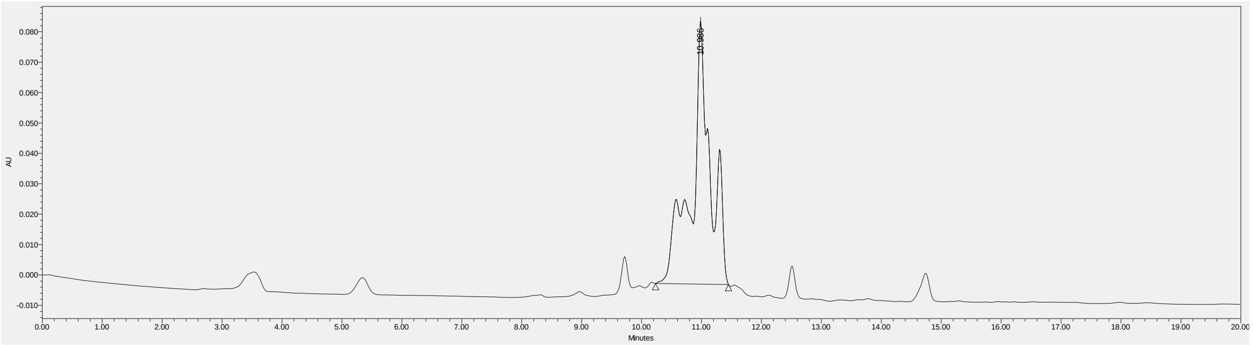

	Retention Time	Area
1	10.986	1900814

Ph-Setmelanotide––Heptane

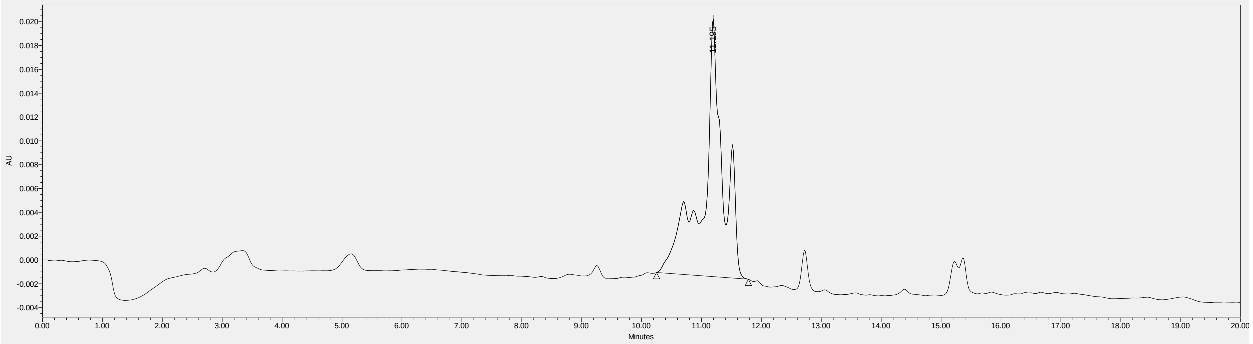

	Retention Time	Area
1	11.195	506251

PEG-Setmelanotide––Ethylene Glycol

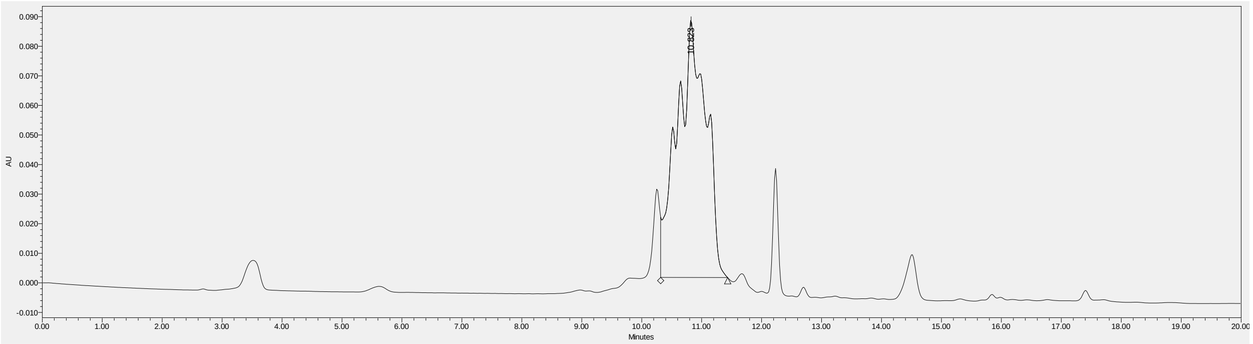

	Retention Time	Area
1	10.823	2959540

PEG-Setmelanotide––Heptane

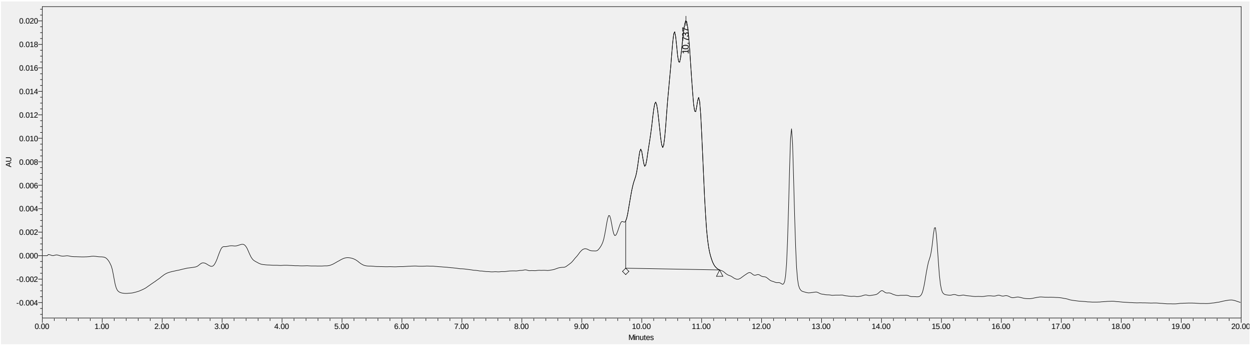

	Retention Time	Area
1	10.737	1052964

C_4-Setmelanotide—Ethylene Glycol

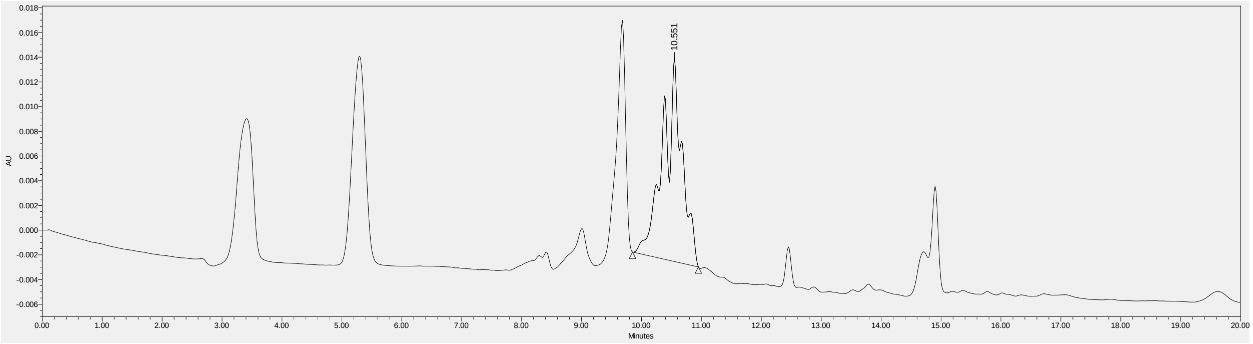

	Retention Time	Area
1	10.551	364299

C_4-Setmelanotide—Heptane

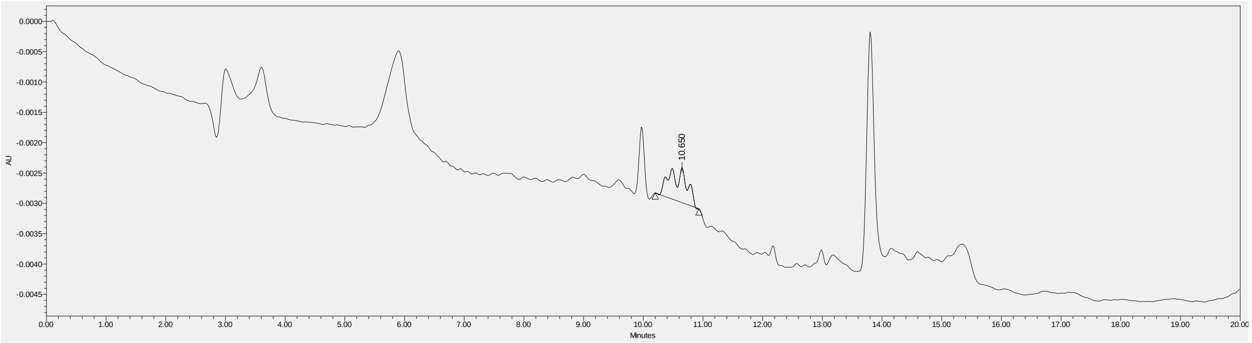

	Retention Time	Area
1	10.650	11127

Setmelanotide—Ethylene Glycol

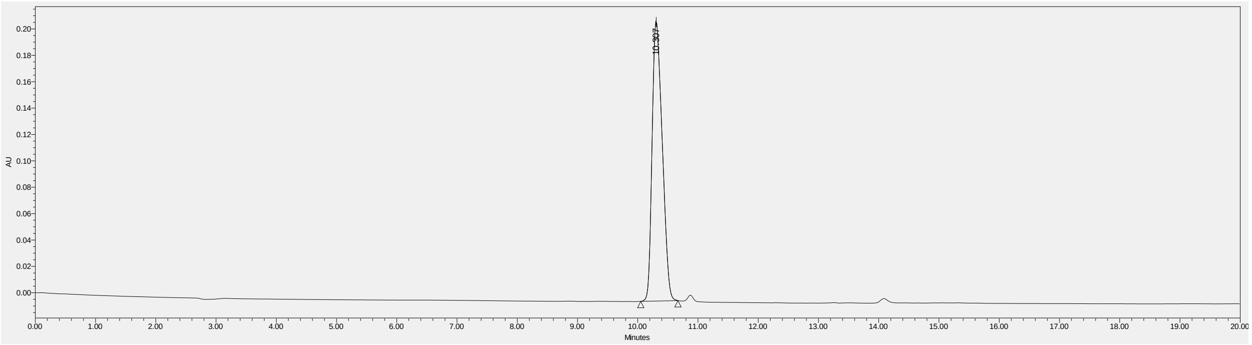

	Retention Time	Area
1	10.307	2482657

Setmelanotide—Heptane

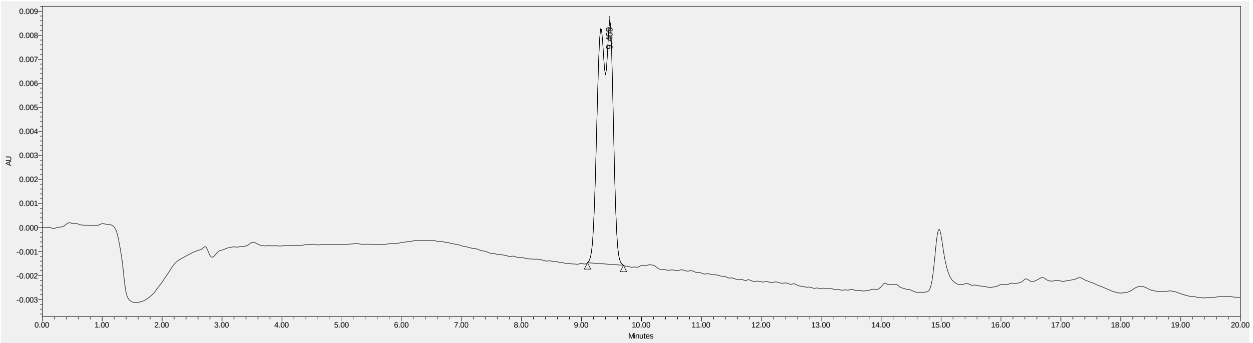

	Retention Time	Area
1	9.469	171644

F$_{12}$-Setmelanotide––Hydrolytic Stability

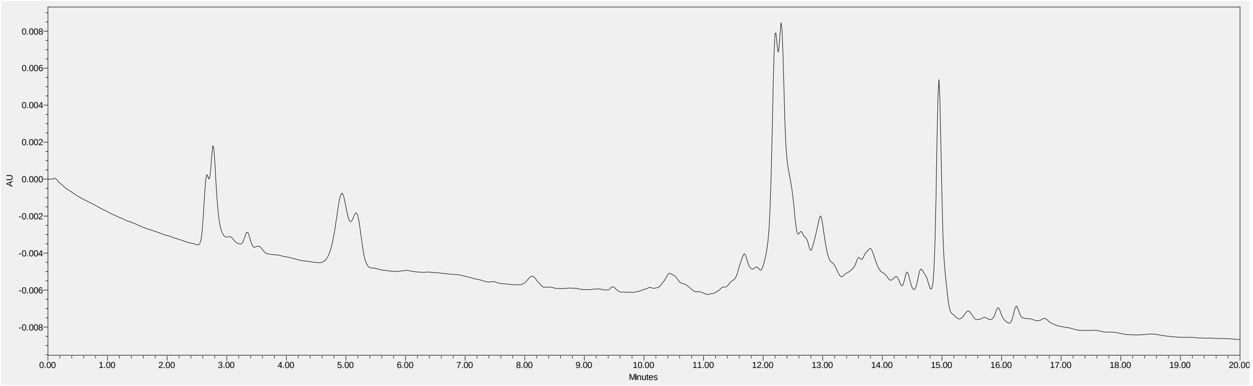

Ph-Setmelanotide––Hydrolytic Stability

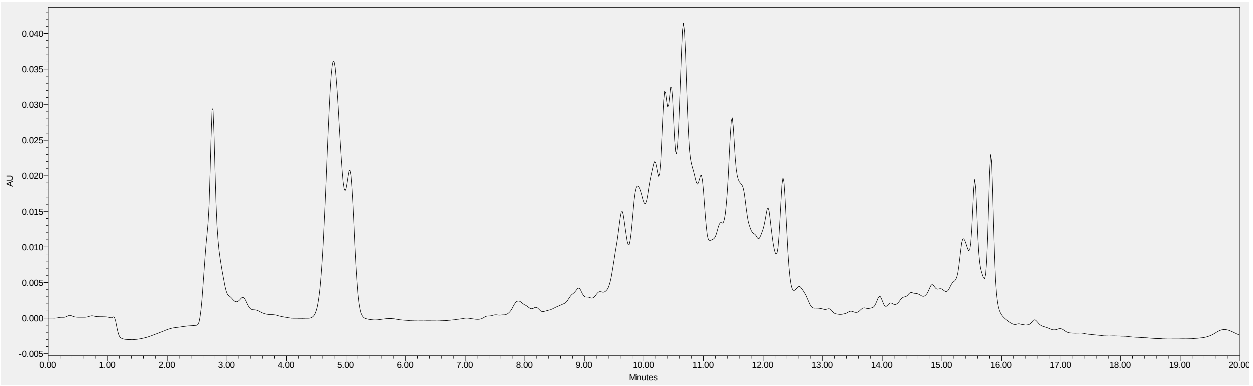

PEG-Setmelanotide––Hydrolytic Stability

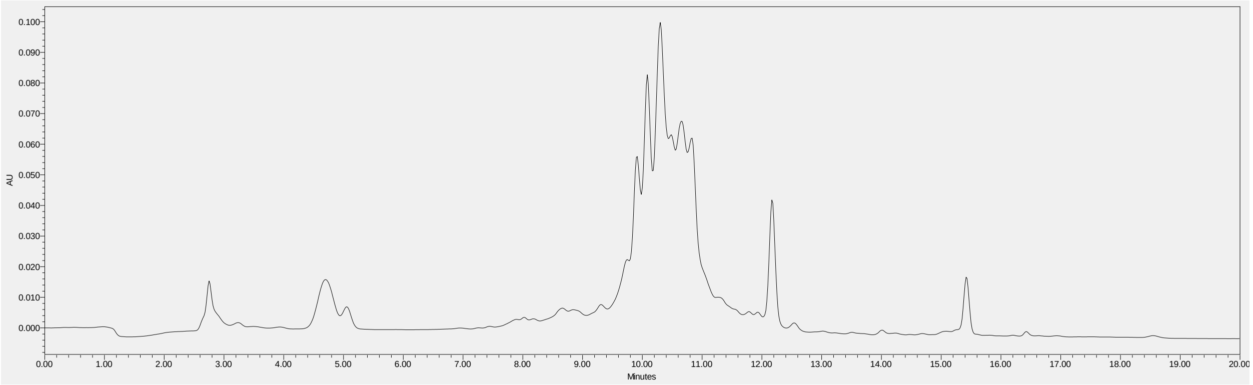

C_4-Setmelanotide—Hydrolytic Stability

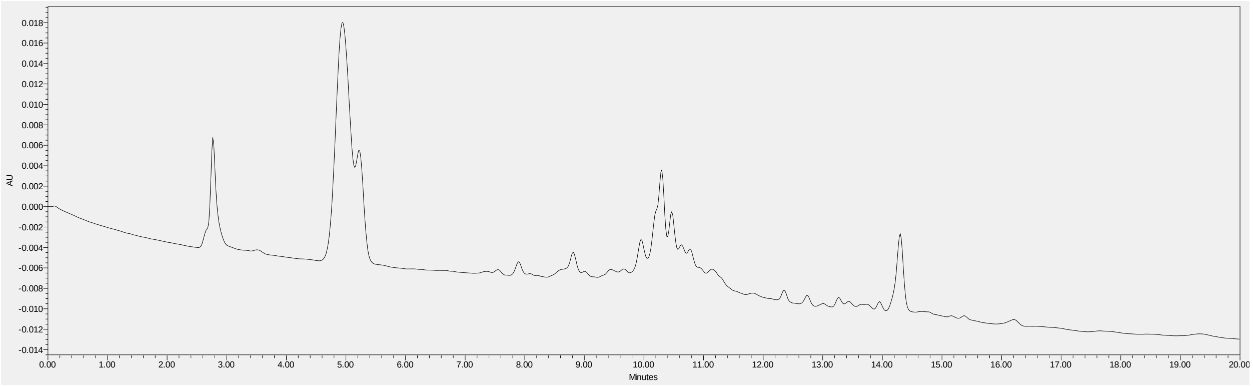

Setmelanotide—Hydrolytic Stability

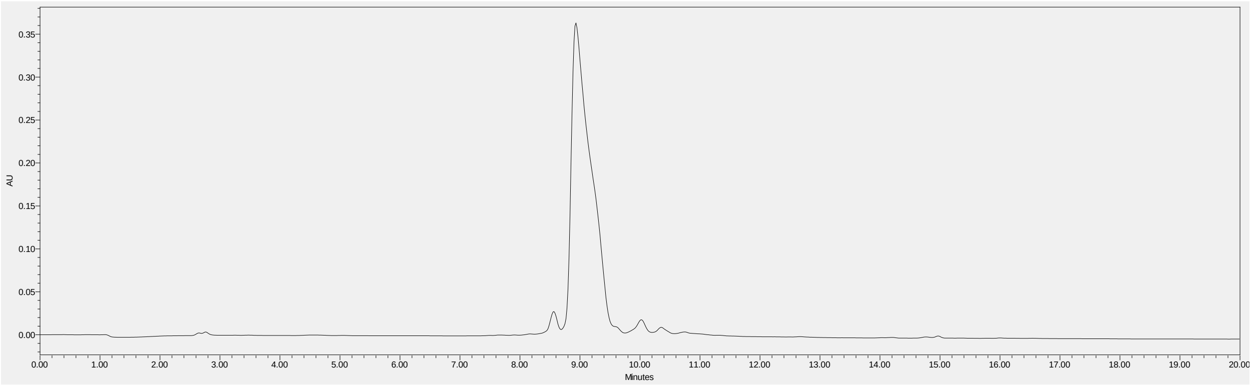

Mechanistic Studies

Radical Clock

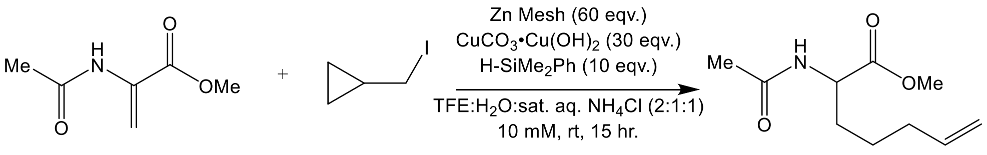

In a 2-dram vial containing a magnetic stir bar, methyl 2-acetamidoacrylate (10 mg, 70 µmol) was dissolved in 3.50 mL of TFE. $HSiMe_2Ph$ (95.2 mg, 700 µmol), (iodomethyl)cyclopropane (63.7 mg, 350 µmol), basic copper carbonate (464 mg, 2.1 mmol), H_2O (1.75 mL), and saturated aqueous NH_4Cl (1.75 mL) were added next. Finally, zinc mesh (273 mg, 4.2 mmol) was added.

The vial was capped with a septum, purged with N_2 for 3 minutes, sealed with parafilm, wrapped in aluminum foil, and allowed to stir at room temperature for 15 hours. The reaction was then filtered, concentrated under reduced pressure to ~3 mL, and extracted with ethyl acetate (3 x 5 mL). The combined organic layers were evaporated, dried under high vacuum, and analyzed by ^{1}H NMR using nitromethane as an internal standard. NMR yield: 26%.

Deuteration from Solvent

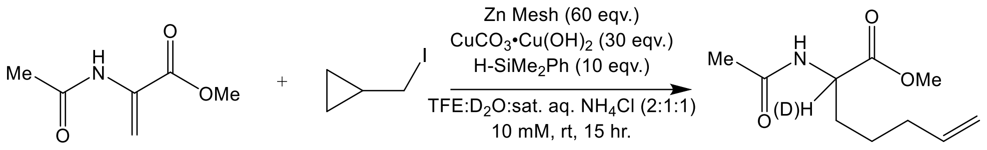

In a 2-dram vial containing a magnetic stir bar, methyl 2-acetamidoacrylate (10 mg, 70 µmol) was dissolve in 3.50 mL of TFE. $HSiMe_2Ph$ (95.2 mg, 700 µmol), (iodomethyl)cyclopropane (63.7 mg, 350 µmol), basic copper carbonate (464 mg, 2.1 mmol), D_2O (1.75 mL), and saturated aqueous NH_4Cl (prepared in D_2O, 1.75 mL) were added next. Finally, zinc mesh (273 mg, 4.2 mmol) was added. The vial was capped with a septum, purged with N_2 for 3 minutes, sealed with parafilm, wrapped in aluminum foil, and allowed to stir at room temperature for 15 hours. The reaction was then filtered, concentrated under reduced pressure to ~3 mL, and extracted with ethyl acetate (3 x 5 mL). The combined organic layers were evaporated, dried under high vacuum, and analyzed by ^{1}H NMR using nitromethane as an internal standard. NMR yield: 45%, *d*-content 65%.

Deuteration from Silane

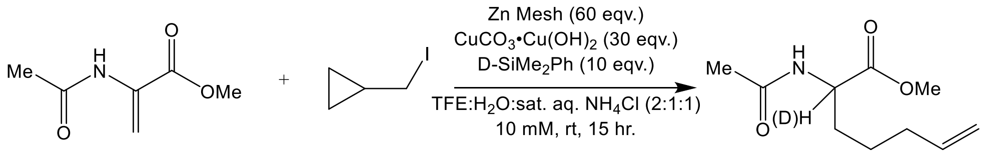

In a 2-dram vial containing a magnetic stir bar, methyl 2-acetamidoacrylate (10 mg, 70 µmol) was dissolve in 3.50 mL of TFE. $DSiMe_2Ph$ (95.2 mg, 700 µmol), (iodomethyl)cyclopropane (63.7 mg, 350 µmol), basic copper carbonate (464 mg, 2.1 mmol), H_2O (1.75 mL), and saturated aqueous NH_4Cl (1.75 mL) were added next. Finally, zinc mesh (273 mg, 4.2 mmol) was added. The vial was capped with a septum, purged with N_2 for 3 minutes, sealed with parafilm, wrapped in aluminum foil, and allowed to stir at room temperature for 15 hours. The reaction was then filtered, concentrated under reduced pressure to ~3 mL, and extracted with ethyl acetate (3 x 5 mL). The

combined organic layers were evaporated, dried under high vacuum, and analyzed by ^{1}H NMR using nitromethane as an internal standard. NMR yield: 31%, *d*-content 48%.

TEMPO Trapping

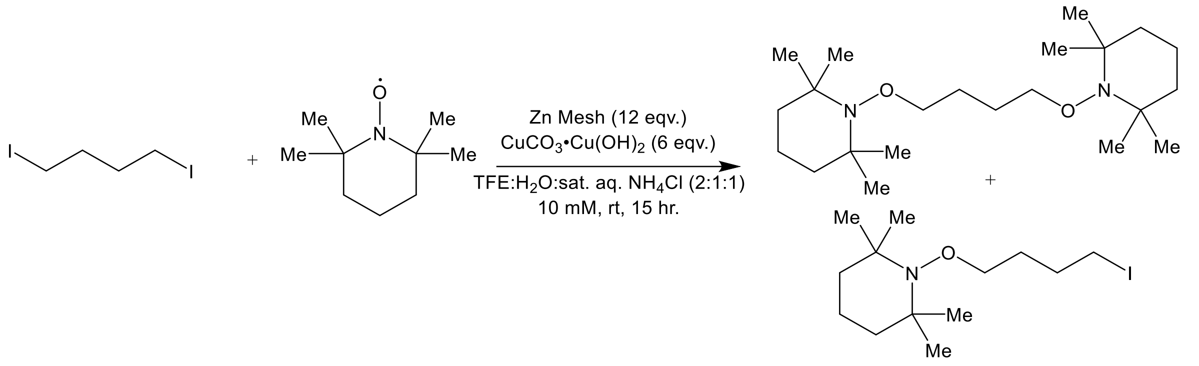

1,4-diiodobutane (5.0 mg, 16 µmol) and TEMPO (126 mg, 800 µmol) were added to a 1-dram vial charged with a magnetic stir bar. TFE (0.800 mL), H_2O (0.400 mL), saturated aqueous NH_4Cl (0.400 mL), and basic copper carbonate (20.4 mg, 96 µmol) were added next, followed by zinc mesh (12.5 mg, 192 µmol). The vial was capped with a septum, purged with N_2 for 3 minutes, sealed with parafilm, wrapped in aluminum foil, and stirred at room temperature for 15 hours. The reaction was then filtered and analyzed by LC-MS. Complete consumption of the di-iodide to mono-TEMPO and bis-TEMPO adducts was observed.

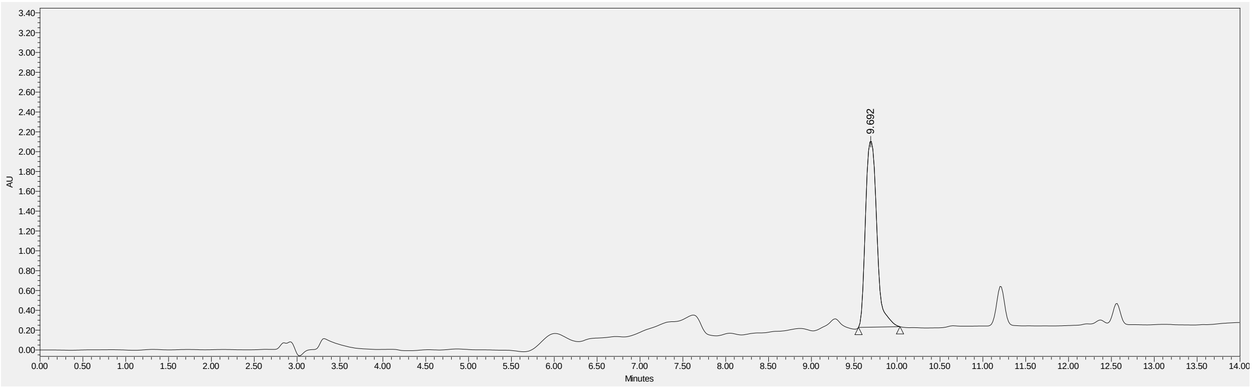

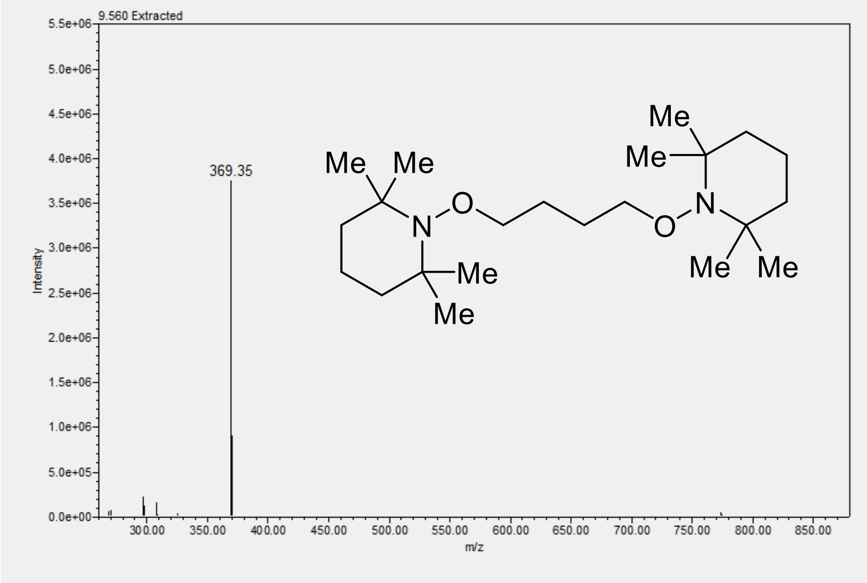
9.560 Extracted
369.35
Intensity
m/z
Me
N
O

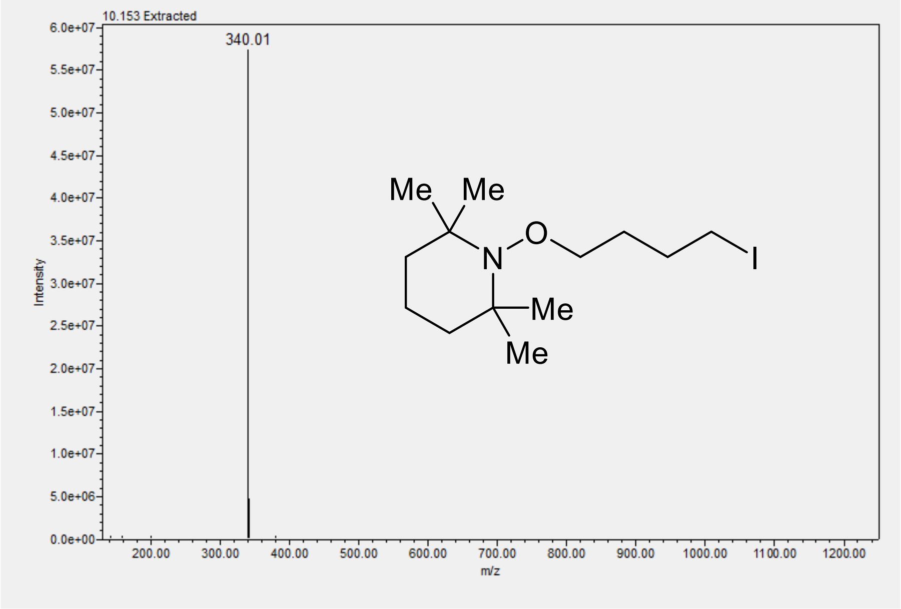
10.153 Extracted
340.01
Intensity
m/z
Me
N
O
I

Table S36. Control Experiements

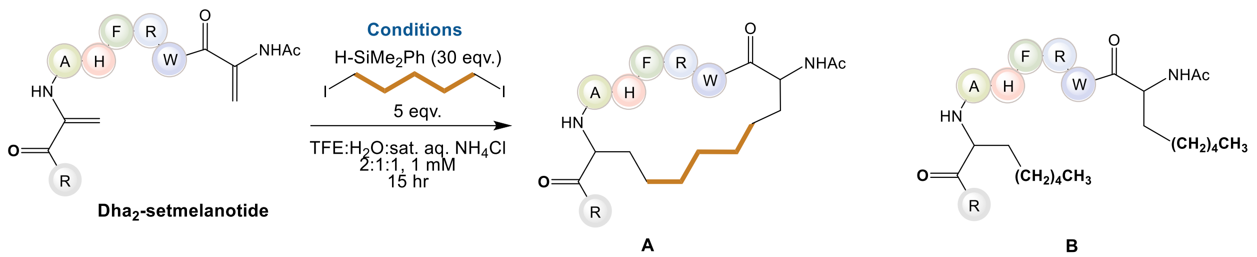

Conditions	% SM Remaining	% Conversion A	% Conversion B
$Zn(OTf)_2$,$CuCO_3 \cdot Cu(OH)_2$	26 %	0 %	0 %
$CuCO_3 \cdot Cu(OH)_2$ only	100 %	0 %	0 %
Cu powder only	100 %	0 %	0 %
Zn mesh, Cu(2-thiophenecarboxylate)	100 %	0 %	0 %

Attempted Generation of Silyl Radical

All reactions were performed using 70 µmol (10 mg) of methyl 2-acetamidoacrylate, (iodomethyl)cyclopropane (63.7 mg, 350 µmol) and $HSiMe_2Ph$ (107 µL, 700 µmol) at 10 mM under an N_2 atmosphere with the conditions listed below. After the indicated time, the reaction was filtered, and the solvent removed under reduced pressure. Reactions were then analyzed by ^1H NMR.

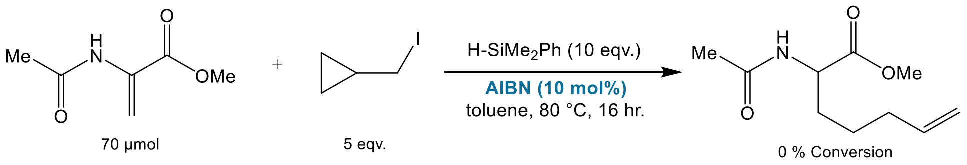

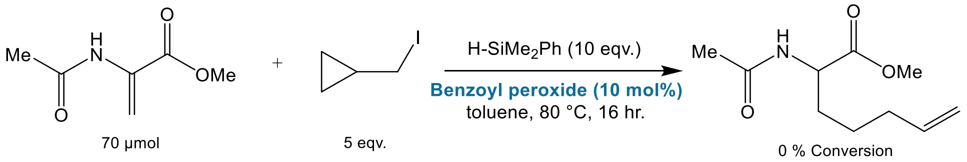

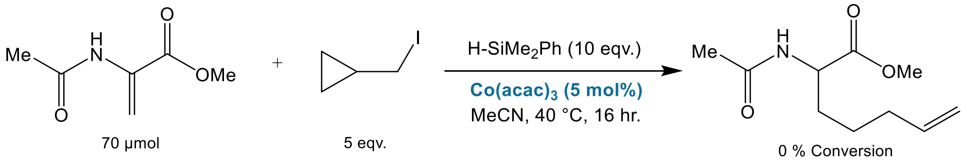

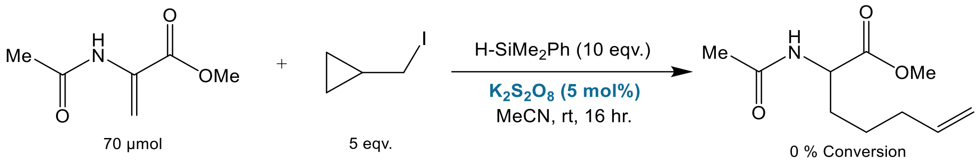
Me
H
N
O
O
OMe
70 µmol
+
I
5 eqv.
H-SiMe2Ph (10 eqv.)
K2S2O8 (5 mol%)
MeCN, rt, 16 hr.
0 % Conversion

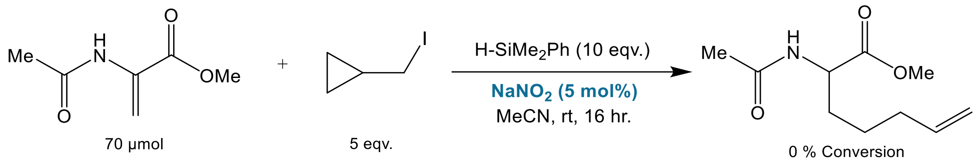
Me
H
N
O
O
OMe
70 µmol
+
I
5 eqv.
H-SiMe2Ph (10 eqv.)
NaNO2 (5 mol%)
MeCN, rt, 16 hr.
0 % Conversion

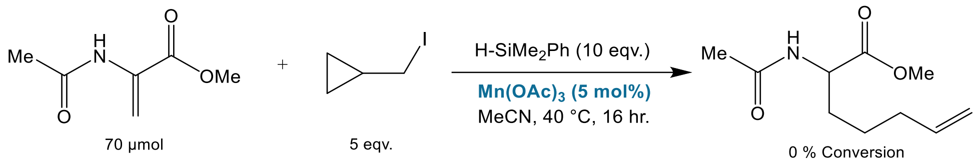
Me
H
N
O
O
OMe
70 µmol
+
I
5 eqv.
H-SiMe2Ph (10 eqv.)
Mn(OAc)3 (5 mol%)
MeCN, 40 °C, 16 hr.
0 % Conversion

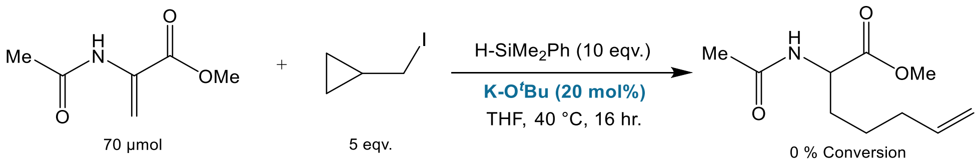
Me
H
N
O
O
OMe
70 µmol
+
I
5 eqv.
H-SiMe2Ph (10 eqv.)
K-OtBu (20 mol%)
THF, 40 °C, 16 hr.
0 % Conversion

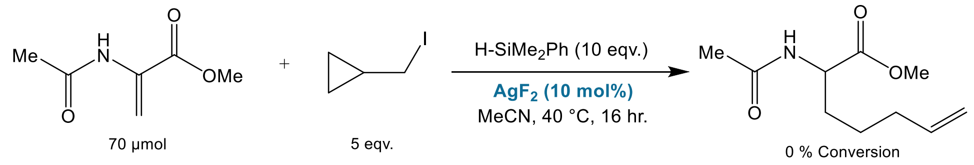
Me
H
N
O
O
OMe
70 µmol
+
I
5 eqv.
H-SiMe2Ph (10 eqv.)
AgF2 (10 mol%)
MeCN, 40 °C, 16 hr.
0 % Conversion

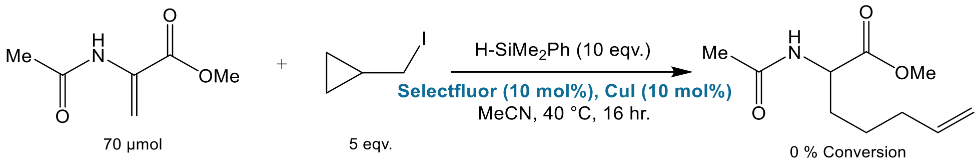
Me
H
N
O
O
OMe
70 µmol
+
I
5 eqv.
H-SiMe2Ph (10 eqv.)
Selectfluor (10 mol%), CuI (10 mol%)
MeCN, 40 °C, 16 hr.
0 % Conversion

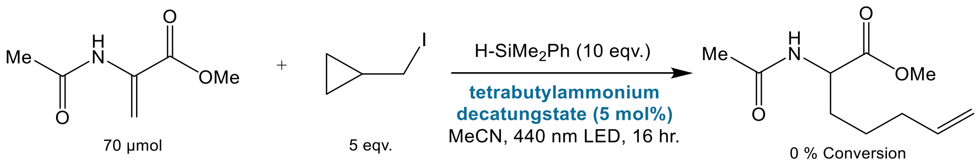
Me
H
N
O
O
OMe
70 µmol
+
I
5 eqv.
H-SiMe2Ph (10 eqv.)
tetrabutylammonium
decatungstate (5 mol%)
MeCN, 440 nm LED, 16 hr.
0 % Conversion

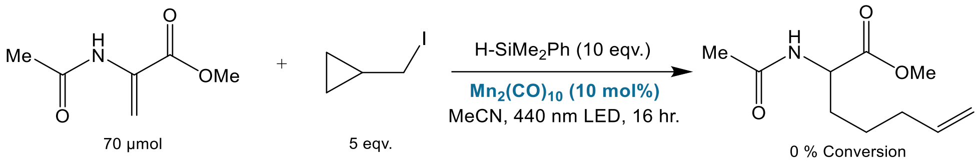

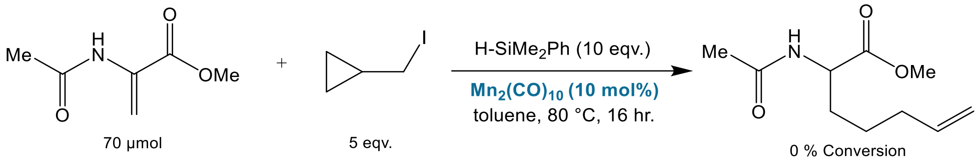

Hydrosilane Diastereoselectivity

All reactions were carried out using 1.0 mg Dha_2-setmelanotide, 5.0 equivalents of 1,5-diiodopentane and 30 equivalents of hydrosilane following the general procedure for peptide cyclization on pg. **S54**. Upon completion of the reaction, the mixture was filtered and analyzed by LC-MS. The diastereomeric ratio between cyclic products and the ratio of cyclic products to dialkylated products are both reported at 280 nm.

No silane

Cyclic: dialkylated 48:52

d.r. 1.9 : 1.7 : 1.0 : 1.8

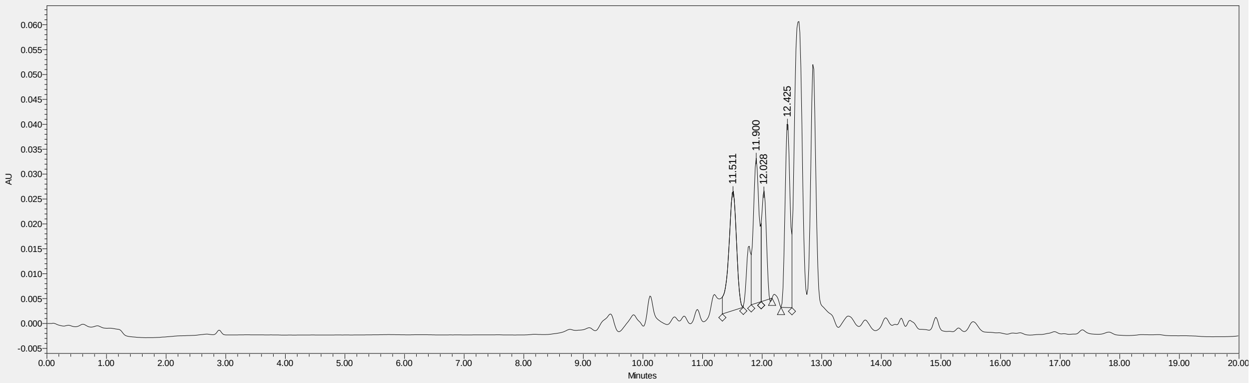

	Name	Retention Time	Area	% Area
1	Diast. 1	11.511	220124	29.15
2	Diast. 2	11.900	200851	26.59
3	Diast. 3	12.028	117971	15.62
4	Diast. 4	12.425	216298	28.64

HSiMe$_2$Ph

Cyclic: dialkylated 75:25

d.r. 14.5 : 6.0 : 3.6 : 1.0

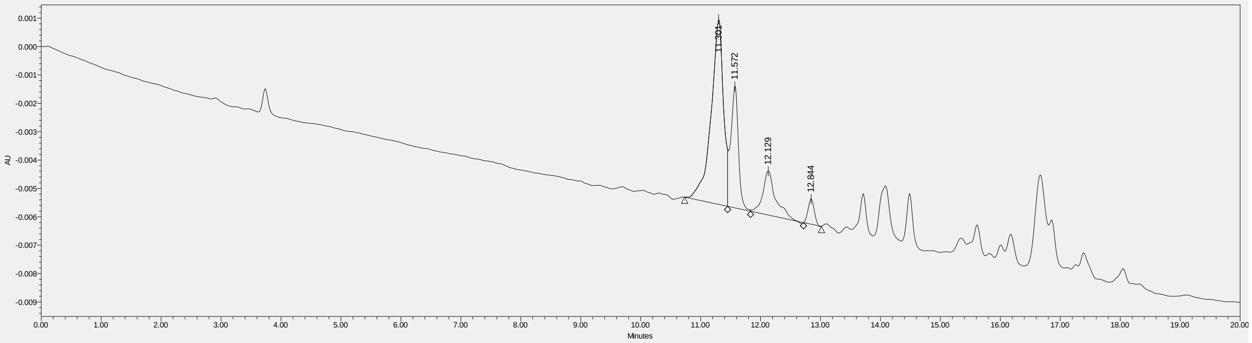

	Name	Retention Time	Area	% Area
1	Diast. 1	11.301	93356	57.72
2	Diast. 2	11.572	38671	23.91
3	Diast. 3	12.129	23282	14.39
4	Diast. 4	12.844	6434	3.98

HSiPh$_2$Me

Cyclic: dialkylated 40:60

d.r. 2.4 : 1.6 : 1.7 : 1.0

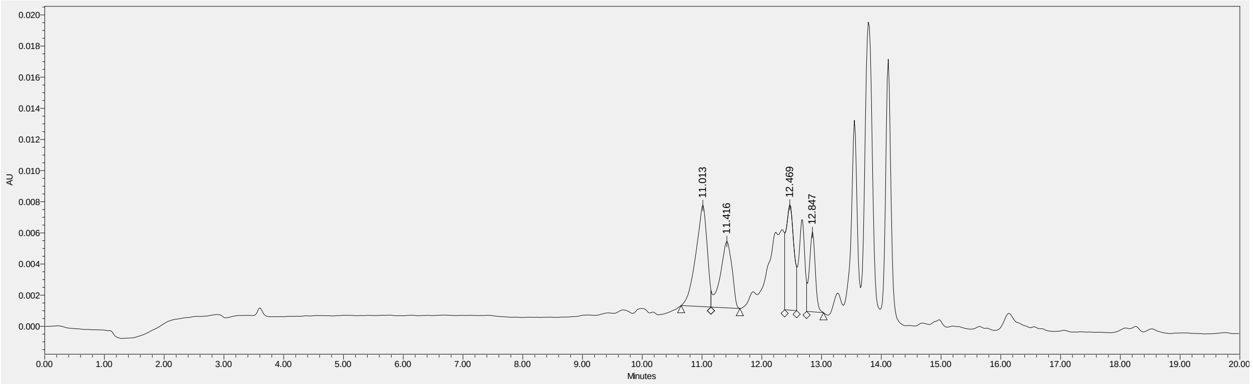

	Name	Retention Time	Area	% Area
1	Diast. 1	11.013	85083	35.32
2	Diast. 2	11.416	57738	23.97
3	Diast. 3	12.469	62263	25.85
4	Diast. 4	12.847	35779	14.85

HSi(Et)$_3$

Cyclic: dialkylated 67:34

d.r. 2.1 : 1.6 : 1.2 : 1.0

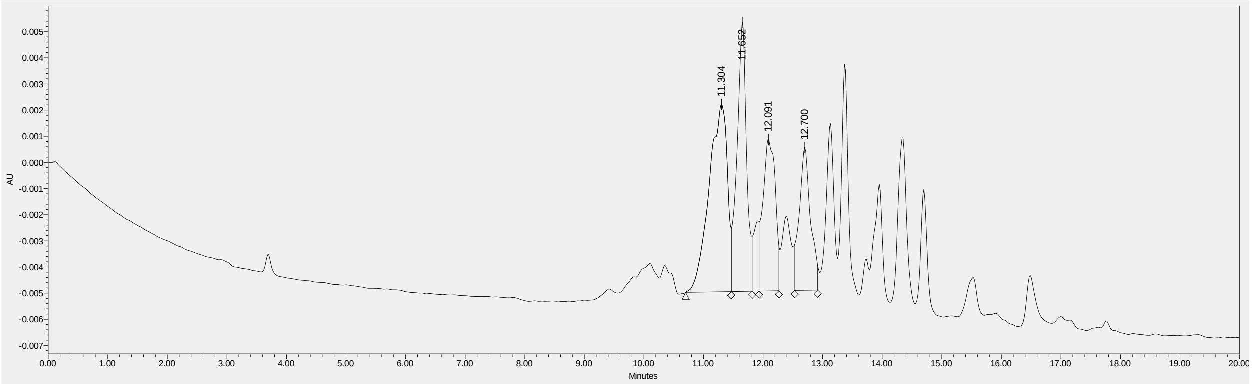

	Name	Retention Time	Area	% Area
1	Diast. 1	11.304	153197	36.12
2	Diast. 2	11.652	115401	27.21
3	Diast. 3	12.091	84017	19.81
4	Diast. 4	12.700	71520	16.86

$HSi(OEt)_3$

Cyclic: dialkylated 75:25

d.r. 1.9 : 1.0 : 1.3 : 1.0

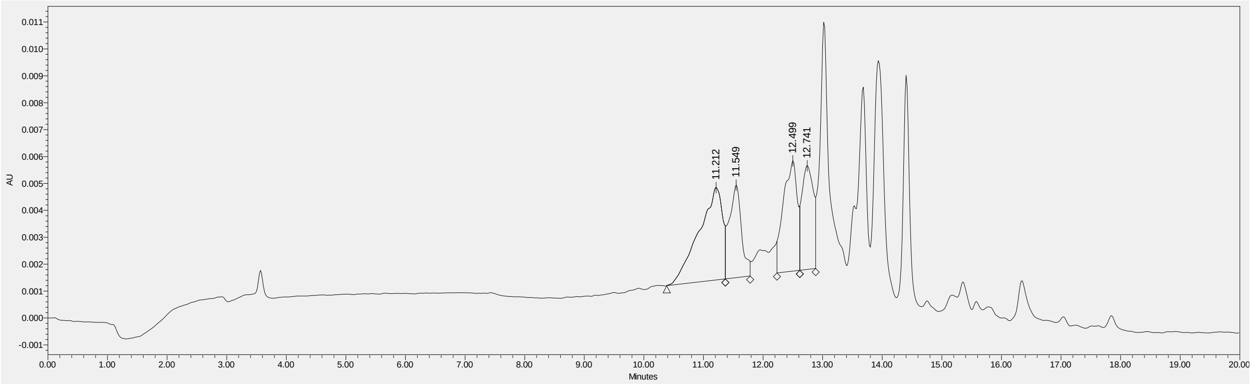

	Name	Retention Time	Area	% Area
1	Diast. 1	11.212	94357	35.95
2	Diast. 2	11.549	49697	18.93
3	Diast. 3	12.499	66532	25.35
4	Diast. 4	12.741	51887	19.77

$HSi(^nBu)_3$

Cyclic: dialkylated 74:26

d.r. 1.0 : 10.2 : 6.3

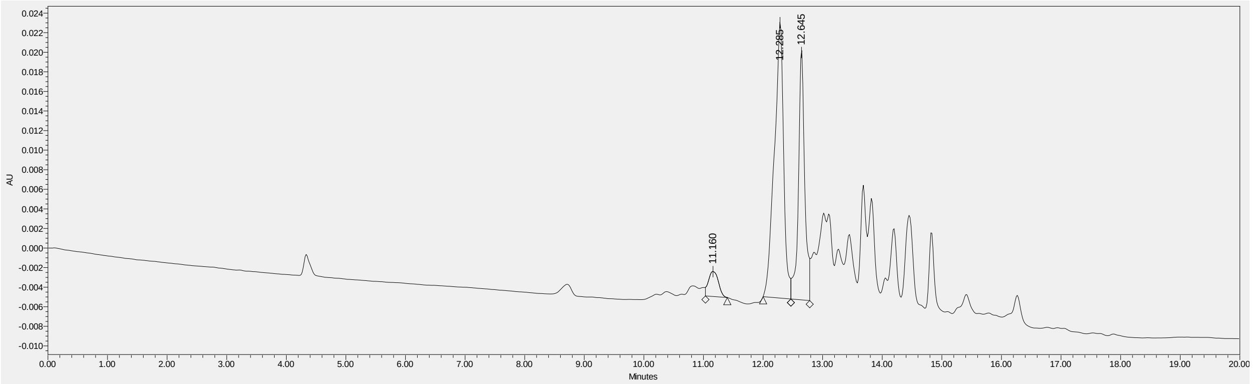

	Name	Retention Time	Area	% Area
1	Diast. 1	11.160	29857	5.71
2	Diast. 2	12.285	305896	58.45
3	Diast. 3	12.645	187595	35.85

HSi(^{i}Pr)$_3$

Cyclic: dialkylated 88:12

d.r. 3.1 : 2.2 : 1.1 : 1.0

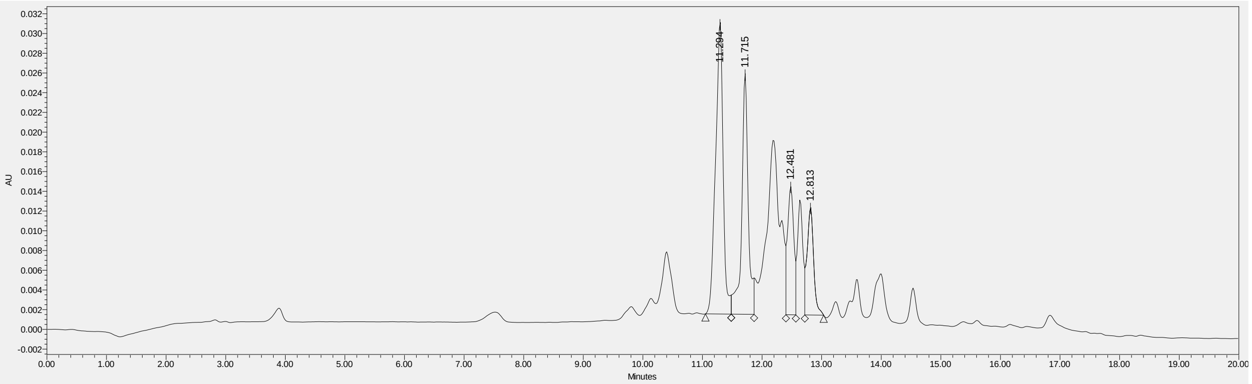

	Name	Retention Time	Area	% Area
1	Diast. 1	11.294	261360	41.75
2	Diast. 2	11.715	184975	29.55
3	Diast. 3	12.481	95468	15.25
4	Diast. 4	12.813	84142	13.44

HSiMe$_2$^{t}Bu

Cyclic: dialkylated 71:29

d.r. 6.1 : 1.8 : 1.0

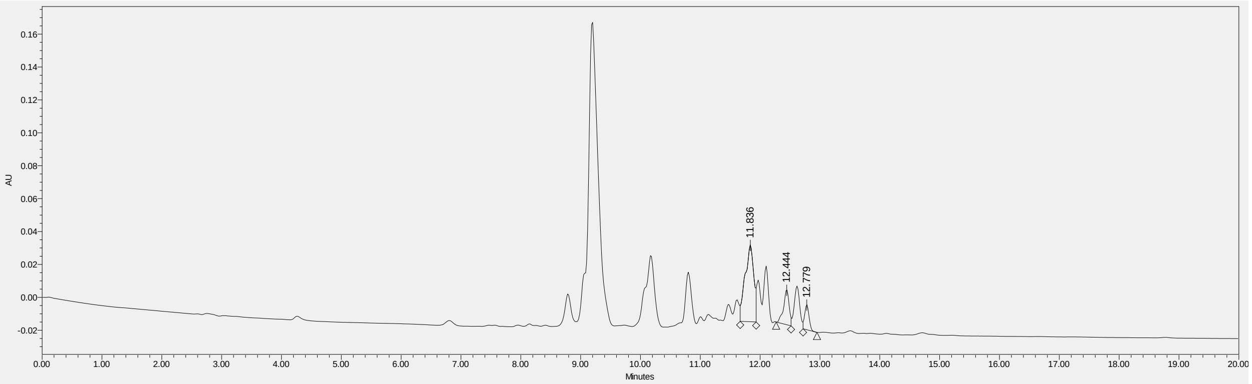

	Name	Retention Time	Area	% Area
1	Diast. 1	11.836	469664	68.82
2	Diast. 2	12.444	136042	19.93
3	Diast. 3	12.779	76736	11.24

$HSiMe_2{}^iPr$

Cyclic: dialkylated 70:30

d.r. 1.9 : 1.5 : 1.7 : 1.0

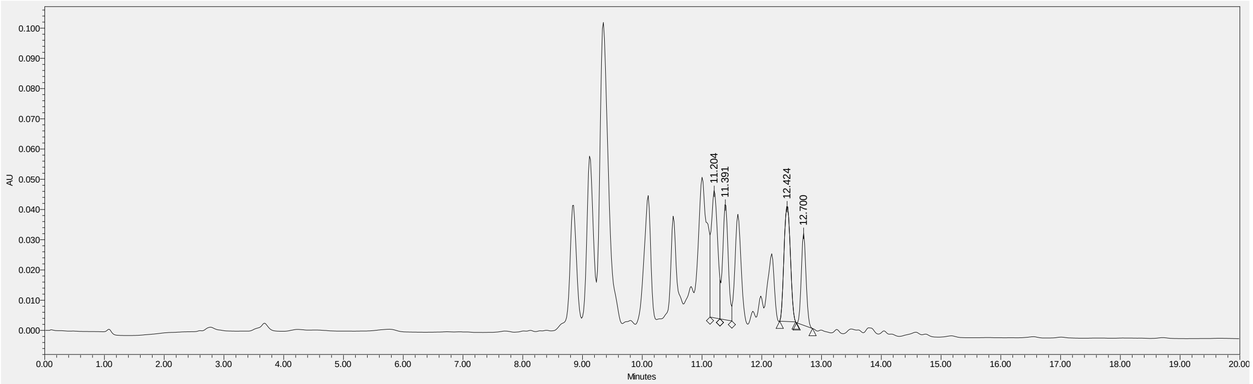

	Name	Retention Time	Area	% Area
1	Diast. 1	11.204	311808	30.79
2	Diast. 2	11.391	252456	24.93
3	Diast. 3	12.424	282123	27.86
4	Diast. 4	12.700	166210	16.41

DFT Calculations

(1) $H{-}SiPh_2Me \rightleftharpoons H\cdot + \cdot SiPh_2Me$ **BDE = 94 kcal mol^{-1}**

(2) $\cdot SiPh_2Me + I{-}CH_3 \rightleftharpoons I{-}SiPh_2Me + \cdot CH_3$ **ΔE = -17 kcal mol^{-1}**

(3) $H{-}SiEt_3 \rightleftharpoons H\cdot + \cdot SiEt_3$ **BDE = 96 kcal mol^{-1}**

(4) $\cdot SiEt_3 + I{-}CH_3 \rightleftharpoons I{-}SiEt_3 + \cdot CH_3$ **ΔE = -21 kcal mol^{-1}**

(5) $H{-}Si(OEt)_3 \rightleftharpoons H\cdot + \cdot Si(OEt)_3$ **BDE = 98 kcal mol^{-1}**

(6) $\cdot Si(OEt)_3 + I{-}CH_3 \rightleftharpoons I{-}Si(OEt)_3 + \cdot CH_3$ **ΔE = -19.5 kcal mol^{-1}**

(7) $H{-}Si(\textit{i}\text{-}Pr)_3 \rightleftharpoons H\cdot + \cdot Si(\textit{i}\text{-}Pr)_3$ **BDE = 95 kcal mol^{-1}**

(8) $\cdot Si(\textit{i}\text{-}Pr)_3 + I{-}CH_3 \rightleftharpoons I{-}Si(\textit{i}\text{-}Pr)_3 + \cdot CH_3$ **ΔE = -19 kcal mol^{-1}**

(9) $H{-}SiMe_2{}^tBu \rightleftharpoons H\cdot + \cdot SiMe_2{}^tBu$ **BDE = 96 kcal mol^{-1}**

(10) $\cdot SiMe_2{}^tBu + I{-}CH_3 \rightleftharpoons I{-}SiMe_2{}^tBu + \cdot CH_3$ **ΔE= -20 kcal mol^{-1}**

(11) $H{-}SiMe_2{}^iPr \rightleftharpoons H\cdot + \cdot SiMe_2{}^iPr$ **BDE = 96 kcal mol^{-1}**

(12) $\cdot SiMe_2{}^iPr + I{-}CH_3 \rightleftharpoons I{-}SiMe_2{}^iPr + \cdot CH_3$ **ΔH = -20 kcal mol^{-1}**

(13) $H{-}Si(\textit{n}\text{-}Bu)_3 \rightleftharpoons H\cdot + \cdot Si(\textit{n}\text{-}Bu)_3$ **BDE = 96 kcal mol^{-1}**

(14) $\cdot Si(\textit{n}\text{-}Bu)_3 + I{-}CH_3 \rightleftharpoons I{-}Si(\textit{n}\text{-}Bu)_3 + \cdot CH_3$ **ΔE = -20 kcal mol^{-1}**

(15) $H{-}SiMe_2Ph \rightleftharpoons H\cdot + \cdot SiMe_2Ph$ **BDE = 96 kcal mol^{-1}**

(16) $\cdot SiMe_2Ph + I{-}CH_3 \rightleftharpoons I{-}SiMe_2Ph + \cdot CH_3$ **ΔE = -21 kcal mol^{-1}**

(17)

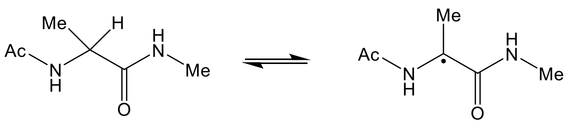

+ H· **BDE = 87 kcal mol^{-1}**

H—SiPh$_2$Me

SPARTAN '18 Quantum Mechanics Program: (x86/Darwin) build 1.4.4

Job type: Single point.
Method: RB3LYP
Basis set: 6-311+G**
Number of basis functions: 400
Number of electrons: 106

SCF model:
A restricted hybrid HF-DFT SCF calculation will be
performed using Pulay DIIS + Geometric Direct Minimization

SCF total energy: -793.4861369 hartrees

Reason for exit: Successful completion
Quantum Calculation CPU Time : 2:51.50
Quantum Calculation Wall Time: 2:56.11

SPARTAN '18 Properties Program: (x86/Darwin) build
1.4.4

Reason for exit: Successful completion
Properties CPU Time : 1.31
Properties Wall Time: 2.34

H·

SPARTAN '18 Quantum Mechanics Program: (x86/Darwin) build 1.4.4

Job type: Single point.
Method: UB3LYP
Basis set: 6-311+G**
Number of basis functions: 6
Number of electrons: 1 (1 unpaired)
Parallel Job: 4 threads

SCF model:
An unrestricted hybrid HF-DFT SCF calculation will be
performed using Pulay DIIS + Geometric Direct Minimization

SCF total energy: -0.5021559 hartrees

Reason for exit: Successful completion
Quantum Calculation CPU Time : 1.30
Quantum Calculation Wall Time: .54

SPARTAN '18 Properties Program: (x86/Darwin) build
1.4.4

Reason for exit: Successful completion
Properties CPU Time : .05
Properties Wall Time: .06

·$SiPh_2Me$

SPARTAN '18 MECHANICS PROGRAM: (x86/Darwin) build 1.4.4

Frequency Calculation

Adjusted 3 (out of 81) low frequency modes

Reason for exit: Successful completion
Mechanics CPU Time : .05
Mechanics Wall Time: .08

SPARTAN '18 Quantum Mechanics Program: (x86/Darwin) build 1.4.4

Job type: Geometry optimization.
Method: UB3LYP
Basis set: 6-311+G**
Number of basis functions: 394
Number of electrons: 105 (1 unpaired)
Parallel Job: 2 threads

SCF model:
An unrestricted hybrid HF-DFT SCF calculation will be
performed using Pulay DIIS + Geometric Direct Minimization

Optimization:

Step	Energy	Max Grad.	Max Dist.
1	-792.827899	0.012205	0.104731
2	-792.829682	0.003938	0.100633
3	-792.830000	0.002879	0.012892
4	-792.830051	0.002814	0.171615
5	-792.830728	0.003419	0.171729
6	-792.831408	0.005526	0.172065
7	-792.831824	0.008464	0.154985
8	-792.830880	0.012046	0.123174
9	-792.832227	0.008757	0.164322
10	-792.832472	0.011055	0.164839
11	-792.832589	0.012167	0.014268
12	-792.832655	0.011229	0.033140
13	-792.832827	0.008877	0.052281
14	-792.833179	0.008245	0.073364
15	-792.833712	0.005635	0.054196
16	-792.834103	0.002347	0.038727
17	-792.834234	0.001602	0.027223
18	-792.834250	0.000420	0.016591

Reason for exit: Successful completion
Quantum Calculation CPU Time : 2:09:51.04
Quantum Calculation Wall Time: 1:06:14.14

SPARTAN '18 Properties Program: (x86/Darwin) build 1.4.4

Reason for exit: Successful completion
Properties CPU Time : 1.47
Properties Wall Time: 2.51

I—CH$_3$

SPARTAN '18 Quantum Mechanics Program: (x86/Darwin) build 1.4.4

Job type: Single point.
Method: RB3LYP
Basis set: 6-311+G**
Number of basis functions: 102
Number of electrons: 62

SCF model:
A restricted hybrid HF-DFT SCF calculation will be
performed using Pulay DIIS + Geometric Direct Minimization

SCF total energy: -6959.4482180 hartrees

Reason for exit: Successful completion
Quantum Calculation CPU Time : 3.63
Quantum Calculation Wall Time: 6.69

SPARTAN '18 Properties Program: (x86/Darwin) build
1.4.4

Reason for exit: Successful completion
Properties CPU Time : .10
Properties Wall Time: 1.21

I—SiPh$_2$Me

SPARTAN '18 Quantum Mechanics Program: (x86/Darwin) build 1.4.4

Job type: Single point.
Method: RB3LYP
Basis set: 6-311+G**
Number of basis functions: 456
Number of electrons: 158

SCF model:
A restricted hybrid HF-DFT SCF calculation will be
performed using Pulay DIIS + Geometric Direct Minimization

SCF total energy: -7712.4542980 hartrees

Reason for exit: Successful completion
Quantum Calculation CPU Time : 4:42.03
Quantum Calculation Wall Time: 4:51.33

SPARTAN '18 Properties Program: (x86/Darwin) build
1.4.4

Reason for exit: Successful completion
Properties CPU Time : 2.14
Properties Wall Time: 3.27

$\cdot CH_3$

SPARTAN '18 Quantum Mechanics Program: (x86/Darwin) build 1.4.4

Job type: Single point.
Method: UB3LYP
Basis set: 6-311+G**
Number of basis functions: 40
Number of electrons: 9 (1 unpaired)

SCF model:
An unrestricted hybrid HF-DFT SCF calculation will be
performed using Pulay DIIS + Geometric Direct Minimization

SCF total energy: -39.8551658 hartrees

Reason for exit: Successful completion
Quantum Calculation CPU Time : .47
Quantum Calculation Wall Time: 3.28

SPARTAN '18 Properties Program: (x86/Darwin) build
1.4.4

Reason for exit: Successful completion
Properties CPU Time : .08
Properties Wall Time: .16

H—$SiEt_3$

SPARTAN '18 Quantum Mechanics Program: (x86/Darwin) build 1.4.4

Job type: Single point.
Method: RB3LYP
Basis set: 6-311+G**
Number of basis functions: 258
Number of electrons: 66
Parallel Job: 2 threads

SCF model:
A restricted hybrid HF-DFT SCF calculation will be
performed using Pulay DIIS + Geometric Direct Minimization

SCF total energy: -527.8902149 hartrees

Reason for exit: Successful completion
Quantum Calculation CPU Time : 42.02
Quantum Calculation Wall Time: 23.82

SPARTAN '18 Properties Program: (x86/Darwin) build
1.4.4

Reason for exit: Successful completion
Properties CPU Time : .49
Properties Wall Time: .47

·$SiEt_3$

SPARTAN '18 Quantum Mechanics Program: (x86/Darwin) build 1.4.4

Job type: Single point.
Method: UB3LYP
Basis set: 6-311+G**
Number of basis functions: 252
Number of electrons: 65 (1 unpaired)

SCF model:
An unrestricted hybrid HF-DFT SCF calculation will be
performed using Pulay DIIS + Geometric Direct Minimization

SCF total energy: -527.2356661 hartrees

Reason for exit: Successful completion
Quantum Calculation CPU Time : 53.60
Quantum Calculation Wall Time: 56.30

SPARTAN '18 Properties Program: (x86/Darwin) build
1.4.4

Reason for exit: Successful completion
Properties CPU Time : .53
Properties Wall Time: .52

I—$SiEt_3$

SPARTAN '18 Quantum Mechanics Program: (x86/Darwin) build 1.4.4

Job type: Single point.
Method: RB3LYP
Basis set: 6-311+G**
Number of basis functions: 314
Number of electrons: 118

SCF model:
A restricted hybrid HF-DFT SCF calculation will be
performed using Pulay DIIS + Geometric Direct Minimization

SCF total energy: -7446.8627172 hartrees

Reason for exit: Successful completion
Quantum Calculation CPU Time : 1:20.81
Quantum Calculation Wall Time: 1:25.76

SPARTAN '18 Properties Program: (x86/Darwin) build
1.4.4

Reason for exit: Successful completion
Properties CPU Time : .70
Properties Wall Time: 1.73

H—Si(OEt)$_3$

SPARTAN '18 Quantum Mechanics Program: (x86/Darwin) build 1.4.4

Job type: Single point.
Method: RB3LYP
Basis set: 6-311+G**
Number of basis functions: 324
Number of electrons: 90

SCF model:
A restricted hybrid HF-DFT SCF calculation will be performed using Pulay DIIS + Geometric Direct Minimization

SCF total energy: -753.7302635 hartrees

Reason for exit: Successful completion
Quantum Calculation CPU Time : 1:20.53
Quantum Calculation Wall Time: 1:24.18

SPARTAN '18 Properties Program: (x86/Darwin) build 1.4.4

Reason for exit: Successful completion
Properties CPU Time : .77
Properties Wall Time: 1.75

·Si(OEt)$_3$

SPARTAN '18 Quantum Mechanics Program: (x86/Darwin) build 1.4.4

Job type: Single point.
Method: UB3LYP
Basis set: 6-311+G**
Number of basis functions: 318
Number of electrons: 89 (1 unpaired)

SCF model:
An unrestricted hybrid HF-DFT SCF calculation will be performed using Pulay DIIS + Geometric Direct Minimization

SCF total energy: -753.0713507 hartrees

Reason for exit: Successful completion
Quantum Calculation CPU Time : 1:36.97
Quantum Calculation Wall Time: 1:40.38

SPARTAN '18 Properties Program: (x86/Darwin) build 1.4.4

Reason for exit: Successful completion
Properties CPU Time : .88
Properties Wall Time: .86

I—Si(OEt)$_3$

SPARTAN '18 MECHANICS PROGRAM: (x86/Darwin) build 1.4.4

Frequency Calculation

Adjusted 6 (out of 78) low frequency modes

Reason for exit: Successful completion
Mechanics CPU Time : .04
Mechanics Wall Time: 1.05

SPARTAN '18 Quantum Mechanics Program: (x86/Darwin) build 1.4.4

Job type: Geometry optimization.
Method: RB3LYP
Basis set: 6-311+G**
Number of basis functions: 380
Number of electrons: 142

SCF model:
A restricted hybrid HF-DFT SCF calculation will be
performed using Pulay DIIS + Geometric Direct Minimization

Optimization:

Step	Energy	Max Grad.	Max Dist.
1	-7672.685891	0.014975	0.090000
2	-7672.691422	0.011047	0.083011
3	-7672.693004	0.007461	0.063900
4	-7672.693582	0.003525	0.073688
5	-7672.693871	0.002006	0.079419
6	-7672.694046	0.001315	0.131316
7	-7672.694186	0.002758	0.117351
8	-7672.694389	0.002365	0.093604
9	-7672.694742	0.001799	0.105993
10	-7672.694861	0.001317	0.109263
11	-7672.695049	0.000988	0.117801
12	-7672.695133	0.002181	0.123792
13	-7672.695122	0.002740	0.045324
14	-7672.695233	0.001309	0.040610
15	-7672.695272	0.000801	0.036906
16	-7672.695304	0.000538	0.031787
17	-7672.695319	0.000419	0.030831

Reason for exit: Successful completion
Quantum Calculation CPU Time : 1:13:00.10
Quantum Calculation Wall Time: 1:15:51.77

SPARTAN '18 Properties Program: (x86/Darwin) build 1.4.4

Reason for exit: Successful completion
Properties CPU Time : 1.21
Properties Wall Time: 2.88

H—Si(*i*-Pr)$_3$

SPARTAN '18 Quantum Mechanics Program: (x86/Darwin) build 1.4.4

Job type: Single point.
Method: RB3LYP
Basis set: 6-311+G**
Number of basis functions: 360
Number of electrons: 90

SCF model:
A restricted hybrid HF-DFT SCF calculation will be
performed using Pulay DIIS + Geometric Direct Minimization

SCF total energy: -645.8512200 hartrees

Reason for exit: Successful completion
Quantum Calculation CPU Time : 2:11.06
Quantum Calculation Wall Time: 2:14.92

SPARTAN '18 Properties Program: (x86/Darwin) build
1.4.4

Reason for exit: Successful completion
Properties CPU Time : 1.09
Properties Wall Time: 2.10

·Si(*i*-Pr)$_3$

SPARTAN '18 MECHANICS PROGRAM: (x86/Darwin) build 1.4.4

Frequency Calculation

Adjusted 2 (out of 93) low frequency modes

Reason for exit: Successful completion
Mechanics CPU Time : .05
Mechanics Wall Time: .05

SPARTAN '18 Quantum Mechanics Program: (x86/Darwin) build 1.4.4

Job type: Geometry optimization.
Method: UB3LYP
Basis set: 6-311+G**
Number of basis functions: 354
Number of electrons: 89 (1 unpaired)

SCF model:
An unrestricted hybrid HF-DFT SCF calculation will be
performed using Pulay DIIS + Geometric Direct Minimization

Optimization:

Step	Energy	Max Grad.	Max Dist.
1	-645.192830	0.021979	0.119432
2	-645.197004	0.006114	0.092618
3	-645.197446	0.001956	0.011361
4	-645.197507	0.001701	0.040240
5	-645.197649	0.000515	0.006131
6	-645.197655	0.000165	0.003410

Reason for exit: Successful completion
Quantum Calculation CPU Time : 55:08.46
Quantum Calculation Wall Time: 56:16.83

SPARTAN '18 Properties Program: (x86/Darwin) build 1.4.4

Reason for exit: Successful completion
Properties CPU Time : 1.14
Properties Wall Time: 2.18

I—Si(i-Pr)$_3$

SPARTAN '18 MECHANICS PROGRAM: (x86/Darwin) build 1.4.4

Frequency Calculation

Adjusted 2 (out of 96) low frequency modes

Reason for exit: Successful completion
Mechanics CPU Time : .05
Mechanics Wall Time: .09

SPARTAN '18 Quantum Mechanics Program: (x86/Darwin) build 1.4.4

Job type: Geometry optimization.
Method: RB3LYP
Basis set: 6-311+G**
Number of basis functions: 416
Number of electrons: 142

SCF model:
A restricted hybrid HF-DFT SCF calculation will be
performed using Pulay DIIS + Geometric Direct Minimization

Optimization:

Step	Energy	Max Grad.	Max Dist.
1	-7564.817363	0.009851	0.145278
2	-7564.819833	0.004318	0.081399
3	-7564.820147	0.002768	0.008635
4	-7564.820219	0.002577	0.151496
5	-7564.820589	0.002590	0.082881
6	-7564.820695	0.001081	0.031572
7	-7564.820755	0.000522	0.053686
8	-7564.820774	0.000331	0.025326

Reason for exit: Successful completion
Quantum Calculation CPU Time : 1:25:11.11
Quantum Calculation Wall Time: 1:27:58.82

SPARTAN '18 Properties Program: (x86/Darwin) build 1.4.4

Reason for exit: Successful completion
Properties CPU Time : 1.86
Properties Wall Time: 3.28

H—SiMe$_2$^{t}Bu

SPARTAN '18 Quantum Mechanics Program: (x86/Darwin) build 1.4.4

Job type: Single point.
Method: RB3LYP
Basis set: 6-311+G**
Number of basis functions: 258
Number of electrons: 66

SCF model:
A restricted hybrid HF-DFT SCF calculation will be
performed using Pulay DIIS + Geometric Direct Minimization

SCF total energy: -527.8962082 hartrees

Reason for exit: Successful completion
Quantum Calculation CPU Time : 48.39
Quantum Calculation Wall Time: 51.20

SPARTAN '18 Properties Program: (x86/Darwin) build
1.4.4

Reason for exit: Successful completion
Properties CPU Time : .48
Properties Wall Time: .46

$\cdot SiMe_2{}^tBu$

SPARTAN '18 Quantum Mechanics Program: (x86/Darwin) build 1.4.4

Job type: Single point.
Method: UB3LYP
Basis set: 6-311+G**
Number of basis functions: 252
Number of electrons: 65 (1 unpaired)

SCF model:
An unrestricted hybrid HF-DFT SCF calculation will be
performed using Pulay DIIS + Geometric Direct Minimization

SCF total energy: -527.2416615 hartrees

Reason for exit: Successful completion
Quantum Calculation CPU Time : 57.81
Quantum Calculation Wall Time: 1:01.00

SPARTAN '18 Properties Program: (x86/Darwin) build
1.4.4

Reason for exit: Successful completion
Properties CPU Time : .53
Properties Wall Time: .61

$I—SiMe_2{}^tBu$

SPARTAN '18 Quantum Mechanics Program: (x86/Darwin) build 1.4.4

Job type: Single point.
Method: RB3LYP
Basis set: 6-311+G**
Number of basis functions: 314
Number of electrons: 118

SCF model:
A restricted hybrid HF-DFT SCF calculation will be
performed using Pulay DIIS + Geometric Direct Minimization

SCF total energy: -7446.8662796 hartrees

Reason for exit: Successful completion
Quantum Calculation CPU Time : 1:48.01
Quantum Calculation Wall Time: 1:54.47

SPARTAN '18 Properties Program: (x86/Darwin) build
1.4.4

Reason for exit: Successful completion
Properties CPU Time : .80
Properties Wall Time: 1.80

H—SiMe$_2$^{i}Pr

SPARTAN '18 Quantum Mechanics Program: (x86/Darwin) build 1.4.4

Job type: Single point.
Method: RB3LYP
Basis set: 6-311+G**
Number of basis functions: 224
Number of electrons: 58

SCF model:
A restricted hybrid HF-DFT SCF calculation will be
performed using Pulay DIIS + Geometric Direct Minimization

SCF total energy: -488.5739318 hartrees

Reason for exit: Successful completion
Quantum Calculation CPU Time : 30.25
Quantum Calculation Wall Time: 32.83

SPARTAN '18 Properties Program: (x86/Darwin) build
1.4.4

Reason for exit: Successful completion
Properties CPU Time : .38
Properties Wall Time: .42

·SiMe$_2$^{i}Pr

SPARTAN '18 Quantum Mechanics Program: (x86/Darwin) build 1.4.4

Job type: Single point.
Method: UB3LYP
Basis set: 6-311+G**
Number of basis functions: 218
Number of electrons: 57 (1 unpaired)

SCF model:
An unrestricted hybrid HF-DFT SCF calculation will be
performed using Pulay DIIS + Geometric Direct Minimization

SCF total energy: -487.9193825 hartrees

Reason for exit: Successful completion
Quantum Calculation CPU Time : 36.19
Quantum Calculation Wall Time: 38.74

SPARTAN '18 Properties Program: (x86/Darwin) build
1.4.4

Reason for exit: Successful completion
Properties CPU Time : .40
Properties Wall Time: .43

I—SiMe$_2$^{i}Pr

SPARTAN '18 Quantum Mechanics Program: (x86/Darwin) build 1.4.4

Job type: Single point.
Method: RB3LYP
Basis set: 6-311+G**
Number of basis functions: 280
Number of electrons: 110

SCF model:
A restricted hybrid HF-DFT SCF calculation will be
performed using Pulay DIIS + Geometric Direct Minimization

SCF total energy: -7407.5439905 hartrees

Reason for exit: Successful completion
Quantum Calculation CPU Time : 1:15.63
Quantum Calculation Wall Time: 1:21.51

SPARTAN '18 Properties Program: (x86/Darwin) build
1.4.4

Reason for exit: Successful completion
Properties CPU Time : .68
Properties Wall Time: 1.73

H—Si(*n*-Bu)$_3$

SPARTAN '18 Quantum Mechanics Program: (x86/Darwin) build 1.4.4

Job type: Single point.
Method: RB3LYP
Basis set: 6-311+G**
Number of basis functions: 462
Number of electrons: 114

SCF model:
A restricted hybrid HF-DFT SCF calculation will be
performed using Pulay DIIS + Geometric Direct Minimization

SCF total energy: -763.8355267 hartrees

Reason for exit: Successful completion
Quantum Calculation CPU Time : 3:29.45
Quantum Calculation Wall Time: 3:36.63

SPARTAN '18 Properties Program: (x86/Darwin) build
1.4.4

Reason for exit: Successful completion
Properties CPU Time : 2.07
Properties Wall Time: 3.17

·Si(*n*-Bu)$_3$

SPARTAN '18 Quantum Mechanics Program: (x86/Darwin) build 1.4.4

Job type: Single point.
Method: UB3LYP
Basis set: 6-311+G**
Number of basis functions: 456
Number of electrons: 113 (1 unpaired)
Parallel Job: 2 threads

SCF model:
An unrestricted hybrid HF-DFT SCF calculation will be
performed using Pulay DIIS + Geometric Direct Minimization

SCF total energy: -763.1810586 hartrees

Reason for exit: Successful completion
Quantum Calculation CPU Time : 7:48.27
Quantum Calculation Wall Time: 4:00.90

SPARTAN '18 Properties Program: (x86/Darwin) build
1.4.4

Reason for exit: Successful completion
Properties CPU Time : 2.25
Properties Wall Time: 3.25

I—Si(*n*-Bu)$_3$

SPARTAN '18 Quantum Mechanics Program: (x86/Darwin) build 1.4.4

Job type: Single point.
Method: RB3LYP
Basis set: 6-311+G**
Number of basis functions: 518
Number of electrons: 166
Parallel Job: 4 threads

SCF model:
A restricted hybrid HF-DFT SCF calculation will be
performed using Pulay DIIS + Geometric Direct Minimization

SCF total energy: -7682.8077984 hartrees

Reason for exit: Successful completion
Quantum Calculation CPU Time : 5:33.88
Quantum Calculation Wall Time: 1:37.60

SPARTAN '18 Properties Program: (x86/Darwin) build
1.4.4

Reason for exit: Successful completion
Properties CPU Time : 2.51
Properties Wall Time: 3.61

H—SiMe$_2$Ph

SPARTAN '18 Quantum Mechanics Program: (x86/Darwin) build 1.4.4

Job type: Single point.
Method: RB3LYP
Basis set: 6-311+G**
Number of basis functions: 278
Number of electrons: 74

SCF model:
A restricted hybrid HF-DFT SCF calculation will be
performed using Pulay DIIS + Geometric Direct Minimization

SCF total energy: -601.7094689 hartrees

Reason for exit: Successful completion
Quantum Calculation CPU Time : 1:03.54
Quantum Calculation Wall Time: 1:09.85

SPARTAN '18 Properties Program: (x86/Darwin) build
1.4.4

Reason for exit: Successful completion
Properties CPU Time : .65
Properties Wall Time: 1.90

$\cdot SiMe_2Ph$

SPARTAN '18 Quantum Mechanics Program: (x86/Darwin) build 1.4.4

Job type: Single point.
Method: UB3LYP
Basis set: 6-311+G**
Number of basis functions: 272
Number of electrons: 73 (1 unpaired)

SCF model:
An unrestricted hybrid HF-DFT SCF calculation will be
performed using Pulay DIIS + Geometric Direct Minimization

SCF total energy: -601.0536786 hartrees

Reason for exit: Successful completion
Quantum Calculation CPU Time : 2:43.76
Quantum Calculation Wall Time: 2:52.84

SPARTAN '18 Properties Program: (x86/Darwin) build
1.4.4

Reason for exit: Successful completion
Properties CPU Time : .66
Properties Wall Time: 2.07

$I—SiMe_2Ph$

SPARTAN '18 Quantum Mechanics Program: (x86/Darwin) build 1.4.4

Job type: Single point.
Method: RB3LYP
Basis set: 6-311+G**
Number of basis functions: 334
Number of electrons: 126

SCF model:
A restricted hybrid HF-DFT SCF calculation will be
performed using Pulay DIIS + Geometric Direct Minimization

SCF total energy: -7520.6783979 hartrees

Reason for exit: Successful completion
Quantum Calculation CPU Time : 1:42.16
Quantum Calculation Wall Time: 1:48.69

SPARTAN '18 Properties Program: (x86/Darwin) build
1.4.4

Reason for exit: Successful completion
Properties CPU Time : .84
Properties Wall Time: 2.28

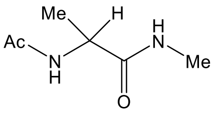

SPARTAN '18 Quantum Mechanics Program: (x86/Darwin) build 1.4.4

Job type: Single point.
Method: RB3LYP
Basis set: 6-311+G**
Number of basis functions: 292
Number of electrons: 78
Parallel Job: 4 threads

SCF model:
A restricted hybrid HF-DFT SCF calculation will be
performed using Pulay DIIS + Geometric Direct Minimization

SCF total energy: -496.0012350 hartrees

Reason for exit: Successful completion
Quantum Calculation CPU Time : 1:04.31
Quantum Calculation Wall Time: 18.58

SPARTAN '18 Properties Program: (x86/Darwin) build
1.4.4

Reason for exit: Successful completion
Properties CPU Time : .55
Properties Wall Time: 1.54

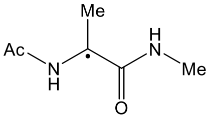

SPARTAN '18 Quantum Mechanics Program: (x86/Darwin) build 1.4.4

Job type: Single point.
Method: UB3LYP
Basis set: 6-311+G**
Number of basis functions: 286
Number of electrons: 77 (1 unpaired)
Parallel Job: 4 threads

SCF model:
An unrestricted hybrid HF-DFT SCF calculation will be
performed using Pulay DIIS + Geometric Direct Minimization

SCF total energy: -495.3599378 hartrees

Reason for exit: Successful completion
Quantum Calculation CPU Time : 2:27.28
Quantum Calculation Wall Time: 46.61

SPARTAN '18 Properties Program: (x86/Darwin) build 1.4.4

Reason for exit: Successful completion
Properties CPU Time : .63
Properties Wall Time: 1.78

NMR Spectra

^{1}H NMR

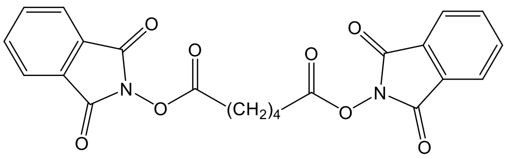

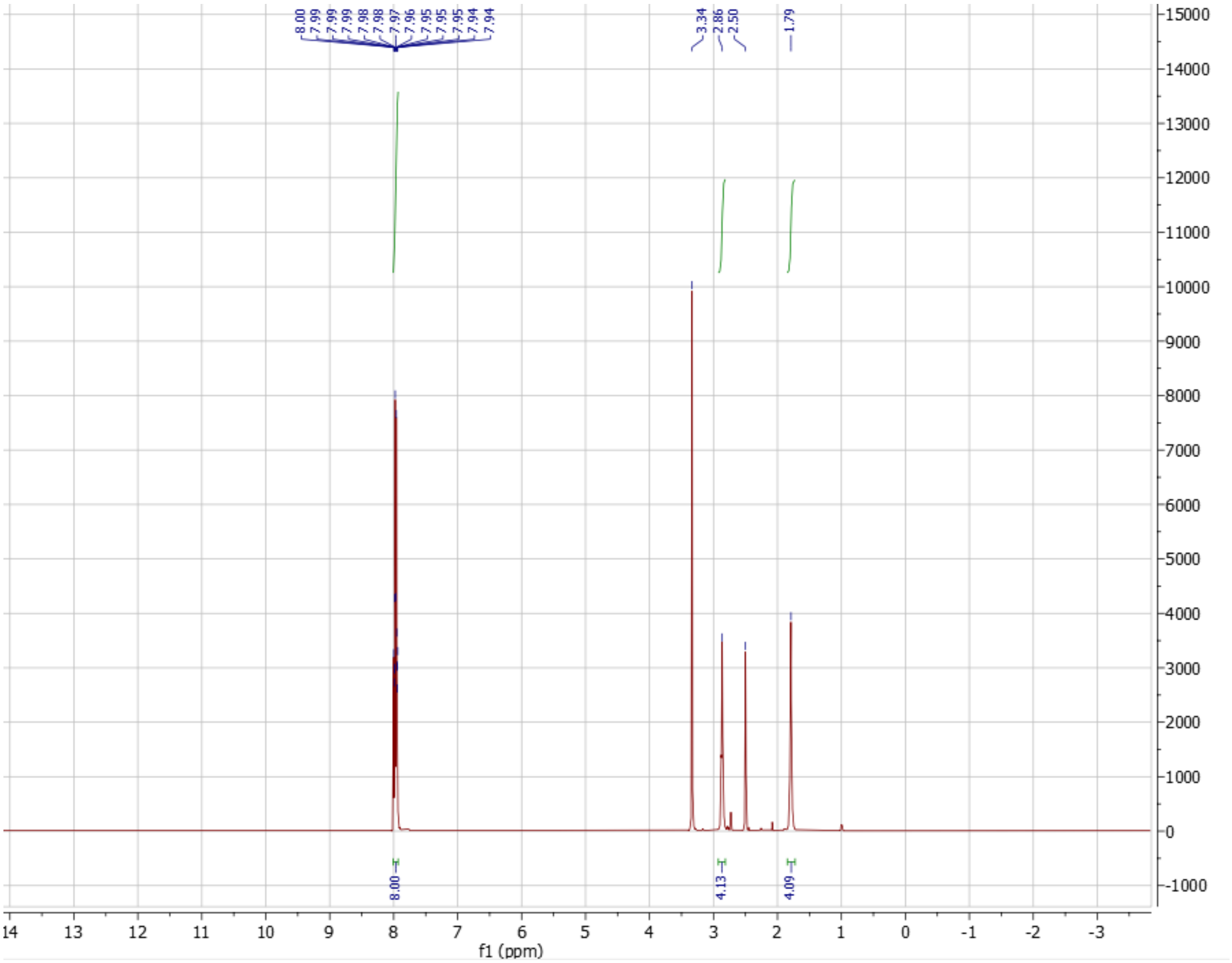

^{13}C NMR

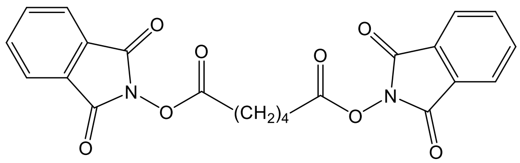
O
N
O
O
(CH2)4
O
O
N
O
O

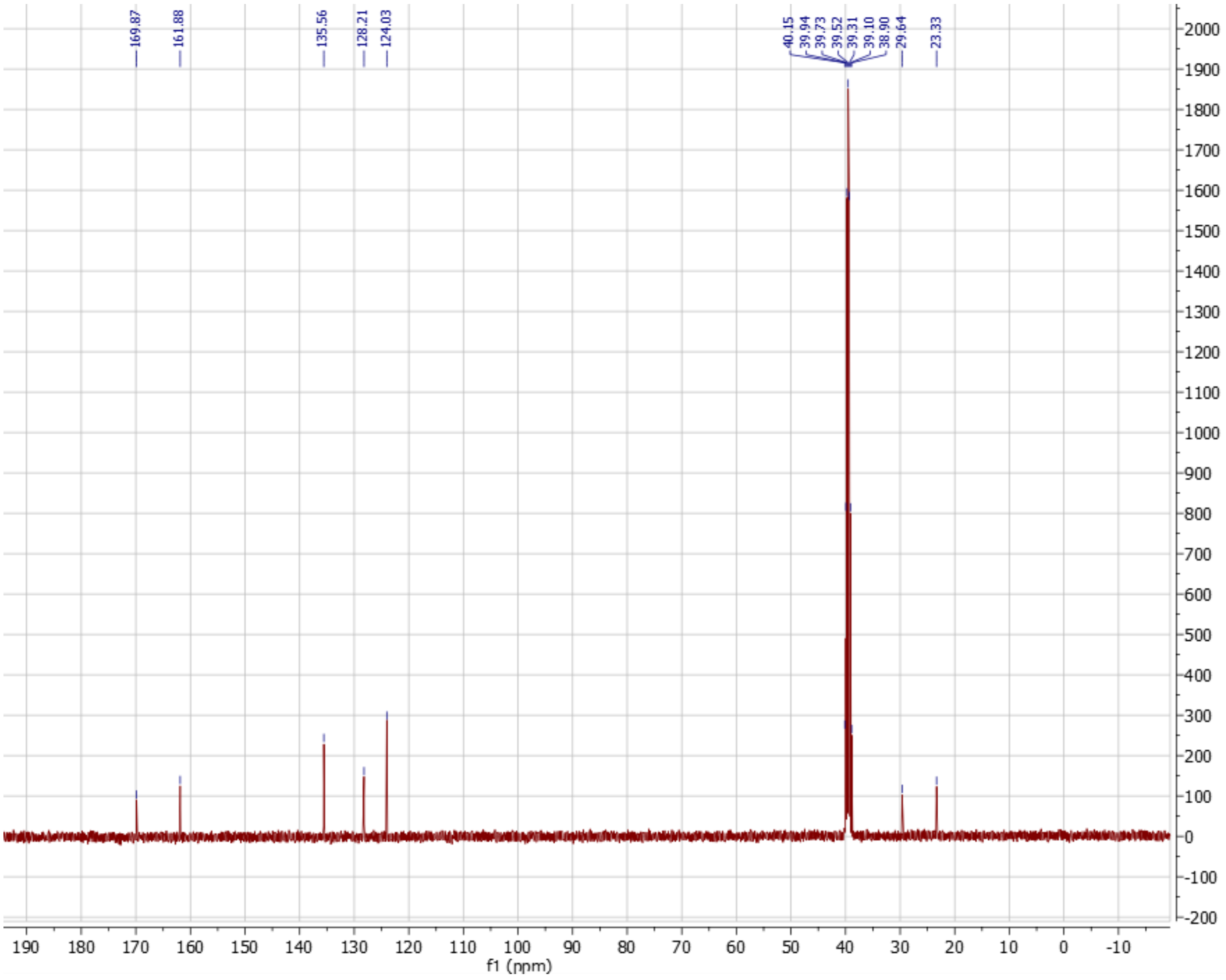
169.87
161.88
135.56
128.21
124.03
40.15
39.94
39.73
39.52
39.31
39.10
38.90
29.64
23.33
f1 (ppm)

^{2}H NMR

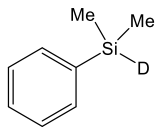
Me
Me
Si
D

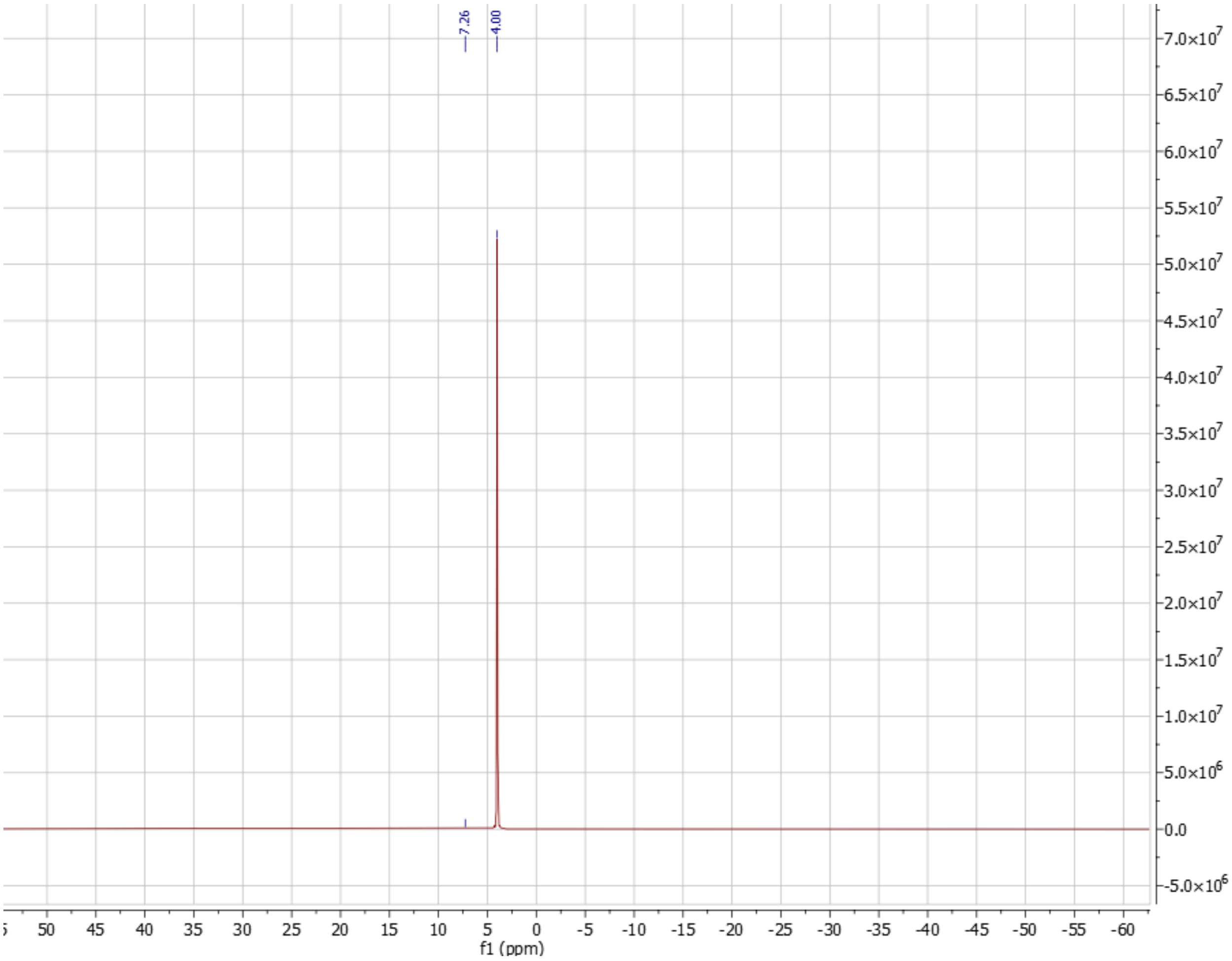
7.26
4.00
7.0×10^7
6.5×10^7
6.0×10^7
5.5×10^7
5.0×10^7
4.5×10^7
4.0×10^7
3.5×10^7
3.0×10^7
2.5×10^7
2.0×10^7
1.5×10^7
1.0×10^7
5.0×10^6
0.0
-5.0×10^6
50 45 40 35 30 25 20 15 10 5 0 -5 -10 -15 -20 -25 -30 -35 -40 -45 -50 -55 -60
f1 (ppm)

^1H NMR

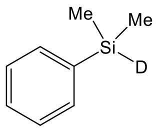

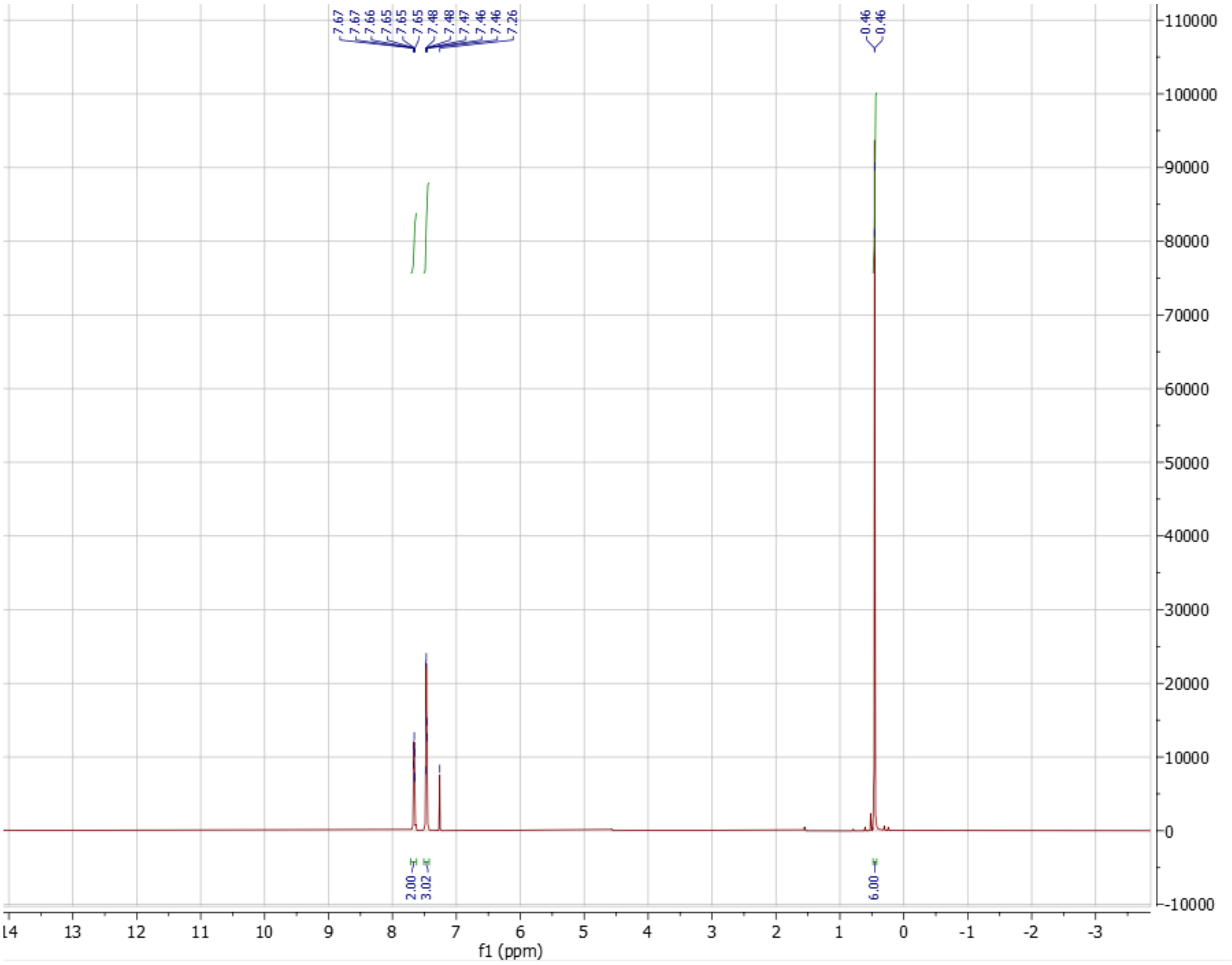

7.67
7.67
7.66
7.65
7.65
7.65
7.48
7.48
7.47
7.46
7.46
7.46
7.26
0.46
0.46
2.00
3.02
6.00
f1 (ppm)

^{13}C NMR

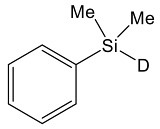

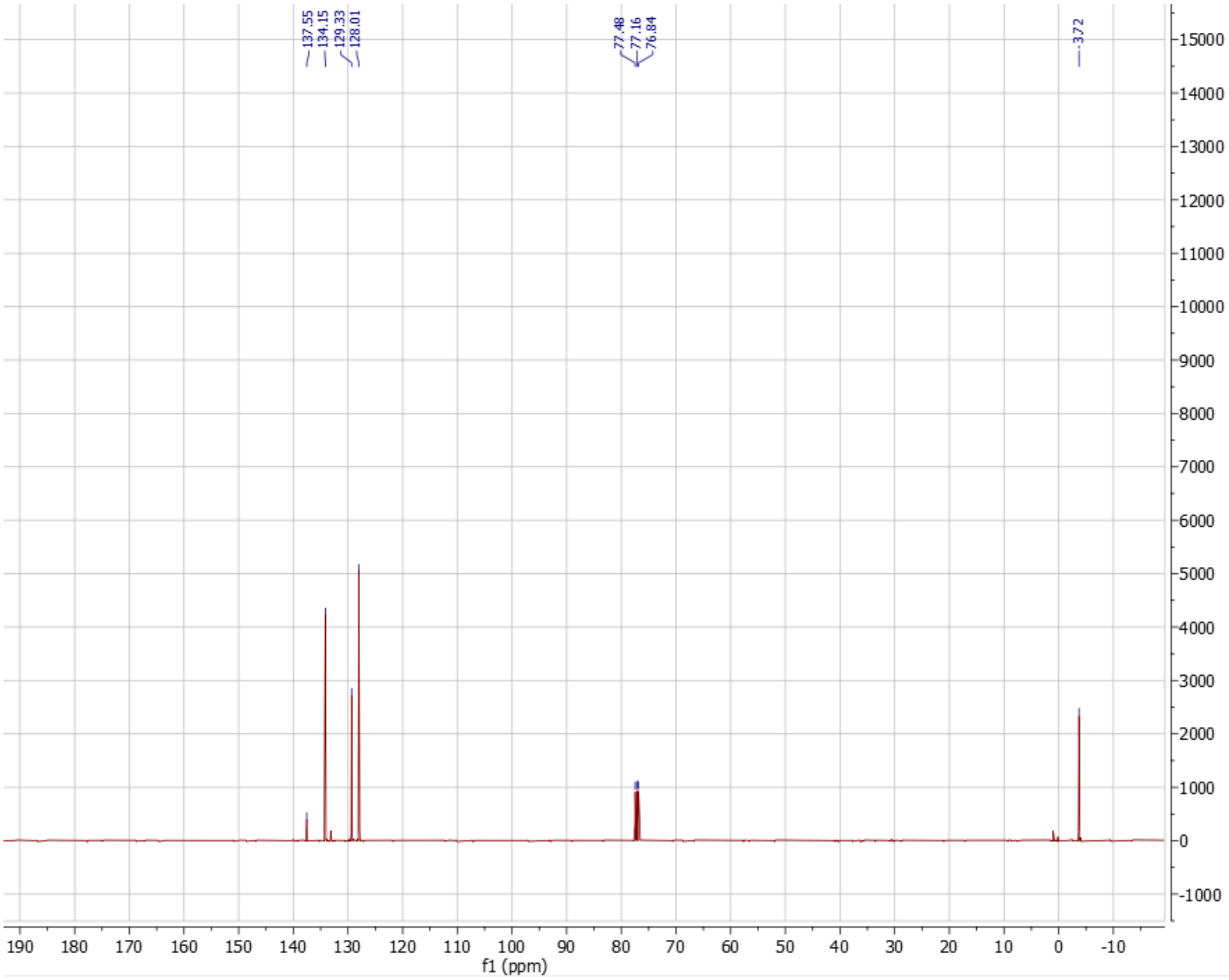

^{1}H NMR

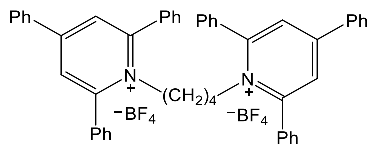

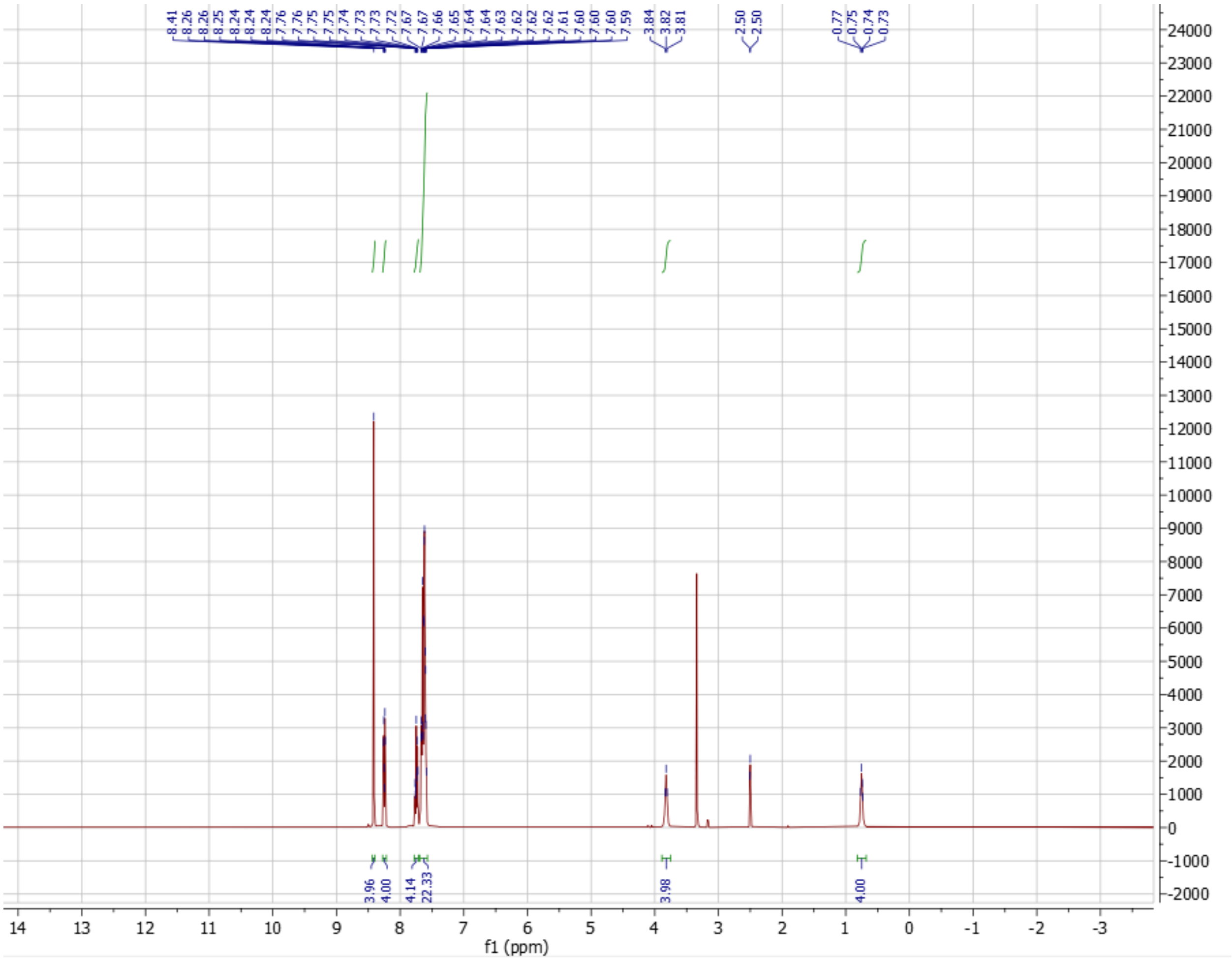

^{13}C NMR

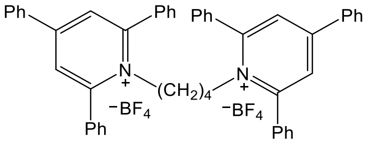
Ph
Ph
Ph
Ph
N
N
(CH2)4
−BF4
−BF4
Ph
Ph

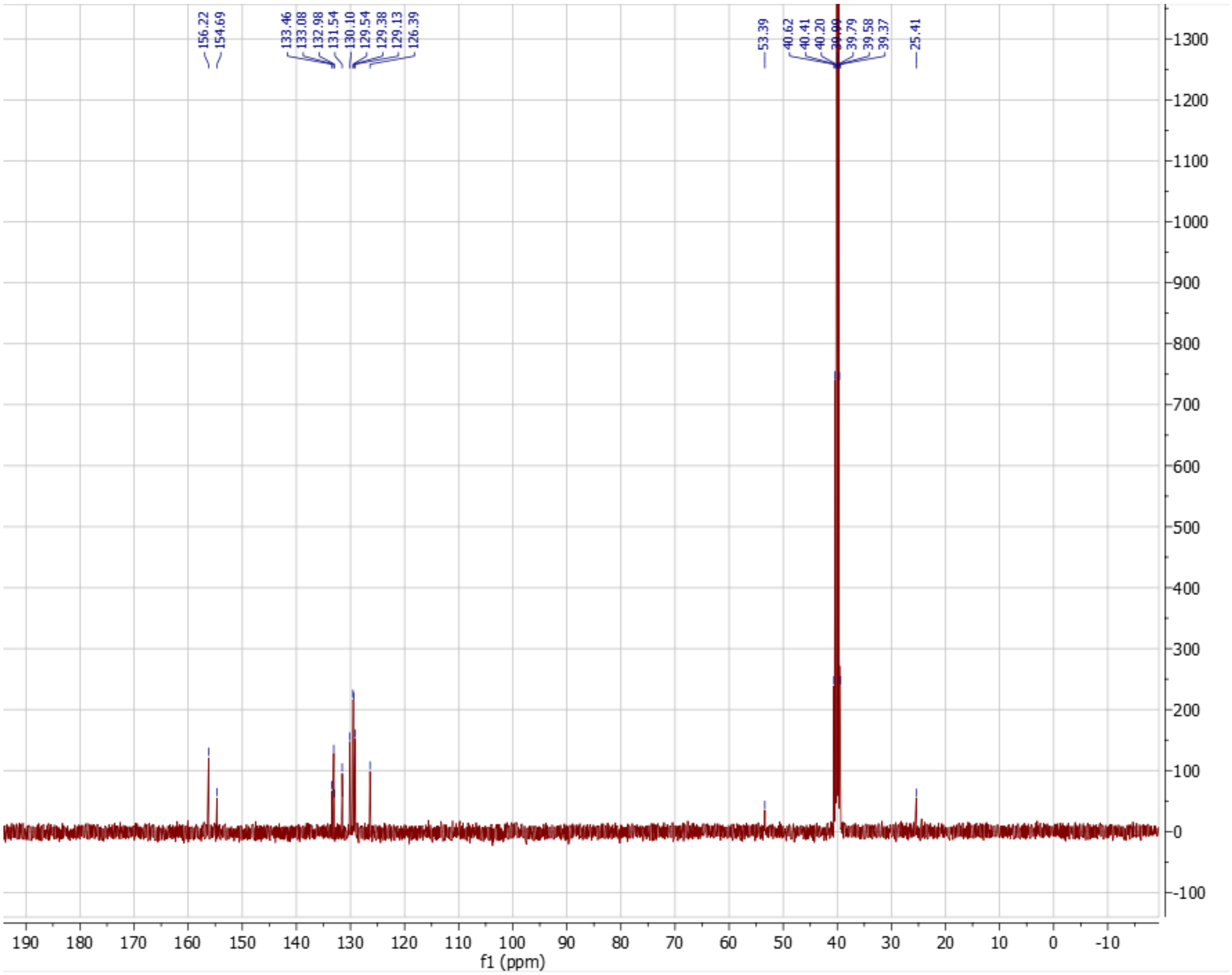
156.22
154.69
133.46
133.08
132.98
131.54
130.10
129.54
129.38
129.13
126.39
53.39
40.62
40.41
40.20
39.79
39.58
39.37
25.41
f1 (ppm)

^{19}F NMR

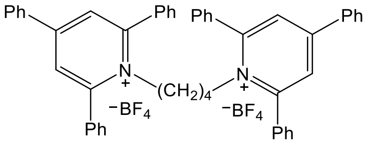

Ph
Ph
Ph
Ph
Ph
Ph
N
N
(CH2)4
−BF4
−BF4

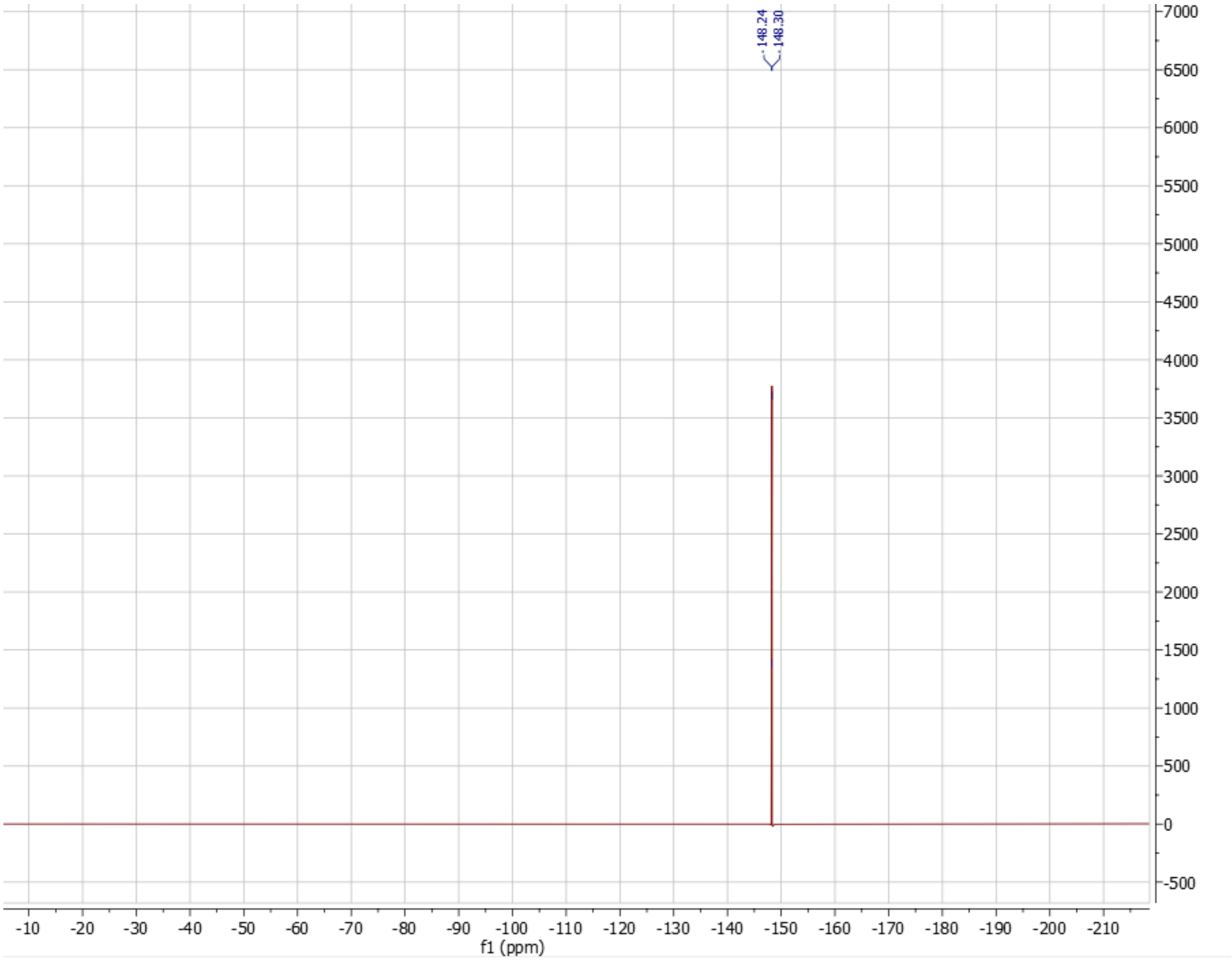

-148.24
-148.30
7000
6500
6000
5500
5000
4500
4000
3500
3000
2500
2000
1500
1000
500
0
-500
-10
-20
-30
-40
-50
-60
-70
-80
-90
-100
-110
-120
-130
-140
-150
-160
-170
-180
-190
-200
-210
f1 (ppm)

^{1}H NMR

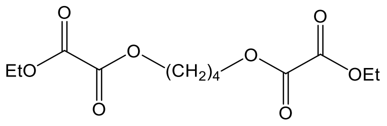
EtO
O
O
O
(CH2)4
O
O
O
OEt

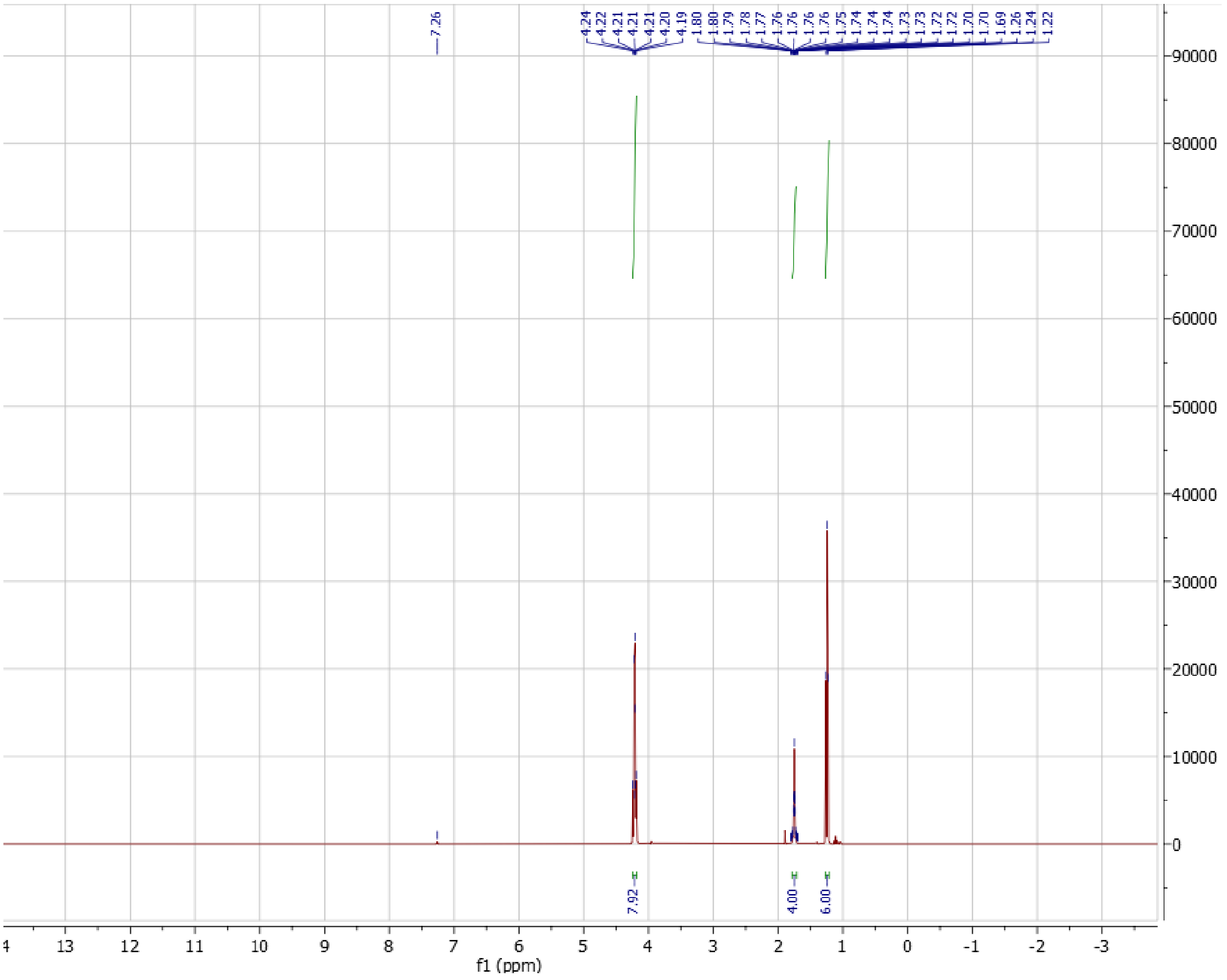
7.26
4.24
4.22
4.21
4.21
4.21
4.20
4.19
1.80
1.80
1.79
1.78
1.77
1.76
1.76
1.76
1.76
1.75
1.74
1.74
1.74
1.73
1.73
1.72
1.72
1.70
1.69
1.26
1.24
1.22
7.92
4.00
6.00
f1 (ppm)

^{13}C NMR

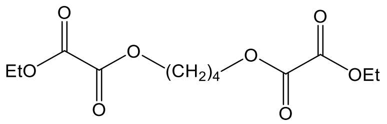

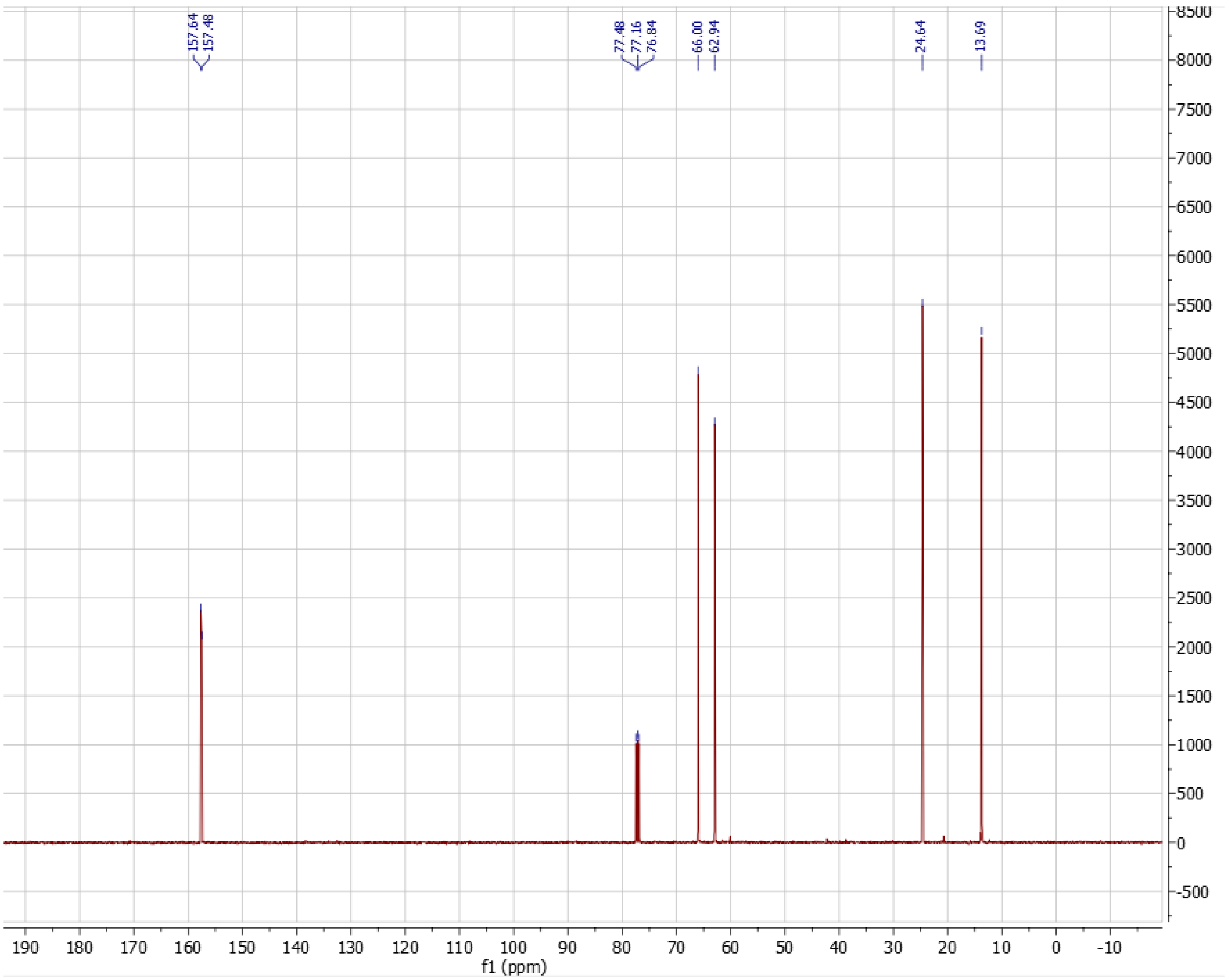

^{1}H NMR

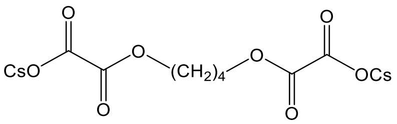

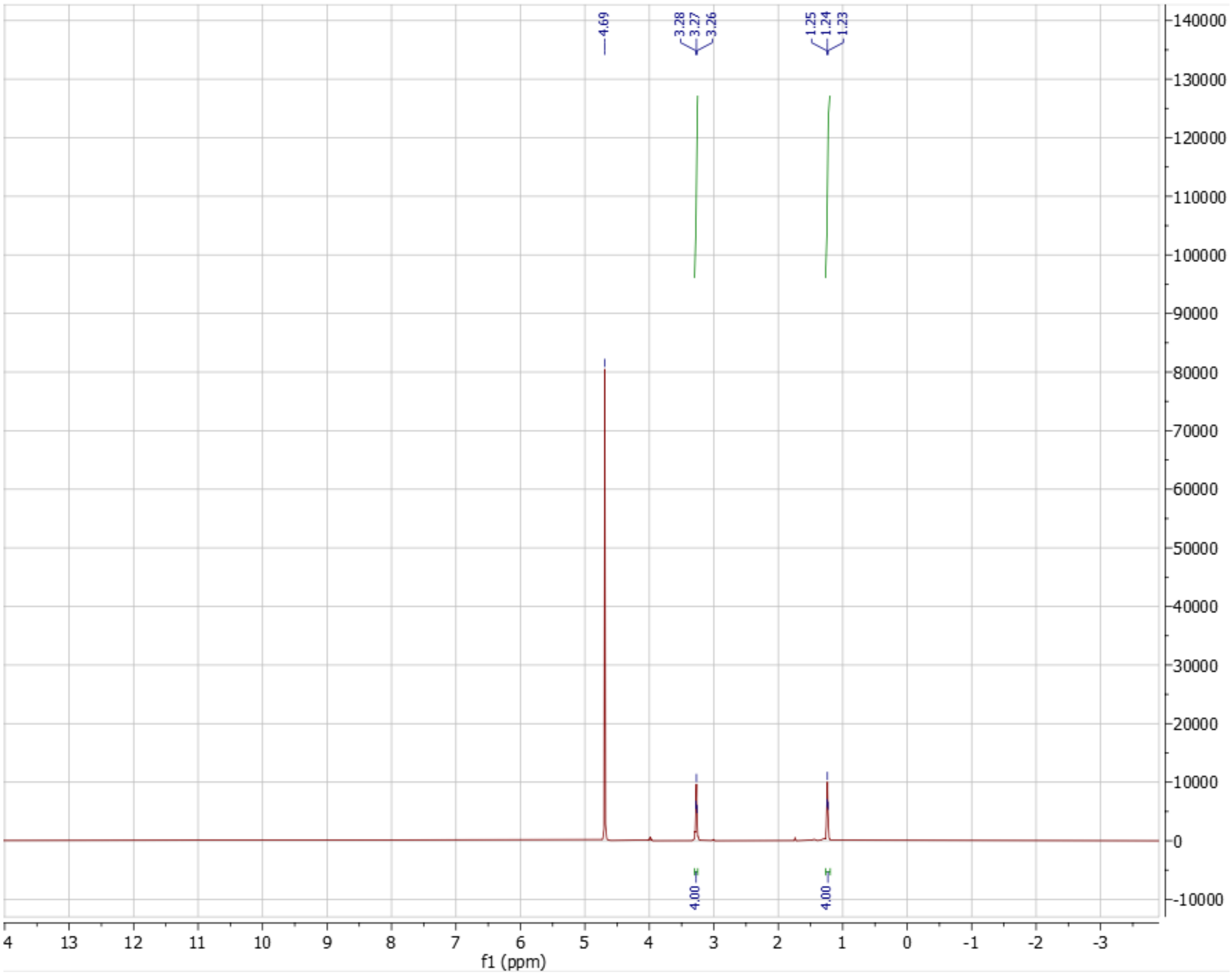

^{1}H NMR

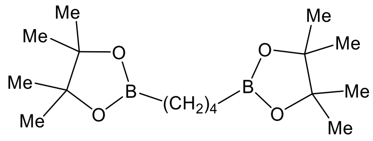

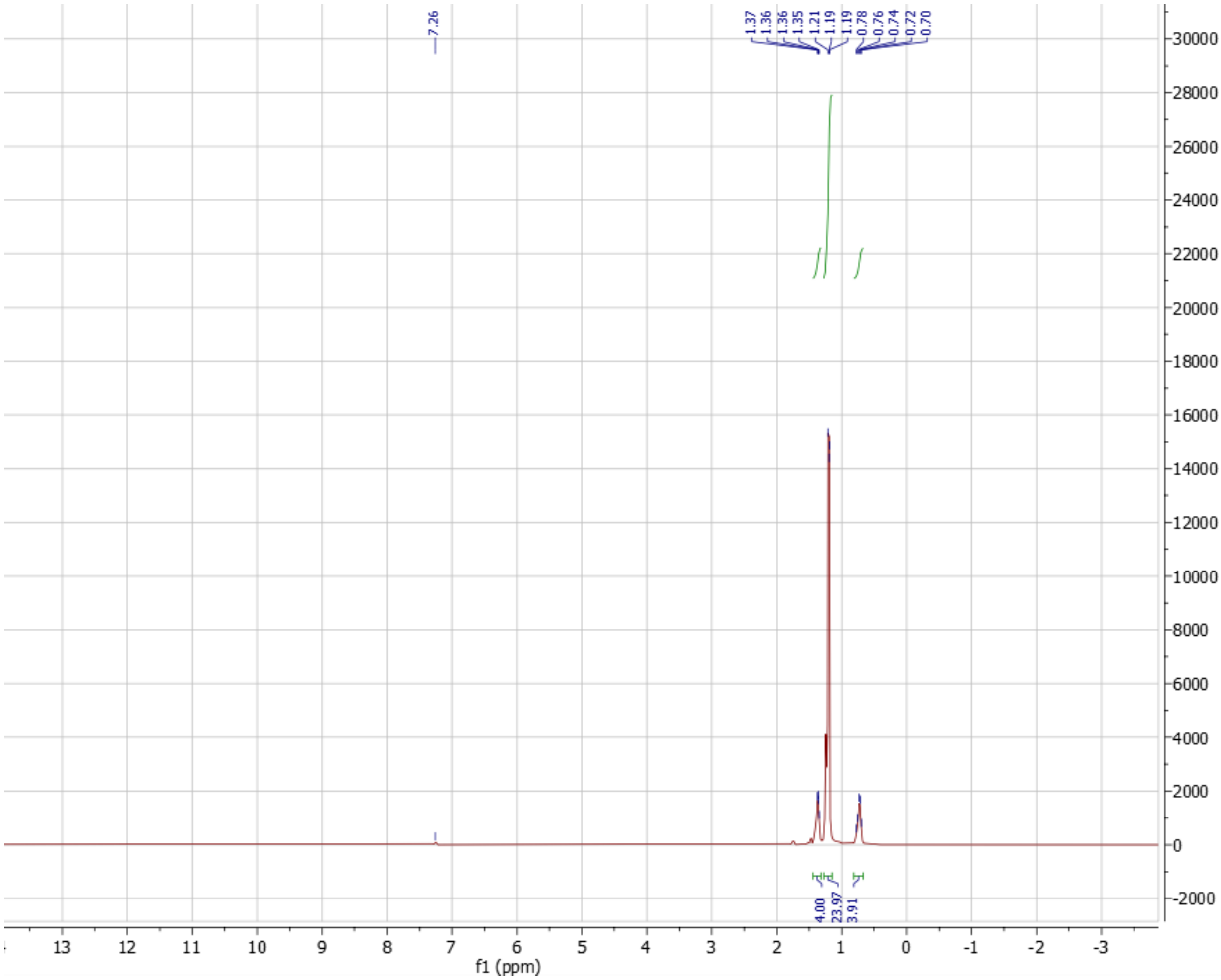

7.26
1.37
1.36
1.36
1.35
1.21
1.19
1.19
0.78
0.76
0.74
0.72
0.70
4.00
23.97
3.91
f1 (ppm)

^{13}C NMR

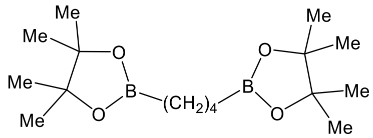
Me
Me
O
Me
B
(CH2)4
Me
O
Me
O
Me
B
Me
O
Me

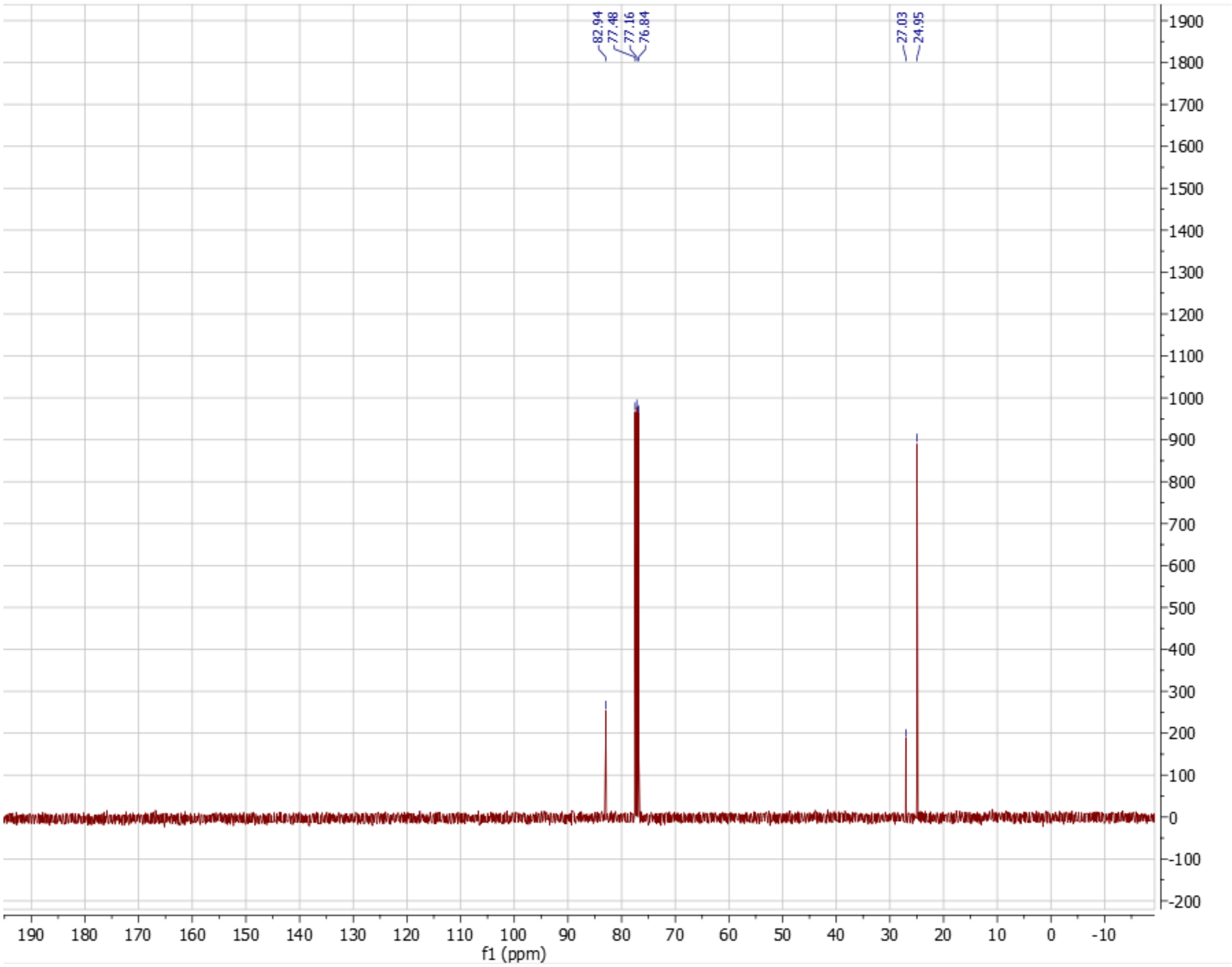
82.94
77.48
77.16
76.84
27.03
24.95
f1 (ppm)

^{11}B NMR

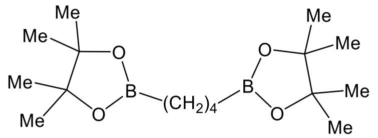

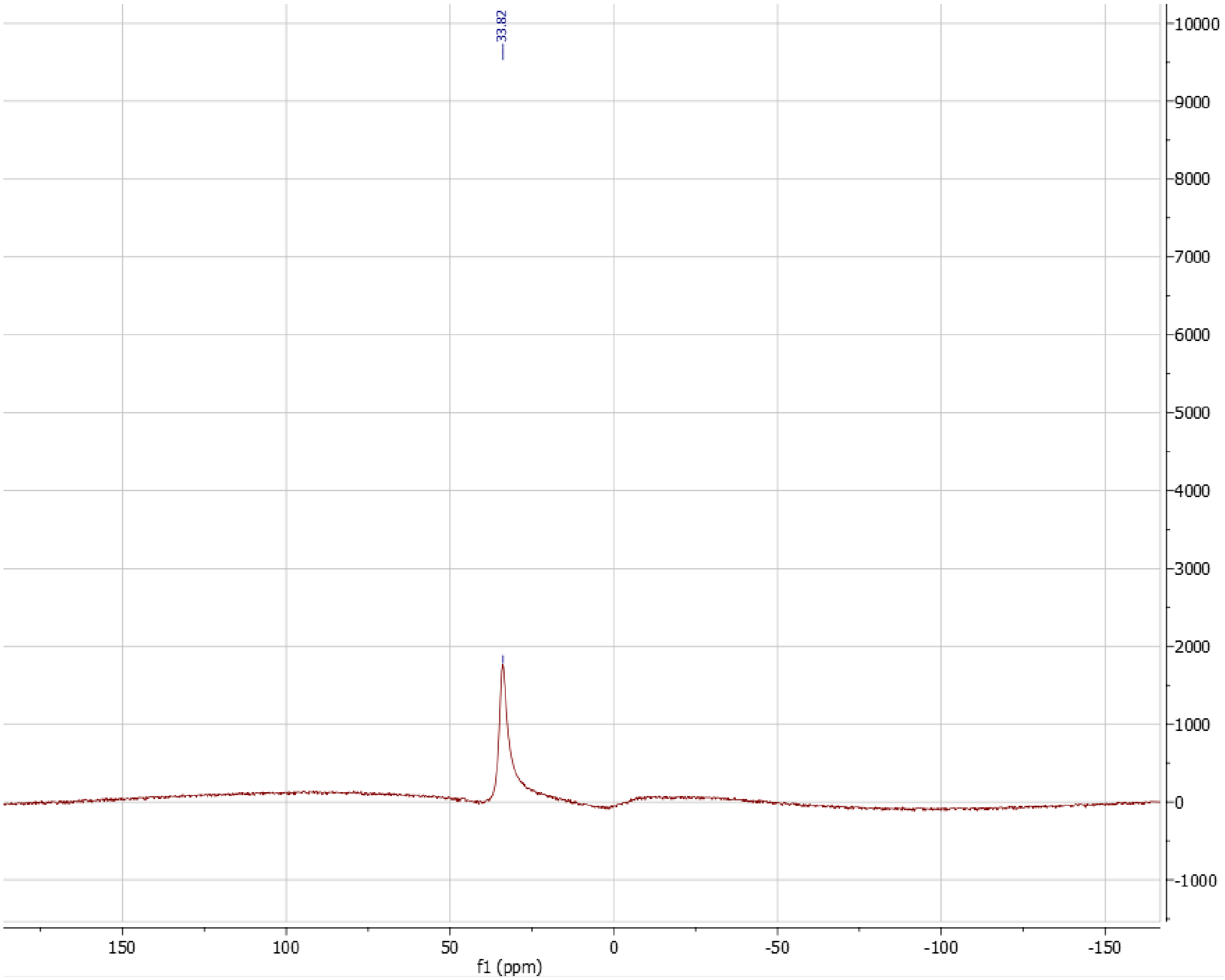

33.82
10000
9000
8000
7000
6000
5000
4000
3000
2000
1000
0
-1000
150
100
50
0
-50
-100
-150
f1 (ppm)

^{1}H NMR

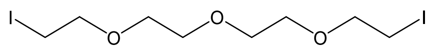

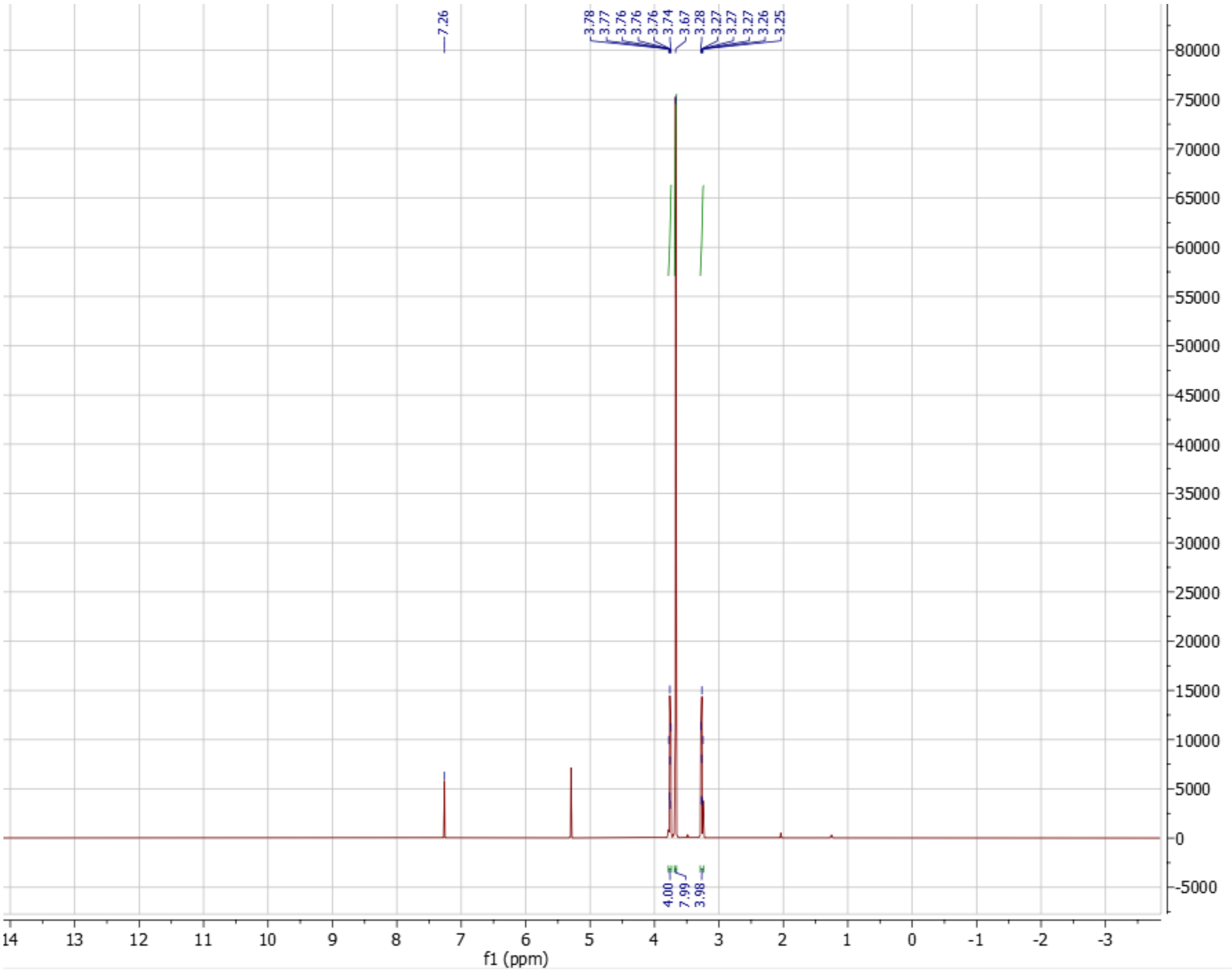
7.26
3.78
3.77
3.76
3.76
3.76
3.74
3.67
3.28
3.27
3.27
3.27
3.26
3.25
4.00
7.99
3.98
f1 (ppm)

^{13}C NMR

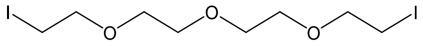

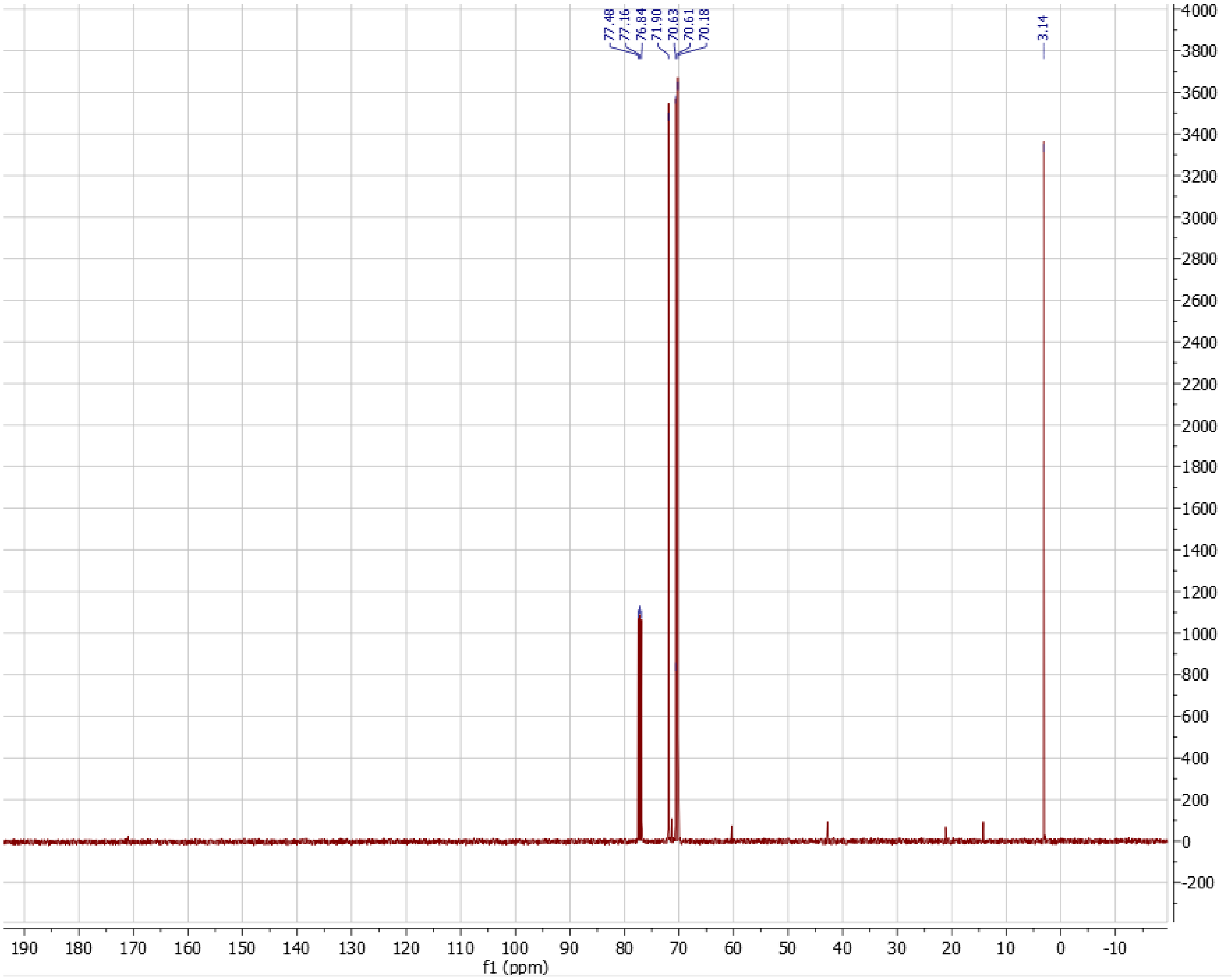

^1H NMR

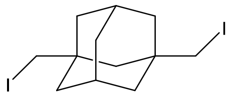

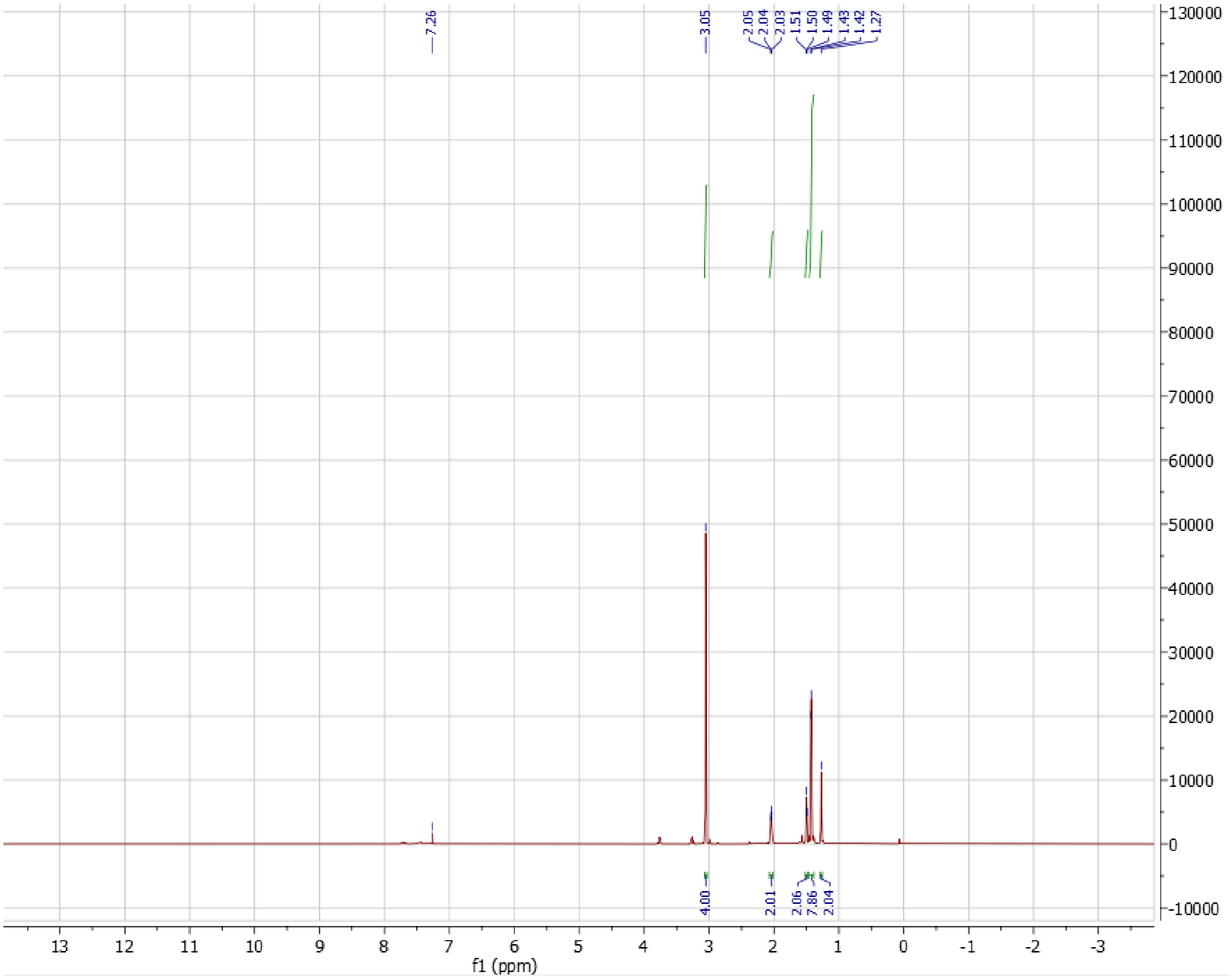
7.26
3.05
2.05
2.04
2.03
1.51
1.50
1.49
1.43
1.42
1.27
4.00
2.01
2.06
7.86
2.04
f1 (ppm)

^{13}C NMR

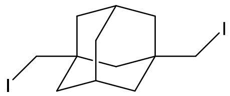

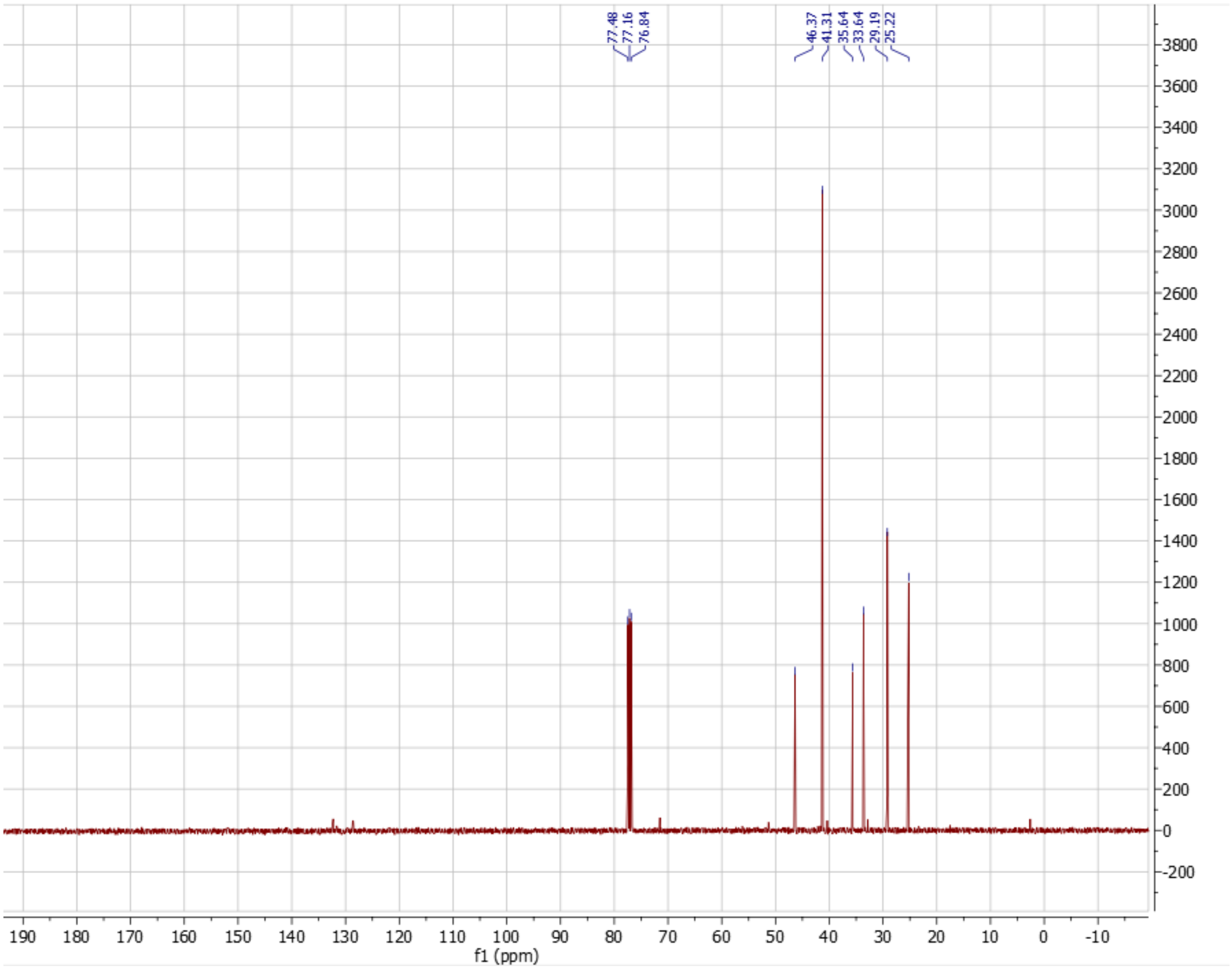

^{1}H NMR

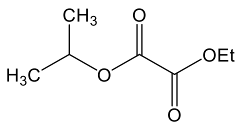

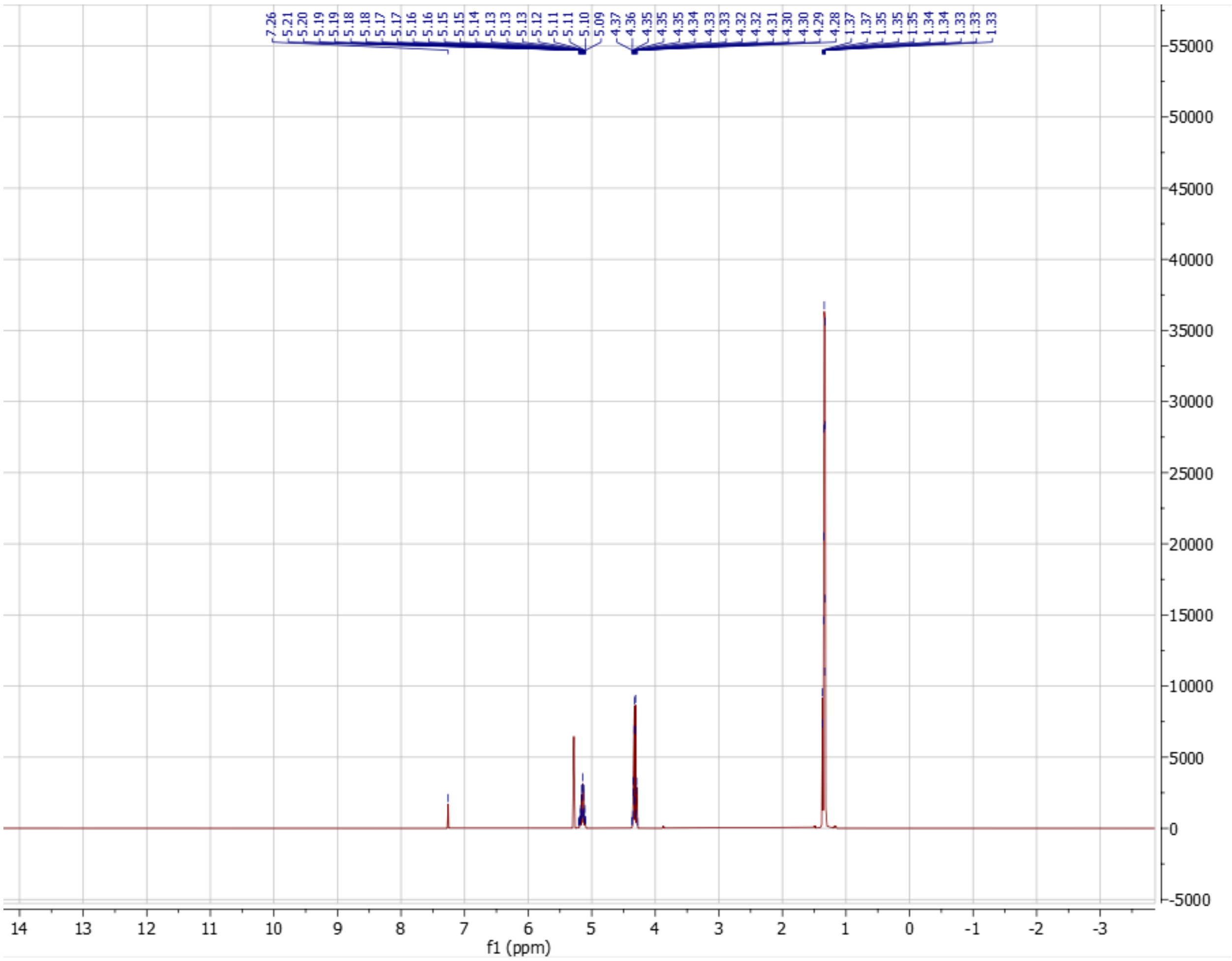

^{13}C NMR

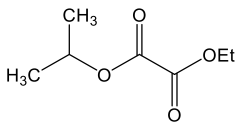

CH3
O
OEt
H3C
O
O

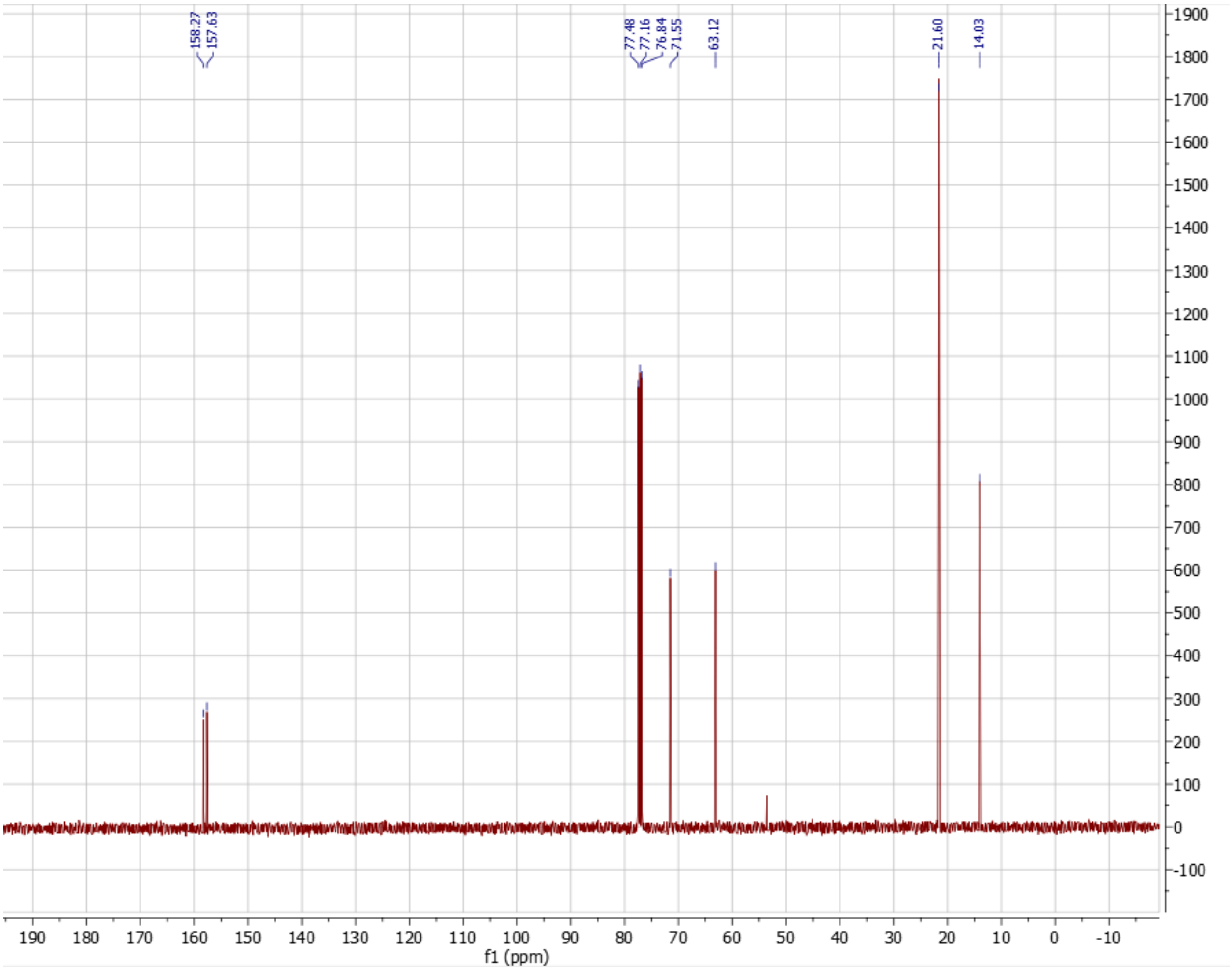

158.27
157.63
77.48
77.16
76.84
71.55
63.12
21.60
14.03
f1 (ppm)

Peptide Diastereomer Characterization

Setmelanotide ^1H

500 MHz, DMSO-d_6

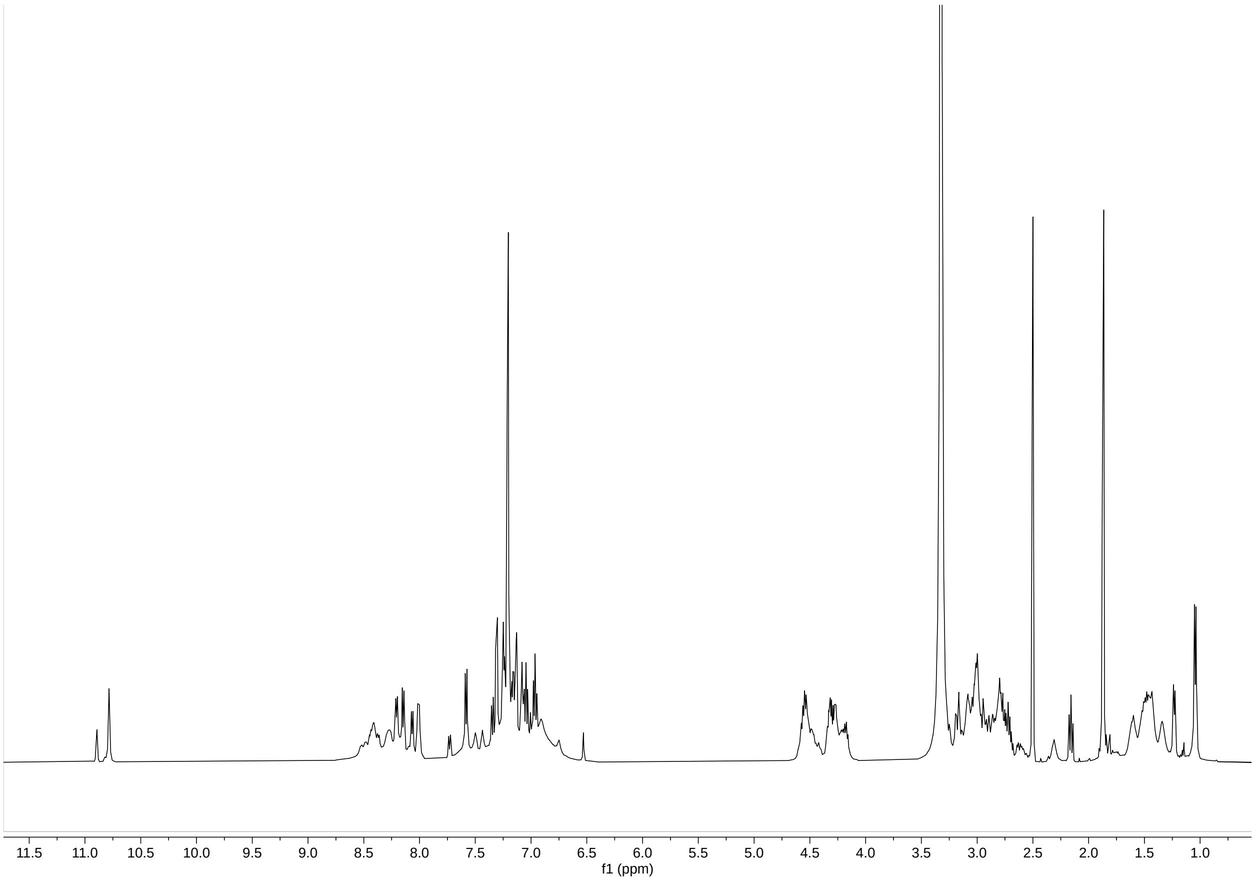

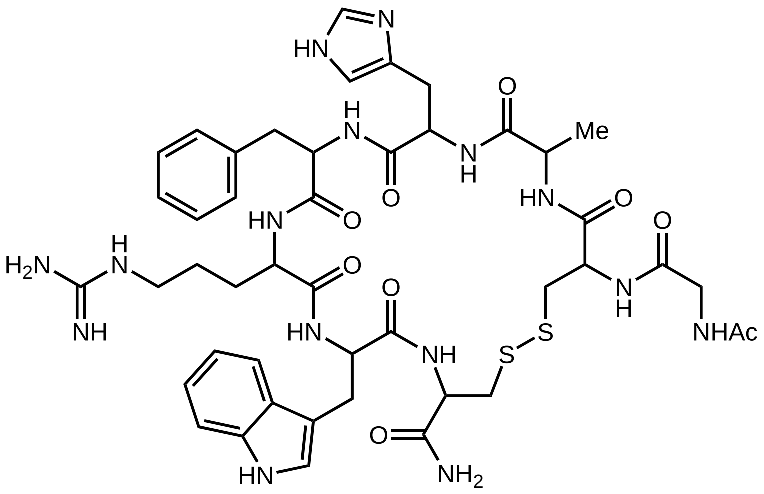

Setmelanotide ^{1}H-^{1}H COSY

500 MHz, DMSO-d_6

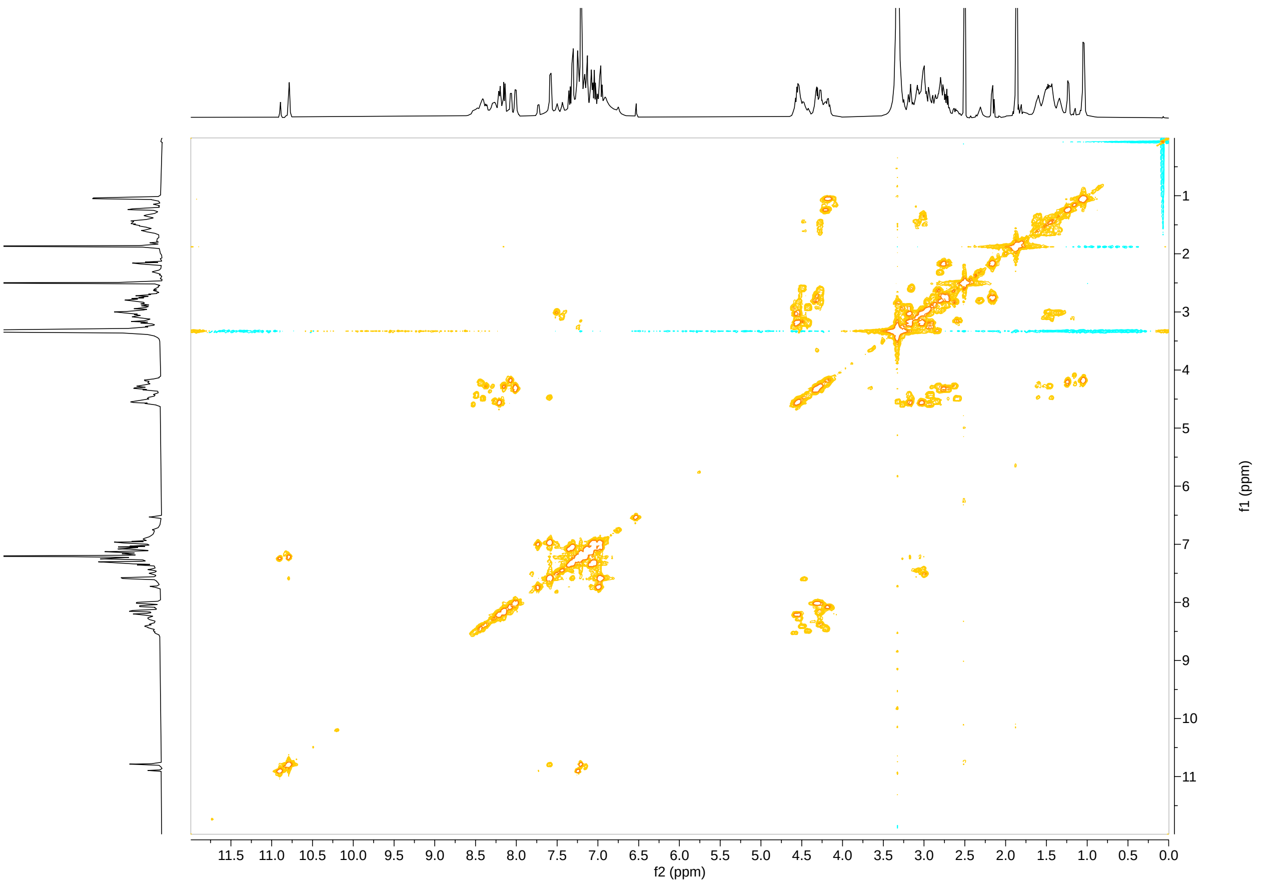

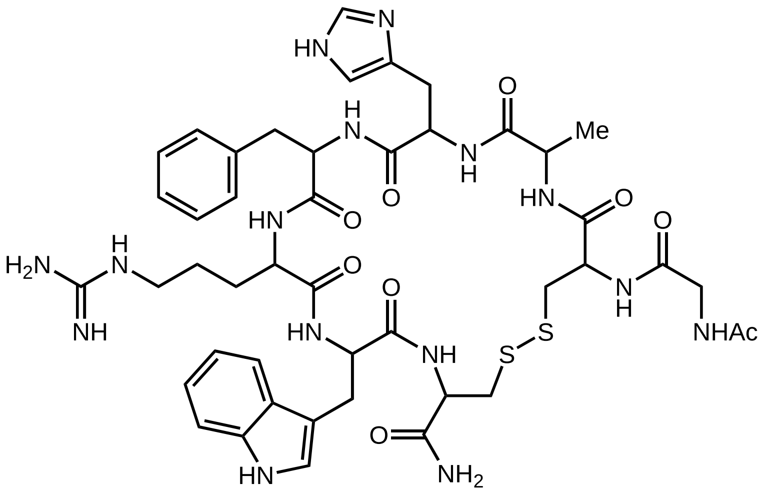

Setmelanotide ^1H-^1H TOCSY

500 MHz, DMSO-d_6

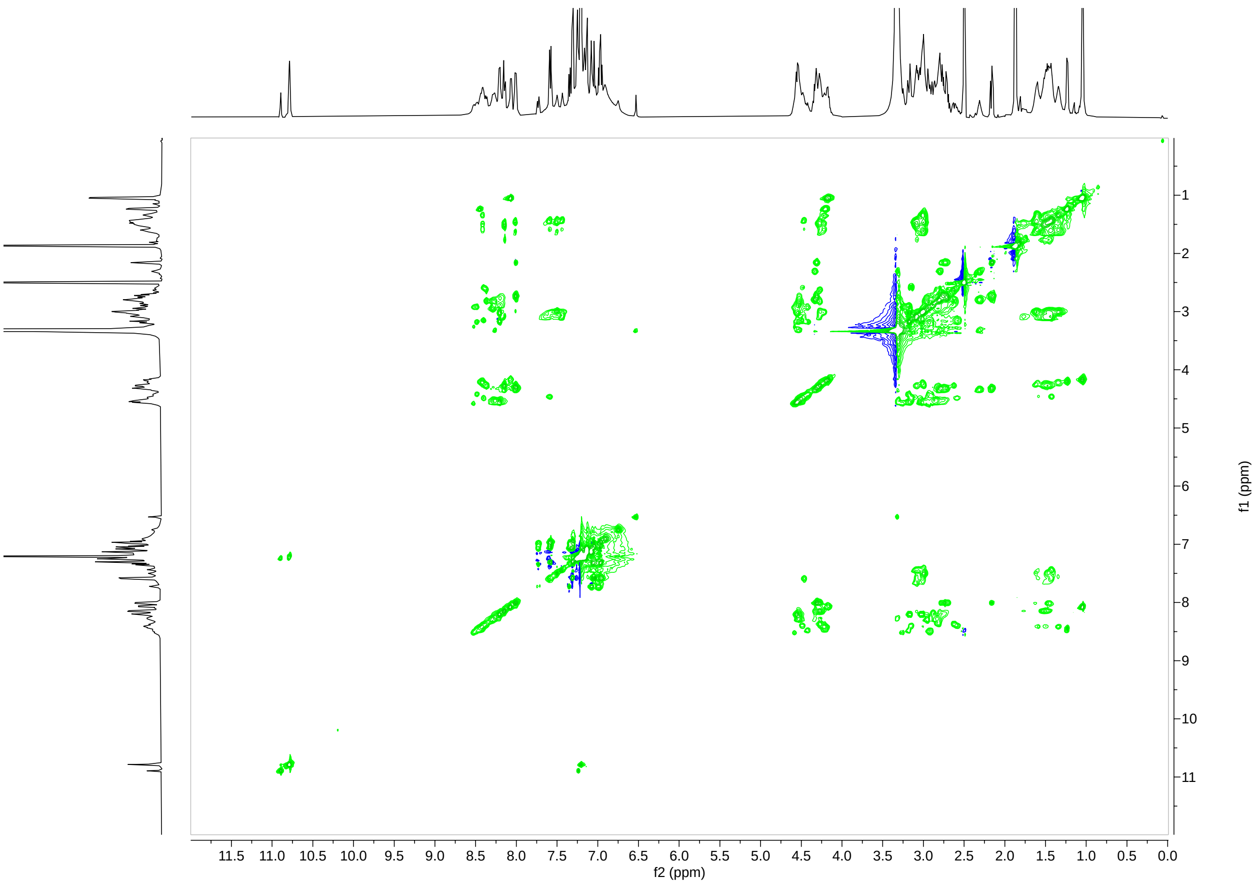

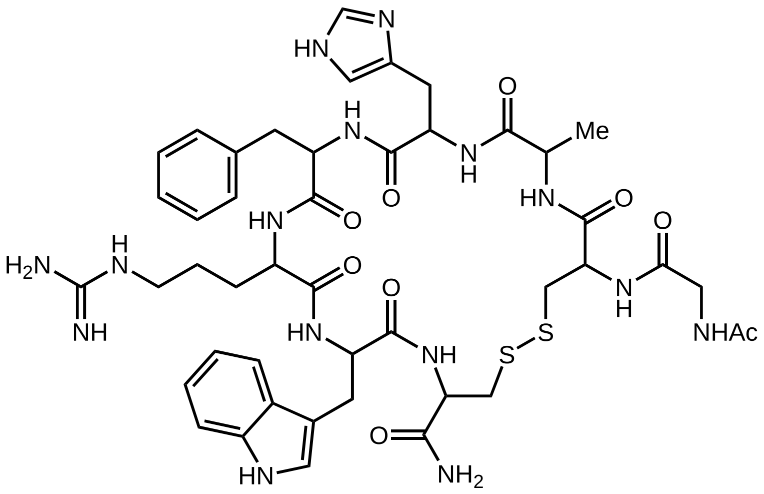

Setmelanotide ^{1}H-^{1}H NOESY

500 MHz, DMSO-d_6

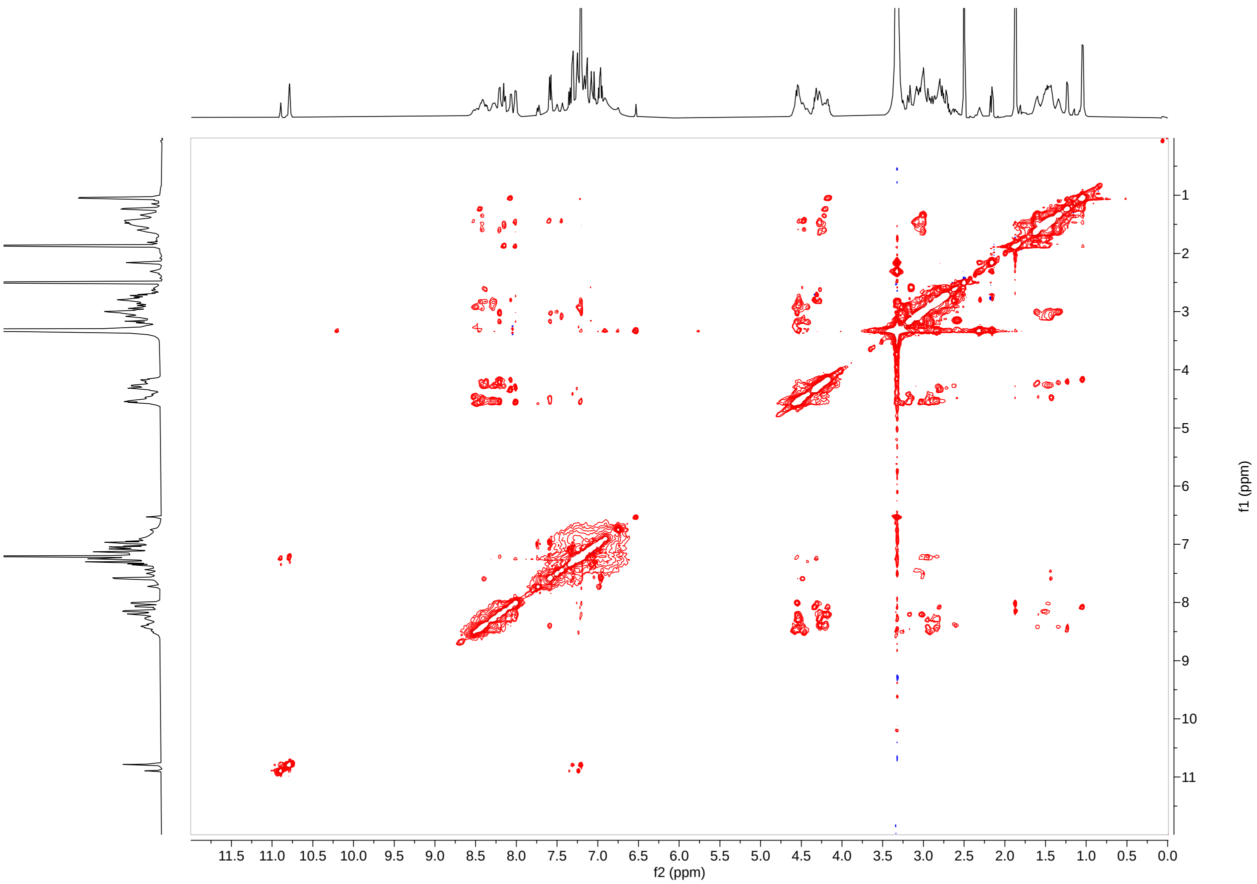

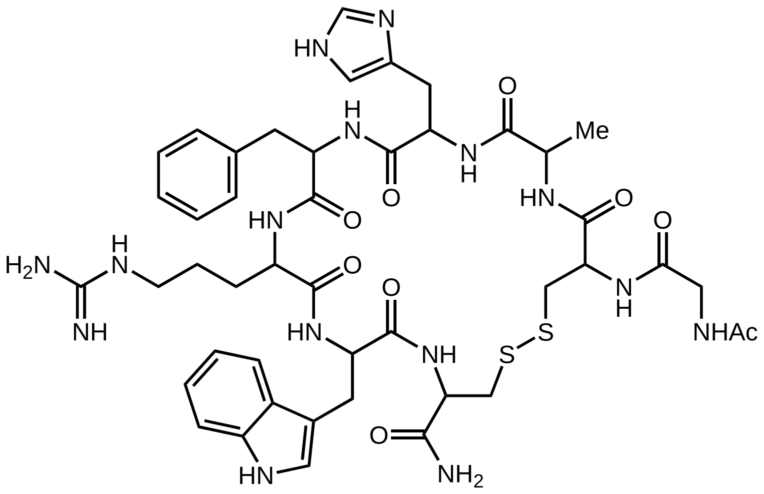

Set-Dha$_2$ ^{1}H

500 MHz, DMSO-d_6

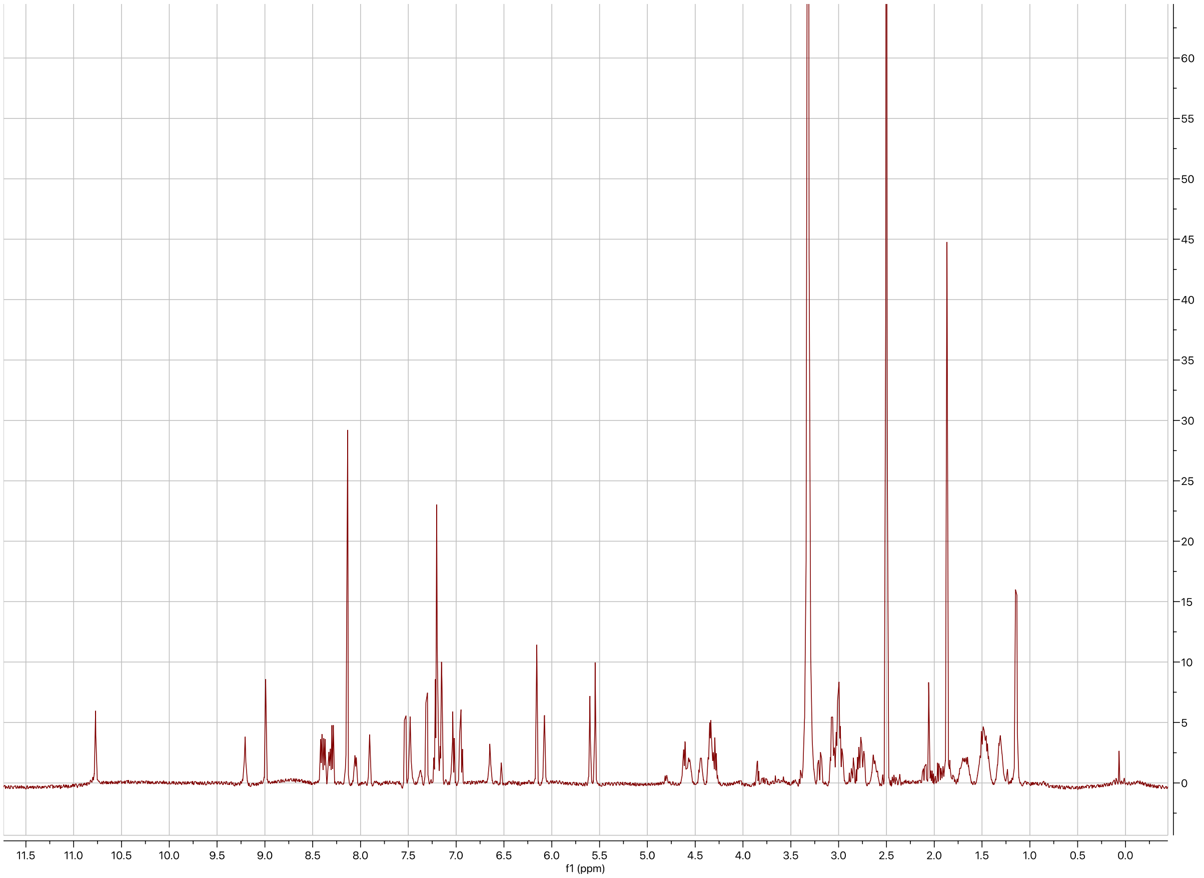

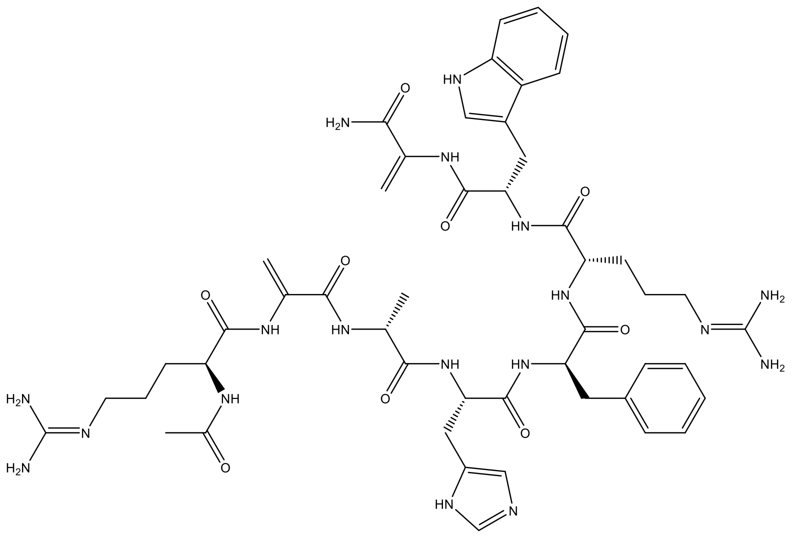

LC spectra for isolated diastereomer **i**

Set-C_5, Figure 2E

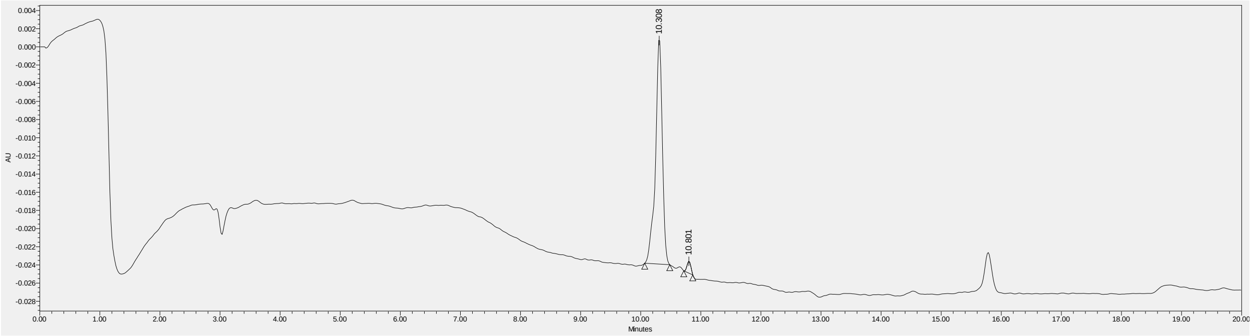

LC spectra for isolated diastereomer **ii**

Set-C_5, Figure 2E

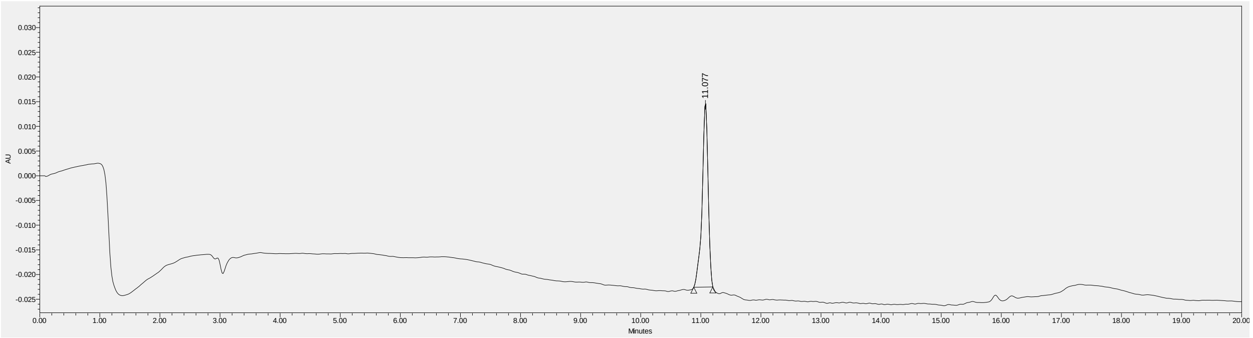

Set-C$_5$, diastereomer **i**, ^{1}H

600 MHz, DMSO-d_6

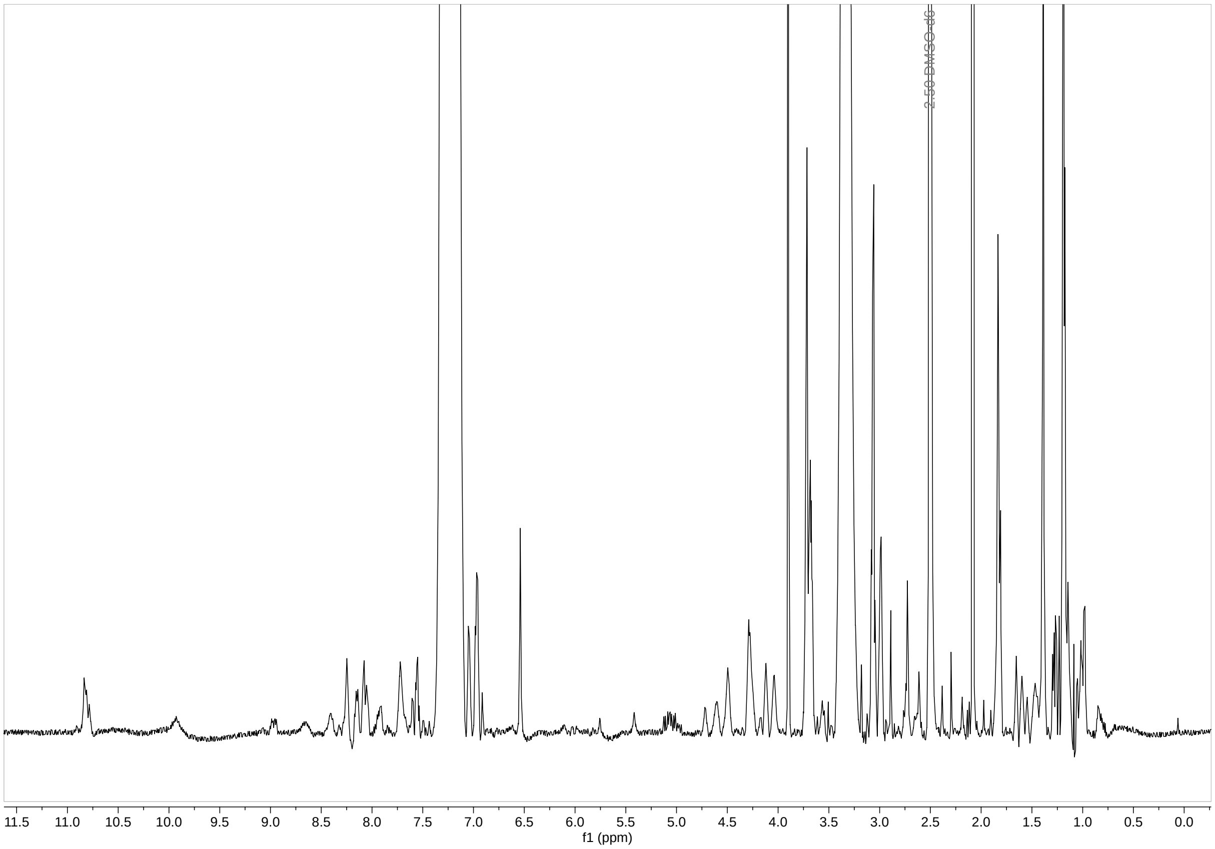

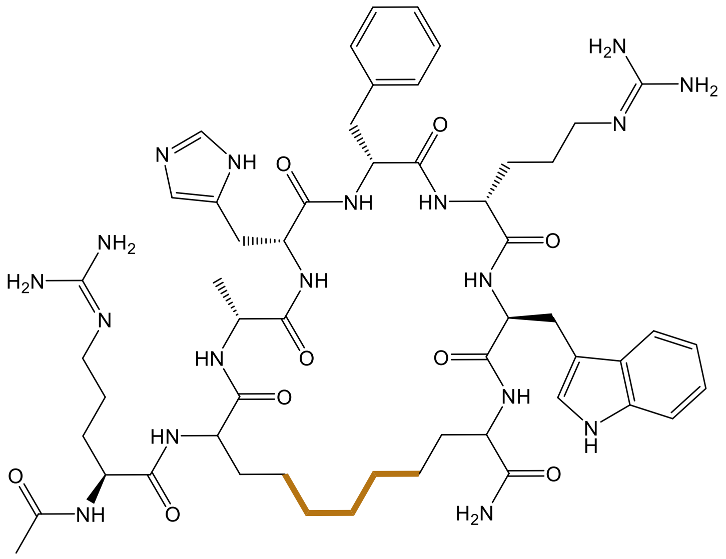

Set-C_5, diastereomer **i**, ^1H-^1H COSY

600 MHz, DMSO-d_6

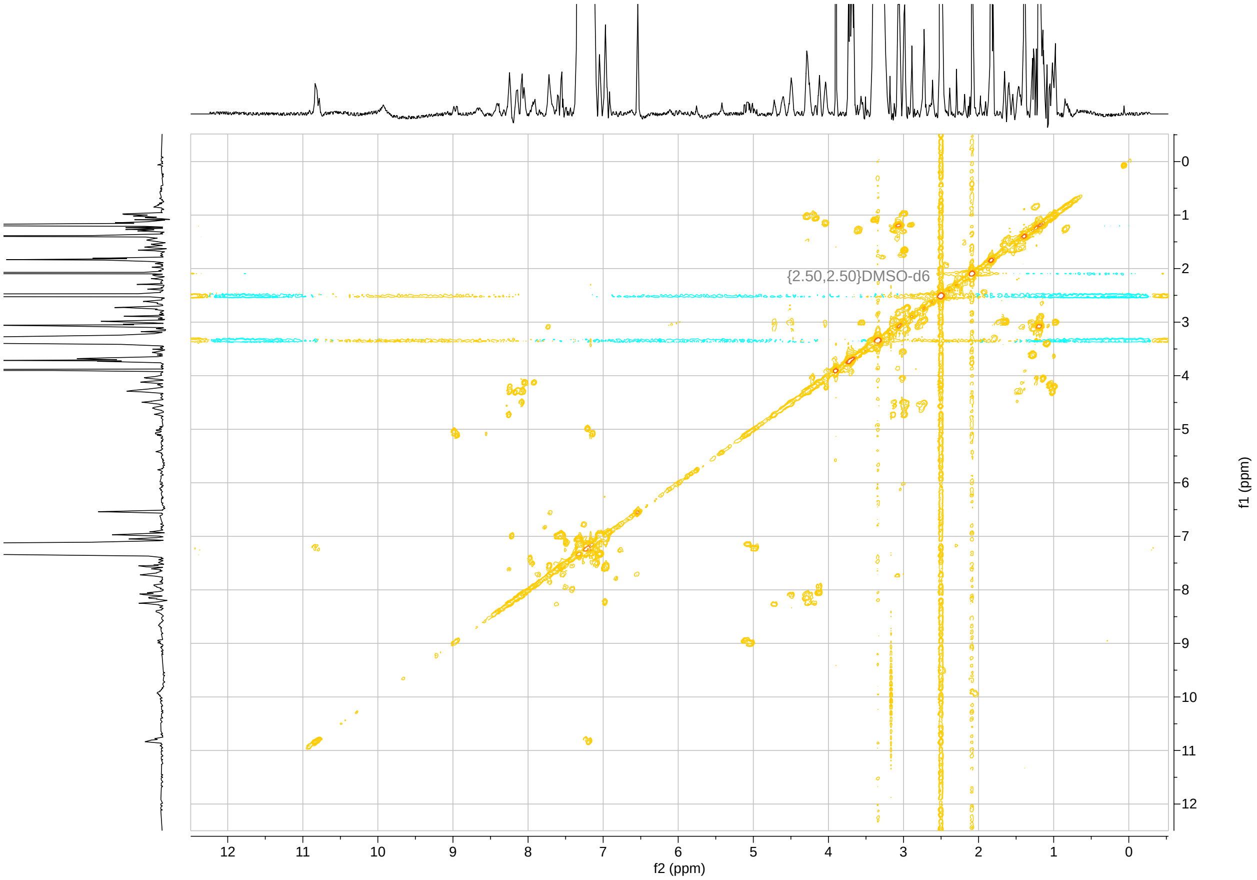

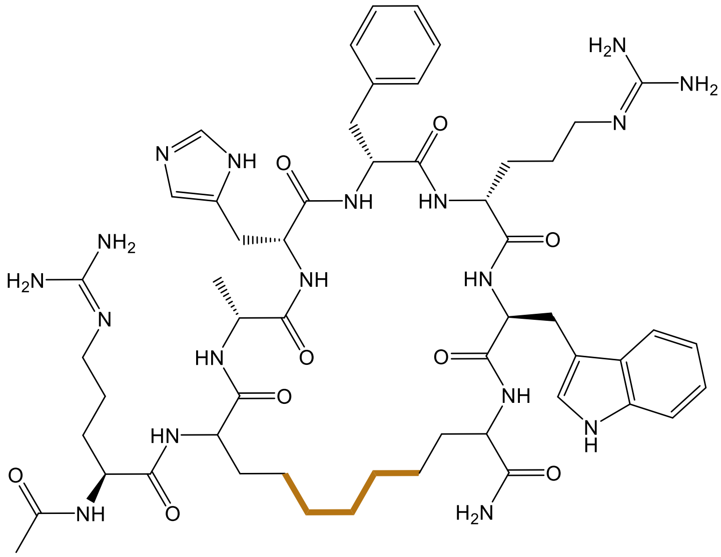

Set-C_5, diastereomer **i**, ^1H-^1H COSY

600 MHz, DMSO-d_6

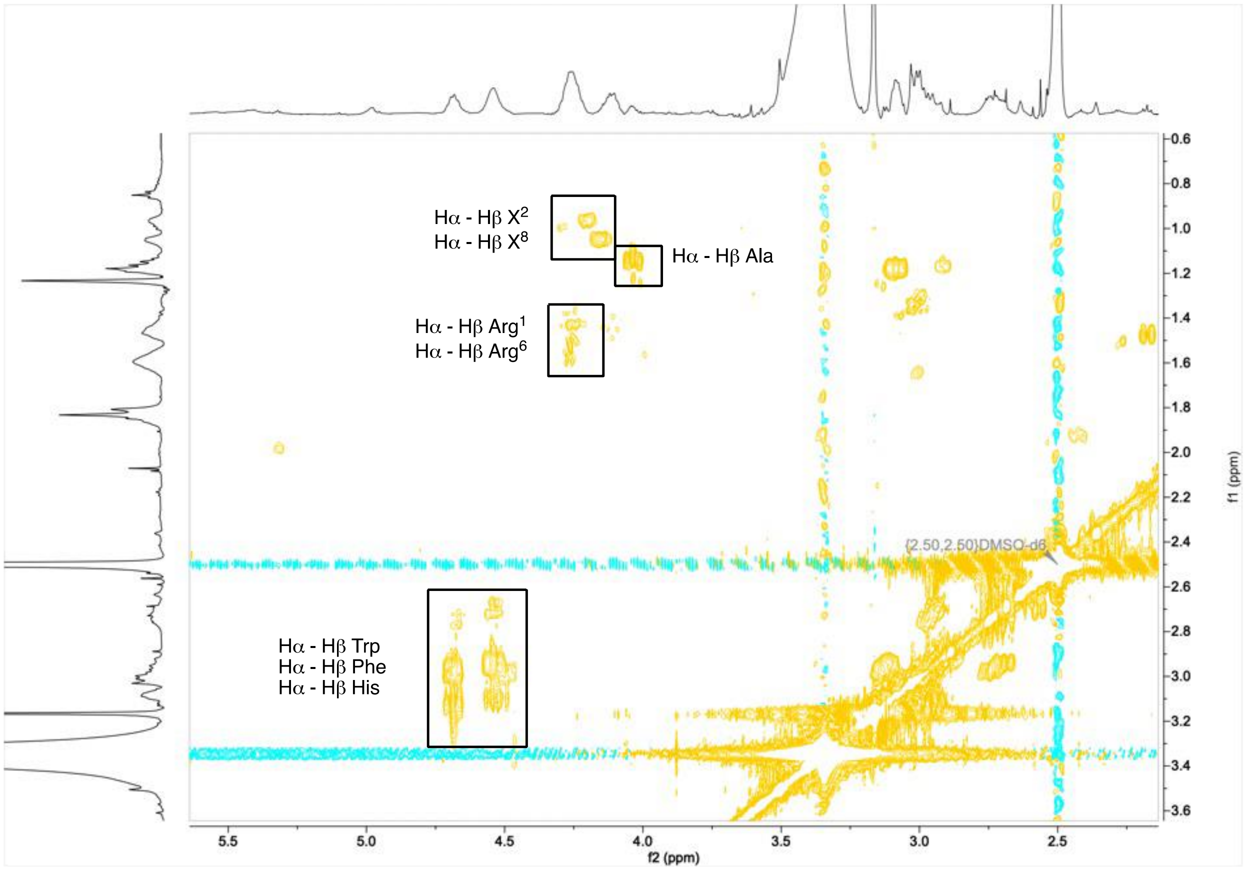

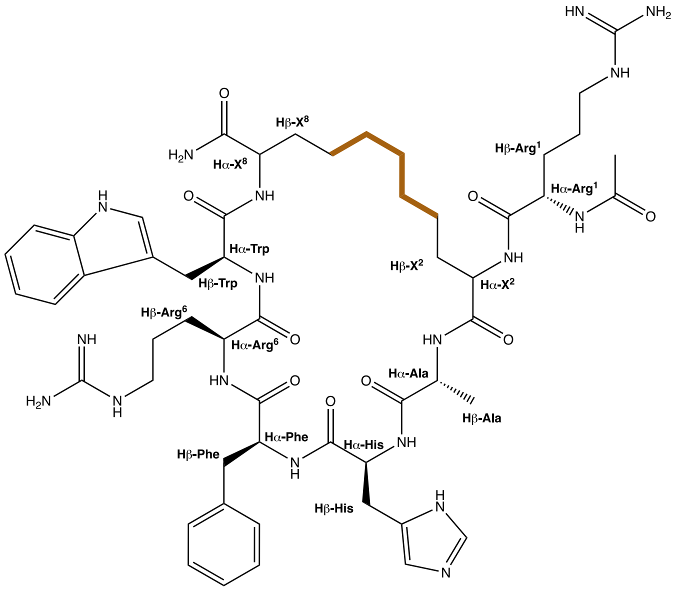

Set-C_5, diastereomer **i**, ^1H-^1H NOESY

600 MHz, DMSO-d_6

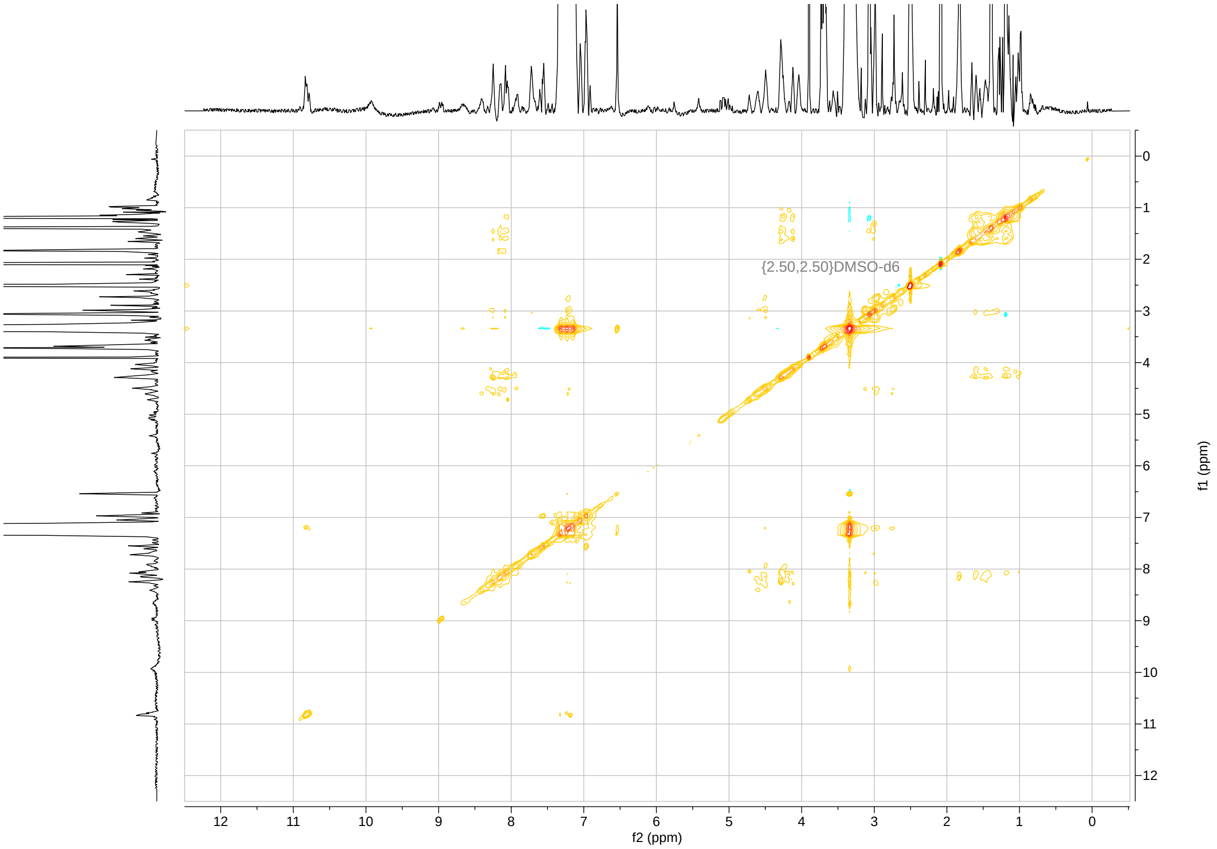

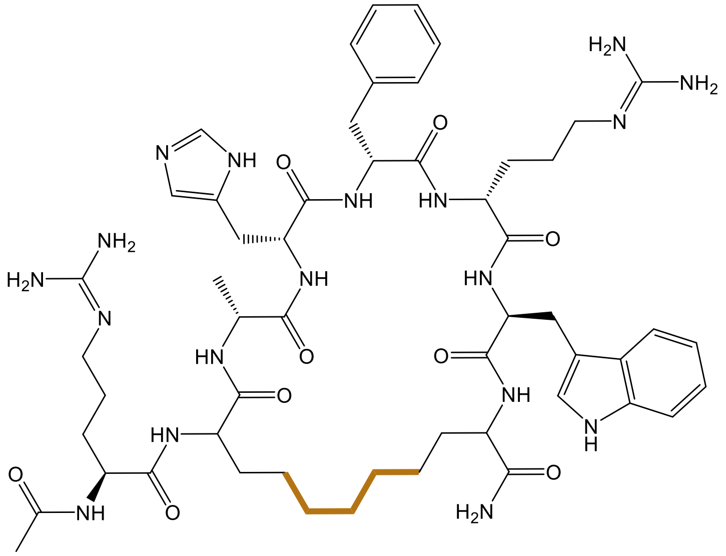

Set-C_5, diastereomer **ii**, ^1H

600 MHz, DMSO-d_6

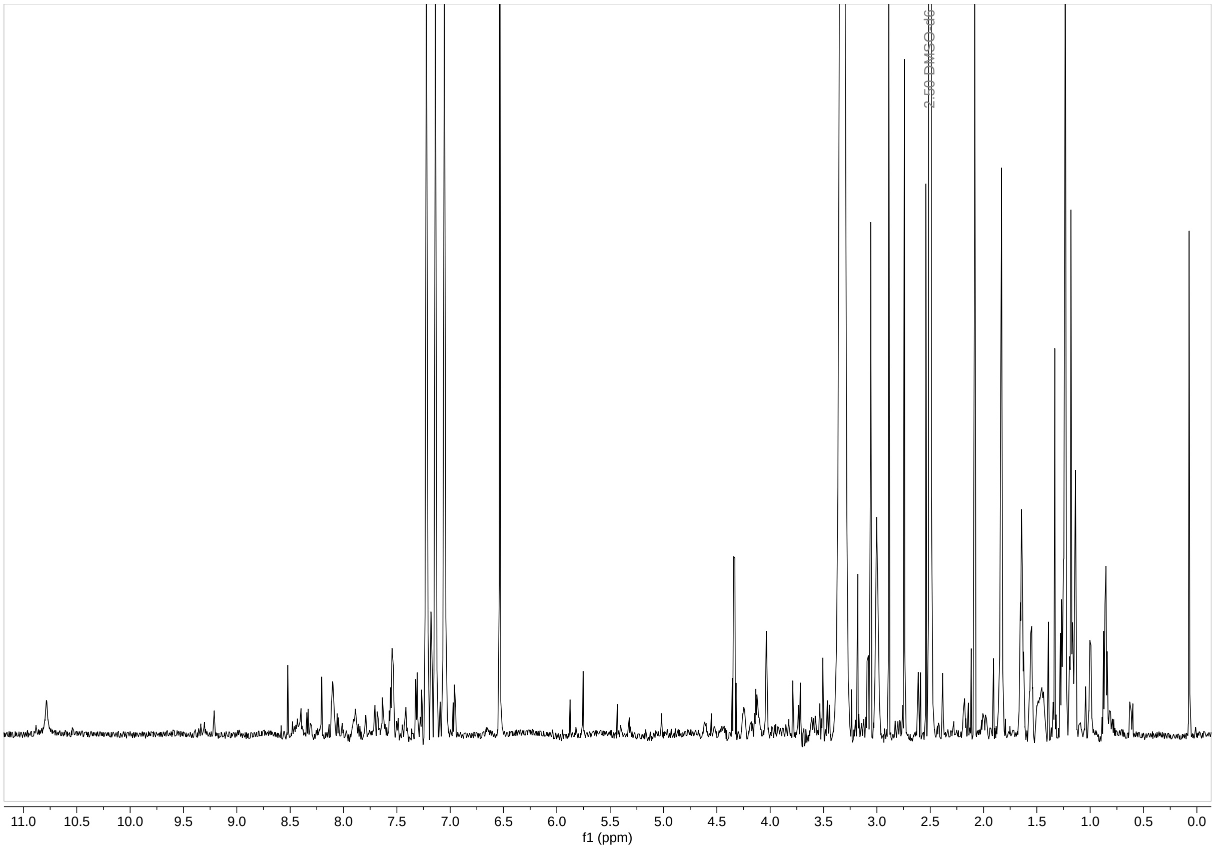

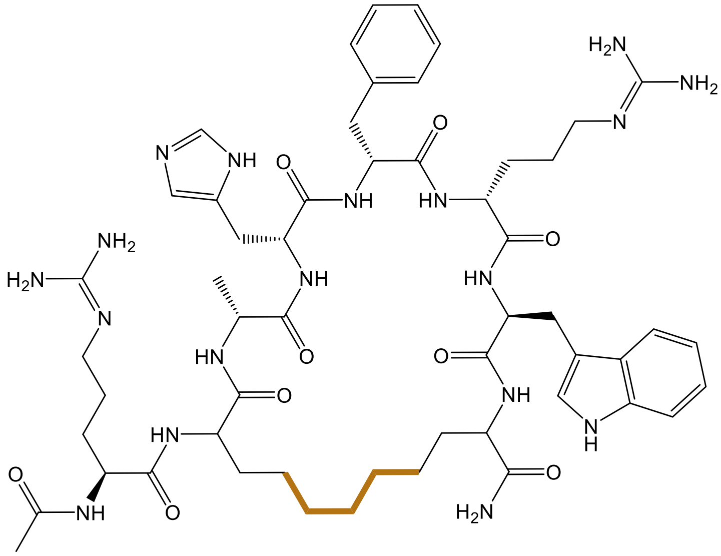

Set-C$_5$, diastereomer **ii**, ^1H-^1H COSY

600 MHz, DMSO-d_6

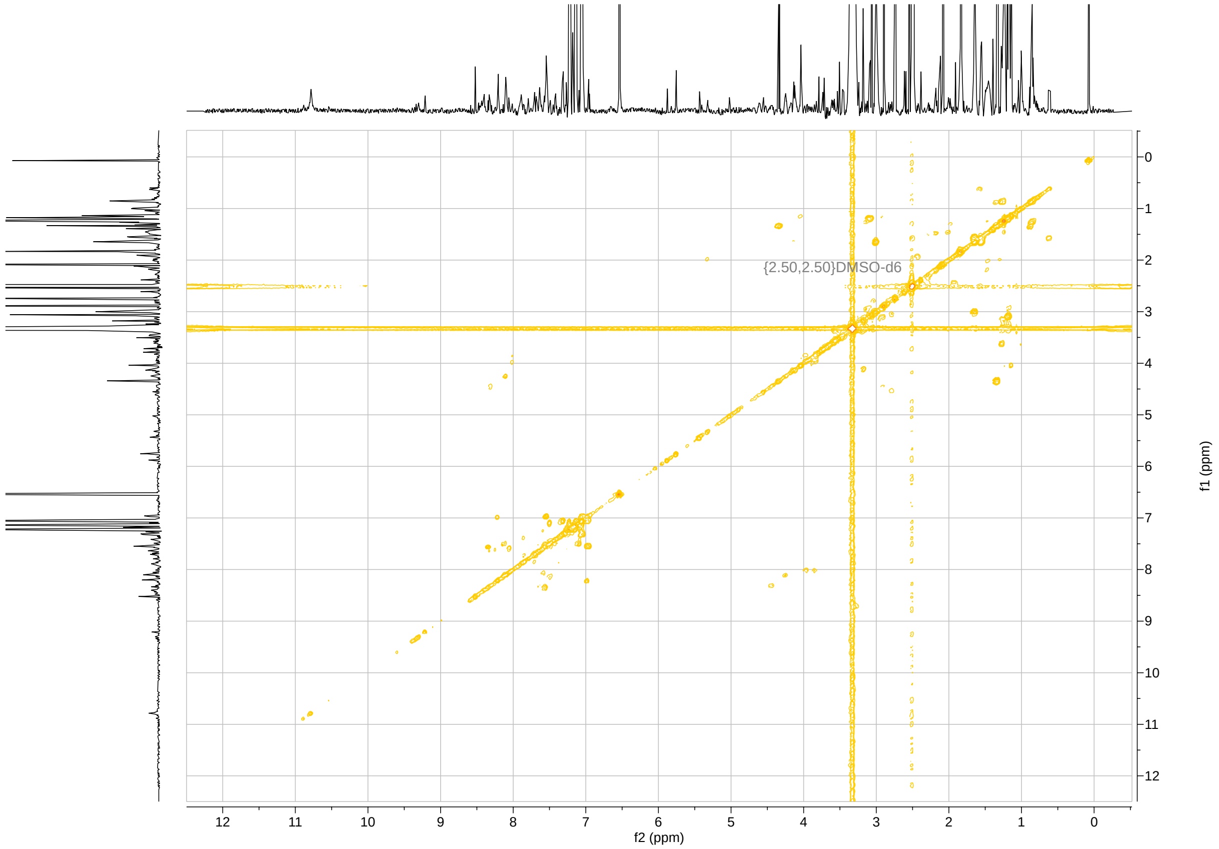

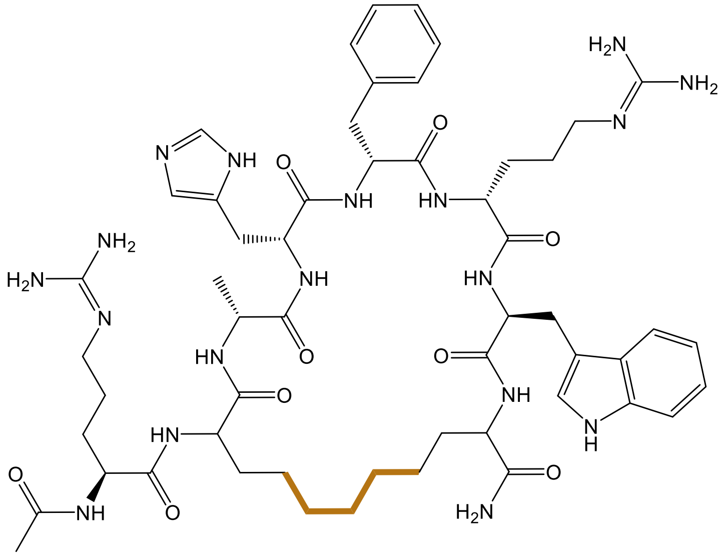

Set-C_5, diastereomer **ii**, ^1H-^1H NOESY

600 MHz, DMSO-d_6

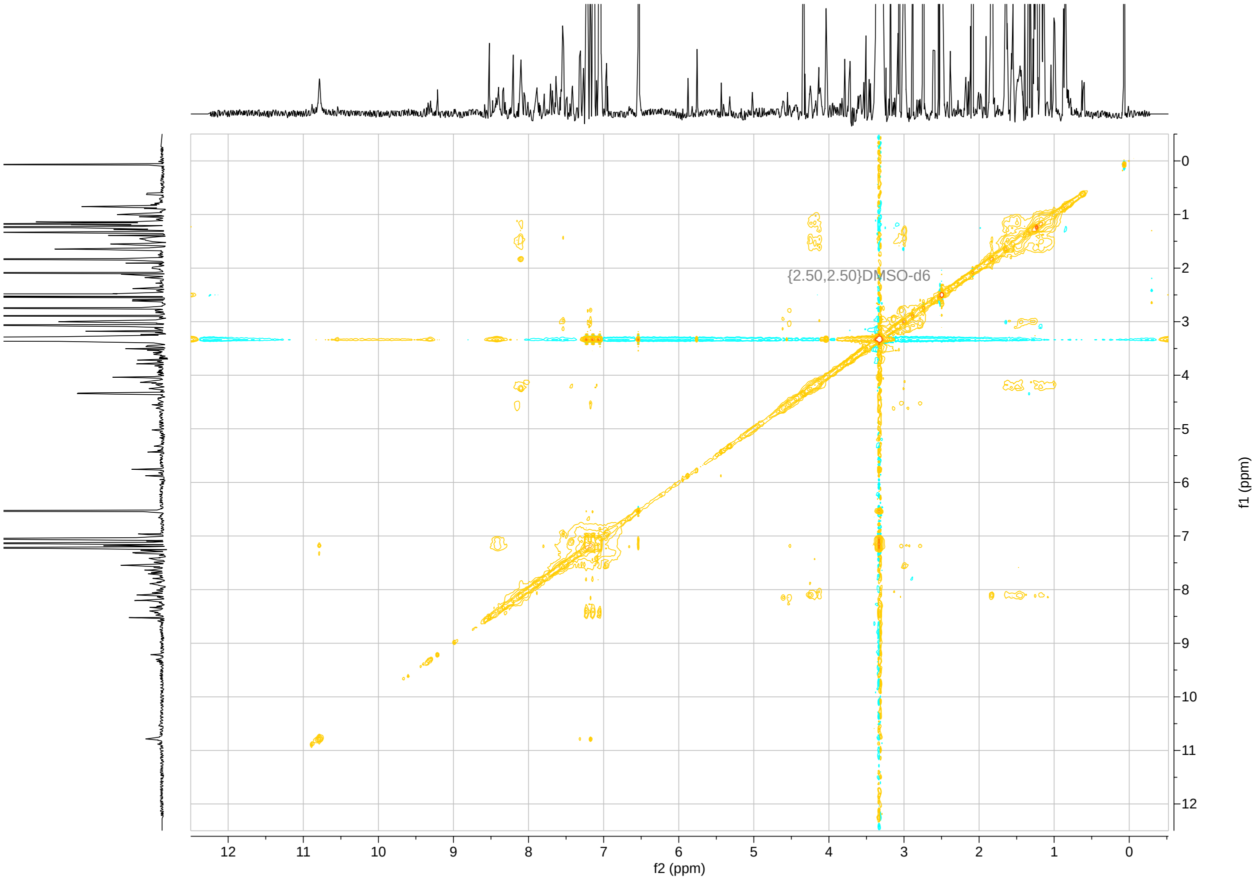

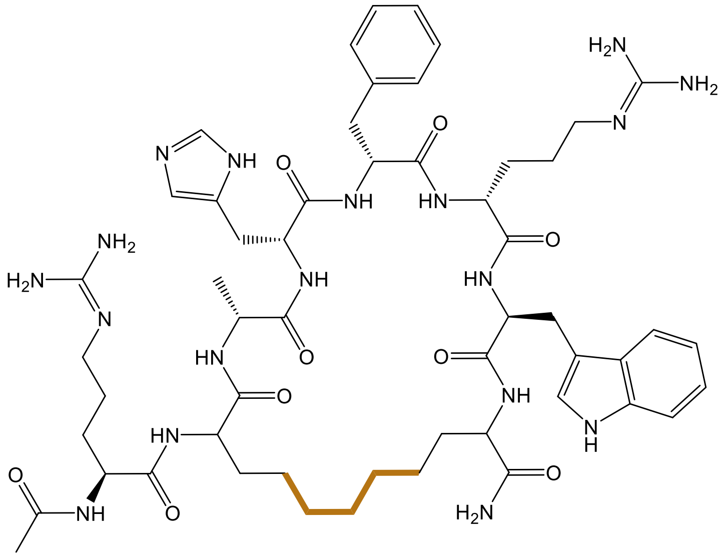

References

1. Morrison, P. M.; Foley, P. J.; Warriner, S. L.; Webb, M. E., Chemical generation and modification of peptides containing multiple dehydroalanines. *Chemical Communications* **2015,** *51* (70), 13470-13473.
2. Xu, Y.; Xu, Z.-J.; Liu, Z.-P.; Lou, H., Visible-light-mediated de-aminative alkylation of N-arylamines with alkyl Katritzky salts. *Organic Chemistry Frontiers* **2019,** *6* (23), 3902-3905.
3. Hsiao, Y.-T.; Beadle, J.; Pascoe, C.; Annadate, R.; Vederas, J. C., Decarboxylative Radical Addition to Methylideneoxazolidinones for Stereocontrolled Synthesis of Selectively Protected Diamino Diacids. *Organic Letters* **2021,** *23* (18), 7270-7273.
4. Ngen, E. J.; Rajaputra, P.; You, Y., Evaluation of delocalized lipophilic cationic dyes as delivery vehicles for photosensitizers to mitochondria. *Bioorganic & Medicinal Chemistry* **2009,** *17* (18), 6631-6640.
5. Ohshita, J.; Iwata, A.; Kanetani, F.; Kunai, A.; Yamamoto, Y.; Matui, C., Ring-Opening Iodo- and Bromosilation of Cyclic Ethers by Treatment with Iodo- and Bromotrialkylsilane Equivalents. *The Journal of Organic Chemistry* **1999,** *64* (21), 8024-8026.
6. Keinan, E.; Perez, D.; Sahai, M.; Shvily, R., Diiodosilane. 2. A multipurpose reagent for hydrolysis and reductive iodination of ketals, acetals, ketones, and aldehydes. *The Journal of Organic Chemistry* **1990,** *55* (9), 2927-2938.
7. Ghiaci, M.; Sedaghat, M. E.; Kalbasi, R. J.; Abbaspur, A., Applications of surfactant-modified clays to synthetic organic chemistry. *Tetrahedron* **2005,** *61* (23), 5529-5534.
8. Petroselli, M.; Angamuthu, V.; Rahman, F.-U.; Zhao, X.; Yu, Y.; Rebek, J., Radical Reactions in Cavitands Unveil the Effects of Affinity on Dynamic Supramolecular Systems. *Journal of the American Chemical Society* **2020,** *142* (5), 2396-2403.
9. Petroselli, M.; Rebek Jr, J.; Yu, Y., Highly Selective Radical Monoreduction of Dihalides Confined to a Dynamic Supramolecular Host. *Chemistry – A European Journal* **2021,** *27* (10), 3284-3287.
10. Kida, T.; Kikuzawa, A.; Higashimoto, H.; Nakatsuji, Y.; Akashi, M., Synthesis of novel cyclodextrin derivatives by aromatic spacer insertion and their inclusion ability. *Tetrahedron* **2005,** *61* (24), 5763-5768.
